# Orpheovirus IHUMI-LCC2: A New Virus among the Giant Viruses

**DOI:** 10.3389/fmicb.2017.02643

**Published:** 2018-01-22

**Authors:** Julien Andreani, Jacques Y. B. Khalil, Emeline Baptiste, Issam Hasni, Caroline Michelle, Didier Raoult, Anthony Levasseur, Bernard La Scola

**Affiliations:** ^1^Aix Marseille Université, IRD, APHM, MEPHI, IHU-Méditerranée Infection, Marseille, France; ^2^Centre National de la Recherche Scientifique, Marseille, France

**Keywords:** Orpheovirus, Cedratvirus, Pithovirus, *Vermamoeba vermiformis*, giant viruses, NCLDV, Orpheoviridae, *Pithoviridae*

## Abstract

Giant viruses continue to invade the world of virology, in gigantic genome sizes and various particles shapes. Strains discoveries and metagenomic studies make it possible to reveal the complexity of these microorganisms, their origins, ecosystems and putative roles. We isolated from a rat stool sample a new giant virus “Orpheovirus IHUMI-LCC2,” using *Vermamoeba vermiformis* as host cell. In this paper, we describe the main genomic features and replicative cycle of Orpheovirus IHUMI-LCC2. It possesses a circular genome exceeding 1.4 Megabases with 25% G+C content and ovoidal-shaped particles ranging from 900 to 1300 nm. Particles are closed by at least one thick membrane in a single ostiole-like shape in their apex. Phylogenetic analysis and the reciprocal best hit for Orpheovirus show a connection to the proposed *Pithoviridae* family. However, some genomic characteristics bear witness to a completely divergent evolution for Orpheovirus IHUMI-LCC2 when compared to Cedratviruses or Pithoviruses.

## Introduction

‘Giant viruses’ is a name commonly given to all viruses which are characterized by a capsid or ovoid shape, a size larger than 0.2 μm and a genome containing more than approximately 200,000 base pairs. This term encompasses a monophyletic group of large double stranded DNA viruses known as the nucleo-cytoplasmic large DNA viruses (NCLDV) (Iyer et al., 2001). The discovery of many Mimiviruses (La Scola et al., 2003; Arslan et al., 2011), Marseilleviruses (Boyer et al., 2009; Dornas et al., 2016) and Pandoraviruses (Philippe et al., 2013; Antwerpen et al., 2015), broke the paradigm of the previously held definition of the frontier between prokaryote and viruses. The “Megavirales” order proposed by Colson et al. (2013) continues to expand to host new arrivals with the potential of replacing the current NCLDV families (Colson et al., 2013; Aherfi et al., 2016). All these viruses share fundamental genes, for example, the conserved five ancestral genes and some others established into clusters of orthologous genes named NCVOGs (Yutin et al., 2009). Their replicative strategies appear to have adapted through their own evolution, as is the case for *Pandoraviruses* or *Mollivirus sibericum* (Abergel et al., 2015; Colson et al., 2017). Major improvements in taxonomy would be needed to definitely classify viruses in their families and in the putative “Megavirales” order. Further investigations should be focused on their genome content, hosts, ecosystems, tropisms and infectivity in order to determine whether their evolution is expansive or reductive or if it happens in a more dynamic accordion-like pattern (Moreira and Brochier-Armanet, 2008; Filée, 2014, 2015; Yutin et al., 2014; Moreira and López-García, 2015).

For now, co-culture on amoeba remains the major tool for isolating giant viruses (Pagnier et al., 2013; Khalil et al., 2016a). We recently combined co-culture with flow cytometry to come up with a faster and more sensitive way of detecting, presumably identifying and purifying the causative agent of lysis (Khalil et al., 2016b, 2017). In 2013, *Pithovirus sibericum* was isolated from a 30,000-year-old sample in the Siberian permafrost, and was described as being the most elongated-ovoid shape currently known for a virus with a maximum length of 1.5 μm. Surprisingly, the circular genome size is “only” of 610,033 base pairs, which appears to be astonishing given their viral particle size. The genome of *P. sibericum* is delivered via a single cork. Two years after the description of Pithovirus, a modern one that we named “*Pithovirus massiliensis LC8”* (Levasseur et al., 2016a) was also isolated and displayed amazing and extreme genomic conservation regarding its ancestor *P. sibericum*, which enabled us to estimate a molecular clock about the evolution of Pithoviruses. Moreover, we recently described a new virus Cedratvirus A11 (Andreani et al., 2016) a possible new genus in the putative *Pithoviridae* family. This virus presented two corks, one at each extremity and a circular genome estimated at 589,068 base pairs. In addition, a new strain, close to Cedratvirus A11, known as *Cedratvirus lausannensis*, was recently isolated (Bertelli et al., 2017) with a genome size estimated at 575,161 base pairs. This latter appears to represent a fourth member of this new emerging family. Our isolated Faustovirus (Bou Khalil et al., 2016) and Pithovirus (Levasseur et al., 2016a) indeed came from the same sampling area. For this reasons and after successfully isolating these viruses, we decided to investigate the same location once again, 4 months later in order to search for the same isolates that could be circulating and to explore the relation to ecosystemic or environmental changes. The result of this work was a new isolate from rat stool sample, which we named Orpheovirus, the genome and replicative cycle features of which we describe in this paper.

## Materials and Methods

### Sample Collection

Twelve different rat stools and nine water samples contaminated by proximity sewage were collected. Rat stools were taken from a dry place one meter from the water sample area. Samples were harvested in November 2015 in La Ciotat, France, at the same GPS location where *P. massiliensis* LC8 (Levasseur et al., 2016a) and Faustovirus LC9 samples had been collected (Bou Khalil et al., 2016; Cherif Louazani et al., 2017) (N43.181834, E5.614423).

### Virus Isolation

*Vermamoeba vermiformis* stain CDC19 was used as cell support. The amoebas were harvested after 48 h of culture in homemade peptone yeast extract glucose medium (PYG) when a concentration of 1.10^6^ amoebas/mL was reached. Cells were then rinsed twice in homemade page’s amoeba saline (PAS) and pelleted at 700 × g for 10 min. The amoebas were then re-suspended in the starvation medium (Bou Khalil et al., 2016) at a concentration of 1.10^6^ amoebas/mL. An antibiotic and antifungal mixture with vancomycin (10 μg/mL), ciprofloxacin (20 μg/mL), imipenem (10 μg/mL), and voriconazole (20 μg/mL) was added to the suspension in order to decrease or eliminate bacterial or fungal contamination. A cell suspension of 250 μL per well was then distributed onto a 48-well plate. The samples were then vortexed and 50 μL were added to each well. The rest of the wells served as negative controls by adding 50 μL of PAS. The plate was incubated at 30°C for 4 days in order to monitor any potential cytopathic effect. This co-culture was repeated twice in the same order. When confronted with a high degree of contamination detected in some wells, filtration using 1.2 μm syringe filter (Merck Millipore) was carried out and gentamycin (20 μg/mL) was added 24 h before the second plate of co-culture (sub-culture 1).

### Viral Production and Purity Control

End-point dilution was performed in order to clone the virus before its production. To do so, we successively inoculated diluted viral supernatant on *V. vermiformis* at a dilution factor of 10. End point dilution was assessed for 5 days and the lysis was controlled by inverted microscopy.

For the production and purification processes, 14 infected flasks of 150 cm^2^ (Corning^®^, Corning, NY, United States) were pelleted using the Beckman coulter^®^ centrifuge Avanti^®^ J-26 XP (Beckman, France) at 14,000 ×*g* for 30 min (Andreani et al., 2016; Levasseur et al., 2016a). A 25% sucrose gradient was used for the final purification step. After finalizing production, we proceeded with genome sequencing.

### Genome Sequencing

Genomic DNA was sequenced on the MiSeq Technology (Illumina Inc., San Diego, CA, United States) using the paired end and mate pair applications. The DNA was barcoded in order to be mixed with 11 other projects for the Nextera Mate Pair sample prep kit (Illumina) and with 16 other projects for the Nextera XT DNA sample prep kit (Illumina).

gDNA was quantified using a Qubit assay with the high sensitivity kit (Life Technologies, Carlsbad, CA, United States) to 131.3 ng/μl.

For the paired end library, dilution was performed requiring 1ng of each genome as input. The “tagmentation” step fragmented and tagged the DNA. Limited cycle PCR amplification (12 cycles) then completed the tag adapters and introduced dual-index barcodes. The library profile was validated on an Agilent 2100 BioAnalyzer (Agilent Technologies Inc., Santa Clara, CA, United States) with a DNA High sensitivity labchip and the fragment size was estimated to 1.5 kb. After purification on AMPure XP beads (Beckman Coulter Inc., Fullerton, CA, United States), the libraries were then normalized on specific beads according to the Nextera XT protocol (Illumina). Normalized libraries were pooled for MiSeq sequencing. Automated cluster generation and paired end sequencing with dual index reads were performed in a single 39-h run in 2 × 250-bp.

A total of 6.6 Gb of information was obtained from a 697,000 per mm^2^ for the density cluster with a cluster passing quality control filters of 94.6% (12,733,000 passed filtered clusters). Within this run, the index representation for Orpheovirus IHUMI-LCC2 was determined to 12.93%. The 1,942,146 paired end reads were trimmed and filtered according to the read qualities.

The mate pair library was prepared with 1.5 μg of genomic DNA using the Nextera mate pair Illumina guide and two libraries were constructed. The genomic DNA sample was simultaneously fragmented and tagged with a mate pair junction adapter. The pattern of the fragmentation was validated on an Agilent 2100 BioAnalyzer (Agilent Technologies Inc., Santa Clara, CA, United States) with a DNA 7500 labchip. The DNA fragments ranged from 1.5 kb to 11 kb with an optimal size at 6.57 and 2.89 kb, respectively. No size selection was performed and 600 and 117 ng, respectively, of tagged fragments were circularized.

The circularized DNA was mechanically sheared to small fragments with an optimal size of 1029 and 1253 bp, respectively, on the Covaris device S2 in T6 tubes (Covaris, Woburn, MA, United States).

The library profile was visualized using a High Sensitivity Bioanalyzer LabChip (Agilent Technologies Inc., Santa Clara, CA, United States) and the final concentration libraries were measured at 5.13 and 5.4 nmol/l, respectively.

In each construction, the libraries were normalized at 2 nM and pooled. After a denaturation step and dilution at 15 pM, the pool of libraries was loaded onto the reagent cartridge and then onto the instrument along with the flow cell. Automated cluster generation and sequencing run were performed in a single 39-h run in a 2 × 151-bp.

Total information of the two flowcells at 6.2 and 7.9 Gb was obtained from a 648,000 and 863,000 cluster density per mm^2^ with a cluster passing quality control filters of 96.1 and 94% (12,144,000 and 15,627,000 passing filter paired reads). Within these runs, the index representation for Orpheovirus IHUMI-LCC2 was determined at 3.16 and 12.43%. The 725,401 and 1,942,196 paired reads were trimmed and assembled with the paired end reads.

### Genome Assembly

Mate pair and paired-end reads were trimmed using CLC Genomics Workbench v7.5^1^. *De novo* assembly of all reads was conducted using 64-word size and 50 bubble size parameters. We obtained 20 scaffolds representing a total size 1,461,620 bp with an average coverage reads ranged from 423 to 551. In parallel, we used an A5 pipeline assembler (Tritt et al., 2012) with standard parameters on 3,884,384 raw reads (paired end reads) representing 621,103,741 nucleotides. We obtained one scaffold of 1,473,699 with a median coverage reads of 295 with a 10th percentile at a coverage of 226. However, two regions of repeats were not completely resolved. Blast alignments of the two different assembling strategies confirmed these two regions and also underlined a high degree of identity between the two methods of assembly (>99%). For these two regions on the A5 assembly, we used GapCloser (Luo et al., 2012) and GapFiller (Nadalin et al., 2012) to fill two gaps and obtained a final single scaffold of 1,473,573 base pairs.

### Genome Alignments and Genome Organization

The MAUVE program (Darling et al., 2004) was used to align and determine nucleotide divergence between genomes. BLAST nucleotide online was used to generate dot plots to explore large repeats in the whole genome and in all specific coding sequences. Emboss Explorer was used online using the following different software programs: palindrome of a 200 maximum length^2^, an e-inverted program, an equicktandem for a fast detection.

### Genome Analysis

Gene prediction was computed using Genemarks software (Besemer et al., 2001). We deleted predicted proteins having a size less than 50 amino acids, and 85 predicted protein from 50 to 99 amino acids were detected by Phyre2 (Kelley et al., 2015) as having abnormal tri-dimensional folding and finally were discarded from our dataset. A Blast protein was performed against the non-redundant (nr) protein database (June 19, 2017). Annotation was performed using a combination of Interpro^3^ version 63.0, a CD-search tool online (Marchler-Bauer and Bryant, 2004) and delta-blastp (Boratyn et al., 2012). Interpro detected 100 transmembrane domain-containing proteins, and with CD-search and delta-blastp they congruently identified domains in 443 proteins.

tRNA prediction was computed online^4^ (Lowe and Chan, 2016) following different standard parameters successively with eukaryotes, archaea and bacteria. We identified orthologous and paralogous genes by using Proteinortho v5 (Lechner et al., 2011) with 60% coverage and 20% amino acid identity and an *e*-value of 10^-2^ as significance thresholds. Moreover, we generated pan-genomic tree on GET_HOMOLOGUES package (Contreras-Moreira and Vinuesa, 2013) using OrthoMCL algorithm with the standard parameters expected for the coverage and *e*-value. We choose 60% as minimum coverage in Blastp pairwise alignments and 1 × 10^-2^ as maximum *e*-value.

### Genome Submission

Orpheovirus IHUMI-LCC2 is available in the EMBL-EBI database under accession number LT906555.

### Phylogenetic Analysis

All phylogenetic analyses were conducted using the following procedures. Blastp was used to find close homologous proteins. Then, the MUSCLE program (Edgar, 2004) was used to align amino acid sequences. The FastTree program (Price et al., 2009) was computed with standard parameters using the maximum likelihood method with 1,000 bootstrap replicates and the Jones–Taylor–Thornton (JTT) model for amino acid substitution. Phylogenetic trees were then visualized using iTOL v3 online (Letunic and Bork, 2016).

## Results

### Virus Isolation

**FIGURE 1:**
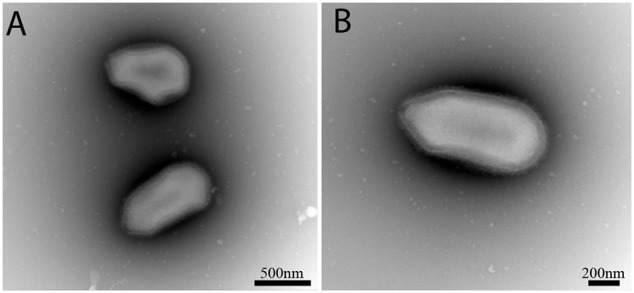
Negative staining obtained from the co-culture supernatant of well LCC2. Scale bars are indicated on each panel. **(A)** Two specimens of Orpheovirus, the top one has a curious irregular form with highly concave forms. **(B)** Classic ovoid shape of Orpheovirus IHUMI-LCC2.

Bacterial contamination is common in viral co-cultures when using stool and sewage samples, with the frequent presence of resistance to the antibiotics and anti-fungal mixtures used. For this, we used a classic mix of antibiotics notably, vancomycin, ciprofloxacin, and imipenem, as previously reported. However, we added gentamicin to our first sub-culture plate, 24 h before inoculating the new plate in order to eliminate resistant bacteria from the stool samples. After three passages on *V. vermiformis*, lysis referring to the cytopathic effect was detected in some wells. We performed negative staining on the supernatant of a rat stool in well LCC2 and observed particles with an elongated aspect (**Figure 1**), some of them appear to be irregular, with a concave shape compared to *Pandoravirus* and *Pithoviruses.* In contrast, the apex appears to be more similar to Pandoravirus. We named it Orpheovirus.

### Replicative Cycle

**FIGURE 2:**
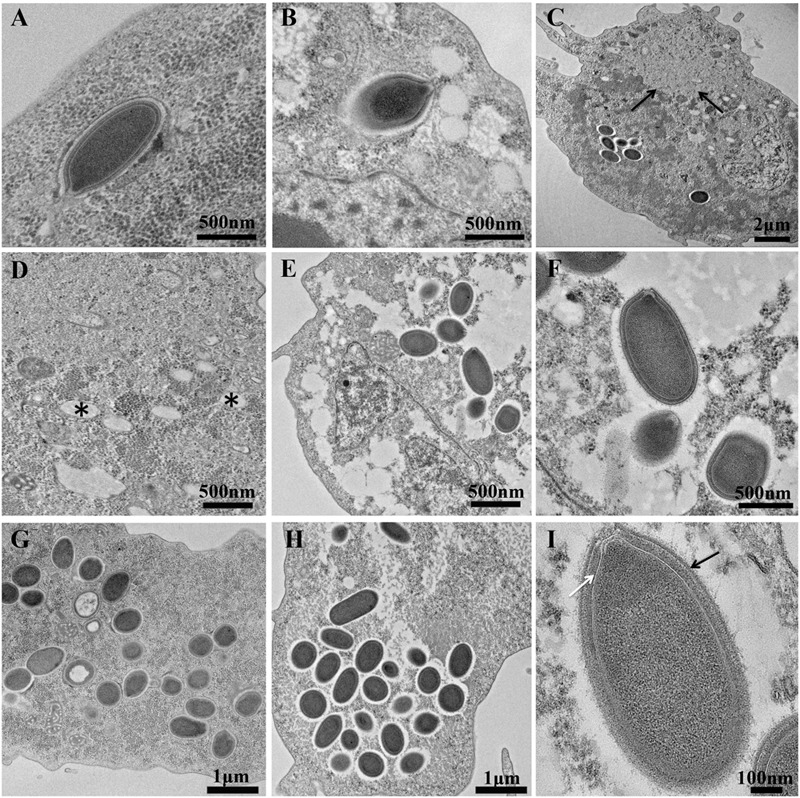
Ultrathin sections of Orpheovirus’s replicative cycle. Scale bars are indicated on each panel. **(A,B)** Represent viral entry at 2 h and 4 h post-infection. **(C)** Represents a section of *Vermamoeba vermiformis* 16 h post-infection, Black arrows delimitate the viral factory. **(D)** High magnification of **(C)** picture, (^∗^) represents some curious vacuoles in contact with the viral factory in the cytoplasm. **(E–G)** Show some cytoplasms and new virus synthetized 20 h post-infection. **(H)** Accumulation of assembled virions at 20 h post-infection. **(I)** Single virion into the cytoplasm of *V. vermiformis* at 24 post-infection, Black arrow points to the external membrane and white arrow indicates the medium dense space.

The length of the Orpheovirus virions range from 900 to 1,100 nm (*N* = 10) with a maximum diameter of about 500 nm (*N* = 13). Some virions could reach 1,300 nm in length; this process was sometimes observed in the host cytoplasm. The cork does not seem to seal by a grid as opposed to Pithoviruses and Cedratviruses. We noticed shapes which were similar to the ostiole-like apex observed in Pandoraviruses (Philippe et al., 2013) with a diameter ranging from 70 to 80 nm (*N* = 8) obstructed by a thick membrane (**Figures 2F,I**). Nevertheless, in Pandoraviruses the tegument is composed of three layers, each measuring about 20 ∼ 25 nm. The Orpheovirus’ particles presented a dark dense outside layer coated with short, sparse fibrils on their external surface (black arrow). This dark layer is followed by a medium dense space (white arrow) which is in direct contact with the thin inner hyperdense membrane surrounding the viral core cavity containing the nucleic acid (**Figure 2I**). Altogether, these layers measure ≈40 nm. The replicative cycle of Orpheovirus showed classical stages of infection and replication in amoeba. Briefly, the virus entry by phagocytosis is the start of the cycle, where particles escape the phagosomal process. DNA delivery occurs in the amoeba cytoplasm via the ostiole-like apex (**Figures 2A,B**). An eclipse phase takes place at 4 h post entry. Functional viral factories (**Figure 2C**) are well installed and detected around 14–16 h post-infection. Similar forms corresponding to early virion synthesis are also observed, as it is the case for Pithoviruses and Cedratviruses (**Figure 2D**). At 20 h post-infection, the host cells’ cytoplasm is fully occupied by newly synthesized virions (**Figures 2E,G,H**). We were also able to detect viruses outside the amoeba due to cell burst or viruses exiting by exocytosis. Complete cell burst occurred between 24 and 38 h post-infection. This slow viral cycle is often observed in the case of *V. vermiformis* used as cell support, which is not the case when using *Acanthamoeba* spp. (Reteno et al., 2015; Andreani et al., 2017).

### Orpheovirus: Main Genomic Characteristics

Orpheovirus has a circular genome estimated at 1,473,573 base pair (including 100 N due to an incomplete elucidate region in its genome) with a GC%-content established at around 25% (**Table 1**). A megablast or a simple blastn against the nr/nt nucleotide collection database revealed no match with other known giant viruses. Dot plots show various areas of repeats (Supplementary Figures S1–S3). We found 57 palindromic sequences, 1,527 tandem repeats and 832 inverted sequence candidates. The number of repeats explains the complexity observed during the genome assembly steps. A comparison with other giant viruses (Supplementary Table S1) showed an extremely high number of tandem repeats and inverted repeats for Orpheovirus.

**Table 1:** Main genomic characteristics of Orpheovirus and other closely related viruses.

Virus	Orpheovirus IHUMI-LCC2	*Cedratvirus lausannensis* CRIB-75	Cedratvirus A11	*Pithovirus massiliensis* LC8	*Pithovirus sibericum*
Morphological features	Ovoid, single ostiole-like	Ovoid double corks	Ovoid double corks	Ovoid single cork	Ovoid, single cork
Genome size (bp)	1,473,573	575,161	589,068	686,015	610,033
GC content (%)	24.98	42.8	42.6	35.4	35.8
tRNA	0	0	0	0	0
Predicted proteins	1199	643	574	476	467
ORFans (%)^1^	≈66	≈45	≈35	N/A^2^	67.5
Coding density	66.4%	83%	78.5%	64%	69%

*^1^ORFans are given at the moment of viral description with an *e*-value at 10^-*5*^. ^2^N/A, not applicable. All ORFans of *P. sibericum* are found in *P. massiliensis* (Levasseur et al., 2016a).*

**FIGURE 3:**
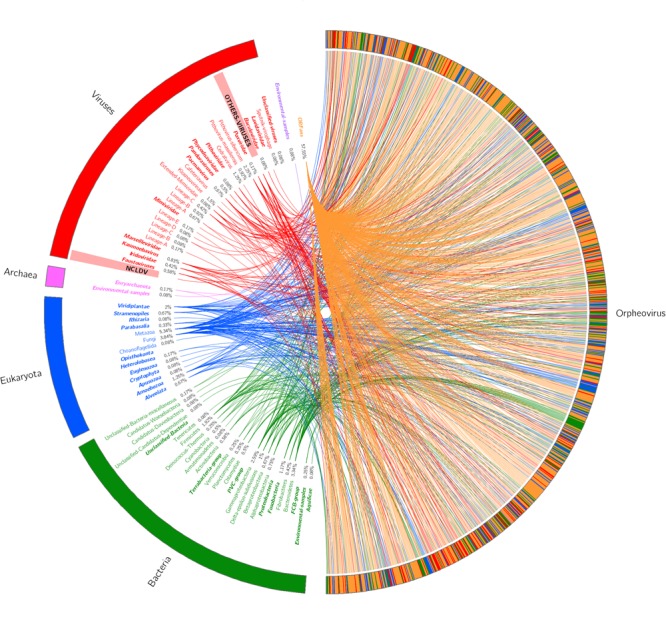
Best hit schema obtained for Orpheovirus IHUMI-LCC2 against the non-redundant database.

1,512 genes were predicted but, following our method, 313 genes with an abnormal conformation already cited in the material and methods section were discarded. We only retained 1,199 genes, resulting in a coding density of around 66.4% (979,005 base pairs). This value is close to that of Pithoviruses but lower than that of Cedratvirus A11. A Blast against the nr database retrieved 509 matched proteins with at least one known protein (≈42.5% of all predicted proteins), and 690 unmatched, which are classified like ORFans genes (≈57.5% of all predicted genes). Of the 509 proteins, two had a hit with unclassified sequences, 148 had a best hit with a virus (≈12.3% of all conserved proteins), 176 with eukaryotes (≈14.7%), and 183 with prokaryotes (≈15.3%) (**Figure 3**). Regarding the 148 best hits with viruses, we observed 27 best hits with *P. sibericum*, 11 with *P. massiliensis*, 15 with Cedratvirus A11, 24 with Mimivirus A, B, and C lineages, and 18 with *Klosneuvirinae*. Hence, the highest best hit viral was obtained with the putative family *Pithoviridae* with 53 best hits, although the value was also important with *Mimiviridae* and associated extend family.

Despite this, the 57.5% of Orpheovirus’ genes are ORFans, with an *e*-value cut-off of 10^-2^. This could increase at ≈66% when we chose a more stringent cut-off value for the blastp at 10^-5^. We found 343 genes that formed 167 clusters of paralogous genes in Proteinortho, regrouping few numbers of genes ranging from two to a maximum of four by cluster. Annotation of these clusters revealed predominant predicted proteins mainly as MORN-repeat (55 sequences), Ankyrin-repeat (37 sequences), and F-box domain-containing (81 sequences).

The Orpheovirus annotation presented translation system components as follows: eight aminoacyl tRNA synthetases (aaRS), four translation factors: three initiation factors and one release factor (Supplementary Table S2). Surprisingly, Orpheovirus didn’t present any tRNA. We used the aminoacyl tRNA synthetase, which appeared to be a good way to distinguish and classify some lineages and to describe hypothetical common ancestor (Abrahão et al., 2017; Schulz et al., 2017). The Glycyl-tRNA synthetase of Orpheovirus was found to branch with the Asgard Glycyl-tRNA synthetase, and not with Catovirus CTV1 nor Klosneuvirus KNV1 homologs. This Asgard superphylum described by metagenomic studies seems to be a controversial bridge between prokaryotes and eukaryotes (Spang et al., 2015; Da Cunha et al., 2017; Zaremba-Niedzwiedzka et al., 2017). Regarding the phylogenetic analysis of each tRNA synthetase (Supplementary Figures S4–S11), we observed different patterns for amino acyl tRNA synthetase. While some are monophyletic with other described giant viruses, others appear to be polyphyletic resulting from potential lateral gene transfer.

### Orpheovirus and Its Divergent Viral Neighborhood

First of all, we searched for five ancestral genes of NCLDV (Colson et al., 2013) encoding the major capsid protein, the helicase-primase (D5), the DNA polymerase elongation subunit family B, the DNA-packaging ATPase (A32), and the viral late transcription factor 3 VLTF3. Four of these genes were found with the exception of the A32-like packaging ATPase, which was absent in all four viruses (Legendre et al., 2014; Andreani et al., 2016). As reported for Cedratvirus A11, *P. massiliensis*, and *P. sibericum*, Orpheovirus presented two distinct RNA polymerase II subunit 1. Multiple ribonucleases such as Ribonuclease R, two Ribonuclease III and one ribonuclease HI were detected in Orpheovirus. Orpheovirus presented glycosyltransferase and numerous proteins involved in lipid pathways. We also identified two proteins presenting multiple fusion bacteria domains involved in Riboflavin (Vitamin B2) biosynthesis. Indeed, we observed in ORPV_596 Tri-functional domains of “Di-Hydro-Folate-Reductase/deoxycytidylate deaminase/Riboflavin biosynthesis protein RibD” presenting a homology with Indivirus ILV1. And the second protein is ORPV_666, annotated like Tri-functional domains “3,4 dihydroxy-2-butanone 4-Phosphate synthase/GTP cyclohydrolase II/Lumazine synthase (RibA+RibB+RibH)” presenting homologies with Indivirus ILV1, *Bacillus subtilis*, and *Acanthamoeba castellanii strain Neff*. Vitreschak (2002) demonstrated that Riboflavin operon gene fusion is frequently found in bacteria (Vitreschak, 2002).

**FIGURE 4:**
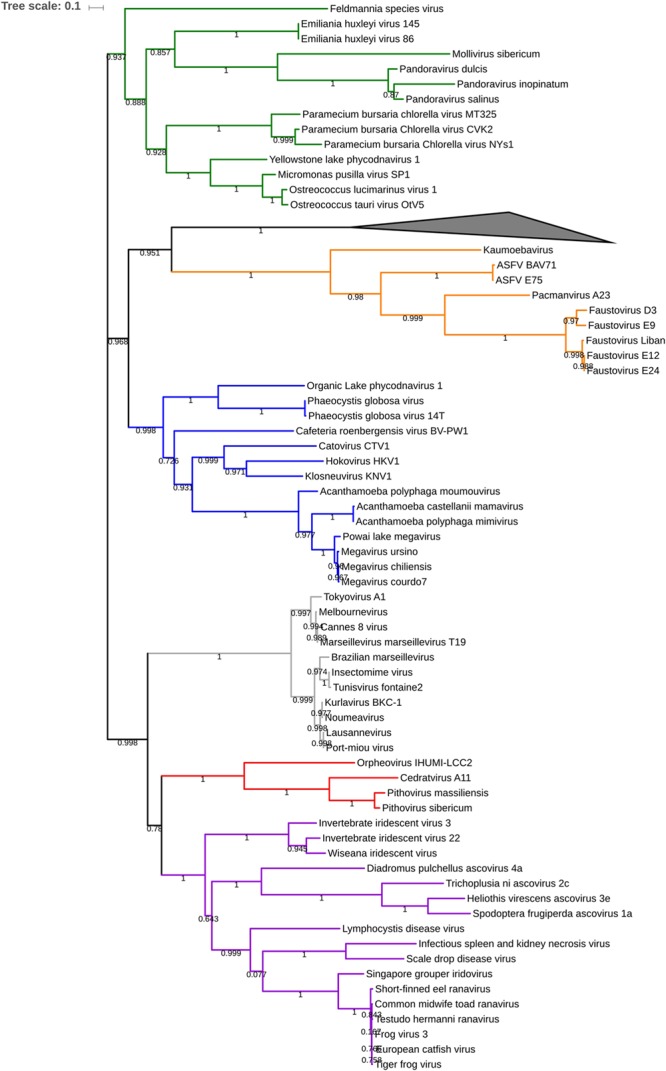
Phylogenetic tree based on 84 DNA polymerase b protein of nucleo-cytoplasmic large DNA viruses (NCLDV). Branch values lower than a bootstrap value of 0.5 were deleted. Colors were assigned for different group of viruses: blue for Mimivirus and extended Mimiviridae; green for Pandoraviruses, *Mollivirus sibericum* and Phycodnaviridae; orange for groups of Asfarviridae, Faustoviruses, Pacmanvirus and Kaumoabevirus; gray for *Marseilleviridae*; red for Orpheovirus, Cedratvirus, and Pithoviruses and purple for *Asco-Iridoviridae*. The collapsed branch represented by a black triangle was used for 15 *Poxviridae* members. The corresponding alignment is available on Supplementary Data Sheet 2 visualized by automatic MView software (https://www.ebi.ac.uk/Tools/msa/mview/). 3,450 positions were used to build the tree.

**FIGURE 5:**
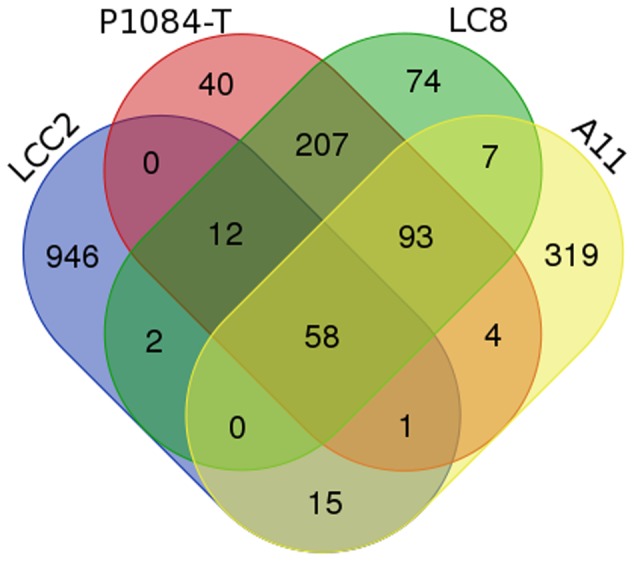
Venn diagram comparison of best reciprocal hit obtained belonging to the predicted proteins of Orpheovirus, Cedratvirus, Pithoviruses. LCC2 represents Orpheovirus IHUMI-LCC2, P1084-T represents *Pithovirus sibericum*, LC8 represents *Pithovirus massiliensis* and A11 represents Cedratvirus A11. The diagram was created online http://bioinformatics.psb.ugent.be/webtools/Venn/.

After that, phylogenetic analysis based on the DNA polymerase B protein, VLTF3 and RNA polymerase II subunit 1 showed deep branching with Cedratvirus and Pithoviruses (**Figure 4** and Supplementary Figures S12, S13). Moreover, 58 reciprocal best hit proteins were only shared between Orpheovirus IHUMI-LCC2, Cedratvirus A11, *P. sibericum P1084-T* and *P. massiliensis LC8.* In addition, 14 reciprocal best hit proteins were found to be shared between Orpheovirus and Pithoviruses (14+58) and 15 between Cedratvirus and Orpheovirus (15+58) (**Figure 5**), while Cedratvirus shared 151 proteins (58+93) with Pithoviruses. Meanwhile, 946 of 1,034 protein clusters (≈91.4%) are unique to Orpheovirus, 319 of 497 clusters (≈64.2%) to Cedratvirus A11, and 114 clusters of 543 (≈21%) to Pithoviruses. There were only two colinearity blocks and nine lines connecting Orpheovirus to other viruses (Supplementary Figure S14).

Following the discovery of this divergence between the four viruses, we decided to investigate Orpheovirus position in the “Megavirales” order further with the help of a parsimonious pan-genomic tree (Supplementary Figure S15). The long branch length observed for Klosneuvirus, *Pandoravirus inopinatum* and Orpheovirus is explained by the large genome size and by the number of predicted proteins compared to the close relative strains in the tree. These long branches could be a new common marker to explain the emergence of new viral family or lineages in the proposed “Megavirales” order. In the case of Orpheovirus, the pan-genomic analysis confirms this distant relation with the proposed *Pithoviridae* family.

### Orpheovirus and Virophage: A Curious Homologous Sequence

**FIGURE 6:**
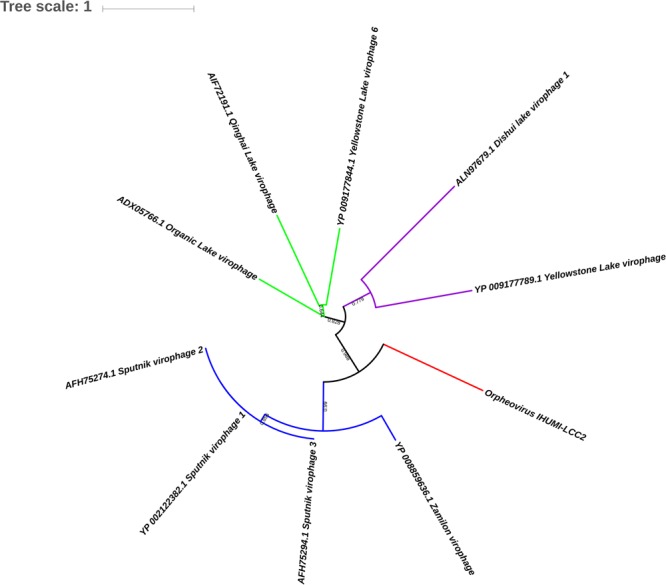
Phylogenetic tree based on V21-like protein of virophages and Orpheovirus IHUMI-LCC2 sequences. 484 positions were used to build the tree. Branch values inferior to a bootstrap value of 0.5 were deleted.

Orpheovirus has a predicted gene a 434 amino acids protein that we called V21-like protein. This protein had homologs in Blastp, respectively, at 86% coverage, 21% identity with Sputnik virophage V21 protein (La Scola et al., 2008), and 85% coverage, 27% identity with Zamilon (Gaia et al., 2014). These two homologous proteins are annotated as hypothetical proteins, and showed no other homology using the blast strategy. However, HHpred online (Supplementary Data Sheet 3) and Phyre2 (Supplementary Figure S16) detected homology between the V21-like Orpheovirus sequence, Sputnik virophage V21 protein, the Zamilon protein, and a putative transferase present in the genomes of Mimivirus lineages A, B, and C. This V21-like protein also shared a common ancestor with all Sputnik virophages, and Zamilon virophage (**Figure 6**). No transposase or other mobile elements could be detected, no other special interest homology with other proteins was detected although a Ribonuclease III such as that in MIMIVIRE (Levasseur et al., 2016b) was present near this V21-like sequence in the genome of Orpheovirus.

## Discussion

Since the isolation of Faustovirus in 2015, all positive samples have been sewages samples or samples collected near to sewage areas (Reteno et al., 2015; Benamar et al., 2016; Bou Khalil et al., 2016; Cherif Louazani et al., 2017). We suspected that rats could also be a potential reservoir of Faustoviruses. In order to decipher the Faustovirus’ reservoirs, and in attempt to study the viral frequency and persistence in the environment, notably during seasonality (Martínez et al., 2007; Johannessen et al., 2017), we decided to explore the same area of sampling 4 months later. We succeeded in re-isolating, in the same area, more Faustoviruses in sewage samples (data not shown) but not in rat stools samples, and a new giant virus was revealed, that we called Orpheovirus IHUMI-LCC2. This virus represents a new virus, the first to come with an ovoid form at a size higher than 1 μm isolated from *V. vermiformis* as a new host cell, and a genome of 1,473,573 bp largely exceeding the genomes of Cedratvirus A11, *P. massiliensis LC8*, and *P. sibericum.* Orpheovirus conserved a replicative cycle which is typical but delayed in terms of cell burst or complete lysis, which could be due to its host *V. vermiformis* showing different features regarding the routinely used host *Acanthamoeba* spp. (Andreani et al., 2017).

Although Orpheovirus appears to share some replicative elements and genomic bases with Cedratvirus A11, *P. massiliensis* LC8, and *P. sibericum*, some other elements highlighted a complete divergent evolution. With its genomic size, high number of paralogs, its eight aminoacyl tRNA synthetase aaRS, its low GC content, and its high number of ORFans (≈66% at a 10^-5^ *e*-value), we propose Orpheovirus as a potential member of a new putative family; the *Orpheoviridae* closely related to the recently proposed *Pithoviridae*. To do so, more viral descriptions including new isolates are needed to understand genomic links in these novel expanding and complex families. Nevertheless, some new viral descriptions such as that of Klosneuvirus (Schulz et al., 2017), have reported complete translational components, and this could create a broader understanding of the viral lifestyle and tRNA synthetase usages. In contrast, aminoacyl tRNA synthetases (aaRS) are frequently (but not entirely) found and described in isolated viruses (Abrahão et al., 2017), and we are still unaware of the viral benefits of possessing aminoacyl tRNA synthetase or tRNA during the infectious cycle. Simultaneously, and following the discovery of the MIMIVIRE system, it has become more widespread to search for virophages sequences in giant viruses genomes. We found a high conserved size and similar V21-like sequence in Orpheovirus that made us investigate the probability of an integrated virophage sequence in the Orpheovirus genome, as is the case for Mavirus (Fischer and Hackl, 2016) in its protist host *Cafeteria roenbergensis* or the Sputnik 2 virophage in the Lentille virus (Desnues et al., 2012). However, no mobile elements could be detected and no relationship could be found even when this sequence was closely located to the Ribonuclease III as is the case in MIMIVIRE. In contrast, the fact that the V21-like sequence of Orpheovirus, together with the V21 of Sputnik and Zamilon, showed homology with a putative transferase present in the genomes of Mimivirus lineages A, B, and C, led us to postulate that these sequences could either have a similar function to transferase or a protein that simply interacts with the putative transferase.

Despite all these findings, the description of Orpheovirus, along with the previous findings in Pandoraviruses, Pithoviruses, and Cedratviruses, has revealed a large range of viruses with various extraordinary ovoid shapes, which have expanded the research characteristics for viral isolation. Some more sewers should be investigated at different time stages or seasonal dates. In addition, animal stool samples should be more commonly considered as potential new reservoirs for giant viruses. Finally, a large part of this vast world of giant viruses is still unknown, particularly its evolution and ancestors. For this reason, more strains should be isolated and described, and more data is needed. It is likely that further descriptions will increase knowledge and diversity across the NCLDV.

## Author Contributions

JA and BL designed the study and experiments. JA, JK, EB, IH, CM, and AL performed the sample collection, virus isolation, experiments and/or analyses. JA, DR, and BL wrote the manuscript. All authors approved the final manuscript.

## Conflict of Interest Statement

The authors declare that the research was conducted in the absence of any commercial or financial relationships that could be construed as a potential conflict of interest.

## Footnotes

1 http://www.clcbio.com/blog/clc-genomics-workbench-7-5/

2 http://emboss.bioinformatics.nl/cgi-bin/emboss/palindrome

3 https://www.ebi.ac.uk/interpro/

4 http://trna.ucsc.edu/tRNAscan-SE/
