# Supplementary material for: Orpheovirus IHUMI-LCC2: A New Virus among the Giant Viruses
Source: Front Microbiol. 2018 Jan 22;8:2643. doi: 10.3389/fmicb.2017.02643 (PMC5786535; doi:10.3389/fmicb.2017.02643)
Supplement: Supplementary file 1 [file Data_Sheet_1.PDF]

Supplementary data: Orpheovirus IHUMI-LCC2: A new virus among the giant viruses.

Julien Andreani[1], Jacques Bou Khalil[1], Emeline Baptiste[1], Issam Hasni[1], Caroline Michele[1], Didier Raoult[1], Anthony Levasseur[1], Bernard La Scola[1#]

[1]Unité de Recherche sur les Maladies Infectieuses et Tropicales Emergentes (URMITE), UM63 CNRS 7278 IRD 198 INSERM 1095, IHU-Méditerranée Infection, 19-21 Boulevard Jean Moulin, 13005, Marseille, France[1]

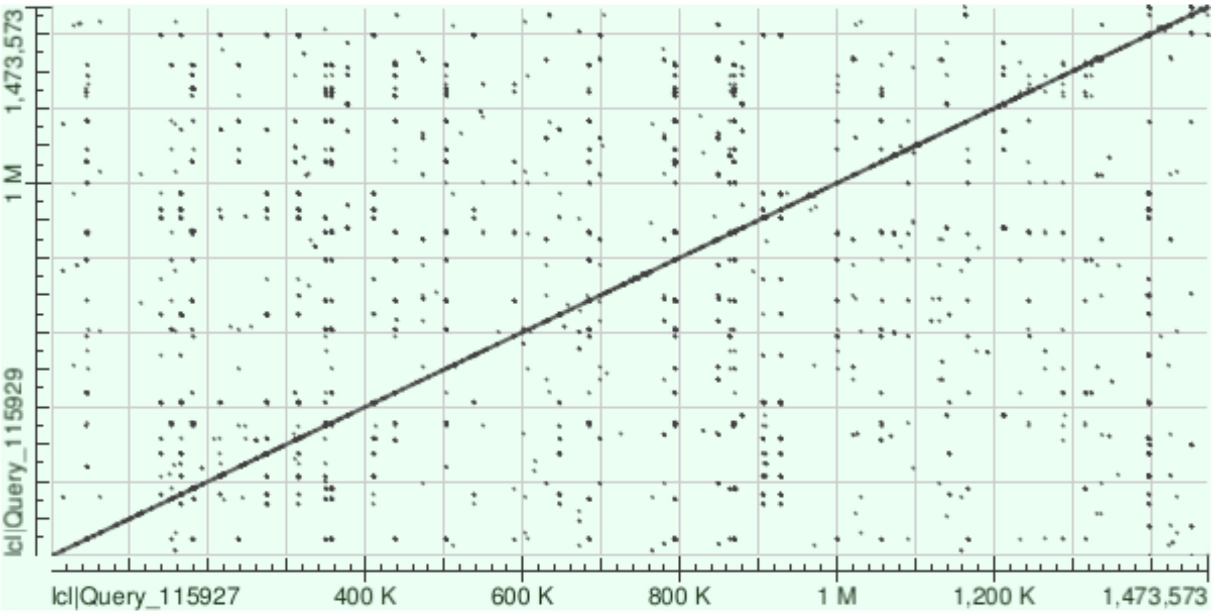

Supplementary figure S1: Dot plot of Orpheovirus complete genome (1,473,573 bp) in highly similar parameter (Megablast).

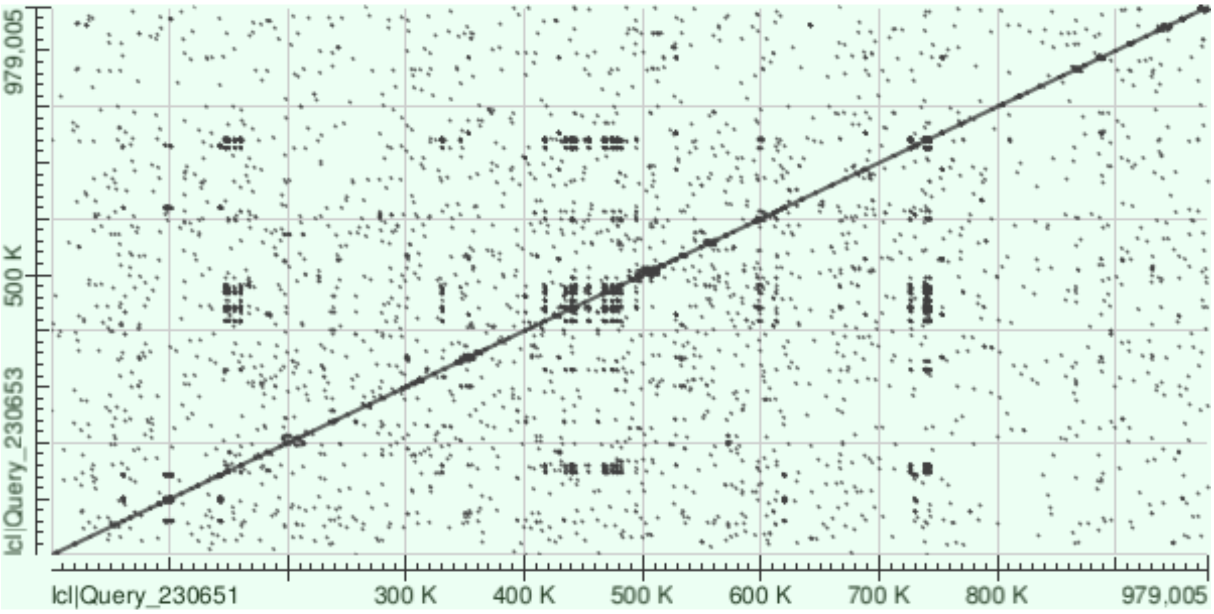

Supplementary figure S2: dot plot of all coding sequences of Orpheovirus IHUMI-LCC2 (979,005 bp) in somewhat similar: blastn

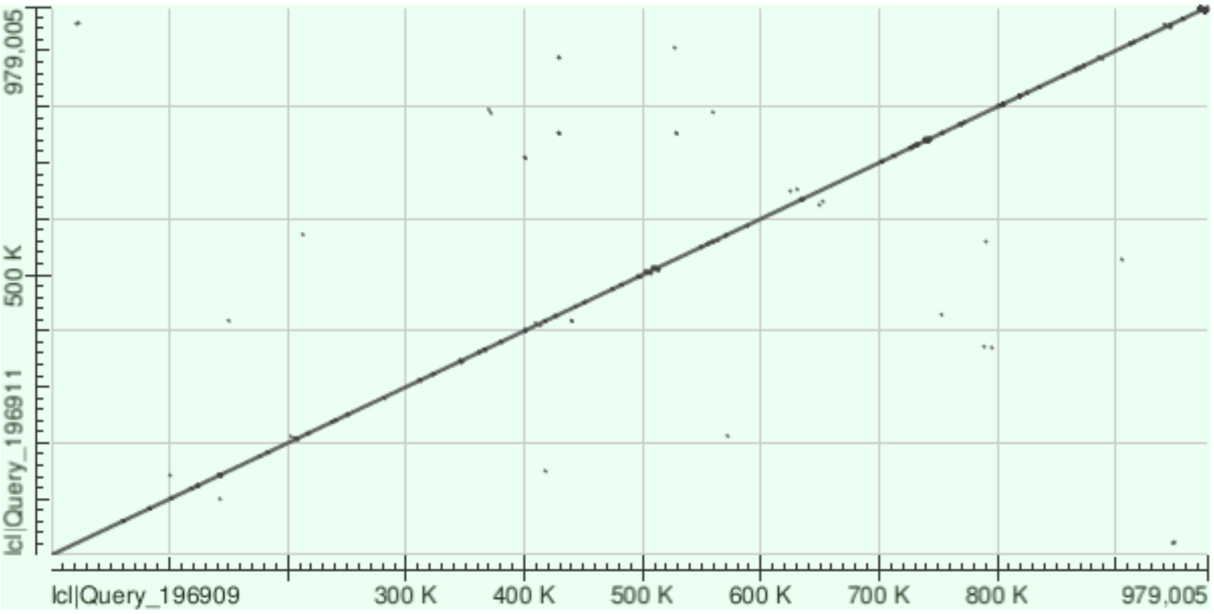

Supplementary figure S3: dot plot of all coding sequences of Orpheovirus IHUMI-LCC2 (979,005 bp) in highly similar parameter: Megablast.

Supplementary Table 1: Repeats chart comparison of some NCLDV.

Virus	Palindrome repeats	Tandem repeats	Inverted repeats	Genome size (Mb)
Orpheovirus IHUMI-LCC2	57	1527	832	1,47
Cedratvirus A11	3	400	135	0,58
Pithovirus massiliensis LC8	443	575	238	0,68
Pithovirus sibericum	478	448	189	0,61
Pandoravirus salinus	1	46	280	2,4
Acanthamoeba polyphaga mimivirus	0	24	158	1,29

Supplementary Table 2: Translation system comparison.

abbreviation	*Translation component system in Orpheovirus*	*Type*	*Other virus*
GlyRS	Glycyl-tRNA synthetase	aaRS	Klosneuvirinae
ArgRS	Arginyl-tRNA synthetase	aaRS	Mimivirus A,B,C; Klosneuvirinae
IleRS	Isoleucyl-tRNA synthetase	aaRS	Mimivirus B, C; CroV, Klosneuvirinae
PheRS	Phenylalanyl-tRNA synthetase	aaRS	Klosneuvirus KNV1
SerRS	Serine-tRNA synthetase	aaRS	Klosneuvirinae
AspRS	Aspartyl-tRNA synthetase	aaRS	Mimivirus B, C; Klosneuvirinae
HisRS	Histidine-tRNA synthetase	aaRS	Klosneuvirinae
TyrRS	Tyrosine-tRNA synthetase	aaRS	Pandoraviruses, Mimivirus A,B ,C; Klosneuvirinae
SUI1	Eukaryotic translation initiation factor SUI1	TF	Mimivirus A,B ,C; CroV; Mrs; Fstv
eIF2B-eIF5	Eukaryotic translation initiation factor eIF2B-eIF5	TF	Klosneuvirinae; Mrs
IF5A	Eukaryotic translation initiation factor IF5A	TF	*Aureococcus anophagefferens virus*
eRF1/aRF1	Eukaryotic peptide chain release factor subunit 1	TF	Klosneuvirinae; Mrs; Mimivirus A,B,C.

CroV: *Cafeteria roenbergensis virus*; Mrs: Marseillevirus; Fstv: Faustovirus.

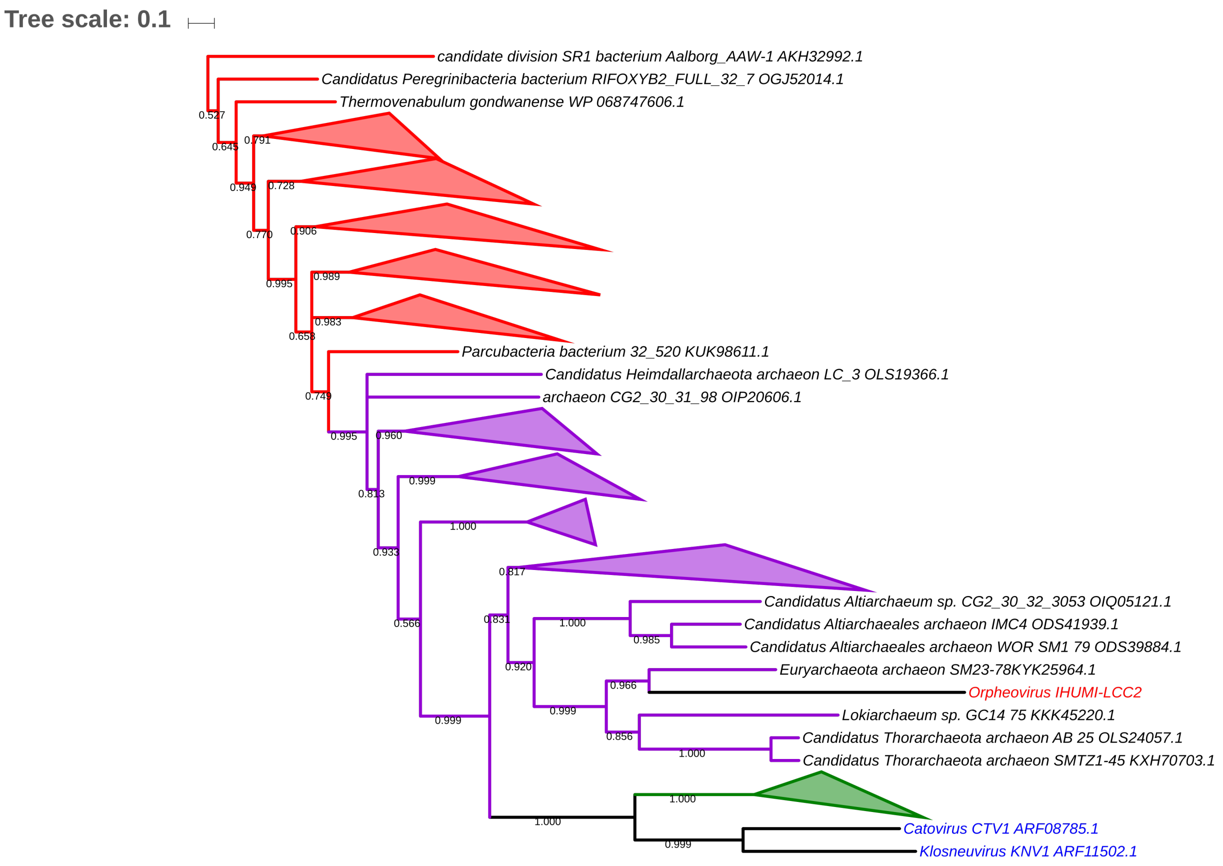

Supplementary figure S4: Phylogenetic tree based on Glycyl-tRNA synthetase. Branched with a bootstrap value under 0.5 were deleted. Bacteria are colored in red, archea and related sequences in purple and eukaryote in green.

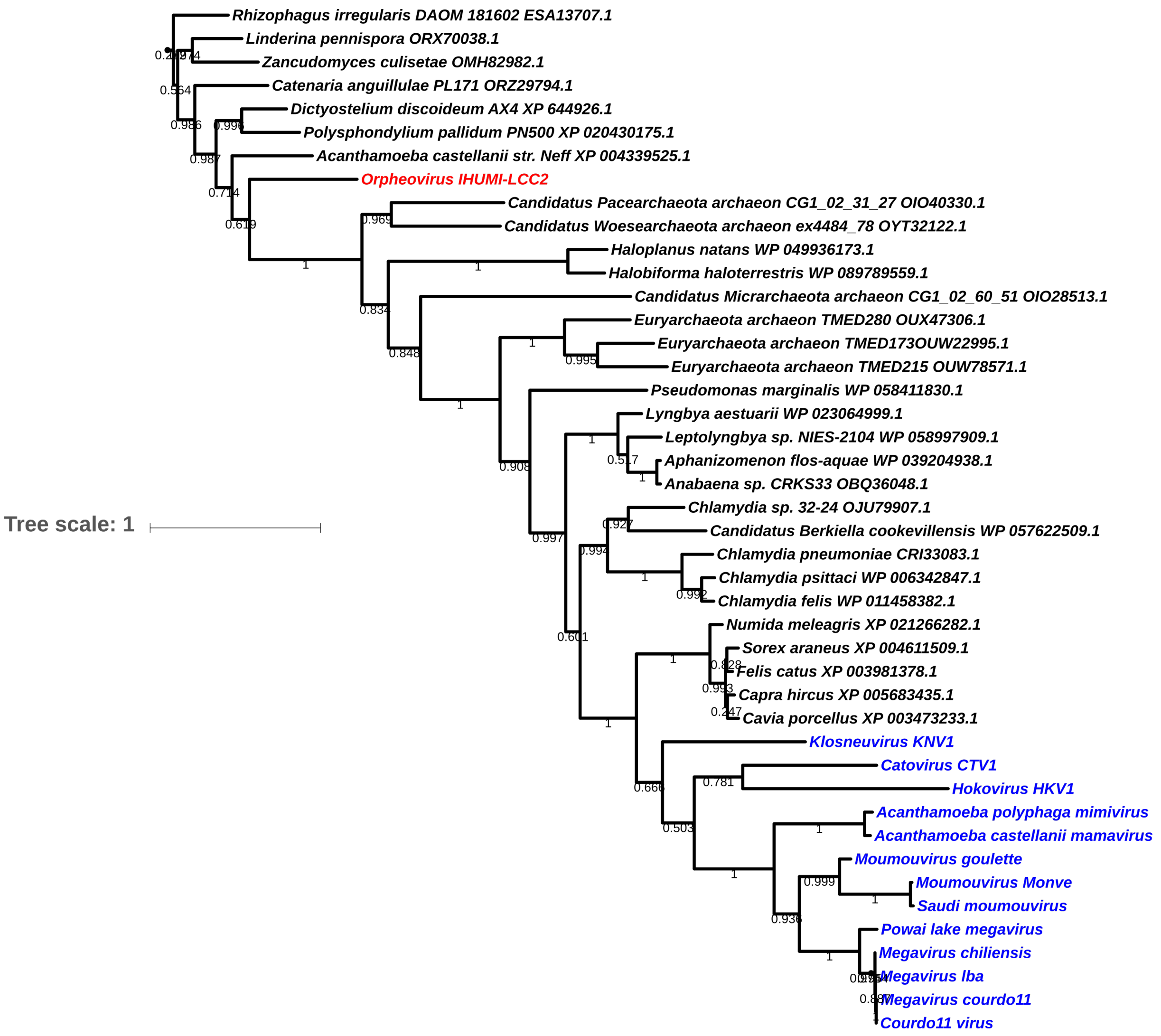

Supplementary figure S5: Phylogenetic tree based on Arginyl-tRNA synthetase.
Branched with a bootstrap value under 0.5 were deleted.

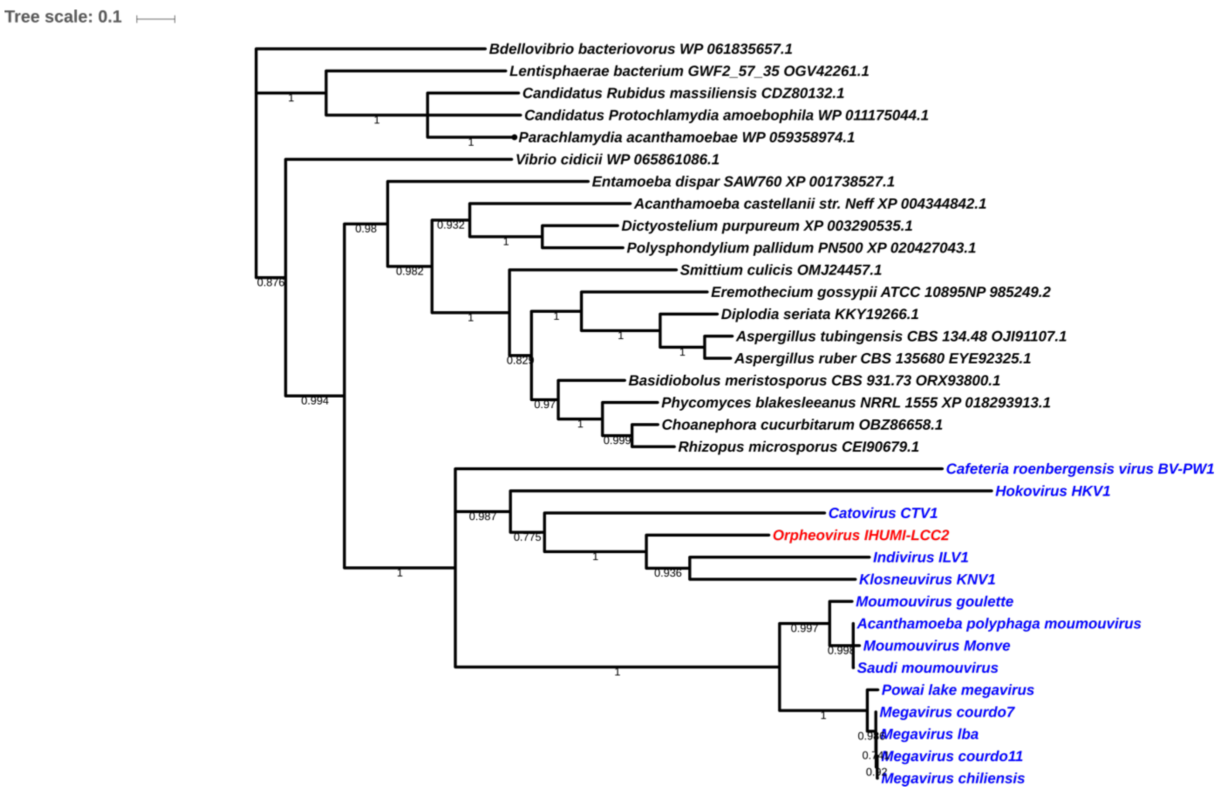

Supplementary figure S6: Phylogenetic tree based on Isoleucyl-tRNA synthetase.
Branched with a bootstrap value under 0.5 were deleted.

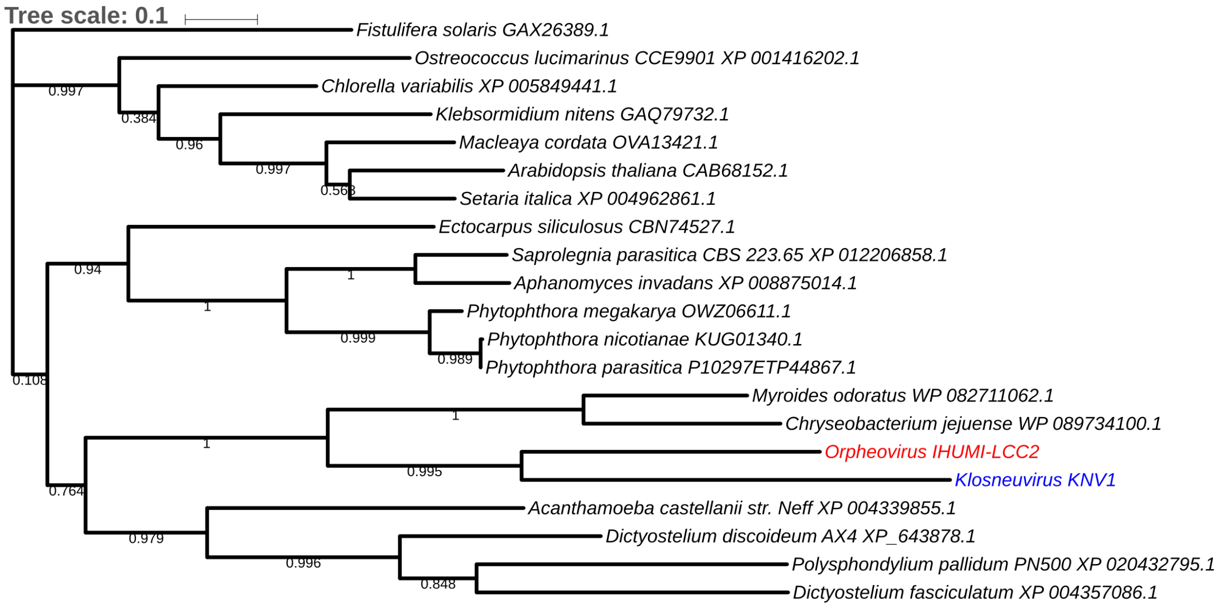

Supplementary figure S7: Phylogenetic tree based on Phenylalanyl-tRNA synthetase.
Branched with a bootstrap value under 0.5 were deleted.

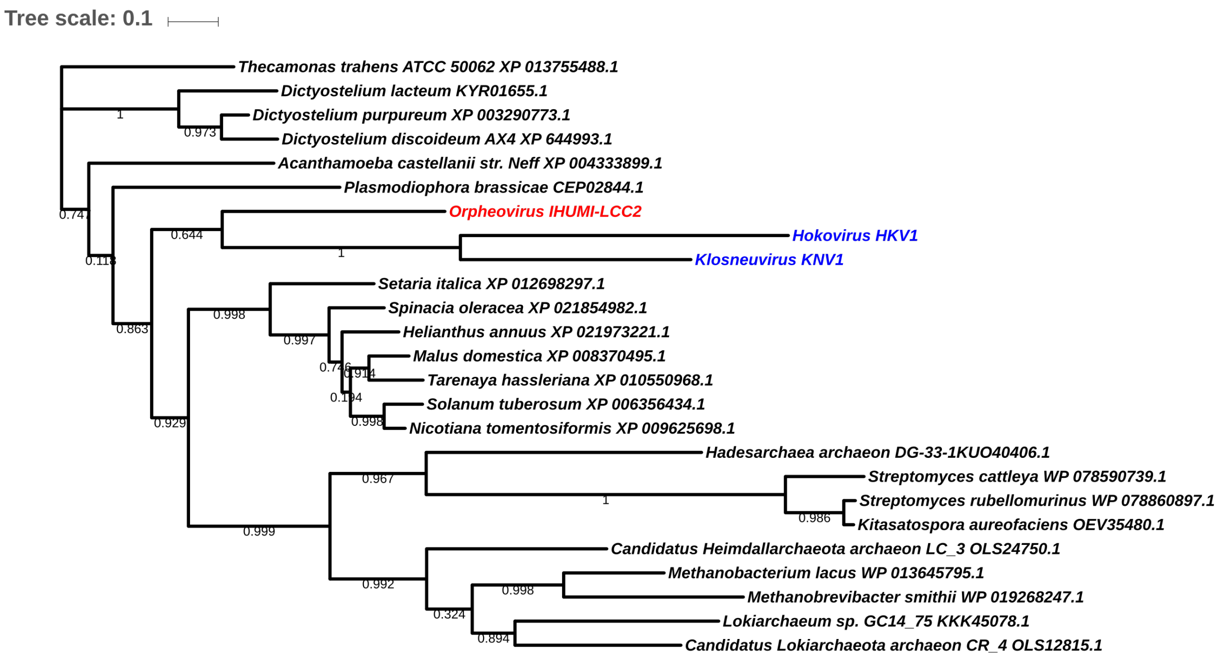

Supplementary figure S8: Phylogenetic tree based on Serine-tRNA synthetase.
Branched with a bootstrap value under 0.5 were deleted.

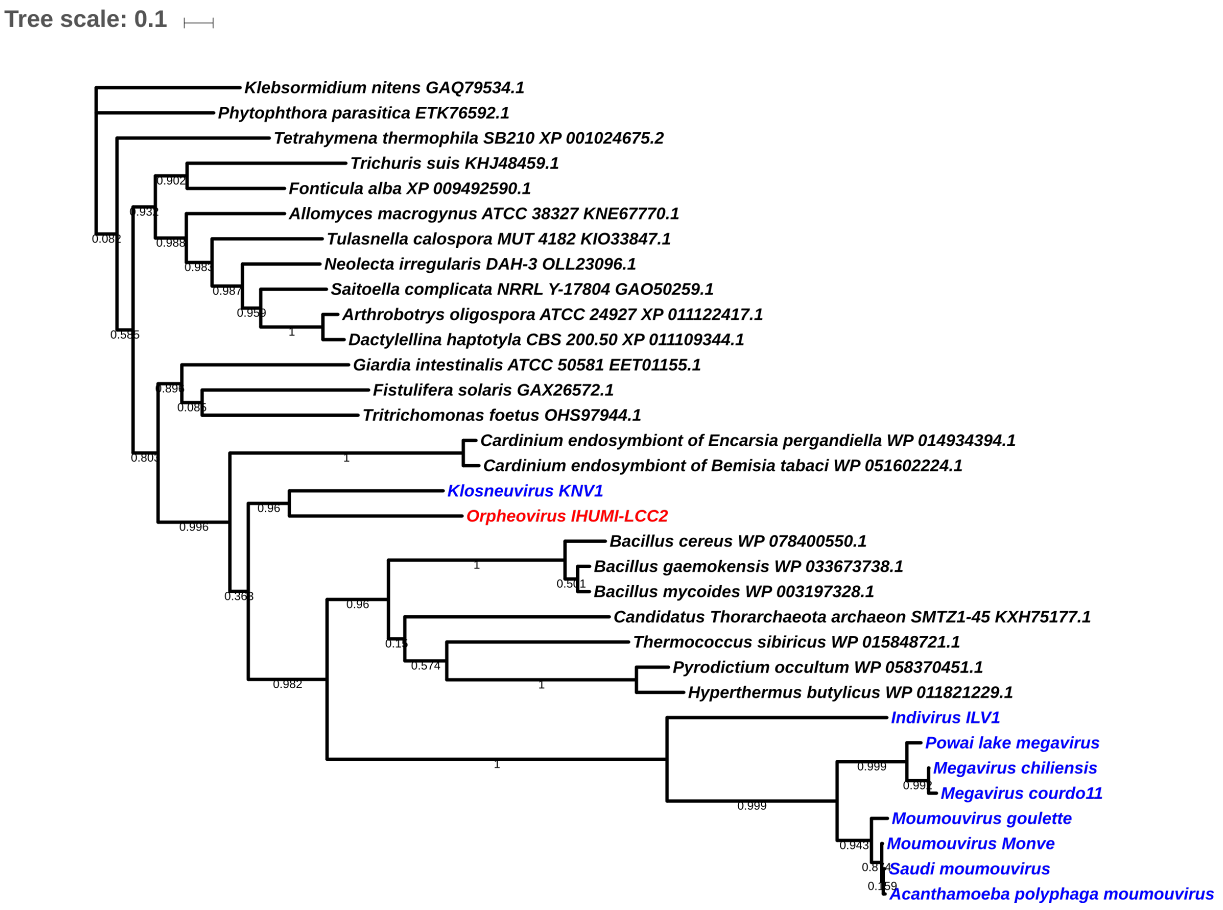

Supplementary figure S9: Phylogenetic tree based on Aspartyl-tRNA synthetase.
Branched with a bootstrap value under 0.5 were deleted.

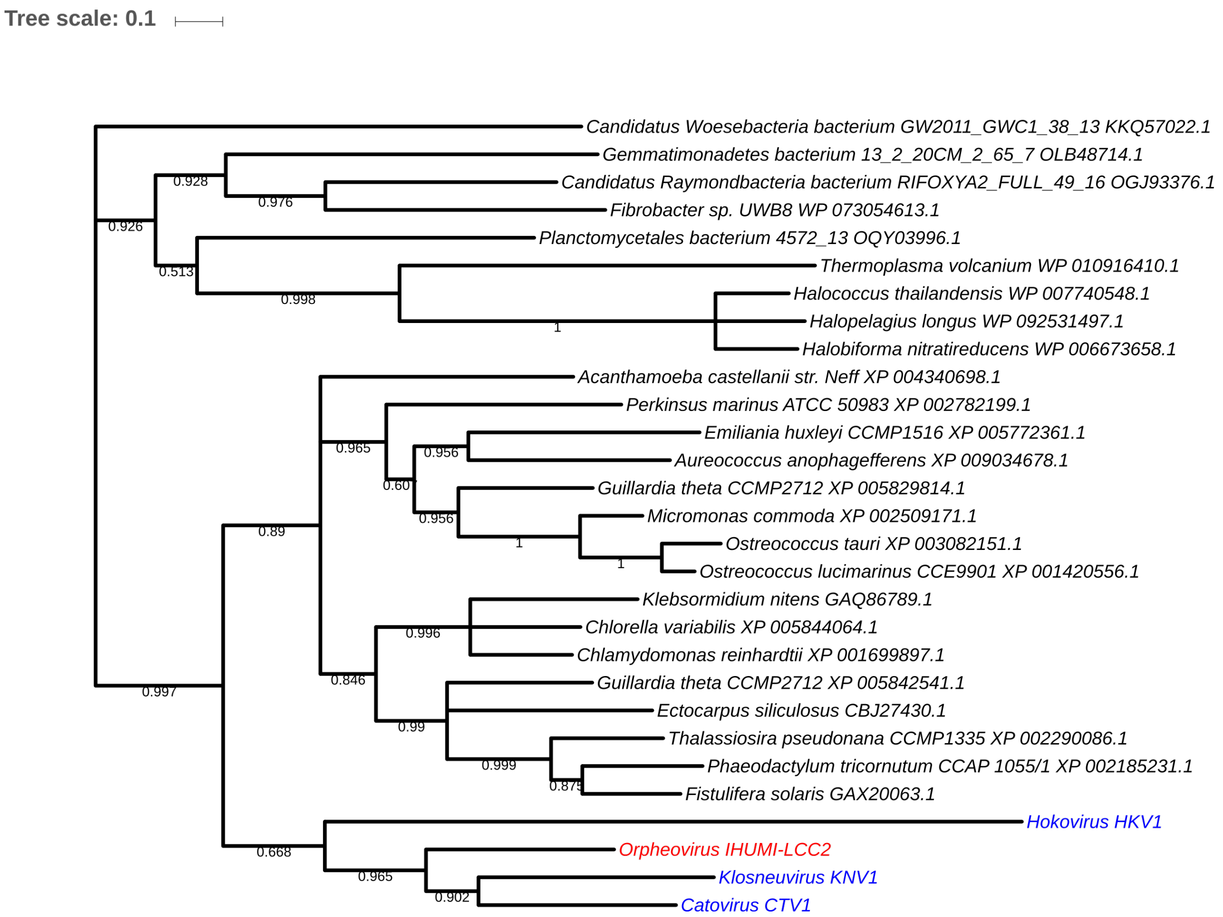

Supplementary figure S10: Phylogenetic tree based on Histidyl-tRNA synthetase.
Branched with a bootstrap value under 0.5 were deleted.

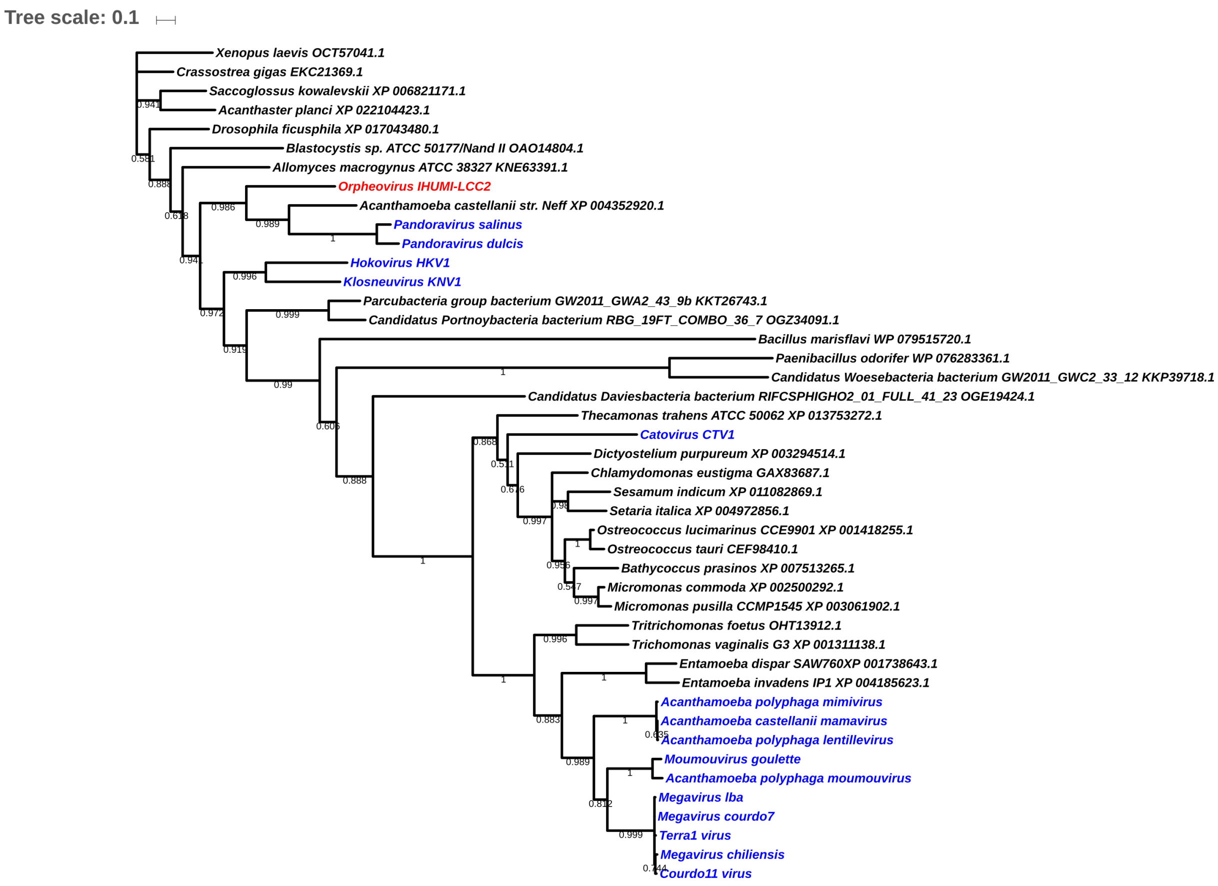

Supplementary figure S11: Phylogenetic tree based on Tyrosyl-tRNA synthetase.
Branched with a bootstrap value under 0.5 were deleted.

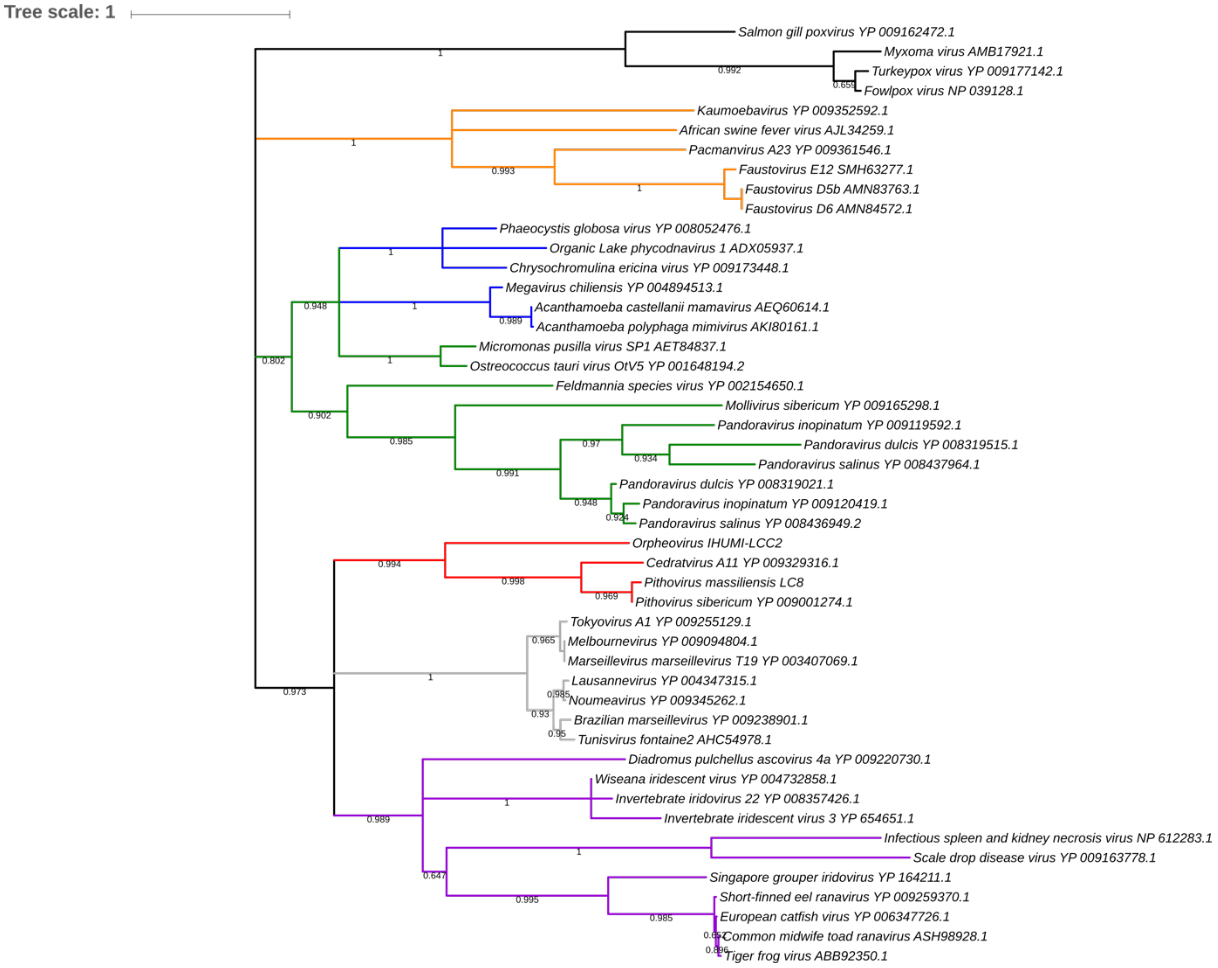

Supplementary figure S12: Phylogenetic tree based on 48 VLTF3 protein of nucleo-cytoplasmic large DNA viruses.

Branch values lower than a bootstrap value of 0.5 were deleted. Colors were assigned for different group of viruses: blue for Mimivirus and extended Mimiviridae; green for Pandoraviruses, *Mollivirus sibericum* and Phycodnaviridae; orange for groups of Asfarviridae, Faustoviruses, Pacmanvirus and Kaumoabevirus; grey for *Marseilleviridae*; red for Orpheovirus, Cedratvirus and Pithoviruses; black for *Poxviridae* members and purple for *Asco-Iridoviridae*.

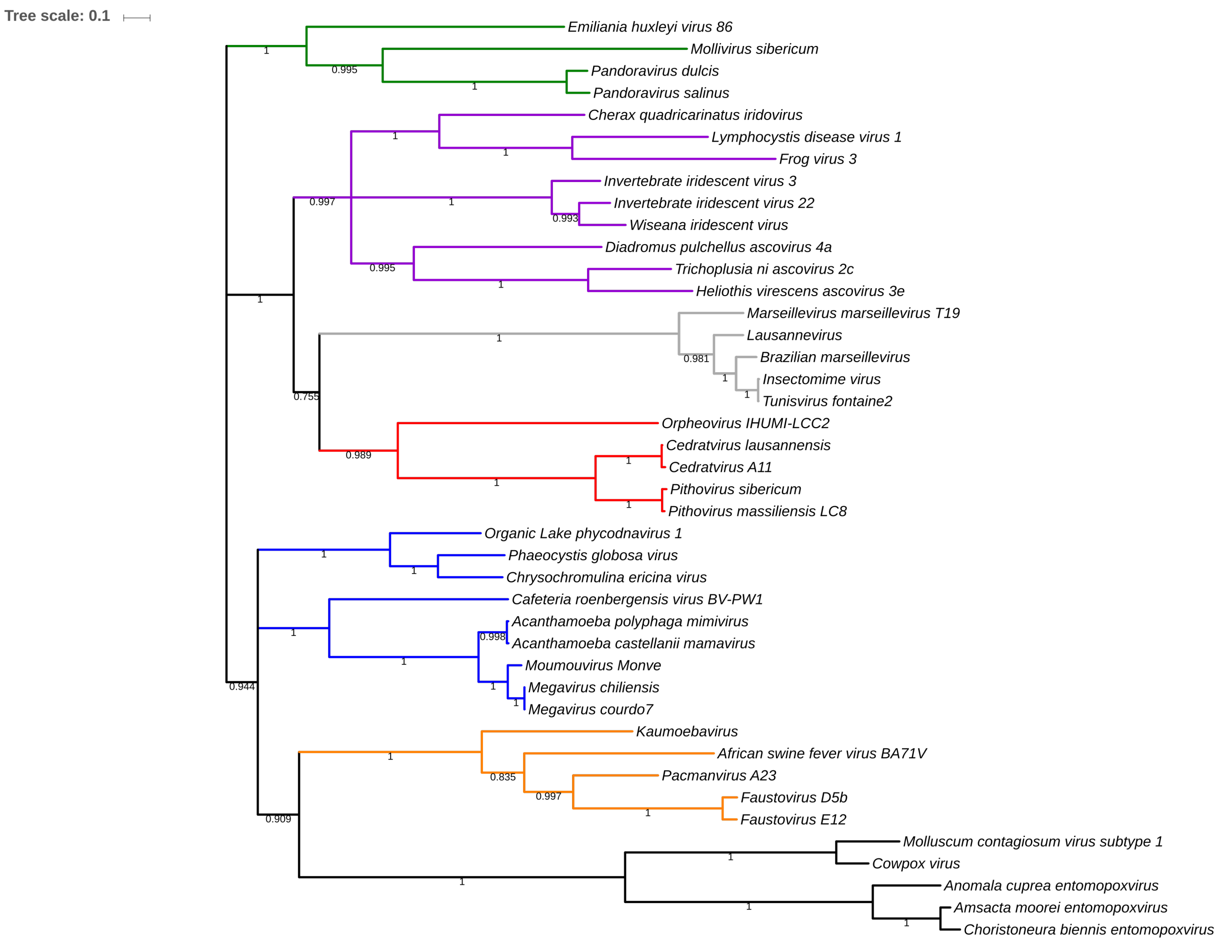

Supplementary figure S13: Phylogenetic tree based on 42 RNA polymerase subunit 1 Rpb1 protein of nucleo-cytoplasmic large DNA viruses.

Branch values lower than a bootstrap value of 0.5 were deleted. Colors were assigned for different group of viruses: blue for Mimivirus and extended Mimiviridae; green for Pandoraviruses, *Mollivirus sibericum* and Emilliania huxleyi virus; orange for groups of Asfarviridae, Faustoviruses, Pacmanvirus and Kaumoabevirus; grey for *Marseilleviridae*; red for Orpheovirus, Cedratvirus and Pithoviruses; black for *Poxviridae* members and purple for *Asco-Iridoviridae*.

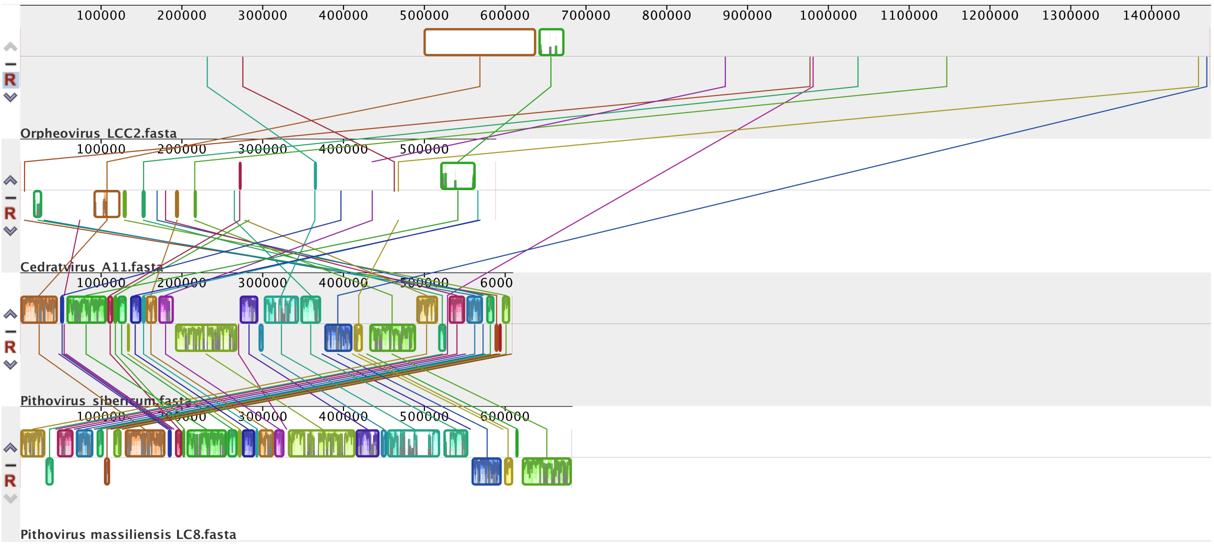

Supplementary figure S14: MAUVE genome comparison performed on 4 viruses.
From the top to the bottom: *Orpheovirus IHUMI-LCC2, Cedratvirus A11, Pithovirus massiliensis LC8, Pithovirus sibericum P1084-T.*
Parameters visualized are solid colinearity block, similarity-plot and -range and connecting lines.

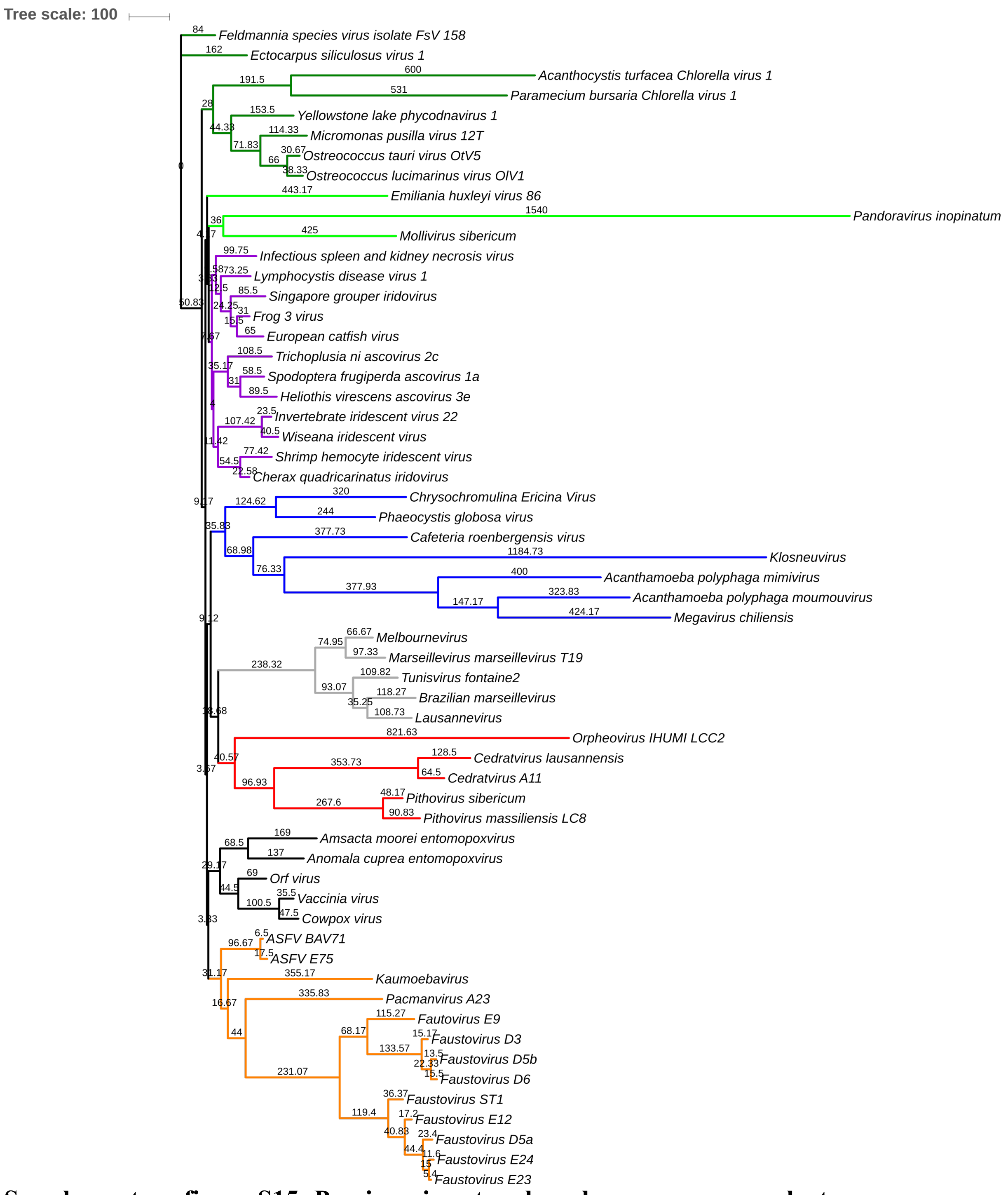

Supplementary figure S15: Parsimonious tree based on pan-genome clusters among NCLDV.

Branch values indicate each branch length. It's a parsimonious tree representation determined by clustering homologous predicted proteins using GET_HOMOLOGUES (OMCL algorithm) with following parameters: 60% coverage and 10^{-2} as e-value cut-off. Colors were assigned for different group of viruses: blue for Mimivirus and extended Mimiviridae; neon green for Pandoraviruses, *Mollivirus sibericum* and Emilliania huxleyi virus; green for *Phycodnaviridae;* orange for groups of Asfarviridae, Faustoviruses, Pacmanvirus and Kaumoabevirus; grey for *Marseilleviridae*; red for Orpheovirus, Cedratviruses and Pithoviruses; black for *Poxviridae* members and purple for *Asco-Iridoviridae*.

#	Template	Alignment Coverage	3D Model	Confidence	% i.d.	Template Information
1	c4amsA_	Alignment	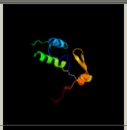	97.6	28	**PDB header:**transferase<br>**Chain:** A: **PDB Molecule:**mg662;<br>**PDBTitle:** a megaviridae orfan gene encode a new nucleotidyl transferase
2	c4amqA_	Alignment	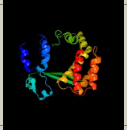	97.0	28	**PDB header:**transferase<br>**Chain:** A: **PDB Molecule:**l544;<br>**PDBTitle:** a megaviridae orfan gene encodes a new nucleotidyl transferase
3	d2doaa1	Alignment	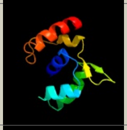	72.1	14	**Fold:**DNA/RNA-binding 3-helical bundle<br>**Superfamily:**"Winged helix" DNA-binding domain<br>**Family:**ELL N2 domain-like
4	c2e5nA_	Alignment	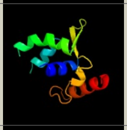	70.3	10	**PDB header:**transcription<br>**Chain:** A: **PDB Molecule:**rna polymerase ii elongation factor ell2;<br>**PDBTitle:** solution structure of the ell_n2 domain of target of rna2 polymerase ii elongation factor ell2
5	d2q66a2	Alignment	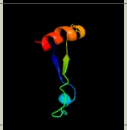	51.9	20	**Fold:**Nucleotidyltransferase<br>**Superfamily:**Nucleotidyltransferase<br>**Family:**Poly(A) polymerase, PAP, N-terminal domain
6	c2q0dA_	Alignment	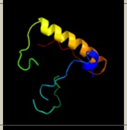	44.9	16	**PDB header:**transferase<br>**Chain:** A: **PDB Molecule:**rna uridylyl transferase;<br>**PDBTitle:** terminal uridylyl transferase 4 from trypanosoma brucei2 with bound atp
7	d2nn4a1	Alignment	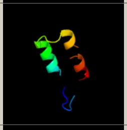	40.9	22	**Fold:**YqgQ-like<br>**Superfamily:**YqgQ-like<br>**Family:**YqgQ-like
8	c1bzkA_	Alignment	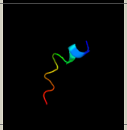	39.8	27	**PDB header:**transport protein<br>**Chain:** A: **PDB Molecule:**protein (band 3 anion transport protein);<br>**PDBTitle:** structural studies on the effects of the deletion in the2 red cell anion exchanger (band3, ae1) associated with3 south east asian ovalocytosis.
9	c1q78A_	Alignment	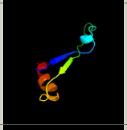	31.1	20	**PDB header:**transferase<br>**Chain:** A: **PDB Molecule:**poly(a) polymerase alpha;<br>**PDBTitle:** crystal structure of poly(a) polymerase in complex with 3'-2 datp and magnesium chloride
10	c2xr4A_	Alignment	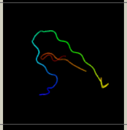	30.8	26	**PDB header:**sugar binding protein<br>**Chain:** A: **PDB Molecule:**lectin;<br>**PDBTitle:** c-terminal domain of bc2l-c lectin from burkholderia cenocepacia
11	d1q79a2	Alignment	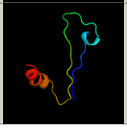	29.9	13	**Fold:**Nucleotidyltransferase<br>**Superfamily:**Nucleotidyltransferase<br>**Family:**Poly(A) polymerase, PAP, N-terminal domain

Supplementary figure S16: Results obtained using Phyre2.
