# Supplementary material for: Orpheovirus IHUMI-LCC2: A New Virus among the Giant Viruses
Source: Front Microbiol. 2018 Jan 22;8:2643. doi: 10.3389/fmicb.2017.02643 (PMC5786535; doi:10.3389/fmicb.2017.02643)
Supplement: Supplementary file 2 [file Data_Sheet_2.PDF]

```
Reference sequence (1): Acanthamoeba_castellanii_mamavirus
Identities normalised by aligned length.
Colored by: identity + property

                                                          1 [         .         .         .         :         .         . 80
 1 Acanthamoeba_castellanii_mamavirus           100.0%      YTPFFYVEIPKQ-----------WKQRQVDKFVEILKNKVQYHCKKNLDEDFDLSKSLIKYAMVKKHKFYN--------
 2 Acanthamoeba_polyphaga_mimivirus              98.6%      YTPFFYVEIPKQ-----------WKQRQVDKFVEILKNKVQYHCKKNLDEDFDLSKSLIKYAMVKKHKFYN--------
 3 Acanthamoeba_polyphaga_moumouvirus            56.0%      FTPYFYVEIPLN-----------WKRNHVAKFIEILKRRVSWSTNNNPNYNYDLSNSLIKYKIVQKYNFYN--------
 4 Amsacta_moorei_entomopoxvirus                 10.1%      FDIYEYFLCDYE---------LSESNTKLKNIKNNIDK-----------YKNS-FNSSYLVLEDYKIIT-------
 5 Anomala_cuprea_entomopoxvirus                  9.7%      FDLYNYFLCSED-----------DVDNNEYLKLLKN-----------NIIKFNNDNIAKIITLNEFKI-------
 6 ASFV_BAV71                                     9.7%      IPVFFDVQIE-------------FNDTNQLFLKSLLT-----------AENI-AYERLETLTQRPVMG-------YR
 7 ASFV_E75                                       9.7%      IPVFFDVQIE-------------FNDTNQLFLKSLLT-----------AENI-AYERLETLTQRPVMG-------YR
 8 Brazilian_marseillevirus                      12.6%      FHPYVYIQLPKK------RN-LKWGKREARILFEHLKT------------KVCKKY-PPVSFELKKKSFLKG--------
 9 Cafeteria_roenbergensis_virus_BV-PW1          20.9%      FHPRFYIKIPEN-----------WTNRHLKRMETFLKS------KDNRKIWNKDRECLVSCSMVKRKKAYG--------
10 Cannes_8_virus                                13.3%      FCPYTYIQLPKK------KK-ISWGKREERLLFEYLKE-----------RVCVRSGPPVSFTREKKFFLKG--------
11 Catovirus_CTV1                                24.3%      FTPYFYIQIDDK-----------WKKDTVDKLVQILKD---SVTIKGDNGWAKCENSFKGYIIEEKYKFRG--------
12 Cedratvirus_A11                               12.1%      YNYVCYLELRPL---MVGKRKMEWKQGIADYLIKTINK-----------SLGEFAPIGTEFLFREKFYY--------
13 Choristoneura_biennis_entomopoxvirus          10.5%      FDIYEYFLCDLQ-----------LSETNEILKYIKN-----------NIDKYTNS-FNSSYIILKDFNIIT-------
14 Common_midwife_toad_ranavirus                 13.2%      FRPGFYVETASC-----------QAVTRALQDF-----------------RDVRAEKRVRDHLYA--------
15 Cowpox_virus                                  10.5%      FPHYFYYVVTDE-----------------------------------IYQSL-APPPFNARPMGKMRT--------
16 Diadromus_pulchellus_ascovirus_4a             12.7%      CKTRLIVEFTDF---------DYLVNNFKNIKHQLEE----------FIFNRR-DKTTVKLVYKSKLYG-----AYRT
17 Ectromelia_virus                              10.5%      FPHYFYYVVSDE-----------------------------------IYQAL-APPPFNARPMGKMRT--------
18 Emiliania_huxleyi_virus_145                   14.6%      FRPVLYYHTNDS---------VSTLKNKIDESLK-------------------LK-GDIDAKVIKRKRTYG--FHPDKDD
19 Emiliania_huxleyi_virus_86                    14.6%      FRPVLYYHTNDS---------VSTLKNKIDESLK-------------------LK-GDIDAKVIKRKRTYG--FHPDKDD
20 European_catfish_virus                        13.1%      FRPGFYVETASC-----------QAVTRALQDF-----------------RDVRVEKRVRDHLYA--------
21 Faustovirus_D3                                 8.8%      VPIYVDIMVPKR-----------FKGQTGEFYTAVMS-----------SLTHGPQRP-EVAHWDVVQGYRLHG--------
22 Faustovirus_E12                                8.9%      VPIYIDIMVPKR-----------FRAQTNEFYTSVMS-----------SLTAGQNRP-DVSHWDIVQGYKLHG--------
23 Faustovirus_E24                                8.9%      VPIYIDIMVPKR-----------FRAQTNEFYTSVMS-----------SLTAGQNRP-DVSHWDIVQGYKLHG--------
24 Faustovirus_E9                                 9.0%      VPIYVDIMVPRR-----------FRNQTAEFYTSVMS-----------SLTHGPQRP-DVAHWDIVHGYRLHG--------
25 Faustovirus_Liban                              9.0%      VPIYVDIMVPKR-----------FRAQTNEFYTSVMS-----------SLTTGQNRP-DVSHWDIVHGYKLHG--------
26 Feldmannia_species_virus                      13.4%      FRPWFYVDLNEFFT-------FAQFKSVVAEQYW-----------------AR-NVVSCELVARKRFIG--------
27 Frog_virus_3                                  13.2%      FRPGFYVETASC-----------QAVTRALQDF-----------------RDVSAEKRVRDHLYA--------
28 Heliothis_virescens_ascovirus_3e              11.3%      VKTKLYVEFPENYDLNP-RR-WSAIRTILKDAIYCKND---------KCDSN-----IQRVSLQPLYG--------
29 Hokovirus_HKV1                                20.3%      FKPYFYVMVPDS---------WYLNKSKPQTFIKHLLE-------NEITRDSDKK-SLYDCEIVRHHAFTE--------
30 Infectious_spleen_and_kidney_necrosis_virus   12.7%      FKQVVYVECTDP-------------QQHDPRSTFT----------------QH-GFRVYETPRACSLYG--------
31 Insectomime_virus                             12.3%      FRPYLYIQLPNK------KN-LKWGKREARILFEHLKT------------KVCKRS-PPVSFELKKKYFLKG--------
32 Invertebrate_iridescent_virus_22              13.4%      FQPFVWVELPTH---------INWKLGENKERLYLY----------------FNQMFPNVKCTLKFCKKLYG--ANLKKKG
33 Invertebrate_iridescent_virus_3               14.2%      FRQFVWVELPSF---------IDWSLSHNVDRVV-----------------RYFEQTWPNVGVQFKMYRKLYGANLKQSRSN
34 Kaumoebavirus                                 10.2%      IQPYFDIEVPDD------QT-VDWNAGGELRSTDVSKR---AAVLRNDIEDALGR-TLVKSRIIMAKPLQG--------
35 Klosneuvirus_KNV1                             15.8%      YTPYFYVKIPKD-----------WKKQKVQVLLDYVKK-----EVEKNPKNADILNSLKEWDLIDRCVFKE--------
36 Kurlavirus_BKC-1                              12.6%      FHPYLYIQLPKK------RN-LSWGKREARVLFEHLTT------------KVCKKF-PPVSYELKKKYFLKG--------
37 Lausannevirus                                 12.4%      FRPYLYIQLPKK------RN-LSWGKREARTLFEHLTT------------RVCKKF-PPVSYELKKKYFLKG--------
38 Lumpy_skin_disease_virus_NI-2490              10.5%      FNFYFYYAVTED-----------------------------------VYISL-SPPAYSSDFLGNMNL--------
39 Lymphocystis_disease_virus                    13.5%      FKPYAYVDYSIN-----------VKSMIKS-----------------DDVFIEYVEKSHLYS--------
40 Marseillevirus_marseillevirus_T19             13.3%      FCPYTYMQLPKK------KK-ISWGKREERLLFEYLKG-----------RVCVRSGPPVSFTREKKFFLKG--------
41 Megavirus_chiliensis                          60.1%      FTPYFYVQIPTN-----------WKKHHVDKFVYYLKRKVSWTTNNNPNYACDLSNSLVNYKMVEKHNFYN--------
42 Megavirus_courdo7                             60.4%      FTPYFYVQIPIS-----------WKKHHVDKFVYYLKRKVSWTTNNNPNYACDLSNSLVNYKMVEKHNFYN--------
43 Megavirus_ursino                              60.1%      FTPYFYVQIPTN-----------WKKHHVDKFIYYLKRKVSWTTNNNPNYAYDLSNSLVNYKMVEKHNFYN--------
44 Melbournevirus                                13.3%      FCPYTYMQLPKK------KK-ISWGKREERLLFEYLKE-----------RVCVRSGPPVSFTREKKFFLKG--------
45 Micromonas_pusilla_virus_SP1                  16.3%      FTPYFFIKLDSR-----------VSAKELYKSIDE------------KCPECVISYSVMDSKDVWG--------
46 Mollivirus_sibericum                           9.9%      PVFKCYVQCPDA---------WTLDEDMEDMVRW-VER-------------QQKLPP-GSISCEATKSQQYCGF-VPESDTQ
47 Molluscum_contagiosum_virus_subtype_1          9.1%      FPHYYYYVVDGG-----------------------------------TLSTL-APPPRAASALGEFEL--------
48 Monkeypox_virus                               10.5%      FPHYFYYVVTDE-----------------------------------IYQSL-SPPPFNARPMGKMRT--------
49 Myxoma_virus                                  10.7%      FHYYFYYVVTED-----------------------------------VYKTL-VPIPTASIFLDSMSI--------
50 Noumeavirus                                   12.5%      FHPYLYIQLPKK------RN-LSWGKREARVLFEHLTT------------KVCKKF-PPVSFEFKKKYFLKG--------
51 Orf_virus                                     10.2%      FVQHFYYVVRAD-----------------------------------AVADIAQPLAWT-RALGPMSV--------
52 Organic_Lake_phycodnavirus_1                  18.2%      FTPFVYIKVSND-----------WNKHKTDEFIEHLKQ-------HPNKSIAYGSKDIVSYEWVKKKSLYG--------
53 Orpheovirus_LCC2                              12.8%      APVYCYIEMPQVVNQRK----KTWLEHDVDEFMGVLKS-----RLRGYSKDSEDH-SPIQYQLEHKKKVYY--------
54 Ostreococcus_lucimarinus_virus_1              16.7%      FTPYFFIKLPPN-----------IDTAKIQRIYNILDE-----------QCKDSLVGYSVTKSKDVWG--------
55 Ostreococcus_tauri_virus_OtV5                 16.7%      FTPYFFIKLPEK-----------IDAGKIRRIYNILDE-----------KCKDSLVAYSVMKSKDVWG--------
56 Pacmanvirus_A23                                9.1%      IEVHVDIMVP-------------YGMTAAQYDDILRGQLT-----------SKQL-PFTGIADIKLHRLHG--------
57 Pandoravirus_dulcis                            9.2%      MRYAIYVLCPAT-----------WGVEHMQALAATVEG-----------YYGIRR-GGVTYQQRALHHMLG--WEPETND
58 Pandoravirus_inopinatum                        8.8%      MRYVIYVLCPAT-----------WGVAHMQALAATIEG-----------YYGIRR-GGVTYQQKPLHHMLG--WEPETND
59 Pandoravirus_salinus                          10.5%      MRYAIYVLCPPS-----------WGVAHMQALAATIEG-----------YYGIRR-GGVTYQQRPLHHMLG--WEPETND
60 Paramecium_bursaria_Chlorella_virus_CVK2      15.4%      FTPYFLLEVPES-----------WSPARTNLFITETAM-----------KYDAI--RPMCLSTKRKNMWG--------
61 Paramecium_bursaria_chlorella_virus_MT325     15.1%      FTPVFLLELPAA-----------WSPSRQKLFITETAI-----------KYGAIKDMCLPVKKKSMWG--------
62 Paramecium_bursaria_Chlorella_virus_NYs1      14.2%      FTPFFLLETPES-----------WSAARTNLFITETAM-----------KYDAI--RPSCLPTKRKNMWG--------
63 Phaeocystis_globosa_virus                     16.9%      FNPFYYIKVGDD-----------WGESDRAEFIAHIKN-----------KLGAYYED-SIVASKLVKRHKLYG--------
64 Phaeocystis_globosa_virus_14T                 16.8%      FNPFYYIKVGDD-----------WGESDRAEFIAHIKN-----------KLGAYYED-SIVASKLVKRHKLYG--------
65 Pithovirus_massiliensis                       11.6%      FLHVCYFELKPI-LVSGRS--VPWDENRASHLLMKIT------------TLLGDN-KPINARFVRKPKLYY--------
66 Pithovirus_sibericum                          11.8%      FLHVCYFELKPI---LVSGRSVPWDENRASHLLMKITT-----------LLGDNKPINARFVRKPKLYY--------
67 Port-miou_virus                               12.4%      FRPYLYIQLPKK------RN-LSWGKREARTLFEHLTT------------RVCKKF-PPVSYELKKKYFLKG--------
68 Powai_lake_megavirus                          60.0%      FTPYFYVQIPTN-----------WKKHHVDKFIYYLKRKVSWTTNNNPNYAYDLSNSLVNYKMVEKHNFYN--------
69 Scale_drop_disease_virus                      12.8%      YKPSIYIEVFDI-------------TEMKEIKQLVIK-----------------L-GGIVTDTPNACNIYS--------
70 Short-finned_eel_ranavirus                    13.1%      FRPGFYVETASC-----------QAVTRALQDF-----------------RDVRAEKRVRDHLYA--------
71 Singapore_grouper_iridovirus                  13.3%      FRPGFYVESIDS-----------AKMLSVLRDL-----------------KSIRTKRRILNHLYS--------
72 Skunkpox_virus                                11.0%      FPHYFYYVVTDE-----------------------------------TYQSL-SPPPLNARNMGQMRT--------
73 Spodoptera_frugiperda_ascovirus_1a            10.9%      VKTKLYCEFPDDYPLERGGAVWRSVRAKLKEAIT-----------CKHDQ-DESNVQLVDMQPLYG--------
74 Testudo_hermanni_ranavirus                    13.2%      FRPGFYVETASC-----------QAVTRALQDF-----------------RDVRAEKRVRDHLYA--------
75 Tiger_frog_virus                              13.1%      FRPGFYVETASC-----------QAVTRALQDV-----------------RDVRVEKRVRDHLYA--------
76 Tokyovirus_A1                                 12.9%      FCPYIYMQLPKK------KG-ISWGKREQRLLFEYLQE-----------RVCAKSGAPVSFERKKKSFLKG--------
77 Trichoplusia_ni_ascovirus_2c                  10.4%      VTTSILLEFPDN---------YDVETNWSRVRMELMSA-----------IFCSTDKTDKNVVMVKKQPLYG--------
78 Tunisvirus_fontaine2                          12.2%      SARICTFNSPTK------KN-LKWGKREARILFEHLKT------------KVCKRS-PPVSFEFKKKYFLKG--------
79 Vaccinia_virus                                10.6%      FPHYFYYVVTDE-----------------------------------IYQSL-SPPPFNARPLGKMRT--------
80 Variola_virus                                 10.3%      FPHYFYYVVTDE-----------------------------------IYQSL-TPPPFNARPMGKMRT--------
81 Volepox_virus                                 10.8%      FPHYFYYVVTED-----------------------------------TYQSL-APPPLNARHLGEMKT--------
82 Wiseana_iridescent_virus                      14.4%      FRPFVWVELPTH---------INWKDNSNKERLCL-------------YFRQLFPEIKCTLKFCKKLYG--ANLKKKG
83 Yaba_monkey_tumor_virus                       10.8%      FPYYFYYALTDE-----------------------------------KYTSL-TPPAFKSVFIGKMTN--------
84 Yellowstone_lake_phycodnavirus_1              17.0%      FRPYFYVKLR-------------VQHNFADFAALIRK-----------KF-QPTEIKEVRAKDLWG--------
   consensus/100%                                           ......h.....................................................................h.........
   consensus/90%                                            h..hh.h.h...           ........ ..........   ...........      ..........ht...h..hh.      ......
   consensus/80%                                            h..hhah.hstp        ..  .....t..t.........   ...............thphh.h..hhs
   consensus/70%                                            Fp.ahYlplspp             ....ttt.p.hh.....             ....t..s.sthphhtttphhs

                                                         81          .         1         .         .         :         .        . 16
 1 Acanthamoeba_castellanii_mamavirus           100.0%      -FRNKQLYNFLLLVFKSHTAMK-EFSSILAR-----------------PLEAKGLTNKPM----LYQRYESNI----
 2 Acanthamoeba_polyphaga_mimivirus              98.6%      -FRNKQLYNFLLLVFKSHTAMK-EFSSILAR-----------------PLEAKGLTNKPM----LYQRYESNI----
 3 Acanthamoeba_polyphaga_moumouvirus            56.0%      -FTNKKCFNFVMLFFKSHTAMK-EFSNALAR-----------------PLKTEGLTKDPM----LYQRFESNI----
 4 Amsacta_moorei_entomopoxvirus                 10.1%      --NEVNIQYYYNYTEDSKITLN-NNDLV---------------------LFMTPYKIEKIY----SKY--NIFI----
 5 Anomala_cuprea_entomopoxvirus                  9.7%      --ISNEVQIQKYYNFRHNLKDK-EIYKNLY-------------------LFLTRFKISRVH----SDY--NHNF----
 6 ASFV_BAV71                                     9.7%      E-KEKEFAPYIRIFFKSLYEQR-KAITY-----------------------LNNMGY----NTA--ADDT----
 7 ASFV_E75                                       9.7%      E-KEKEFAPYIRIFFKSLYERR-KAITY-----------------------LNNMGY----NTA--ADDT----
 8 Brazilian_marseillevirus                      12.6%      ----RVPVQTLRLEFLNIAGAK-MCANAMEN--------RENR---------VEGLGVFAAKEL----RSH--EQNI----
```

```
 9 Cafeteria_roenbergensis_virus_BV-PW1         20.9%   -FTNNKLYKFAVLTFTNYYACK-KFASLFEL--------NKV---------YIPGVTTKAITF----KIY--EANL----
10 Cannes_8_virus                               13.3%   ----KIPVDTLKLEFRTLSAMR-ACAKAMET--------RQHR--------IDGVGIFEAKEL----RTH--EQNI----
11 Catovirus_CTV1                               24.3%   -FTNFKKFKFIKLIFRDLDSMR-GWAGYLNK--------KK----------FYPGISSKAMKF----PLF--ESNI----
12 Cedratvirus_A11                              12.1%   -YQGKKKYPMVKFVFNNPVSLQ-KLTAMTRR---------------PIKFEVKRDGRCVKLEANS----TLW--EDKQ--TQ
13 Choristoneura_biennis_entomopoxvirus         10.5%   --NEVELQSYYNFTEDSKIKLN-NTDLI-----------------------LFMTPYKIERIY----SKY--NRNF----
14 Common_midwife_toad_ranavirus                13.2%   --TRGSSVPFVWVTFRCWFDAR-KASDV-------------------------------LVKAGF----ACH--QCRA----
15 Cowpox_virus                                 10.5%   ----IDIDETISYNLDIKDRKC-SVADM-----------------------WLIEEPKKRNIQ----NAT--MDEF----
16 Diadromus_pulchellus_ascovirus_4a            12.7%   DSGDVKKFPYVEVVFSSRIGML-SFKKKV----------------------TEHAPSMFKEEV----KFH--QCSV----
17 Ectromelia_virus                             10.5%   ----IDIDETISYNLDIKDRKC-SVADM-----------------------WLIEEPKKRSIQ----NAT--MDEF----
18 Emiliania_huxleyi_virus_145                  14.6%   S-TSHEQIRIVEVSFPSVSKMK-AACYR-----------------------SGDKEPPETRLP----KPW--EQGV----
19 Emiliania_huxleyi_virus_86                   14.6%   S-TSHEQIRIVEVSFPSVSKMK-AACYR-----------------------SGDKEPPETRLP----KPW--EQGV----
20 European_catfish_virus                       13.1%   --TRGSSVPFVWVTFRCWFDAR-KASDV-------------------------------LVKAGF----ACH--QCRA----
21 Faustovirus_D3                                8.8%   --FQQQTRPWIRFLFANMGQRS-AFLDLVAQ--------H-----------NEKYKANKKAQY----CTA--NDDSGRPE
22 Faustovirus_E12                               8.9%   --FQRATRPWLRFYFTNLGLRS-AFLDLVGQ--------H-----------NVKYKANKKQQY----QTA--NDDSGRPE
23 Faustovirus_E24                               8.9%   --FQRATRPWLRFYFTNLGLRS-AFLDLVGQ--------H-----------NVKYKANKKQQY----QTA--NDDSGRPE
24 Faustovirus_E9                                9.0%   --FQQQTRPWLRFHFANMGLRS-AFIDLVAQ--------H-----------NEKYKTNKKAQY----CTA--NDDSGRPE
25 Faustovirus_Liban                             9.0%   --FQRATRPWLRFYFTNLGLRS-AFLDLVSQ--------H-----------NVKYKANKKQQY----QTA--NDDSGRPE
26 Feldmannia_species_virus                     13.4%   F-ADGKSFDYVLMTFTGLIPMY-CSRKY-------------------------------LRTLKV----HLH--EDSV----
27 Frog_virus_3                                 13.2%   --TRGSSVPFVWVTFRCWFDAR-KASDV-------------------------------LVKAGF----ACH--QCRA----
28 Heliothis_virescens_ascovirus_3e             11.3%   --AKQVRTYVAIEFTSEVGKR-AFINKISG--------KCDQRTKRTS---AKFPDNVTGDQL----RFH--WMNV----
29 Hokovirus_HKV1                               20.3%   F-TNYKLYTFMKLYFNDYISYR-VYERLFYD--------YDDKGMHFYKKYTNRVCDLENYEF----IVY--EADM----
30 Infectious_spleen_and_kidney_necrosis_virus  12.7%   ---AKGVGTYFAARVPNYNAMR-DVQET--------------------------------QGPF----KIH--ESRV----
31 Insectomime_virus                            12.3%   ----KIPVQTLRLEFMSIAGAK-MCSNAMEN--------RENR--------VENLGVFAPKEL----RAH--EQNI----
32 Invertebrate_iridescent_virus_22             13.4%   DKYTFKLFPFLMCSSPSFKELK-FNLPSKLK--------T-----------PQRIMGLGNLKF----HVH--GQDA----
33 Invertebrate_iridescent_virus_3              14.2%   GEYVHKKFPFLQCSSMSWKMLK-FTLPTALKT-------------------PQRIMGLGSIKF----RVH--GQDA----
34 Kaumoebavirus                                10.2%   --FTKEPKPYIRLYFNNLKDRK-AGLNW-------------------------------AEQEGY----AIF--SNDP----
35 Klosneuvirus_KNV1                            15.8%   -FTNYKLFQFVRLVFHSYEGFR-AYERIFNR--------RL----------LIHSLGMKPIKY----ELY--ESNI----
36 Kurlavirus_BKC-1                             12.6%   ----KVPVSTLRLEFLNIAGAK-MCSNAMEN--------RENR--------VEGLGTFAAKEL----RAH--EQNI----
37 Lausannevirus                                12.4%   ----KVPVSTLRLEFLNIAGAK-MCSNAMEN--------RENR--------IEGLGTFAAKEL----RAH--EQNI----
38 Lumpy_skin_disease_virus_NI-2490             10.5%   ----IDISERVSNHVTDVEKKK-TNVNV-----------------------WLIKEPQKRNIP----KAI--MEEF----
39 Lymphocystis_disease_virus                   13.5%   ---VCTDKRFWKLTFKDGFAKK-RTIAL-------------------------------LTACNV----VIH--EDKA----
40 Marseillevirus_marseillevirus_T19            13.3%   ----KIPVDTLKLEFRTLSAMR-ACAKAMET--------RQHR--------IDGVGIFEAKEL----RTH--EQNI----
41 Megavirus_chiliensis                         60.1%   -FTKKKYFNFVMLVFKSQIAMR-EFSNVLAR--------------------PLKTEGLTKEPM----LYQRFESNI----
42 Megavirus_courdo7                            60.4%   -FTKKKYFNFVMLVFKSQIAMR-EFSNVLAR--------------------PLKTEGLTKEPM----LYQRFESNI----
43 Megavirus_ursino                             60.1%   -FTKKKFFNFVMLVFKSQIAMR-EFSNVLAR--------------------PLKTEGLTKEPM----LYQRFESNI----
44 Melbournevirus                               13.3%   ----KIPVDTLKLEFRTLSAMR-ACAKAMET--------RQHR--------IDSVGIIGAKEL----RTH--EQNI----
45 Micromonas_pusilla_virus_SP1                 16.3%   -FQNNQMFRFMKIDFVNLQTRR-RVDYFLRR--------------------PLHLYSSGLFKA----KVY--ESNL----
46 Mollivirus_sibericum                          9.9%   L-DRAKTWNYCAMGFPSLCHMQ-NAKHLLTQ--------TRN----P----RRAYPSPAEGL-----KVH--EADTFS--
47 Molluscum_contagiosum_virus_subtype_1         9.1%   ----ACIDETLARADALHAPTR-PRSQL-----------------------WLLEEAQPRQIA----GAL--LAEF----
48 Monkeypox_virus                              10.5%   ----IDIDEIISYNLDIKDRKC-SVADM-----------------------WLIEEPKKRSIQ----NAT--MDEF----
49 Myxoma_virus                                 10.7%   ----VDISEYTNNYVSDVTRKK-TMANI-----------------------WLIKENKKRNIP----KVV--MDEF----
50 Noumeavirus                                  12.5%   ----KVPVSTLRLEFLNIAGAK-MCSNAMEN--------RENR--------VEGLGTFAAKEL----RAH--EQNI----
51 Orf_virus                                    10.2%   ----VSIDEIVARSAKIPERQR-SEIEL-----------------------CLVASERKLAPP----EVF--MSDF----
52 Organic_Lake_phycodnavirus_1                 18.2%   -FDANKYYNFIYISCKNMSFVY-KLRSLYYE--------KDTQQ-------LNKGYLYKNAYT----KIY--ECMI----
53 Orpheovirus_LCC2                             12.8%   Y-QGNKKYPMVKTVFKSLEAMR-HCENILRK--------------------GLDMKDGRFIHC----SVW--ESNI----
54 Ostreococcus_lucimarinus_virus_1             16.7%   -FQNNEEFPFMKINFKNLQARR-LTDSFLRR--------PLDRT-------PELFNIFGVRNV----KVY--ESNL----
55 Ostreococcus_tauri_virus_OtV5                16.7%   -FQNNEEFVFMKVNFKHLQARR-LVDSFLRK--------PLDRT-------PELFNIFGVRNV----KVY--ESNL----
56 Pacmanvirus_A23                               9.1%   --FQKEKRSYKRVYFNNLQDRK-KIIEYIRS--------L-----------NKTLKSAGKDKI----ETA--SDDVGRD-
57 Pandoravirus_dulcis                           9.2%   L-TRTKRHNYCVLGFGSARVME-WAANAIHG--------GGNGKLCSRV--RLPLTERDMRSL----QVY--ERRV----
58 Pandoravirus_inopinatum                       8.8%   L-TRTKRHNYCVLGFGSARVME-WAANAIDG--------GDKLCRRLR---EPLLNERDMRSL----QVY--ERRV----
59 Pandoravirus_salinus                         10.5%   L-TRTRRHNYCVLGFGSARVME-WAANAIDG--------REAKLCRRL---REPLNERDMRSL----QVY--ERRV----
60 Paramecium_bursaria_Chlorella_virus_CVK2     15.4%   -FDGGKMRRNMVQFVFKTQAQLR-KAKYR------------------------------LKDQY----QIY--ESSV----
61 Paramecium_bursaria_chlorella_virus_MT325    15.1%   -FDGGVMRRNLAQFAFPTLEKMR-KAKYG------------------------------LKRDY----QIY--ESNV----
62 Paramecium_bursaria_Chlorella_virus_NYs1     14.2%   -YDGGKMRPMVQFVFKTLSQMR-KAKYR-------------------------------LKNEY----QIY--ESSV----
63 Phaeocystis_globosa_virus                    16.9%   F-DDNKLHTFIKISFTNTGAYN-RAKKMFYV--------DSTVDGVFKR--ELIPDGYLYEET----KCYLYEANI----
64 Phaeocystis_globosa_virus_14T                16.8%   F-DDNKLHTFIKISFTNTGAYN-RAKKMFYV--------DSTVDGVFKR--ELIPDGYLYEET----KCYLYEANI----
65 Pithovirus_massiliensis                      11.6%   -FQGKRTFPMIQMVFSSEFSLA-AFRKIASR--------PFHF--------TGANGKKISVSG----QLW--EDRQ----
66 Pithovirus_sibericum                         11.8%   -FQGKRTFPMIQMVFSSEFSLA-AFRKIASR--------------------PFHFTGANGKKISVSGQLW--EDRQ----
67 Port-miou_virus                              12.4%   ----KVPVSTLRLEFLNIAGAK-MCSNAMEN--------RENR--------IEGLGTFAAKEL----RAH--EQNI----
68 Powai_lake_megavirus                         60.0%   -FTKKKFFNFVMLVFKSQIAMR-EFSNVLAR--------------------PLKTEGLTKEPM----LYQRFESNI----
69 Scale_drop_disease_virus                     12.8%   ---CANKGCFGVTYSSFTVLK-TLQSE--------------------------------ATGRF----KIH--ESNI----
70 Short-finned_eel_ranavirus                   13.1%   --TRGSSVPFVWVTFRCWFDAR-KASDV-------------------------------LVKAGF----ACH--QCRA----
71 Singapore_grouper_iridovirus                 13.3%   --SRGSNVLFTWLDFECWFEAK-KASDL-------------------------------LTKAQF----KCH--QTRA----
72 Skunkpox_virus                               11.0%   ----IDIDEVISYNLDIKDRKC-SVSDM-----------------------WLIEEPKKRNIQ----NTT--MDEF----
73 Spodoptera_frugiperda_ascovirus_1a           10.9%   --VGKATKTFVAISFTSEVGKR-AFVNRVCG--------RHRKPPSAA---LKFPNNLSGERI----RFH--WINV----
74 Testudo_hermanni_ranavirus                   13.2%   --TRGSSVPFVWVTFRCWFDAR-KASDV-------------------------------LVKAGF----ACH--QCRA----
75 Tiger_frog_virus                             13.1%   --TRGSSVPFVWVTFRCWFDAR-KASDV-------------------------------LVKAGF----ACH--QCRA----
76 Tokyovirus_A1                                12.9%   ----KIPVDVLKLEFRTLSAMR-ACARAMET--------RQHR--------IDGVGSFDAKEL----RTH--EQNI----
77 Trichoplusia_ni_ascovirus_2c                 10.4%   ---ARHDKYFCKVSFSSDIGKR-AFVMRVNGTVDRGSAATANRKGGKIHQPMKFPNGLTGDMI----KIH--GIDV----
78 Tunisvirus_fontaine2                         12.2%   ----KIPVQTLRLEFMSIAGAK-MCSNAMEN--------RENR--------VENLGVFAPKEL----RAH--EQNI----
79 Vaccinia_virus                               10.6%   ----IDIDETISYNLDIKDRKC-SVADM-----------------------WLIEEPKKRSIQ----NAT--MDEF----
80 Variola_virus                                10.3%   ----IDIDETISYNLDIKDRKC-SVADM-----------------------WLIEEPKKRNIQ----NAT--MDEF----
81 Volepox_virus                                10.8%   ----IDIDETISYNLDIKDRKC-SVADM-----------------------WLIEEPKKRNIQ----NAT--MDEF----
82 Wiseana_iridescent_virus                     14.4%   EEYTFKLFPYLLCSFSSYNTLKYTFPNKLKT--------------------PQRIVGLGNLKM----SVH--GQDA----
83 Yaba_monkey_tumor_virus                      10.8%   ----IDISENISYVVSNETRKK-VDVDI-----------------------WVILEPKKRVLS----DTV--MGDF----
84 Yellowstone_lake_phycodnavirus_1             17.0%   F-QNNLLSRFARIEFDTMRQMR-FCAY------------------------GLRKSDTEFGKL----KLY--ETNI----
   consensus/100%                                       ..........h.....................................................................
   consensus/90%                                        .........hh.h.h.....hp .h..h...          .........   ...........    .hh...tp.
   consensus/80%                                        ....ht.h.hh.h.htsh.thp .h.ph...          ....        ......h.tt.h   thh   tpph
   consensus/70%                                         .ttp.h.hlth.Fpsh.thc thtph...           ...         .....httt.h    phh   pppp

                                                     161          .         .         .         2         .         .         .         .         .  24[illegible]
 1 Acanthamoeba_castellanii_mamavirus          100.0%   -EPHIRFMHIN-NLSSCGWASIDK----DKLKKIPEYSN---CDYSFSV--------NW-------KDVKPSNN---DDRM--AP
 2 Acanthamoeba_polyphaga_mimivirus             98.6%   -EPHIRFMHIN-NLSSCGWASIDK----DKLKKIPEYSN---CDYSFSV--------NW-------KDVKPSNN---DDRM--AP
 3 Acanthamoeba_polyphaga_moumouvirus           56.0%   -EPHIRFMHIN-NLSSCGWVTIDK----SHLKENKEYSN---CDHSYSV--------NW-------KYVKPADN---DDRM--AP
 4 Amsacta_moorei_entomopoxvirus                10.1%   -NQYRWFYVLN-NIEPSGSYRINM----DNMQKIKTYNK---NKTSYYC-------------KNPKLLFSNYVKIDKH--IP
 5 Anomala_cuprea_entomopoxvirus                 9.7%   -NFYRWFYVLN-DINPAGSYKINL-KYCKEFNVQNKFNS-----KTYYC-------------DDPKLLFSNKITSDKF--LP
 6 ASFV_BAV71                                    9.7%   -TCYYRMVSRELKLPLTSWIQLQH----YSYEPRGLVHR-----------FSV--------------TPEDLVSYQ---DDGP--TD
 7 ASFV_E75                                      9.7%   -TCYYRMVSRELKLPLTSWIQLQH----YSYEPRGLVHR-----------FSV--------------TPEDLVSYQ---DDGP--TD
 8 Brazilian_marseillevirus                     12.6%   -DVYTKFFTHR-VLDSTGWIKVVE----EKGLAYEKNSK---ADKEYVC--------DW-------RNVSKGE---DMGL--IN
 9 Cafeteria_roenbergensis_virus_BV-PW1         20.9%   -TPMLRCFHIQ-DIPGCGWVRAEN----FINIKTKNKEST---CNYEIVL-------------SYKDIKPIV---KESN--AP
10 Cannes_8_virus                               13.3%   -DTYTKFFTHR-SINSTGWISVKE----KAG-KAYQKFSK---ADIEFSC--------FW-------KHVAPGD---DEGL--VS
11 Catovirus_CTV1                               24.3%   -EPFLRCMHIR-NLDAVGWVKIES----NKIIALPNETC---CDINIST--------KW-------NNLTRVE---ERTI--MP
12 Cedratvirus_A11                              12.1%   FTPIRKLLTER-GCGYAQWFEIST------------SP---CRQPTST-----IPEWTGDHKTMKPRP---DIKTITSP
13 Choristoneura_biennis_entomopoxvirus         10.5%   -NQYRWFYILN-NIEPAGSYKINM----SNLQNINIYDK---NKTAYYC-------------KNPKLLFLTPIEIDKF--IP
14 Common_midwife_toad_ranavirus                13.2%   -QPVLQLTSLK-DLPTCGWIRLGK----GAVQVKREKYSM---CKREFIV--------QW-----EQVERGP---DVPQ--PE
15 Cowpox_virus                                 10.5%   -LNISWFYISN-GISPDGCYSLDE----QYLTKINNGCYH---CDDPRNC-------------FAKEIPRFD--------IP
16 Diadromus_pulchellus_ascovirus_4a            12.7%   -GTEILFMIER-DCDASGWLNISN----AECSNEKLTL----CENEYRC-------------KSADISRSD---RVDP--VD
17 Ectromelia_virus                             10.5%   -LNISWFYISN-GISPDGCYSLDE----QYLTKINNGCYH---CDDPRNC-------------FAKEIPRFD--------IP
18 Emiliania_huxleyi_virus_145                  14.6%   -DPGSMFMERN-GLTPCGWFKMKA----CKTITKHKISH---CTMEFEV-------------SNPKNIQPVD---LDKI--AP
19 Emiliania_huxleyi_virus_86                   14.6%   -DPGSMFMERN-GLTPCGWFKMKA----CKTITKHKISH---CTMEFEV-------------SNPKNIQPVD---LDKI--AP
20 European_catfish_virus                       13.1%   -QPVLQLTSLR-DLPTCGWIRLGK----GAVQVKREKYSR---CKREFIV--------QW-----EQVERGP---DVPQ--PE
21 Faustovirus_D3                                8.8%   SGYYFNKVARERGINTAGWNLLACDEPGAIREIFAPHGALNNFAKGYEC---------AINCLTRCKNKIKAGL--DP
22 Faustovirus_E12                               8.9%   SGYYFNKIARERGINTAGWNLIASENPADITGINSPHCSLNNFTKGYEC---------NINCLTKCRNKIKAQL--DP
```

```
23 Faustovirus_E24                              8.9%   SGYYFNKIARERGINTAGWNLIASENPADITGINSPHCSLNNFTKGYEC-----------NINCLTKCRNKIKAQL--DP
24 Faustovirus_E9                               9.0%   SGYYFNKMARERGINTAGWNIISSDQPGAITEIYAPYGPLRNFDKGYEC-----------EMNCLTRCKNKIKARL--DP
25 Faustovirus_Liban                            9.0%   SGYYFNKIARERGINTAGWNLIASENLADITEINSPHCSLNNFTKGYEC-----------NINCLTKCRNKIKAQL--DP
26 Feldmannia_species_virus                    13.4%   -DPLLKFFHSS-GIKPSSYFEMDG----FVVWNGQGKTH---CSREYYV-----------AVQNLRPSA---DSGSP-PP
27 Frog_virus_3                                13.2%   -QPVLQLTSLR-DLPTCGWIRLGK---GAVQVKREKYSR---CKREFIV-------QW----EQVERGP---DVPQ--PE
28 Heliothis_virescens_ascovirus_3e            11.3%   -PTELQVLVNA-KLPFAGWIETGR-----LYSPKDRKTR---CDREYSV-----------DIKQVRTSTSM-ACVT--PP
29 Hokovirus_HKV1                              20.3%   -LPNLRFMHER-DLWSVGYAKILA----KKYKNINNDSN---NTYNIEC-------DW----RDIIKVE---DDTI--VK
30 Infectious_spleen_and_kidney_necrosis_virus 12.7%   -SKTMEFTARA-GLPTVGWIQVSQ-----RCVVTRTVTM---AAKEYMVP------NW---RTDVRPAPD--MEGV--PP
31 Insectomime_virus                           12.3%   -DIYTKLFTHR-GLDSTGWIKVVE----KKGLVYEKFSK---ADNEFSC-------DW----NDVSLGE---NRGL--VN
32 Invertebrate_iridescent_virus_22            13.4%   -CPRLQLTSKY-DLPTAGWIKFKG---IEVVCEEDKFTS---CDREFLVDLRLCKTQGIKPKDLISRSV---KTTI--PK
33 Invertebrate_iridescent_virus_3             14.2%   -CPRLQLTSKY-NLPTAGWIQFKG---IEILDQDVKFSQ---SCREFMVD--LSDKNWGKPSTMICRSD---KTTV--PR
34 Kaumoebavirus                               10.2%   -SSYYRKVARETKMTMAGWNYVSE--YEEVDEEDEDASE---DIKTFRV-----------DIRNIREVPQEDKTPM--DP
35 Klosneuvirus_KNV1                           15.8%   -EPNLRFMHIR-DLQAVGWVSIKG---GKYNCFPKDKSP---SNNDICI-----HTNW----KNLDPVK---DTSI--VP
36 Kurlavirus_BKC-1                            12.6%   -DIYTKLFTHR-KLDSTGWLKVVE----NKGATYDKFSK---ADLEFSC-------DW----KNVSLGE---NKGL--IA
37 Lausannevirus                               12.4%   -DIYTKLFTHR-KLDSTGWLKVVE----NKGVTYDKFSK---ADLEFSC-------EW----RNISLGE---NKGL--IA
38 Lumpy_skin_disease_virus_NI-2490            10.5%   -LNVSWFFISN-NISPEGCYSINV---EKLKKINEQCYH---CDCPKEC-----------FATPINRFN---------IN
39 Lymphocystis_disease_virus                  13.5%   -DAVLQMRAIR-KLPAVGWIEATI------FSTSQYITS---CQIEISV-----------KYDRLLPYK---CDEI--PK
40 Marseillevirus_marseillevirus_T19           13.3%   -DTYTKFFTHR-SINSTGWISVKE----KAGKAYQKFSK---ADIEFSC-------FW----KHVAPGD---DEGL--VS
41 Megavirus_chiliensis                        60.1%   -EPHIRCMHIN-NLSSCGWVTIDN----KNLKNNSEYSN---CDYSYSV-------QW----KHIKPSSN--DDRM--AP
42 Megavirus_courdo7                           60.4%   -EPHIRCMHIN-NLSSCGWVTIDN----KNLKNNSEYSN---CDYSYSV-------QW----KHIKPSSN--DDRM--AP
43 Megavirus_ursino                            60.1%   -EPHIRCMHIN-NLSSCGWVTIDN----KNLKNNSEYSN---CDYSYSV-------QW----KHIKPSSN--DDRM--AP
44 Melbournevirus                              13.3%   -DTYTKFFTHR-SINSTGWISVKE----KAGKAYQKFSK---ADIEFSC-------FW----KHVAPGD---DEGL--VS
45 Micromonas_pusilla_virus_SP1                16.3%   -DPVLRLMHRT-GIQSTGWLDTGD------KCIRSHLAN---VDIDLFCN------DW----TTLTPVN---RDDI--AP
46 Mollivirus_sibericum                         9.9%   IGGVHKLLDDI-NILPSSWITISQ---WDVPERYYTHSQ---IEVEAHR-------------CDIKPAEGKITERV--AK
47 Molluscum_contagiosum_virus_subtype_1        9.1%   -MHITWFFLRN-EIEPNGCYRVPD---AELEPVCARCFH---CAAPRRA-----------FATRVPLFE---------VT
48 Monkeypox_virus                             10.5%   -LNISWFYISN-GISPDGCYSLDE---QYLTKINNGCYH---CDDPRNC-----------FAKEIPRFD---------IP
49 Myxoma_virus                                10.7%   -LNITWFFISN-DVSPEGCYRIDV---SKLEKLNNKCYH---CNDPKDL-----------FSNPISRFE---------VP
50 Noumeavirus                                 12.5%   -DIYTKLFTHR-KLDSTGWLKVVE----NKGATYDKFSK---ADLEFSC-------DW----KNVSLGE---NKGL--IA
51 Orf_virus                                   10.2%   -LNVSWFFVAH-DIDPDGCYRVDP---ALLRDLGSNCFH---CDDPGAC-----------FAEKIPRFN---------VT
52 Organic_Lake_phycodnavirus_1                18.2%   -PPLLRFFHIQ-NISPSGWIEVKR-----FSRNKMKKTH---MDVDINC-----------DYKNIYPLD---KEIM--VP
53 Orpheovirus_LCC2                            12.8%   -SPSWKMLTMT-NCAYSQWFTVDA----HRVPVAEKVST---LENEFIVY------DW-TKLRALPPEQT--SGWV--TN
54 Ostreococcus_lucimarinus_virus_1            16.7%   -DPVLRLMHRT-GIQSTGWLETGD------KCIRSHLAN---VDMDLFCN------DW----TTLKPVA---RDDV--AP
55 Ostreococcus_tauri_virus_OtV5               16.7%   -DPVLRLMHRT-GIQSTGWLDTGD------KCIRSHLAR---VDLDLFC------NDW----TTLKPVA---RDDI--AP
56 Pacmanvirus_A23                              9.1%   -NYYFPKIAREFKFATADWNRIEE---YCVVNPSSVTTN---CTYVLKVNVQNFKKLTKEKRREYLKPGQLLEKVIERDS
57 Pandoravirus_dulcis                          9.2%   -DPVHKALERLGGLQPCSWFDVRR---WRVPELYHTHAQ---IEVEVDA-------------RDIVPRPE--IDVM--AP
58 Pandoravirus_inopinatum                      8.8%   -DPVHKALERLGGLQPCSWFDVQR---WRVPELYHTHAQ---IEVEADV-------------RDIVARPE--IDVM--AP
59 Pandoravirus_salinus                        10.5%   -DPVHKALERLGGLQPCSWFDVQR---WRIPDLYHTHAQ---IEVEADA-------------RDIVPRPE--IDAM--AP
60 Paramecium_bursaria_Chlorella_virus_CVK2    15.4%   -DPIIRVFHLR-NINPADWIRVSK-----AYPAQTRISN---SDIEVET-----------SFQHLGPVD---DKTV--PP
61 Paramecium_bursaria_chlorella_virus_MT325   15.1%   -DPIVRLFHIR-KINPAGWVQIKQ-----SYPVMTRISR---SDIEVDC-----------NFTTVSGSE---LTTP--PP
62 Paramecium_bursaria_Chlorella_virus_NYs1    14.2%   -DPIIRIFHLR-NINPADWMHVSK-----AFPVETRISN---SDIEVET-----------SFQHLGPSD---VKEV--PP
63 Phaeocystis_globosa_virus                   16.9%   -PPLLKLFHIQ-EISPSGWIQIQS---DKITKMRQKSTH---CAYEYIT-----------SYKHLKKAD---KDDI--VK
64 Phaeocystis_globosa_virus_14T               16.8%   -PPLLKLFHIQ-EISPSGWIQIQS---DKITKMRQKSTH---CAYEYIT-----------SYKHLKKAD---KDDI--VK
65 Pithovirus_massiliensis                     11.6%   -SPVRRLLTER-DCRFAQWFRISA---VEVPENHKVS-----TISEFQG-------DW-RTLKSYSAIES--KGWV--TA
66 Pithovirus_sibericum                        11.8%   -SPVRRLLTER-DCRFAQWFRISV-----VEVPENHKVS---TISEFQG-------DW----RTLKSYSAIESKGWVTAP
67 Port-miou_virus                             12.4%   -DIYTKLFTHR-KLDSTGWLKVVE----NKGVTYDKFSK---ADLEFSC-------DW----RNISLGE---NKGL--IA
68 Powai_lake_megavirus                        60.0%   -EPHIRCMHIN-NLSSCGWVTIDN----KNLKNNSEYSN---CDYSYSV-------QW----KHIKPSNN--DDRM--AP
69 Scale_drop_disease_virus                    12.8%   -SKTLQFVSCA-QLPTAGWIQVSK---KCIKPVDKHVTT---CDVEYLVF------DW--QKQIQSVTE---ELLP--AT
70 Short-finned_eel_ranavirus                  13.1%   -QPVLQLTSLG-DLPTCGWIRLGK---GAVRVKREKYSR---CKREFIV-------QW----DQVERGP---DVPQ--PE
71 Singapore_grouper_iridovirus                13.3%   -QPVLQLLSRA-DLPTCGWITTKE---PPVCVKRDKYSK---CKYEYVT-------DW----KNLSECD---RDDQ--PL
72 Skunkpox_virus                              11.0%   -LNISWFYISN-GISPDGCYSLDE---QYLTKINNGCYH---CDDPRSC-----------FAKEIPRFD---------IP
73 Spodoptera_frugiperda_ascovirus_1a          10.9%   -PTELQVLVQH-KLPFAGWVETGR-----LYEPRSRLTR---CDREYCT-----------DLPALKSSELL-ACVA--PS
74 Testudo_hermanni_ranavirus                  13.2%   -QPVLQLTSLK-DLPTCGWIRLGK---GAVQVKREKYSR---CKREFIV-------QW----EQVERGP---DVPQ--PE
75 Tiger_frog_virus                            13.1%   -QPVLQLTSLR-DLPTCGWIRLGK---GAVQVKREKYSR---CKREFIV-------QW----EQVERGP---DVPQ--PE
76 Tokyovirus_A1                               12.9%   -DTYTKLFTLR-SIDSTGWISVKE----KAGKNYEKFSK---ADVEFSC-------FW----KHISTGE---NEGS--AP
77 Trichoplusia_ni_ascovirus_2c                10.4%   -PTELQVLEEL-DIPPCGWIETHN--AYKCATITKRFTH------QYRA-----------NVKTFKYCT---RMECIPPV
78 Tunisvirus_fontaine2                        12.2%   -DIYTKLFTHR-GLDSTGWIKVVE----KKGLVYEKFSK---ADNEFSC-------DW----NDVSLGE---NRGL--VN
79 Vaccinia_virus                              10.6%   -LNISWFYISN-GISPDGCYSLDE---QYLTKINNGCYH---CDDPRNC-----------FAKKIPRFD---------IP
80 Variola_virus                               10.3%   -LNISWFYISN-GISPDGCYSLDD---QYLTKINNGCYH---CGDPRNC-----------FAKEIPRFD---------IP
81 Volepox_virus                               10.8%   -LNISWFYISN-RISPDGCYSLDE---QYLTKINNGCYH---CDDPRSC-----------FAKEIPRFD---------IP
82 Wiseana_iridescent_virus                    14.4%   -CPRLQLTSKY-NLPTAGWIKFKG---TEIINEEDKFTS---CDREFLVDLGLCKAHNIKPEDLISRSS---KTTI--PK
83 Yaba_monkey_tumor_virus                     10.8%   -LNITWFYMLN-NVCPDGCYVIDI---KMLEKINDNCYH---CNDPQQL-----------FLNPIPKFD---------VS
84 Yellowstone_lake_phycodnavirus_1            17.0%   -DPVLRFMHVT-GIRSTGWLTCAA-----TEPDYDTT-----CDINLWA----------PEHTNIKPVD---RDDV--AP
   consensus/100%                                      ......hh.....h...t...h..........................h...............................
   consensus/90%                                        ..h..hh..t.tl..suhhph..   .......t.h..   .t..h.s.      ..  ...ptl...........  ..
   consensus/80%                                        ..hh.hh..p tlsssGahpltt   ..h....pthsp   sp.th.s       ..   ..ppl...t.  ....  .s
   consensus/70%                                        tshhphhthp slsssGWhplsp   ..hhth.pphsp   sshphts       ..   ..pplt.sp.  pt..  ss

                                                    241       :         .         .         .         .         3         .         . 32
 1 Acanthamoeba_castellanii_mamavirus         100.0%   F----------KIMGYDIECVSC--DQNFPQAERP---------------------------------------------
 2 Acanthamoeba_polyphaga_mimivirus            98.6%   F----------KIMGYDIECVSC--DQNFPQAERP---------------------------------------------
 3 Acanthamoeba_polyphaga_moumouvirus          56.0%   L----------KIMGYDIECISC--DHNFPQAERE---------------------------------------------
 4 Amsacta_moorei_entomopoxvirus               10.1%   A----------SRVSIDIECQH---FGEFPTAN-----------------------------------------------
 5 Anomala_cuprea_entomopoxvirus                9.7%   V----------SRLSFDIECKH---NGIFPKPE-----------------------------------------------
 6 ASFV_BAV71                                   9.7%   HS---------IVMAYDIETYSPV-KGTVPDPNQA---------------------------------------------
 7 ASFV_E75                                     9.7%   HS---------IVMAYDIETYSPV-KGTVPDPNQA---------------------------------------------
 8 Brazilian_marseillevirus                    12.6%   T----------KKLSFDIEAYSAH-HSAFPDPNIK---------------------------------------------
 9 Cafeteria_roenbergensis_virus_BV-PW1        20.9%   L----------RICSFDIECDSC--DGMFPQANRP---------------------------------------------
10 Cannes_8_virus                              13.3%   T----------KKLSFDIEAYSKN-HSAFPDPNMK---------------------------------------------
11 Catovirus_CTV1                              24.3%   F----------VIASFDLECTSE--DGSFPQAERD---------------------------------------------
12 Cedratvirus_A11                             12.1%   -----------KIFSFDIECYSNN-HRAMPKEYIP---------------------------------------------
13 Choristoneura_biennis_entomopoxvirus        10.5%   V----------SRVSIDIECQH---FGEFPTPN-----------------------------------------------
14 Common_midwife_toad_ranavirus               13.2%   V----------TVVALDLEVNSEV-ENAMPK-DRP---------------------------------------------
15 Cowpox_virus                                10.5%   R----------SYLFLDIECHF---DKKFPSVF-----------------------------------------------
16 Diadromus_pulchellus_ascovirus_4a           12.7%   V----------KIMAWDIEARCKN-IGKDPGSDS----------------------------------------------
17 Ectromelia_virus                            10.5%   R----------SYLFLDIECHF---DKKFPSVF-----------------------------------------------
18 Emiliania_huxleyi_virus_145                 14.6%   I----------LIASYDFEMYSE--TRGFPMADRQ---------------------------------------------
19 Emiliania_huxleyi_virus_86                  14.6%   I----------LIASYDFEMYSE--TRGFPMADRQ---------------------------------------------
20 European_catfish_virus                      13.1%   V----------TVVALDLEVNSEV-ENAMPK-DRP---------------------------------------------
21 Faustovirus_D3                               8.8%   ITRRLLERPKKLSMFWDIET-----EGGRDIDDHD---------------------------------------------
22 Faustovirus_E12                              8.9%   ITRRLIERPKALSMFWDIET-----EGGRDIEDHD---------------------------------------------
23 Faustovirus_E24                              8.9%   ITRRLIERPKALSMFWDIET-----EGGRDIEDHD---------------------------------------------
24 Faustovirus_E9                               9.0%   ITRRLLERPKKLSMFWDIET-----EGGRDIEDHD---------------------------------------------
25 Faustovirus_Liban                            9.0%   ITRRLIERPKALSMFWDIET-----EGGRDIEDHD---------------------------------------------
26 Feldmannia_species_virus                    13.4%   I----------PMCSYDIES-----SGLDPA-------------------------------------------------
27 Frog_virus_3                                13.2%   V----------TVVALDLEVNSEV-ENAMPK-DRP---------------------------------------------
28 Heliothis_virescens_ascovirus_3e            11.3%   L----------KTFAWDIEAKIN--DMSSPGTHE----------------------------------------------
29 Hokovirus_HKV1                              20.3%   Y----------VVASFDLECKPEN-LDNFPKAKRP---------------------------------------------
30 Infectious_spleen_and_kidney_necrosis_virus 12.7%   A----------KIVYFDIEVKSDH-ENVFPS-DRD---------------------------------------------
31 Insectomime_virus                           12.3%   T----------KKMSFDIEAYSKH-HSAFPDPNIK---------------------------------------------
32 Invertebrate_iridescent_virus_22            13.4%   P----------LVLSFDLEVNSED-TITMPKASRP---------------------------------------------
33 Invertebrate_iridescent_virus_3             14.2%   P----------LILSFDLEVNSEE-VVTMPKATKP---------------------------------------------
34 Kaumoebavirus                               10.2%   S----------LELCWDIETMGNEQKNFGVKTDKD---------------------------------------------
35 Klosneuvirus_KNV1                           15.8%   L----------IIAAFDIECMSE--DGSFPQPQRD---------------------------------------------
36 Kurlavirus_BKC-1                            12.6%   T----------KKISFDIEAYSKH-HSAFPDPNIK---------------------------------------------
```

```
37 Lausannevirus                                  12.4%   T----------KKISFDIEAYSKH-HSAFPDPNIK---------------------------------------------
38 Lumpy_skin_disease_virus_NI-2490               10.5%   R----------SYLFLDIECQF---DKKFPSVF-----------------------------------------------
39 Lymphocystis_disease_virus                     13.5%   I----------RILALDIETASE--DEQFPK-DRP---------------------------------------------
40 Marseillevirus_marseillevirus_T19              13.3%   T----------KKLSFDIEAYSKN-HSAFPDPNMK---------------------------------------------
41 Megavirus_chiliensis                           60.1%   L----------KIMGYDIECVSC--DHNFPQADRE---------------------------------------------
42 Megavirus_courdo7                              60.4%   L----------KIMGYDIECVSC--DHNFPQADRE---------------------------------------------
43 Megavirus_ursino                               60.1%   L----------KIMGYDIECVSC--DHNFPQADRE---------------------------------------------
44 Melbournevirus                                 13.3%   T----------KKLSFDIEAYSKN-HSAFPDPNMK---------------------------------------------
45 Micromonas_pusilla_virus_SP1                   16.3%   F----------VVASFDIECNSS--TGKFPDADVN---------------------------------------------
46 Mollivirus_sibericum                            9.9%   M----------WKAAWDVEAHSTV-ARRFPNPDLV---------------------------------------------
47 Molluscum_contagiosum_virus_subtype_1           9.1%   H----------TYLFFDIECQF---DKKFPSVF-----------------------------------------------
48 Monkeypox_virus                                10.5%   R----------SYLFLDIECHF---DKKFPSVF-----------------------------------------------
49 Myxoma_virus                                   10.7%   R----------SYLFLDIECHF---NKKFPSVF-----------------------------------------------
50 Noumeavirus                                    12.5%   T----------KKISFDIEAYSKH-HSAFPDPNIK---------------------------------------------
51 Orf_virus                                      10.2%   R----------SGLFLDIECHF---EKKFPSVF-----------------------------------------------
52 Organic_Lake_phycodnavirus_1                   18.2%   Y----------KICSFDIEANSS--HGDFPEAAKDYKKVAYDIVYYLEHQNVQDYDYILRELLENVFGFKDTLLIDKCYV
53 Orpheovirus_LCC2                               12.8%   P----------AVVVFDIETYSPN-HKAMPRRNLA---------------------------------------------
54 Ostreococcus_lucimarinus_virus_1               16.7%   F----------VVASVDIECNSS--TGKFPDANIP---------------------------------------------
55 Ostreococcus_tauri_virus_OtV5                  16.7%   F----------VVASVDIECNSS--TGKFPDADVT---------------------------------------------
56 Pacmanvirus_A23                                 9.1%   T----------QVAMWDIETHRTIQNGVVPTPEDT---------------------------------------------
57 Pandoravirus_dulcis                             9.2%   V----------WKASWDVECYSR--DGSFPRADHPRG-------------------------------------------
58 Pandoravirus_inopinatum                         8.8%   V----------WKASWDVECYSR--DGSFPRADHPRG-------------------------------------------
59 Pandoravirus_salinus                           10.5%   V----------WKASWDVECYSR--DGSFPRADHPRG-------------------------------------------
60 Paramecium_bursaria_Chlorella_virus_CVK2       15.4%   L----------VIASWDIETYSK--DRKFPLAENP---------------------------------------------
61 Paramecium_bursaria_chlorella_virus_MT325      15.1%   L----------VIASWDIETYSK--ERKFPLSSNP---------------------------------------------
62 Paramecium_bursaria_Chlorella_virus_NYs1       14.2%   L----------IIASWDIETYSK--DRKFPLAENP---------------------------------------------
63 Phaeocystis_globosa_virus                      16.9%   Y----------SICSFDIEASSS--HGDFPVPIKDYKKLATNILEYYNEMEDKTKFDVTCFKKLIDAGYGYERCPDIATV
64 Phaeocystis_globosa_virus_14T                  16.8%   Y----------SICSFDIEASSS--HGDFPVPIKDYKKLATNILEYYNEMEDKTKFDVTCFKKLIDAGYGYERCPDIATV
65 Pithovirus_massiliensis                        11.6%   P----------KIFSFDIECYSDN-HKAMPKEYAI---------------------------------------------
66 Pithovirus_sibericum                           11.8%   -----------KIFSFDIECYSDN-HKAMPKEYAI---------------------------------------------
67 Port-miou_virus                                12.4%   T----------KKISFDIEAYSKH-HSAFPDPNIK---------------------------------------------
68 Powai_lake_megavirus                           60.0%   L----------KIMGYDIECVSC--DHNFPQADRE---------------------------------------------
69 Scale_drop_disease_virus                       12.8%   P----------TIAYFDLEVNSEL-VNTFPQ-DRD---------------------------------------------
70 Short-finned_eel_ranavirus                     13.1%   V----------TVVALDLEVNSEV-ENAMPK-DRP---------------------------------------------
71 Singapore_grouper_iridovirus                   13.3%   I----------TWVALDLEVNSEL-ENAMPK-DRP---------------------------------------------
72 Skunkpox_virus                                 11.0%   R----------SYLFLDIECHF---DKKFPSVF-----------------------------------------------
73 Spodoptera_frugiperda_ascovirus_1a             10.9%   L----------KTFAWDIEAKIN--DMSMPGYHV----------------------------------------------
74 Testudo_hermanni_ranavirus                     13.2%   V----------TVVALDLEVNSEV-ENAMPK-DRP---------------------------------------------
75 Tiger_frog_virus                               13.1%   V----------TVVALDLEVNSEV-ETAMPK-DRP---------------------------------------------
76 Tokyovirus_A1                                  12.9%   T----------VEMSFDIEAYSHN-HSAFPDPNIK---------------------------------------------
77 Trichoplusia_ni_ascovirus_2c                   10.4%   L----------KTMAWDIEARIH--DISSPGLHI----------------------------------------------
78 Tunisvirus_fontaine2                           12.2%   T----------KKMSFDIEAYSKH-HSAFPDPNIK---------------------------------------------
79 Vaccinia_virus                                 10.6%   R----------SYLFLDIECHF---DKKFPSVF-----------------------------------------------
80 Variola_virus                                  10.3%   R----------SYLFLDIECHF---DKKFPSVF-----------------------------------------------
81 Volepox_virus                                  10.8%   R----------SYLFLDIECHF---DKKFPSVF-----------------------------------------------
82 Wiseana_iridescent_virus                       14.4%   P----------LVLSFDLEVNSED-TVTMPKASRP---------------------------------------------
83 Yaba_monkey_tumor_virus                        10.8%   R----------SYLFLDIECHF---EKKFPSVF-----------------------------------------------
84 Yellowstone_lake_phycodnavirus_1               17.0%   L----------KIMSFDIECYSK--SGNFPDPMKT---------------------------------------------
   consensus/100%                                         .............h.hDhE..........s..................................................
   consensus/90%                                          .          .hh.hDlEs.... ptthP.....
   consensus/80%                                          h          .hh.hDlEs.... ptthPt....
   consensus/70%                                          h          hhhuaDIEs.sp. ctthPpsph.

                                                       321         .         .         :         .         .         .         .         4 40
 1 Acanthamoeba_castellanii_mamavirus            100.0%   --------------------------------------------------------------------------------
 2 Acanthamoeba_polyphaga_mimivirus               98.6%   --------------------------------------------------------------------------------
 3 Acanthamoeba_polyphaga_moumouvirus             56.0%   --------------------------------------------------------------------------------
 4 Amsacta_moorei_entomopoxvirus                  10.1%   --------------------------------------------------------------------------------
 5 Anomala_cuprea_entomopoxvirus                   9.7%   --------------------------------------------------------------------------------
 6 ASFV_BAV71                                      9.7%   --------------------------------------------------------------------------------
 7 ASFV_E75                                        9.7%   --------------------------------------------------------------------------------
 8 Brazilian_marseillevirus                       12.6%   --------------------------------------------------------------------------------
 9 Cafeteria_roenbergensis_virus_BV-PW1           20.9%   --------------------------------------------------------------------------------
10 Cannes_8_virus                                 13.3%   --------------------------------------------------------------------------------
11 Catovirus_CTV1                                 24.3%   --------------------------------------------------------------------------------
12 Cedratvirus_A11                                12.1%   --------------------------------------------------------------------------------
13 Choristoneura_biennis_entomopoxvirus           10.5%   --------------------------------------------------------------------------------
14 Common_midwife_toad_ranavirus                  13.2%   --------------------------------------------------------------------------------
15 Cowpox_virus                                   10.5%   --------------------------------------------------------------------------------
16 Diadromus_pulchellus_ascovirus_4a              12.7%   --------------------------------------------------------------------------------
17 Ectromelia_virus                               10.5%   --------------------------------------------------------------------------------
18 Emiliania_huxleyi_virus_145                    14.6%   --------------------------------------------------------------------------------
19 Emiliania_huxleyi_virus_86                     14.6%   --------------------------------------------------------------------------------
20 European_catfish_virus                         13.1%   --------------------------------------------------------------------------------
21 Faustovirus_D3                                  8.8%   --------------------------------------------------------------------------------
22 Faustovirus_E12                                 8.9%   --------------------------------------------------------------------------------
23 Faustovirus_E24                                 8.9%   --------------------------------------------------------------------------------
24 Faustovirus_E9                                  9.0%   --------------------------------------------------------------------------------
25 Faustovirus_Liban                               9.0%   --------------------------------------------------------------------------------
26 Feldmannia_species_virus                       13.4%   --------------------------------------------------------------------------------
27 Frog_virus_3                                   13.2%   --------------------------------------------------------------------------------
28 Heliothis_virescens_ascovirus_3e               11.3%   --------------------------------------------------------------------------------
29 Hokovirus_HKV1                                 20.3%   --------------------------------------------------------------------------------
30 Infectious_spleen_and_kidney_necrosis_virus    12.7%   --------------------------------------------------------------------------------
31 Insectomime_virus                              12.3%   --------------------------------------------------------------------------------
32 Invertebrate_iridescent_virus_22               13.4%   --------------------------------------------------------------------------------
33 Invertebrate_iridescent_virus_3                14.2%   --------------------------------------------------------------------------------
34 Kaumoebavirus                                  10.2%   --------------------------------------------------------------------------------
35 Klosneuvirus_KNV1                              15.8%   --------------------------------------------------------------------------------
36 Kurlavirus_BKC-1                               12.6%   --------------------------------------------------------------------------------
37 Lausannevirus                                  12.4%   --------------------------------------------------------------------------------
38 Lumpy_skin_disease_virus_NI-2490               10.5%   --------------------------------------------------------------------------------
39 Lymphocystis_disease_virus                     13.5%   --------------------------------------------------------------------------------
40 Marseillevirus_marseillevirus_T19              13.3%   --------------------------------------------------------------------------------
41 Megavirus_chiliensis                           60.1%   --------------------------------------------------------------------------------
42 Megavirus_courdo7                              60.4%   --------------------------------------------------------------------------------
43 Megavirus_ursino                               60.1%   --------------------------------------------------------------------------------
44 Melbournevirus                                 13.3%   --------------------------------------------------------------------------------
45 Micromonas_pusilla_virus_SP1                   16.3%   --------------------------------------------------------------------------------
46 Mollivirus_sibericum                            9.9%   --------------------------------------------------------------------------------
47 Molluscum_contagiosum_virus_subtype_1           9.1%   --------------------------------------------------------------------------------
48 Monkeypox_virus                                10.5%   --------------------------------------------------------------------------------
49 Myxoma_virus                                   10.7%   --------------------------------------------------------------------------------
50 Noumeavirus                                    12.5%   --------------------------------------------------------------------------------
```

```
51 Orf_virus                                       10.2%   --------------------------------------------------------------------------------
52 Organic_Lake_phycodnavirus_1                    18.2%   EQSYTYEQFESHMELLFKRKIATNYVVANKLKEIFSKDDEDDEESSYVKLKKINMNDVLTMLKDITIDKPNKIVHLTNVL
53 Orpheovirus_LCC2                                12.8%   --------------------------------------------------------------------------------
54 Ostreococcus_lucimarinus_virus_1                16.7%   --------------------------------------------------------------------------------
55 Ostreococcus_tauri_virus_OtV5                   16.7%   --------------------------------------------------------------------------------
56 Pacmanvirus_A23                                  9.1%   --------------------------------------------------------------------------------
57 Pandoravirus_dulcis                              9.2%   --------------------------------------------------------------------------------
58 Pandoravirus_inopinatum                          8.8%   --------------------------------------------------------------------------------
59 Pandoravirus_salinus                            10.5%   --------------------------------------------------------------------------------
60 Paramecium_bursaria_Chlorella_virus_CVK2        15.4%   --------------------------------------------------------------------------------
61 Paramecium_bursaria_chlorella_virus_MT325       15.1%   --------------------------------------------------------------------------------
62 Paramecium_bursaria_Chlorella_virus_NYs1        14.2%   --------------------------------------------------------------------------------
63 Phaeocystis_globosa_virus                       16.9%   YPKLKNISKEQLDNIFSNFMLYIPAKDQSRKDYIKENEESDTDSDNDDEDDKKENDGADEAAAFHKRHKKVKKYHKTANL
64 Phaeocystis_globosa_virus_14T                   16.8%   YPKLKNISKEQLDNIFSNFMLYIPAKDQSRKDYIKENEESDTDSDNDDEDDKKENDGADEAAAFHKRHKKVKKYHKTANL
65 Pithovirus_massiliensis                         11.6%   --------------------------------------------------------------------------------
66 Pithovirus_sibericum                            11.8%   --------------------------------------------------------------------------------
67 Port-miou_virus                                 12.4%   --------------------------------------------------------------------------------
68 Powai_lake_megavirus                            60.0%   --------------------------------------------------------------------------------
69 Scale_drop_disease_virus                        12.8%   --------------------------------------------------------------------------------
70 Short-finned_eel_ranavirus                      13.1%   --------------------------------------------------------------------------------
71 Singapore_grouper_iridovirus                    13.3%   --------------------------------------------------------------------------------
72 Skunkpox_virus                                  11.0%   --------------------------------------------------------------------------------
73 Spodoptera_frugiperda_ascovirus_1a              10.9%   --------------------------------------------------------------------------------
74 Testudo_hermanni_ranavirus                      13.2%   --------------------------------------------------------------------------------
75 Tiger_frog_virus                                13.1%   --------------------------------------------------------------------------------
76 Tokyovirus_A1                                   12.9%   --------------------------------------------------------------------------------
77 Trichoplusia_ni_ascovirus_2c                    10.4%   --------------------------------------------------------------------------------
78 Tunisvirus_fontaine2                            12.2%   --------------------------------------------------------------------------------
79 Vaccinia_virus                                  10.6%   --------------------------------------------------------------------------------
80 Variola_virus                                   10.3%   --------------------------------------------------------------------------------
81 Volepox_virus                                   10.8%   --------------------------------------------------------------------------------
82 Wiseana_iridescent_virus                        14.4%   --------------------------------------------------------------------------------
83 Yaba_monkey_tumor_virus                         10.8%   --------------------------------------------------------------------------------
84 Yellowstone_lake_phycodnavirus_1                17.0%   --------------------------------------------------------------------------------
   consensus/100%                                          ................................................................................
   consensus/90%
   consensus/80%
   consensus/70%

                                                    401         .         .         .         .         :         .         .         . 48
 1 Acanthamoeba_castellanii_mamavirus             100.0%   ------------------------------SDKIIQIGITMYRYG---SMKC-----YEQHIL-----------------
 2 Acanthamoeba_polyphaga_mimivirus                98.6%   ------------------------------SDKIIQIGITMYRYG---SM-KC----YEQHIL-----------------
 3 Acanthamoeba_polyphaga_moumouvirus              56.0%   ------------------------------TDKIIQIGITMYRYG---SM-EC----YEQHIL-----------------
 4 Amsacta_moorei_entomopoxvirus                   10.1%   ------------------------------KFPISHICIDWYMDK---NTN------PIKKII-----------------
 5 Anomala_cuprea_entomopoxvirus                    9.7%   ------------------------------TCPISHICAEWYDDG---DSSI-----SKKKIF-----------------
 6 ASFV_BAV71                                       9.7%   ------------------------------NDVVFMICMRIFWIH---ST-EP----LASTCI-----------------
 7 ASFV_E75                                         9.7%   ------------------------------NDVVFMICMRIFWIH---STEP-----LASTCI-----------------
 8 Brazilian_marseillevirus                        12.6%   ------------------------------ENAITQIAYTVEETN---GG-------IWKEVI-----------------
 9 Cafeteria_roenbergensis_virus_BV-PW1            20.9%   ------------------------------NDNIIQIGCTYTLLG---KS-EP----YRQYIC-----------------
10 Cannes_8_virus                                  13.3%   ------------------------------ENVVTHVAWTVSDLD---GT-------IFKEVL-----------------
11 Catovirus_CTV1                                  24.3%   ------------------------------GDQIIQIGTTFSRFG---EN-EC----FYQHIL-----------------
12 Cedratvirus_A11                                 12.1%   ------------------------------EHVVYMISCVVFRNG---RPE------REKYLI-----------------
13 Choristoneura_biennis_entomopoxvirus            10.5%   ------------------------------KFPISHICIDWFMES---NIN------PVKKII-----------------
14 Common_midwife_toad_ranavirus                   13.2%   ------------------------------GDEVFMAGAIILRPG---KK-------PKRVLL-----------------
15 Cowpox_virus                                    10.5%   ------------------------------INPISHTSYCYIDLS------------GKRLLF-----------------
16 Diadromus_pulchellus_ascovirus_4a               12.7%   ------------------------------TDCVYQISCVFWYKD---R--------ISNHLL-----------------
17 Ectromelia_virus                                10.5%   ------------------------------INPISHTSYCYIDLS------------GKRLLF-----------------
18 Emiliania_huxleyi_virus_145                     14.6%   ------------------------------GDHIAIIGVAFWRLGTPVED-------TKTVLL-----------------
19 Emiliania_huxleyi_virus_86                      14.6%   ------------------------------GDHIAIIGVAFWRLGTPVED-------TKTVLL-----------------
20 European_catfish_virus                          13.1%   ------------------------------GDEVFMAGAIILRPG---KK-------PKRVLL-----------------
21 Faustovirus_D3                                   8.8%   ------------------------------NMDVFNISMMFYTLN------------SDKPLL-----------------
22 Faustovirus_E12                                  8.9%   ------------------------------NMDVFNISMMFYTLS------------SDKPIL-----------------
23 Faustovirus_E24                                  8.9%   ------------------------------NMDVFNISMMFYTLS------------SDKPIL-----------------
24 Faustovirus_E9                                   9.0%   ------------------------------NMDVFNISMMFYTLN------------SDRPIL-----------------
25 Faustovirus_Liban                                9.0%   ------------------------------NMDVFNISMMFYTLS------------SDKPIL-----------------
26 Feldmannia_species_virus                        13.4%   ------------------------------SDYVFQVSLCFGFLG---EDLDSRSAISDSFVI-----------------
27 Frog_virus_3                                    13.2%   ------------------------------GDEVFMAGAIILRPG---KK-------PKRVLL-----------------
28 Heliothis_virescens_ascovirus_3e                11.3%   ------------------------------DDEVYMISISVSD--------------NTDHLI-----------------
29 Hokovirus_HKV1                                  20.3%   ------------------------------NDKVIQIGTTFNYYG---NA-DC----YKSHII-----------------
30 Infectious_spleen_and_kidney_necrosis_virus     12.7%   ------------------------------DEVIFQIGLVLCSGN---TV-------LRTDLL-----------------
31 Insectomime_virus                               12.3%   ------------------------------ENAVTQIAYTIEETD---GT-------IWKEAI-----------------
32 Invertebrate_iridescent_virus_22                13.4%   ------------------------------GDVVFQISCVFSRLG---SNV------YDKYLL-----------------
33 Invertebrate_iridescent_virus_3                 14.2%   ------------------------------GDVVFQISCIFNRLGGVENE-------VERYLL-----------------
34 Kaumoebavirus                                   10.2%   ------------------------------GGKVFAICVAICRVS---QK-KP----LCTIGL-----------------
35 Klosneuvirus_KNV1                               15.8%   ------------------------------GDKIIQIGTTFSRYG---ED-EC----FYKHII-----------------
36 Kurlavirus_BKC-1                                12.6%   ------------------------------ENAVTQAAYTVEETN---GE-------IWKEAI-----------------
37 Lausannevirus                                   12.4%   ------------------------------ENAVTQVAYTVEETN---GE-------IWKEAI-----------------
38 Lumpy_skin_disease_virus_NI-2490                10.5%   ------------------------------TNPVSHISCCYIDLT------------NLEFKF-----------------
39 Lymphocystis_disease_virus                      13.5%   ------------------------------GDEIFQISLIFE---------------ERKILL-----------------
40 Marseillevirus_marseillevirus_T19               13.3%   ------------------------------ENVVTHVAWTVSDLD---GT-------IFKEVL-----------------
41 Megavirus_chiliensis                            60.1%   ------------------------------TDKIIQIGITMYRYG---SM-EC----YEQYIL-----------------
42 Megavirus_courdo7                               60.4%   ------------------------------TDKIIQIGITMYRYG---SM-EC----YEQYIL-----------------
43 Megavirus_ursino                                60.1%   ------------------------------TDKIIQIGITMYRYG---SM-EC----YEQYIL-----------------
44 Melbournevirus                                  13.3%   ------------------------------ENVVTHVAWTVSDLD---GT-------IFKEVL-----------------
45 Micromonas_pusilla_virus_SP1                    16.3%   ------------------------------GDACFQIAISLCKFG---TD-EP----YEKICL-----------------
46 Mollivirus_sibericum                             9.9%   ------------------------------DNVTICINTCVSQHDREADKDGV----VNKCHY-----------------
47 Molluscum_contagiosum_virus_subtype_1            9.1%   ------------------------------SNPVSHISFCYVDRL------------GTEVRF-----------------
48 Monkeypox_virus                                 10.5%   ------------------------------INPISHTSYCYIDLS------------GKRLLF-----------------
49 Myxoma_virus                                    10.7%   ------------------------------INPVSHISCCYIDLS------------NDEYRF-----------------
50 Noumeavirus                                     12.5%   ------------------------------ENAVTQVAYTVEETN---GE-------IWKEAI-----------------
51 Orf_virus                                       10.2%   ------------------------------KNAVSHISFCVVDKD------------GAEKRF-----------------
52 Organic_Lake_phycodnavirus_1                    18.2%   DETFPQLK----------------------GDQVTFIGSTFVNYG---EE-KP----YLNHCI-----------------
53 Orpheovirus_LCC2                                12.8%   ------------------------------KHVAYTISCIYQKYG---KPET-----RKRYNI-----------------
54 Ostreococcus_lucimarinus_virus_1                16.7%   ------------------------------GDACFQIAISLCKFG---SD-EP----YDKTCL-----------------
55 Ostreococcus_tauri_virus_OtV5                   16.7%   ------------------------------GDACFQIAISLCKFG---SD-EP----YDKTCL-----------------
56 Pacmanvirus_A23                                  9.1%   ------------------------------DYTIFMMCSAYFWHH---SD-EP----LFEVCA-----------------
57 Pandoravirus_dulcis                              9.2%   ------------------------------CDHTICINTYFSQHR---SDGRPPRVVQTSHHFGAVRVHRPVEAERDRVD
58 Pandoravirus_inopinatum                          8.8%   ------------------------------CDHTICINTYFSQHR---SDGRPPRVVQTSHHFGAVRVHRPVDAERDRVD
59 Pandoravirus_salinus                            10.5%   ------------------------------CDHTICINTYFSQHR---TDGRPPRVVQTSHHFGAVRVHRPVDAERDRVD
60 Paramecium_bursaria_Chlorella_virus_CVK2        15.4%   ------------------------------TDYCIQIATTFQKYG---EP-EP----YRRVVV-----------------
61 Paramecium_bursaria_chlorella_virus_MT325       15.1%   ------------------------------TDYVTQIATSFQRYG---EE-EP----YRRVVV-----------------
62 Paramecium_bursaria_Chlorella_virus_NYs1        14.2%   ------------------------------ADYCIQIATTFQKYG---EP-EP----YRRVVV-----------------
63 Phaeocystis_globosa_virus                       16.9%   LQIIHDDKCENDTKLFELNKALTKFYPELEGDMVTFIGMTFLNYS---EK-KP----HTRYII-----------------
64 Phaeocystis_globosa_virus_14T                   16.8%   LQIIHDDKCENDTKLFELNKALTKFYPELEGDMVTFIGMTFLNYS---EK-KP----HTRYII-----------------
```

```
65 Pithovirus_massiliensis                          11.6%  ------------------------------TNVVYMISCVVFRNK---TDE------RQKYLI-----------------
66 Pithovirus_sibericum                             11.8%  ------------------------------TNVVYMISCVVFRNK---TNE------REKYLI-----------------
67 Port-miou_virus                                  12.4%  ------------------------------ENAVTQVAYTVEETN---GE-------IWKEAI-----------------
68 Powai_lake_megavirus                             60.0%  ------------------------------TDKIIQIGITMYRYG---SM-EC----YEQYIL-----------------
69 Scale_drop_disease_virus                         12.8%  ------------------------------DDVIFQIGVVICKGA---QT-------IDSLLL-----------------
70 Short-finned_eel_ranavirus                       13.1%  ------------------------------GDEVFMAGAIILRPG---KK-------PKRILL-----------------
71 Singapore_grouper_iridovirus                     13.3%  ------------------------------GDEIFMAGVIVVTPN---RP-------PKRLLF-----------------
72 Skunkpox_virus                                   11.0%  ------------------------------INPISHTSYCYIDLS------------GKRLLF-----------------
73 Spodoptera_frugiperda_ascovirus_1a               10.9%  ------------------------------DDEVYMVSIAVSD--------------GTDHLI-----------------
74 Testudo_hermanni_ranavirus                       13.2%  ------------------------------GDEVFMAGAIILRPG---KK-------PKRVLL-----------------
75 Tiger_frog_virus                                 13.1%  ------------------------------GDEVFMAGVIILRPG---KK-------PKRVLL-----------------
76 Tokyovirus_A1                                    12.9%  ------------------------------ENVTTHVAWTVSGLD---GA-------TFKEVL-----------------
77 Trichoplusia_ni_ascovirus_2c                     10.4%  ------------------------------DDEIYMMSVSVSD--------------GKDYLL-----------------
78 Tunisvirus_fontaine2                             12.2%  ------------------------------ENAVTQIAYTIEETD---GT-------IWKEAI-----------------
79 Vaccinia_virus                                   10.6%  ------------------------------INPISHTSYCYIDLS------------GKRLLF-----------------
80 Variola_virus                                    10.3%  ------------------------------INPISHTSYCYIDLS------------GKRLLF-----------------
81 Volepox_virus                                    10.8%  ------------------------------INPISHTSCCYVDLS------------GKRLLF-----------------
82 Wiseana_iridescent_virus                         14.4%  ------------------------------GDVIFQISCVFSRLG---SND------YNKYLL-----------------
83 Yaba_monkey_tumor_virus                          10.8%  ------------------------------TNPVSHVSCCYVNLF------------GRELKF-----------------
84 Yellowstone_lake_phycodnavirus_1                 17.0%  ------------------------------EDCVFQIGMTTRKFG---SD-EP----MERKCL-----------------
   consensus/100%                                          .................................h..hs..h..................p..h.................
   consensus/90%                                                                          tt.h..hsh.h...t   .....    ..p.hh
   consensus/80%                                                                          ts.lh.huhhh.p.s   .. ..    ..p.hh
   consensus/70%                                                                          ss.lhpluhsh.phs   t. ..    hpchll

                                                         481          .         5         .         .         .         .         :       . 56
 1 Acanthamoeba_castellanii_mamavirus              100.0%  --------------------TL--KKCA---------PIE---------------------------------GVN-VEC
 2 Acanthamoeba_polyphaga_mimivirus                 98.6%  --------------------TL--KKCA---------PIE---------------------------------GVN-VEC
 3 Acanthamoeba_polyphaga_moumouvirus               56.0%  --------------------TL--KKCA---------RIK---------------------------------GTN-VEC
 4 Amsacta_moorei_entomopoxvirus                    10.1%  --------------------TL--INYEIIKNYVGEKKDKF--------------------------------IYTEVNK
 5 Anomala_cuprea_entomopoxvirus                     9.7%  --------------------IL--INTNIILDYVNYVDKF---------------------------------DDKFIND
 6 ASFV_BAV71                                        9.7%  --------------------TM--APCK---------KSS---------------------------------EWT-TIL
 7 ASFV_E75                                          9.7%  --------------------TM--APCK---------KSS---------------------------------EWT-TIL
 8 Brazilian_marseillevirus                         12.6%  --------------------SL--GNCL---------RIR---------------------------------GAK-VLN
 9 Cafeteria_roenbergensis_virus_BV-PW1             20.9%  --------------------TL--NSCD---------NIE---------------------------------GAT-VES
10 Cannes_8_virus                                   13.3%  --------------------SL--GDCL---------RIP---------------------------------GVL-VRN
11 Catovirus_CTV1                                   24.3%  --------------------TL--GSCD---------PIE---------------------------------GAI-VVS
12 Cedratvirus_A11                                  12.1%  --------------------VL--GELEIE-------------------------------------------GVT-VIN
13 Choristoneura_biennis_entomopoxvirus             10.5%  --------------------TL--INYEIIKNYKGEQKDRF--------------------------------IYTEIDE
14 Common_midwife_toad_ranavirus                    13.2%  --------------------SL--EADDYPEAEVLAER-----------------------------------GYA-VQQ
15 Cowpox_virus                                     10.5%  --------------------TL--INEEMLTEQEIQEAVDR--------------------------------GCLRIQS
16 Diadromus_pulchellus_ascovirus_4a                12.7%  --------------------TL--GPCSVV-------------------------------------------ENATLTV
17 Ectromelia_virus                                 10.5%  --------------------TL--INEEMLTEQEMQEAVDR--------------------------------GCLRIQS
18 Emiliania_huxleyi_virus_145                      14.6%  --------------------CL--NECA---------PVE---------------------------------GSY-VES
19 Emiliania_huxleyi_virus_86                       14.6%  --------------------CL--NECA---------PVE---------------------------------GSY-VES
20 European_catfish_virus                           13.1%  --------------------SL--EADDYPEAEVLAER-----------------------------------GYA-VQQ
21 Faustovirus_D3                                    8.8%  --------------------SI--GLTKHNSI-----SFD---------------------------------GYT--IV
22 Faustovirus_E12                                   8.9%  --------------------SI--GLSKHNTR-----SFD---------------------------------GYS--IV
23 Faustovirus_E24                                   8.9%  --------------------SI--GLSKHNTR-----SFD---------------------------------GYS--IV
24 Faustovirus_E9                                    9.0%  --------------------SI--GLTKHNSI-----SLD---------------------------------GYT--IV
25 Faustovirus_Liban                                 9.0%  --------------------SI--GLSKHNTR-----SFD---------------------------------GYS--IV
26 Feldmannia_species_virus                         13.4%  --------------------CV--GDVE---------SVE---------------------------------GTP-ILC
27 Frog_virus_3                                     13.2%  --------------------SL--EADDYPEAEVLAER-----------------------------------GYA-VQQ
28 Heliothis_virescens_ascovirus_3e                 11.3%  --------------------LI--APDGCGEAMKRSMGAG---------------------------------DELTVHV
29 Hokovirus_HKV1                                   20.3%  --------------------TL--GSCD---------PIE---------------------------------GVE-VES
30 Infectious_spleen_and_kidney_necrosis_virus      12.7%  --------------------SL--PGRDY--------------------------------------------DDS-VYQ
31 Insectomime_virus                                12.3%  --------------------SL--GNCL---------RLP---------------------------------NAK-VLN
32 Invertebrate_iridescent_virus_22                 13.4%  --------------------SL--GDPQK--------NIV---------------------------------GAN-ILK
33 Invertebrate_iridescent_virus_3                  14.2%  --------------------SL--GNPTE--------QIV---------------------------------GAT-ILR
34 Kaumoebavirus                                    10.2%  --------------------TR--YDCEIPDLAETEEILREIGKEGDGTYDDISAEETSRALRAIEKKGEKSREFDYIVK
35 Klosneuvirus_KNV1                                15.8%  --------------------TL--GSCD---------KIE---------------------------------GAD-VES
36 Kurlavirus_BKC-1                                 12.6%  --------------------SL--GNCL---------KLP---------------------------------DAK-VIN
37 Lausannevirus                                    12.4%  --------------------SL--GGCL---------KLP---------------------------------NAK-VIN
38 Lumpy_skin_disease_virus_NI-2490                 10.5%  --------------------TL--INEDILSNKDKKDATLK--------------------------------GYYKAKS
39 Lymphocystis_disease_virus                       13.5%  --------------------SL--PGKDYD-------KIN---------------------------------PDIKVLQ
40 Marseillevirus_marseillevirus_T19                13.3%  --------------------SL--GDCL---------RIP---------------------------------GVL-VRN
41 Megavirus_chiliensis                             60.1%  --------------------TL--KKCA---------RIK---------------------------------GTN-VEC
42 Megavirus_courdo7                                60.4%  --------------------TL--KKCA---------RIK---------------------------------GTN-VEC
43 Megavirus_ursino                                 60.1%  --------------------TL--KKCA---------RIK---------------------------------GTN-VEC
44 Melbournevirus                                   13.3%  --------------------SL--GDCL---------RIP---------------------------------GVL-VRN
45 Micromonas_pusilla_virus_SP1                     16.3%  --------------------CY--KNTS---------------------------------------------GPD-VRS
46 Mollivirus_sibericum                              9.9%  --------------------NV--SGPNADRRINRK-RAD---------------------------------EIRALVR
47 Molluscum_contagiosum_virus_subtype_1             9.1%  --------------------SL--LNSDLLSED----SLR---------------------------------GCRRIAT
48 Monkeypox_virus                                  10.5%  --------------------TL--INEEMLTEQEIQEAVDR--------------------------------GCLRIQS
49 Myxoma_virus                                     10.7%  --------------------TL--LNEDMLTEDEIHEAVSR--------------------------------GYHRAES
50 Noumeavirus                                      12.5%  --------------------SL--GNCL---------KLP---------------------------------DAK-VIN
51 Orf_virus                                        10.2%  --------------------TL--TNSDMLSDADLEEAARR--------------------------------EIPVCLD
52 Organic_Lake_phycodnavirus_1                     18.2%  --------------------CL--NDSHNI-------------------------------------------GDNVLEC
53 Orpheovirus_LCC2                                 12.8%  --------------------IF--GECQ---------EVK---------------------------------DGE-IIR
54 Ostreococcus_lucimarinus_virus_1                 16.7%  --------------------CY--KQTDP--------NLE---------------------------------GSN-ILS
55 Ostreococcus_tauri_virus_OtV5                    16.7%  --------------------CY--KKTDP--------NLE---------------------------------GST-IRS
56 Pacmanvirus_A23                                   9.1%  --------------------VD--RACNARP------------------------------------------GINLVIE
57 Pandoravirus_dulcis                               9.2%  DPKQRERWGVREMDLTS---AG--RPEATATTAGHMVDAEEEPSQ----------------------------REVYVMQ
58 Pandoravirus_inopinatum                           8.8%  DPRQRERWGVL---------EM--DRDGTDPMATEAPDAQQQQKQRQARSH----------------------HEVYVMQ
59 Pandoravirus_salinus                             10.5%  DPRQRAHWSVFEMNRAAPADTM--ATETADAQQQQQQQEQQPRSH----------------------------REVYVMQ
60 Paramecium_bursaria_Chlorella_virus_CVK2         15.4%  --------------------CY--KQTA---------PVE---------------------------------GVE-IIS
61 Paramecium_bursaria_chlorella_virus_MT325        15.1%  --------------------CF--KDTG---------KVE---------------------------------GVE-IVS
62 Paramecium_bursaria_Chlorella_virus_NYs1         14.2%  --------------------CY--KQTA---------SVE---------------------------------GVE-IIS
63 Phaeocystis_globosa_virus                        16.9%  --------------------VK--GGCEVPEKYKSW-VLE---------------------------------NNVKIIE
64 Phaeocystis_globosa_virus_14T                    16.8%  --------------------VK--GGCEVPEKYKSW-VLE---------------------------------NNVKIIE
65 Pithovirus_massiliensis                          11.6%  --------------------VM--GKVG---------EIP---------------------------------GTT-IIE
66 Pithovirus_sibericum                             11.8%  --------------------VM--GKVG---------EIP---------------------------------GTT-IIE
67 Port-miou_virus                                  12.4%  --------------------SL--GGCL---------KLP---------------------------------NAK-VIN
68 Powai_lake_megavirus                             60.0%  --------------------TL--KKCA---------RIK---------------------------------GTI-VEC
69 Scale_drop_disease_virus                         12.8%  --------------------SL--TGNDYV-------KTD---------------------------------DYR-IQQ
70 Short-finned_eel_ranavirus                       13.1%  --------------------SL--GADDYPEAEVLAER-----------------------------------GYA-VQQ
71 Singapore_grouper_iridovirus                     13.3%  --------------------SL--TGQDYE-------DLE--PEE----------------------------AIE-VRQ
72 Skunkpox_virus                                   11.0%  --------------------TL--INEEMLTEDEIQEAVDR--------------------------------GCLRIAS
73 Spodoptera_frugiperda_ascovirus_1a               10.9%  --------------------ITAPGECERDLVDALRKDKDI--------------------------------DNLNVHV
74 Testudo_hermanni_ranavirus                       13.2%  --------------------SL--EADDYPEAEVLAER-----------------------------------GYA-VQQ
75 Tiger_frog_virus                                 13.1%  --------------------SL--EADDYPEAEVLAER-----------------------------------GYA-VQQ
76 Tokyovirus_A1                                    12.9%  --------------------SL--GNSL---------KIP---------------------------------GVV-VKN
77 Trichoplusia_ni_ascovirus_2c                     10.4%  --------------------TL--GPVSNEDKI----RFD---------------------------------EDTDIKV
78 Tunisvirus_fontaine2                             12.2%  --------------------SL--GNCL---------RLP---------------------------------NAK-VLN
```

```
79 Vaccinia_virus                                 10.6%   --------------------TL--INEEMLTEQEIQEAVDR--------------------------------GCLRIQS
80 Variola_virus                                  10.3%   --------------------TL--INEEMLTEQEIQEAVDR--------------------------------GCLRIQS
81 Volepox_virus                                  10.8%   --------------------TL--INEEMLTEEEIQEAVNR--------------------------------GCLRISS
82 Wiseana_iridescent_virus                       14.4%   --------------------SL--GKPKE--------SIV---------------------------------GAT-ILS
83 Yaba_monkey_tumor_virus                        10.8%   --------------------TL--INRDMLSDFDINEAKKL--------------------------------GYYEIDS
84 Yellowstone_lake_phycodnavirus_1               17.0%   --------------------CF--KNTA---------------------------------------------GPD-AES
   consensus/100%                                         ................................................................................
   consensus/90%                                                              sh  .................                                t...h..
   consensus/80%                                                              sh  htpt.............                                s...l.p
   consensus/70%                                                              ol  tsst.........th.                                 sh..l.p

                                                       561         .         .         .         6         .         .         .         64
 1 Acanthamoeba_castellanii_mamavirus            100.0%   YKK----------EKGLLRGFAKKIA-ELRPDFKTGYNNFGFDDKYIYDR---ILRIDKREGKKQGVNINALKNKFMDEI
 2 Acanthamoeba_polyphaga_mimivirus               98.6%   YKK----------EKGLLRGFAKKIA-ELRPDFKTGYNNFGFDDKYIYDR---ILRIDKREGKKQGVNINALKNKFMDEI
 3 Acanthamoeba_polyphaga_moumouvirus             56.0%   YKT----------EKGLLRGWARLIS-RIRPDFKAGYNNFGFDDKYIIDR---INRIDQDEAKRQGITVDELENKFLDEI
 4 Amsacta_moorei_entomopoxvirus                  10.1%   LLNTNKVYITIYCTEKYMLHFVLYTL-RQDFDYVLTYNGHNFDFTYIQDR----RKIN------------------KLKG
 5 Anomala_cuprea_entomopoxvirus                   9.7%   IMNNNNVYNYIYATEQVILEFMIYLF-LQDFDYILSYNGHNFDIPYIQGR---LNYYR------------------MGNL
 6 ASFV_BAV71                                      9.7%   CSS----------EKNLLLSFAEQFS-RWAPDICTGFNDSRYDWPFIVEK---SMQHG------------------ILEE
 7 ASFV_E75                                        9.7%   CSS----------EKNLLLSFAEQFS-RWAPDICTGFNDSRYDWPFIVEK---SMQHG------------------ILEE
 8 Brazilian_marseillevirus                       12.6%   ART----------EKELLLLFFRRVS-DIDPDLVIGYNIIKFDWNYLMTR---AKRHG------------------IFQR
 9 Cafeteria_roenbergensis_virus_BV-PW1           20.9%   FDV----------ESDLLLAWLEEIK-NSDADILTGYNIFYFDEKYIMDR---AKYLG------------------IEID
10 Cannes_8_virus                                 13.3%   YPT----------EKRLLAAMIERIS-EIDPDIIIGYNSLKFDWNYLMIR---IKKCG------------------LFQR
11 Catovirus_CTV1                                 24.3%   CKT----------EKELLIEWSKMIR-KTNPDIITGYNIFGFDYKYLKNR---AKKLG------------------IEYQ
12 Cedratvirus_A11                                12.1%   VNS----------EEELCDALVDLII-KTNPDIITGYNIFGFDYPYLYSR------LG------------------LYNQ
13 Choristoneura_biennis_entomopoxvirus           10.5%   LLTKDKVYITIYCTEKYMLHFILYTL-RKDFDYILTYNGHSFDFTYIQGR---RKFYN------------------LNE-
14 Common_midwife_toad_ranavirus                  13.2%   YPN----------ERSLISGLCDMLS-SVKPQVVTGYNVLGFDIDYLLKR---CVRLG------------------MEEE
15 Cowpox_virus                                   10.5%   LMEMDYERELVLCSEIVLLQIAKQLL-ELTFDYIVTFNGHNFDLRYITNR---LELLT------------------GEKI
16 Diadromus_pulchellus_ascovirus_4a              12.7%   VDS----------ERDLIVAFAKLIQ-KEQPNIITGWNIFTFDFKFMINR---AERNM------------------CMHE
17 Ectromelia_virus                               10.5%   LMEMDYERELVLCSEIVLLQIAKQLL-ELTFDYIVTFNGHNFDLRYITNR---LELLT------------------GEKI
18 Emiliania_huxleyi_virus_145                    14.6%   YNT----------EAELYNAFRDLITVHSDADVCTGYNIFGFDNEYITTR---AKMCK------------------ASRF
19 Emiliania_huxleyi_virus_86                     14.6%   YNT----------EAELYNAFRDLITVHSDADVCTGYNIFGFDNEYITTR---AKMCK------------------ASRF
20 European_catfish_virus                         13.1%   YPD----------ERSLISSLCDMLS-SAKPQVITGYNVLGFDIDYLLKR---CVRLG------------------MEEE
21 Faustovirus_D3                                  8.8%   CGS----------ELGVVDTFLAILA-RYLPDYDNGFNSQRFDWRVMFHK---FRRYA------------------KFNY
22 Faustovirus_E12                                 8.9%   CGS----------ELGVVKTFLEILA-RYLPDYGNGFNSQRFDWRVMFHK---FKRYN------------------MFNY
23 Faustovirus_E24                                 8.9%   CGS----------ELGVVKTFLEILA-RYLPDYGNGFNSQRFDWRVMFHK---FKRYN------------------MFNY
24 Faustovirus_E9                                  9.0%   CGS----------EIGVVDTFLAILA-RYLPDYGNGFNSQRFDWRVMFHK---FRRYG------------------KFNY
25 Faustovirus_Liban                               9.0%   CGS----------ELGVVKTFLEILA-RYLPDYGNGFNSQRFDWRVMFHK---FKRYN------------------LFSY
26 Feldmannia_species_virus                       13.4%   VQN----------ELQLLKKFREIVV-ERQVCILVGYNSYQFDGQFLYKR---AVDTY------------------NYQD
27 Frog_virus_3                                   13.2%   YPN----------ERSLISGLCDMLS-SVKPQVVTGYNVLGFDIDYLLKR---CVRLG------------------MEEE
28 Heliothis_virescens_ascovirus_3e               11.3%   CPN----------EVSLLLEFNKVCR-SIGAVARMGWNVNRFDCVVLMAR---ANRLN------------------CSTT
29 Hokovirus_HKV1                                 20.3%   YEK----------EEEVLLAWRDLIL-RENPDIITGYNIFGFDYKYLFVR---AKQLG------------------IERE
30 Infectious_spleen_and_kidney_necrosis_virus    12.7%   YAT----------EGELLHAFIAYIR-EHEVVAVCGYNIMGFDIPYIIKR---CARTS------------------MLGT
31 Insectomime_virus                              12.3%   ART----------ERELLLTFFRRFS-EIDVDLVIGYNIIKFDWNYLMTR---AKICG------------------VFQR
32 Invertebrate_iridescent_virus_22               13.4%   YST----------EQKLLMGFKDLVN-EKNPNVVIGYNIFNFDIPYLMDR---ANHKS------------------IYPV
33 Invertebrate_iridescent_virus_3                14.2%   YST----------EKKLLMGFRDLVN-EKNPNVITGYNIFNFDIPYLMDR---TVYKS------------------IFVE
34 Kaumoebavirus                                  10.2%   CQN----------EKDLILTFAKVIN-RFKPEYIVDFNGHNYDWPFIIWR--VINRYR------------------IEEQ
35 Klosneuvirus_KNV1                              15.8%   YEN----------EVAVLLAWTKMMN-RMNPDVVTGYNIFGFDYWYMHKR---AKKFG------------------CEKS
36 Kurlavirus_BKC-1                               12.6%   ART----------EKELLSIFFRRLS-EIDADVYIGYNILKFDWNYLMVR---AKKCG------------------LFQR
37 Lausannevirus                                  12.4%   ART----------EKELLLIFFRRLS-EIDADVYIGYNILKFDWNYLMVR---AKKCG------------------LFQR
38 Lumpy_skin_disease_virus_NI-2490               10.5%   VSDVDYTKEIIICSEITMLKVAKKLL-ELTFDYVVTFNGNNFDLRYISNR---LELLT------------------SGKI
39 Lymphocystis_disease_virus                     13.5%   YNT----------EKNLLEGLIKVIT-NLNPDALVGYNILKFDIDYILKR---CRRWL------------------VVES
40 Marseillevirus_marseillevirus_T19              13.3%   YPT----------EKRLLAAMIERIS-EIDPDIIIGYNSLKFDWNYLMIR---IKKCG------------------LFQR
41 Megavirus_chiliensis                           60.1%   YKT----------EKGLIRGWAKKIS-EIRPDFKGGYNNFGFDDKYIFDR---INRIDREEADRQGISVNELENRFLDEI
42 Megavirus_courdo7                              60.4%   YKT----------EKGLIRGWAKKIS-EIRPDFKGGYNNFGFDDKYIFDR---INRIDREEADRQGISVNELENRFLDEI
43 Megavirus_ursino                               60.1%   YKT----------EKGLIRGWAKKIS-EIRPDFKGGYNNFGFDDKYIFDR---INRIDREEADRQGISVNELENRFLDEI
44 Melbournevirus                                 13.3%   YPT----------EKRLLAAMIERIS-EIDPDIIIGYNSLKFDWNYLMIR---IKKCG------------------LFQR
45 Micromonas_pusilla_virus_SP1                   16.3%   FDT----------EREMLEAFQKYIQ-EKDVDIITGWNIFGFDLEYIYKR---AFICG------------------CNSN
46 Mollivirus_sibericum                            9.9%   AKSPDIDLSLYNDELVMLEDWSDFIAIHVQPSMLEGYYVTKFDMRWVAKR---ATLLVLNKARAMRQKRPRRNYLDDDED
47 Molluscum_contagiosum_virus_subtype_1           9.1%   PDEFALEPGVTFCSEVVLLQLTKRLL-ERHFDFIVTFNGNNFDIRYVSNR---LQLLT------------------QSSV
48 Monkeypox_virus                                10.5%   LMEMDYERELVLCSEIVLLRIAKQLL-ELTFDYVVTFNGHNFDLRYITNR---LELLT------------------GEKI
49 Myxoma_virus                                   10.7%   CNDVDYKKEFILCTEITMLRIAKKLL-ELSFDFIVTFNGNNFDLRYVSNR---LELLT------------------DERI
50 Noumeavirus                                    12.5%   ARS----------EKELLSIFFRRLS-EIDADVYIGYNILKFDWNYLMVR---AKKCG------------------LFQR
51 Orf_virus                                      10.2%   PADVKFDAEVTLCPEVTLLRVAKRLL-EMPLDFVVTFNGHNFDLRYLDSR---LSLLT------------------GEHI
52 Organic_Lake_phycodnavirus_1                   18.2%   YST----------EKDVLCAWSRLIM-KEDPDIIIGYNIFGFDYPFMYER---ANQLN------------------CMDE
53 Orpheovirus_LCC2                               12.8%   VNT----------EIELINKFCEVIK-QLDPDIISGYNILGFDWGYLDAR---LKRVG------------------IKEW
54 Ostreococcus_lucimarinus_virus_1               16.7%   FST----------EKEMLEAFHKYLH-KKDVDIITGWNIFGFDMEYIYKR---AQING------------------CHYS
55 Ostreococcus_tauri_virus_OtV5                  16.7%   YET----------EREMLEAFQKYLH-TKDVDIITGWNIFGFDMEYIYKR---AQVNR------------------CHYE
56 Pacmanvirus_A23                                 9.1%   CES----------ELNVLNAHMEAMS-RMAPDITGAFNGGNFDWPLYKEK---LRRAG------------------LLTT
57 Pandoravirus_dulcis                             9.2%   CAT----------ELEAIEGWRDLIVLDVQPSVVEGYNTDAFDFGWLGVRAERCARYG------------------VRSR
58 Pandoravirus_inopinatum                         8.8%   CAT----------ELEAIEGWRDLIVLDVQPSVVEGYNTDAFDFGWLGVRAERCARYG------------------VRSR
59 Pandoravirus_salinus                           10.5%   CAT----------ELEAIEGWRDLIVLDVQPSVVEGYNTDAFDFDWLGTRAQRCARYG------------------VRSR
60 Paramecium_bursaria_Chlorella_virus_CVK2       15.4%   CLE----------ESDVMNTWMKILQ-DEKTDVSIGYNTWQYDLRYVHGR---TQMCV------------------DDMT
61 Paramecium_bursaria_chlorella_virus_MT325      15.1%   CSE----------EQDMINAWMTIVS-EEKTDVLIGYNVFQYDWKYVSGR---AQMLV------------------DDAS
62 Paramecium_bursaria_Chlorella_virus_NYs1       14.2%   CAE----------EADVMNTWMTILQ-DEKTDVSIGYNLWQYDLRYIHGR---SMMCV------------------DDIT
63 Phaeocystis_globosa_virus                      16.9%   KTT----------EKGVLLEFTKIMT-LENPHIVTGYNINGFDFDFMFKR---SKEIG------------------CVED
64 Phaeocystis_globosa_virus_14T                  16.8%   KTT----------EKGVLLEFTKIMT-LENPHIVTGYNINGFDFDFMFKR---SKEIG------------------CVED
65 Pithovirus_massiliensis                        11.6%   VET----------EEQLCFAFANLIR-ETDPDIVTGFNIFGFDYPYLYFR---LGMYL--------------------QD
66 Pithovirus_sibericum                           11.8%   VET----------EEQLCFAFANLIR-ETDPDIVTGFNIFGFDYPYLYFR------LG------------------MYLQ
67 Port-miou_virus                                12.4%   ART----------EKELLLIFFRRLS-EIDADVYIGYNILKFDWNYLMVR---AKKCG------------------LFQR
68 Powai_lake_megavirus                           60.0%   YKT----------EKGLIRGWAKKIS-EIRPDFKGGYNNFGFDDKYIFDR---INRIDREEAERQGISVNELENRFLDEI
69 Scale_drop_disease_virus                       12.8%   YES----------ECDLIQAFIDHIT-ANKINVLCGYNVMGFDIPYLIKR---CTRLS------------------ILGM
70 Short-finned_eel_ranavirus                     13.1%   YPD----------ERSLISGLCDMLS-SVKPQVVTGYNVLGFDIDYLLKR---CVRLG------------------MEEE
71 Singapore_grouper_iridovirus                   13.3%   YES----------EKALLLGLCKMLT-ALKPQMVCGYNVLGFDIDYMLQR---CRRLG------------------IEEA
72 Skunkpox_virus                                 11.0%   VMEMDYERELVLCSEIVLLQIAKQLL-ELTFDFVVTFNGHNFDLRYITNR---LELLT------------------GDKI
73 Spodoptera_frugiperda_ascovirus_1a             10.9%   CRD----------EVSLLLKFNQVTR-SIGAVARMGWNVNRFDCVVMMAR---ATQLN------------------CVNT
74 Testudo_hermanni_ranavirus                     13.2%   YPN----------ERSLISGLCDMLS-SVKPQVVTGYNVLGFDIDYLLKR---CVRLG------------------MEEE
75 Tiger_frog_virus                               13.1%   YPN----------ERSLISGLCDMLS-SVKPQVVTGYNVLGFDIDYLLKR---CVRLG------------------MEEE
76 Tokyovirus_A1                                  12.9%   YPT----------EKRLLAAMIERIA-EIDPDIIVGYNSLKFDWNYLMVR---IKKCG------------------LFQR
77 Trichoplusia_ni_ascovirus_2c                   10.4%   YQT----------EERLLLGFSELCR-KLSVVARMGWNSSRFDCKVLLAR---ANRLN------------------CINS
78 Tunisvirus_fontaine2                           12.2%   ART----------ERELLLTFFRRFS-EIDVDLVIGYNIIKFDWNYLMTR---AKICG------------------VFQR
79 Vaccinia_virus                                 10.6%   LMEMDYERELVLCSEIVLLRIAKQLL-ELTFDYVVTFNGHNFDLRYITNR---LELLT------------------GEKI
80 Variola_virus                                  10.3%   LMEMDYERELVLCSEIVLLQIAKQLL-ELTFDYIVTFNGHNFDLRYITNR---LELLT------------------GEKI
81 Volepox_virus                                  10.8%   VMEMDYERELVLCSEIVLLQIAKQLL-ELTFDFVVTFNGHNFDLRYITNR---LELLT------------------GDKI
82 Wiseana_iridescent_virus                       14.4%   YPT----------EQKLLMGFKNLVN-EKNPNVIIGYNIFNFDIPYLMDR---ANHKS------------------IYPV
83 Yaba_monkey_tumor_virus                        10.8%   VLDMDYSKEFIFCSEIILLKISKKLL-ELSFDFIVTFNGHNFDLRYISNR---LELLT------------------SGKI
84 Yellowstone_lake_phycodnavirus_1               17.0%   FDT----------EKKLLQAFDKYLI-KTDPDIITGWNIFGFDLEFLQVR---AVKNG------------------LAPT
   consensus/100%                                         ................hh..h...h.......h..sa....aD..hh..+..............................
   consensus/90%                                          h.p..........p..hh.th.p.h. p.t.phh.saN..tFD..hh..R   h..ht                  ....
   consensus/80%                                          h.s          Ettll.thhchl. phpsshhhuaN..tFDh.alhtR   h.hhs                  h.p.
   consensus/70%                                          hts          Ectll.tahchlt chpsDhhhGaNhhtFDhpYlhpR   hphhs                  h.p.

                                                       641         :         .         .         .         .         7         .         72
 1 Acanthamoeba_castellanii_mamavirus            100.0%   ------LRT-IGKVNNNYLIENEGLDRIPIYTTVKDKKISSKASRFIQIRGGTYVENGNNLKYVQSPGITYF-EVKNLSS
 2 Acanthamoeba_polyphaga_mimivirus               98.6%   ------LRT-IGKVNNNYLIENEGLDRIPIYTTVKDKKISSKASRFIQIRGGTYVENGNNLKYVQSPGITYF-EVKNLSS
```

```
 3 Acanthamoeba_polyphaga_moumouvirus          56.0%  ------LCI-IGKVNNKYLMENEGLKKS----------------------------------------LSYF-EVKNLSS
 4 Amsacta_moorei_entomopoxvirus               10.1%  ------LCL-----------------------------------------------------------DNVY-STNEIKI
 5 Anomala_cuprea_entomopoxvirus                9.7%  ------VTQNIMGVNNL---------------------------------------------------LINQ-KITNLKT
 6 ASFV_BAV71                                   9.7%  ------IFNKMSLFWHQKLDTIL---------------------------------------------KCYYVKEKRVKI
 7 ASFV_E75                                     9.7%  ------IFNKMSLFWHQKLDTIL---------------------------------------------KCYYVKEKRVKI
 8 Brazilian_marseillevirus                    12.6%  ------MCELLTRIFAV---------------------------------------------------PASE-DKMSWSS
 9 Cafeteria_roenbergensis_virus_BV-PW1        20.9%  ------IKY-LSKLKTF---------------------------------------------------RCPF-KEQKLAS
10 Cannes_8_virus                              13.3%  ------LCTTLTRIFAV---------------------------------------------------PASE-DKMSWKS
11 Catovirus_CTV1                              24.3%  ------FSK-LSRVNDE---------------------------------------------------CTQF-KEKDLSS
12 Cedratvirus_A11                             12.1%  ------EWRNISRTREG---------------------------------------------------VNLL-KEINWKS
13 Choristoneura_biennis_entomopoxvirus        10.5%  ------LCLVNAHKSNE---------------------------------------------------LKIY--------
14 Common_midwife_toad_ranavirus               13.2%  ------LCL-TGMAAER---------------------------------------------------PAKE-RTISWSS
15 Cowpox_virus                                10.5%  ------IFRSPDKKEAVH--------------------------------------------------LCIY-ERNQSSH
16 Diadromus_pulchellus_ascovirus_4a           12.7%  ------FTN-FGMVDE----------------------------------------------------EGQI-TTVKWSS
17 Ectromelia_virus                            10.5%  ------IFRSPDKKEAVY--------------------------------------------------LCIY-ERNQSSH
18 Emiliania_huxleyi_virus_145                 14.6%  ------AYN--GRLITV---------------------------------------------------KTES-QAKELES
19 Emiliania_huxleyi_virus_86                  14.6%  ------AYN--GRLITV---------------------------------------------------KTES-QAKELES
20 European_catfish_virus                      13.1%  ------LCL-TGMAAER---------------------------------------------------PAKE-RTISWSS
21 Faustovirus_D3                               8.8%  INDNISLYQ-FGQKCNL---------------------------------------------------EKKFFTREQIKI
22 Faustovirus_E12                              8.9%  INEHISLYQ-FGQKCNL---------------------------------------------------ERKFFTREQIKI
23 Faustovirus_E24                              8.9%  INEHISLYQ-FGQKCNL---------------------------------------------------ERKFFTREQIKI
24 Faustovirus_E9                               9.0%  INEMISLYQ-FGAKCNL---------------------------------------------------EKKFFTREQIKI
25 Faustovirus_Liban                            9.0%  INEHISLYQ-FGQKCNL---------------------------------------------------ERKFFTREQIKI
26 Feldmannia_species_virus                    13.4%  ------FCK-IGFLRND---------------------------------------------------KASL-KTKVLES
27 Frog_virus_3                                13.2%  ------LCL-TGMAAER---------------------------------------------------PAKE-RTISWSS
28 Heliothis_virescens_ascovirus_3e            11.3%  ------LLD-LGLALDV---------------------------------------------------PGCV-STTAGRT
29 Hokovirus_HKV1                              20.3%  ------FSL-LSRLNSY---------------------------------------------------NCEY-VKKNLNS
30 Infectious_spleen_and_kidney_necrosis_virus 12.7%  ------LRR-IGFDNRR---------------------------------------------------LAIE-KT-----
31 Insectomime_virus                           12.3%  ------MCDLLTRIPSM---------------------------------------------------PATE-DKMSWSS
32 Invertebrate_iridescent_virus_22            13.4%  ------WSQ-QGFAKGK---------------------------------------------------SGIQ-REIKWSS
33 Invertebrate_iridescent_virus_3             14.2%  ------WAQ-QGFAKNR---------------------------------------------------PGIP-REIRWSS
34 Kaumoebavirus                               10.2%  ------FMKLVSCE------------------------------------------------------PVPE-DQRKWTW
35 Klosneuvirus_KNV1                           15.8%  ------FIK-FNRLNNE---------------------------------------------------ETPY-IKKDLSS
36 Kurlavirus_BKC-1                            12.6%  ------MCDLLTRIFAV---------------------------------------------------PASE-DKMSWRS
37 Lausannevirus                               12.4%  ------MCDLLTRISAV---------------------------------------------------PASE-DKMSWRS
38 Lumpy_skin_disease_virus_NI-2490            10.5%  ------IFKSPDKKEHVH--------------------------------------------------LCIY-ERNLSSH
39 Lymphocystis_disease_virus                  13.5%  ------FKS-IS-KYYK---------------------------------------------------PARE-KTISWSS
40 Marseillevirus_marseillevirus_T19           13.3%  ------LCTTLTRIFTV---------------------------------------------------PASE-DKMSWKS
41 Megavirus_chiliensis                        60.1%  ------LNI-MGKVNNVYLIENEGVNKLTDDSNTRQHEGIRKS-------------------------LTYF-EIKNLSS
42 Megavirus_courdo7                           60.4%  ------LNI-MGKVNNVYLIENEGVNKLTDDSNTRQHEGIRKS-------------------------LTYF-EIKNLSS
43 Megavirus_ursino                            60.1%  ------LNI-MGKVNNVYLIENEGVNKLTDDSNTRQHEGIRKS-------------------------LTYF-EIKNLSS
44 Melbournevirus                              13.3%  ------LCTTLTRIFTV---------------------------------------------------PASE-DKMSWKS
45 Micromonas_pusilla_virus_SP1                16.3%  ------FFK-LGKLKDQ---------------------------------------------------SCEI-VVKKLSS
46 Mollivirus_sibericum                         9.9%  ALDEASLLEATERDAYRCTRVFRMGCFFGW--------------------------------------YGRL-GSSSLSS
47 Molluscum_contagiosum_virus_subtype_1        9.1%  ------CFRLPDQREPVK--------------------------------------------------LVIY-ERSLASH
48 Monkeypox_virus                             10.5%  ------IFRSPDKKEAVH--------------------------------------------------LCIY-ERNQSSH
49 Myxoma_virus                                10.7%  ------IFKSPDRQEVVH--------------------------------------------------LCIY-ERNLSSH
50 Noumeavirus                                 12.5%  ------MCDLLTRIFAV---------------------------------------------------PASE-DKMSWRS
51 Orf_virus                                   10.2%  ------RFRLPDGTETVN--------------------------------------------------FCVY-ERTKSSH
52 Organic_Lake_phycodnavirus_1                18.2%  ------FMV-LGRNKEQ---------------------------------------------------STKL-FETSIVL
53 Orpheovirus_LCC2                            12.8%  ------PC--FGRIKNR---------------------------------------------------HPSF-TEFSWES
54 Ostreococcus_lucimarinus_virus_1            16.7%  ------FFN-LGKLKDT---------------------------------------------------ESEL-VIKKLSS
55 Ostreococcus_tauri_virus_OtV5               16.7%  ------FFN-LGKLRDT---------------------------------------------------ESEL-VIKKLSS
56 Pacmanvirus_A23                              9.1%  ------LKKKFSSLLPIINGRYADTDESVA--------------------------------------KWCF-RSEEIKI
57 Pandoravirus_dulcis                          9.2%  ------LFE-AGVLIGE---------------------------------------------------HTPM-RRKDLDS
58 Pandoravirus_inopinatum                      8.8%  ------LFE-AGVLIGE---------------------------------------------------HTPM-RRKDLDS
59 Pandoravirus_salinus                        10.5%  ------LFE-AGVLIGE---------------------------------------------------HTPM-RRKDLDS
60 Paramecium_bursaria_Chlorella_virus_CVK2    15.4%  GEDKVKLSN-LGRLLSG---------------------------------------------------GGEV-VERDLSS
61 Paramecium_bursaria_chlorella_virus_MT325   15.1%  ADDTVFVDT-LGRLLEG---------------------------------------------------GGAV-VERELAS
62 Paramecium_bursaria_Chlorella_virus_NYs1    14.2%  GEDNVRLKN-LGRLLVG---------------------------------------------------GGEV-IERDLSS
63 Phaeocystis_globosa_virus                   16.9%  ------FLK-LSKNIDE---------------------------------------------------VC---MTKDWKT
64 Phaeocystis_globosa_virus_14T               16.8%  ------FLK-LSKNIDE---------------------------------------------------VC---MTKDWKT
65 Pithovirus_massiliensis                     11.6%  ------WPA-MGRLRSK----A----------------------------------------------KTIL-SEINWKS
66 Pithovirus_sibericum                        11.8%  ------DWPAMGRLRSK---------------------------------------------------AKTILSEINWKS
67 Port-miou_virus                             12.4%  ------MCDLLTRISAV---------------------------------------------------PASE-DKMSWRS
68 Powai_lake_megavirus                        60.0%  ------LNI-MGKVNNVYLIENEGVNKLSDDSNTRQHEGIRKS-------------------------LTYF-EIKNLSS
69 Scale_drop_disease_virus                    12.8%  ------FRR-MGSDPTK---------------------------------------------------ISIE-KTVNWMS
70 Short-finned_eel_ranavirus                  13.1%  ------LCL-TGMAAER---------------------------------------------------PAKE-RTISWSS
71 Singapore_grouper_iridovirus                13.3%  ------LCS-VGMAAHR---------------------------------------------------PAKE-RVISWSS
72 Skunkpox_virus                              11.0%  ------IFRSPDKKEAVH--------------------------------------------------LCIY-ERNQSSH
73 Spodoptera_frugiperda_ascovirus_1a          10.9%  ------LLD-MGFAKDV---------------------------------------------------PGQV-STTAGRT
74 Testudo_hermanni_ranavirus                  13.2%  ------LCL-TGMAAER---------------------------------------------------PAKE-RTISWSS
75 Tiger_frog_virus                            13.1%  ------LCL-TGMAAER---------------------------------------------------PAKE-RTISWSS
76 Tokyovirus_A1                               12.9%  ------LCNSVTRIFAV---------------------------------------------------PASE-DKMSWKS
77 Trichoplusia_ni_ascovirus_2c                10.4%  ------VLD-LGMNP-----------------------------------------------------PGKI-STNSGRT
78 Tunisvirus_fontaine2                        12.2%  ------MCDLLTRIPSM---------------------------------------------------PATE-DKMSWSS
79 Vaccinia_virus                              10.6%  ------IFRSPDKKEAVH--------------------------------------------------LCIY-ERNQSSH
80 Variola_virus                               10.3%  ------IFRSPDKKEAVH--------------------------------------------------LCIY-ERNQSSH
81 Volepox_virus                               10.8%  ------IFRSPDKKEAVH--------------------------------------------------LCIY-ERNQSSH
82 Wiseana_iridescent_virus                    14.4%  ------WSQ-QGFAKEK---------------------------------------------------SGIQ-REIKWSS
83 Yaba_monkey_tumor_virus                     10.8%  ------YFRSPDKKETVH--------------------------------------------------MCIY-ERNLSSH
84 Yellowstone_lake_phycodnavirus_1            17.0%  ------W----GRFKDS---------------------------------------------------PIEL-VTKNLSS
   consensus/100%                                     ................................................................................
   consensus/90%                                      ......h....s................                                        .t.. ...t.p.
   consensus/80%                                            hh...shh.t..                                                  .s.. pp.phpp
   consensus/70%                                            hhp.hsth.sh                                                   .s.. pphphpo

                                                   721         .         .         :         .         .         .         .         8 80
 1 Acanthamoeba_castellanii_mamavirus         100.0%  SA------------LGDNELKF-I-QIPGVLSID--MMKVIQRDH-RLIGYKLDNVSANFITEKADKIIEMPHNQEDSDS
 2 Acanthamoeba_polyphaga_mimivirus            98.6%  SA------------LGDNELKF-I-QIPGVLSID--MMKVIQRDH-RLIGYKLDNVSANFITEKADKIIEMPHNQEDSDS
 3 Acanthamoeba_polyphaga_moumouvirus          56.0%  SA------------LGDNELKF-L-QVPGILSVD--MMKVIQRDH-RLIGYKLDNVSANFITQKALRCVENEKSDEDQC-
 4 Amsacta_moorei_entomopoxvirus               10.1%  SKF-----------SYNQDTTYEIDSTNGIIFLD--LYNYIKKTYPSSNSYKLSEITKERFNIF-CKISYNNNEYIIE--
 5 Anomala_cuprea_entomopoxvirus                9.7%  IQ----------------DIRHDIASNNATTYLD--IYNYVKEFY-KLPSYKLDTIAKYKFNINAKTVTDGKITT-----
 6 ASFV_BAV71                                   9.7%  SA------------EKSIISSF-L-HTPGCLPID--VRNMCMQLYPKAEKTSLKAFLENCGLDS----------------
 7 ASFV_E75                                     9.7%  SA------------EKSIISSF-L-HTPGCLPID--VRNMCMQLYPKAEKTSLKAFLENCGLDS----------------
 8 Brazilian_marseillevirus                    12.6%  SA------------YGTQKFVF-T-KIPGVSQID--MYVEFERNH-KLDKNTLDHVSELFLGEK----------------
 9 Cafeteria_roenbergensis_virus_BV-PW1        20.9%  SA------------LGENLLRY-F-DTPGLVHID--LMKDVQKTY-NLSSYKLDKVASNFIKGNVKEIKNISTKNKSP--
10 Cannes_8_virus                              13.3%  SA------------YGTQEFKF-L-KIPGVSQID--MYIEFERNE-KLDKNTLDFVAEKFLGER----------------
11 Catovirus_CTV1                              24.3%  AA------------LGKNIMTY-Y-DMTGRINID--LMKVVQRDY-KLASYKLDYVASYFIKEDIISVSCDPGKNTTK--
12 Cedratvirus_A11                             12.1%  SA------------YSRQHYYM-M-DAQGRINFD--LYTLARREM-KLLRYNLETVAQENLGRG----------------
13 Choristoneura_biennis_entomopoxvirus        10.5%  --------------SYNKDTTYEIDSNNGIIFLD--LYNYIKKIY-NYNSYKLGEIAKERFNIL-SKIIDNGDEYIIM--
14 Common_midwife_toad_ranavirus               13.2%  SA------------FGAQKYSY-L-DWEGVVAVD--LLPIIKRDY-KFDSYRLDFVAETLLGSN----------------
15 Cowpox_virus                                10.5%  KGV-----------GGMANTTFHVNNNNGTIFFD--LYSFIQKSE-KLDSYKLDSISKNAFSCMGKVLNRGVREMTFI--
16 Diadromus_pulchellus_ascovirus_4a           12.7%  KA------------FSATNVQY-V-DVEGVLCID--LIEVVRKDY-KLDSYSLNNVSKHFLKNE----------------
```

```
17 Ectromelia_virus                          10.5%  KGV-----------GGMANTTFHVNNNNGTIFFD--LYSFIQKSE-KLDSYKLDSIAKNAFGCMTKVLNRGVREMTFI--
18 Emiliania_huxleyi_virus_145               14.6%  SA------------LGQNRMFP-I-EWKGRCNFD--LFNFIKSNH-KLSLYGLGPVSQHFIGET----------------
19 Emiliania_huxleyi_virus_86                14.6%  SA------------LGQNRMFP-I-EWKGRCNFD--LFNFIKSNH-KLSLYGLGPVSQHFIGET----------------
20 European_catfish_virus                    13.1%  SA------------FGAQKYSY-L-DWEGVVAVD--LLPIIKRDY-KFDSYRLDFVAETLLGSN----------------
21 Faustovirus_D3                             8.8%  NA------------EKTHISDY-VAKIPGMLDSD--TQPLFYKLY-PKMEIKLERSLNYFLKLNDLPA------------
22 Faustovirus_E12                            8.9%  NA------------EKTHISDY-VAKFPGMLDSD--TQPLFYKLY-PKMEIKLERSLNYFLQLNDLPS------------
23 Faustovirus_E24                            8.9%  NA------------EKTHISDY-VAKFPGMLDSD--TQPLFYKLY-PKMEIKLERSLNYFLQLNDLPS------------
24 Faustovirus_E9                             9.0%  NA------------EKTHISDY-VAKIPGMLDSD--TQPLFYKLY-PKMEIKLERSLNYFLQLNDLPA------------
25 Faustovirus_Liban                          9.0%  NA------------EKTHISDY-VAKFPGMLDSD--TQPLFYKLY-PKMEIKLERSLNYFLQLNDLPA------------
26 Feldmannia_species_virus                  13.4%  SA------------LGKNELSQ-F-VIPGRVEFD--ALMTVRRNH-KLGSYKLDSVCRHFFGGK----------------
27 Frog_virus_3                              13.2%  SA------------FGAQKYSY-L-DWEGVVAVD--LLPIIKRDY-KFDSYRLDFVTETLLGSN----------------
28 Heliothis_virescens_ascovirus_3e          11.3%  FGR-----------FSPNDAIY-F-DTHGVLCLD--VMEMFKSTYTKLPKYSLQYVSQKFLGTT----------------
29 Hokovirus_HKV1                            20.3%  AG------------LGDNKLKY-Y-EMTGRINID--MLKVIQRDY-KLPSYKLDDVVQEFIKEQVKKIEEQDDTTILY--
30 Infectious_spleen_and_kidney_necrosis_virus 12.7%  AG------------VGHAKMTY-I-QWEGVLTID--LMPIIMMDH-KLDSYSLDYVANHFVKAG----------------
31 Insectomime_virus                         12.3%  SA------------YGTQKFAF-T-RIPGVSQID--MYVEFERNH-KLDKNTLDHVSELFLGEK----------------
32 Invertebrate_iridescent_virus_22          13.4%  SA------------YKNQEFKF-L-DCEGRLYID--LLPVVQRDF-KLNDYKLKTVSTHFIGET----------------
33 Invertebrate_iridescent_virus_3           14.2%  SA------------YKNQEFKY-L-DCEGRLYID--LLPVVQRDF-KLNDYKLKTVSTFFIGET----------------
34 Kaumoebavirus                             10.2%  SGMDLEKTEEIKLEAGRNTNAV-FVKAPGFIPVDARVQYMTMQEYKTKEKSSLNFYLSENGLDS----------------
35 Klosneuvirus_KNV1                         15.8%  AA------------MGQNKLKF-Y-AMQGRIQVD--LLKVVQKDY-KLGSYKLDNVAAEFIKEAVVKVEMKDGLTTIF--
36 Kurlavirus_BKC-1                          12.6%  SA------------YGTQNFKF-I-RIPGASQID--MYVEFERNH-KLDKNTLDHVSELFLGEK----------------
37 Lausannevirus                             12.4%  SA------------YGTQNFKF-I-RIPGASQID--MYVEFERNH-KLDKNTLDHVSELFLGEK----------------
38 Lumpy_skin_disease_virus_NI-2490          10.5%  KGV-----------GGVANTTYHVNNNNGTTFFD--LYTYIQKSE-KLDSYKLDSISKNAFSCSVKLKSENDNLLTFV--
39 Lymphocystis_disease_virus                13.5%  AA------------FKCQEYVF-V-DWEGIIVLD--LLPIVERDY-KLDNYKLETVANHFLNSG----------------
40 Marseillevirus_marseillevirus_T19         13.3%  SA------------YGTQEFKF-L-KIPGVSQID--MYIEFERNE-KLDKNTLDFVAEKFLGER----------------
41 Megavirus_chiliensis                      60.1%  SA------------LGDNELKF-F-QVPGIISVD--MMKVIQRDH-RLIGYKLDNVSANFITQKALRCVETGKNNDDE--
42 Megavirus_courdo7                         60.4%  SA------------LGDNELKF-F-QVPGIISVD--MMKVIQRDH-RLIGYKLDNVSANFITQKALRCVETGKNNDDE--
43 Megavirus_ursino                          60.1%  SA------------LGDNELKF-F-QVPGIISVD--MMKVIQRDH-RLIGYKLDNVSANFITQKALRCVETGKNNDDE--
44 Melbournevirus                            13.3%  SA------------YGTQEFKF-L-KIPGVSQID--MYIEFERNE-KLDKNTLDFVAEKFLGER----------------
45 Micromonas_pusilla_virus_SP1              16.3%  SA------------LGDNLLKL-F-PMSGRFVFD--MFHEVKKGY-KLDSYSLNNVSKLYIGDQ----------------
46 Mollivirus_sibericum                       9.9%  SAR-----------GDNELFTY---PTPGRPHVD--MLYQIAFNY-KLSSYALSAVSEHFFRDKDGNVPEIMT-------
47 Molluscum_contagiosum_virus_subtype_1      9.1%  RGA-----------GGMANTSFHVNNNNGTIFFD--LYTFIQKAE-RLDSYKLDSISKNVFHCRARVLDAGAGECTLA--
48 Monkeypox_virus                           10.5%  KGV-----------CGMANTTFHVNNNNGTIFFD--LYSFIQKSE-KLDSYKLDSISKNAFSCMGKVLNRGVREMTFI--
49 Myxoma_virus                              10.7%  KGV-----------GGVSNTTYHVNNNNGTTFFD--LYTYIQKSE-KLDSYKLDSISKNVFNCDCVLTEIQNDTMTFV--
50 Noumeavirus                               12.5%  SA------------YGTQNFKF-I-RIPGASQID--MYVEFERNH-KLDKNTLDHVSELFLGEK----------------
51 Orf_virus                                 10.2%  KGV-----------GGVSSTTFHINNNNGTIYFD--LYAFIQRTE-KLDSYKLDAISKNAFHCTAVVEDARPDAVRFR--
52 Organic_Lake_phycodnavirus_1              18.2%  AS-------------GPYDLKM-L-PMSGRLQID--LYTYMRKEF-NLPSYKLDYVSSYLICDKVIRYENNEEGQSRI--
53 Orpheovirus_LCC2                          12.8%  SG------------YGFNTIGI-MNNLDGRMNID--MLPIVRRDY-KLPKYDLDTVSKKFIKRG----------------
54 Ostreococcus_lucimarinus_virus_1          16.7%  SA------------LGDNLLKL-L-PMSGRFIFD--MFHEIKKGY-KLDSYKLDNVSKLYLGDQ----------------
55 Ostreococcus_tauri_virus_OtV5             16.7%  SA------------LGDNLLKL-L-PMPGRFIFD--MFHEVKKGY-KLDSYKLDNVSKLYLGDQ----------------
56 Pacmanvirus_A23                            9.1%  DA------------ETRHHLDC-VANFPGMLDTD--VLPVFLKLY-PRAEVRKSASLNFFLAKNKLES------------
57 Pandoravirus_dulcis                        9.2%  AA------------KGSNTLNF-I-PMPGRILVD--MYHIVKAEK-RLESYTLDDVCRAIFPKDESLR------------
58 Pandoravirus_inopinatum                    8.8%  AA------------KGSNTLNF-I-PMPGRILVD--MYHIVKAEK-RLESYTLDDVCRSIFPKDESLR------------
59 Pandoravirus_salinus                      10.5%  AA------------KGSNTLNF-I-PMPGRILID--MYHIVKAEK-RLESYTLDDVCRSIFAKDESLR------------
60 Paramecium_bursaria_Chlorella_virus_CVK2  15.4%  NA------------FGQNKFFL-L-DMPGVMQID--LLQWFRKNR-NLESYSLNNVSKLYLGDQ----------------
61 Paramecium_bursaria_chlorella_virus_MT325 15.1%  NA------------FGQNFFYY-L-DTPGVIQLD--LLQWMRKNR-NLESYSLNNVSKLYLGDQ----------------
62 Paramecium_bursaria_Chlorella_virus_NYs1  14.2%  NA------------FGQNKFFL-L-DMPGVMQID--LLQWFRKNR-NLESYSLNNVSKLYLGDQ----------------
63 Phaeocystis_globosa_virus                 16.9%  GEMEIAKNKIVLA-SGEYNLSF-I-NMPGRIIVD--MCVVFRREY-TLSSNKLDYVSSYFISDSVKKIDVDKENNQTR--
64 Phaeocystis_globosa_virus_14T             16.8%  GEMEIAKNKIVLA-SGEYNLSF-I-NMPGRIIVD--MCVVFRREY-TLSSNKLDYVSSYFISDSVKKIDVDKENNQTR--
65 Pithovirus_massiliensis                   11.6%  SA------------YSRQRYYM-M-QMEGRVNFD--LFTLVRREM-KLLRYNLDTVSKQYLGRG----------------
66 Pithovirus_sibericum                      11.8%  SA------------YSRQRYYM-M-QMEGRVNFD--LYTLVRREM-KLLRYNLDTVSKQYLGRG----------------
67 Port-miou_virus                           12.4%  SA------------YGTQNFKF-I-RIPGASQID--MYVEFERNH-KLDKNTLDHVSELFLGEK----------------
68 Powai_lake_megavirus                      60.0%  SA------------LGDNELKF-F-QVPGIISVD--MMKVIQRDH-RLIGYKLDNVSANFITQKALKCVETGKNHDSE--
69 Scale_drop_disease_virus                  12.8%  SA------------YRCQKYTY-I-LWEGLLTLD--LLPIIAHDH-KMDSYKLENVAQHFIKCG----------------
70 Short-finned_eel_ranavirus                13.1%  SA------------FGAQKYSY-L-DWEGVVAVD--LLPIIKRDY-KFDSYRLDFVAETLLGSN----------------
71 Singapore_grouper_iridovirus              13.3%  TA------------FGAQKYNY-L-DWEGLIPID--LLPIIRRDY-KMDSYRLDFVAETLLGAN----------------
72 Skunkpox_virus                            11.0%  KGV-----------GGMANTTFHVNNNNGTIFFD--MYSFIQKSE-KLDSYKLDSISKNAFGCMSKVLNRGVREMTFI--
73 Spodoptera_frugiperda_ascovirus_1a        10.9%  YGK-----------ISPSEPIY-F-DTHGVLCLD--VMEMFRSTYTKLPKYSLQYVSGKFLNAS----------------
74 Testudo_hermanni_ranavirus                13.2%  SA------------FGAQKYSY-L-DWEGVVAVD--LLPIIKRDY-KFDSYRLDFVAETLLDSN----------------
75 Tiger_frog_virus                          13.1%  SA------------FGAQKYSY-L-DWEGVVAVD--LLPIIKRDY-KFDSYRLDFVAETLLGSN----------------
76 Tokyovirus_A1                             12.9%  SA------------YGTQEFKF-L-KIPGISQID--MYIDFERNE-KLDKNTLDFVAEKFLGEK----------------
77 Trichoplusia_ni_ascovirus_2c              10.4%  FGP-----------IGDYEPIY-I-DTEGVVCLD--VMEMFKSTYTKLPKFSLQYVSEVFLN------------------
78 Tunisvirus_fontaine2                      12.2%  SA------------YGTQKFAF-T-RIPGVSQID--MYVEFERNH-KLDKNTLDHVSELFLGEK----------------
79 Vaccinia_virus                            10.6%  KGV-----------GGMANTTFHVNNNNGTIFFD--LYSFIQKFE-KLDSYKLDSISKNAFSCMGKVLNKGVREMTFI--
80 Variola_virus                             10.3%  KGV-----------GGMANTTFHVNNNNGTIFFD--LYSFIQKSE-KLDSYKLDSISKNAFSCMGKVLNRGVREMTFI--
81 Volepox_virus                             10.8%  KGV-----------GGMANTTFHVNNNNGTIFFD--LYSFIQKSE-KLDSYKLDSISKNAFGCMSKVLNRGIRQMTFI--
82 Wiseana_iridescent_virus                  14.4%  SA------------YKNQEFKF-L-DCEGRLYID--LLPVVQRDF-KLNDYKLKTVSTHFIGET----------------
83 Yaba_monkey_tumor_virus                   10.8%  KGA-----------CGVSNTTYHVNNNNGTIFFD--LYSFIQKSE-KLDSYKLDHISKNVFSCNTKLITVENSHCTFV--
84 Yellowstone_lake_phycodnavirus_1          17.0%  SA------------LGNNMLKM-V-PMRGRYVFD--LFQDVKREH-KLESYSLNNVSKHFLKDQ----------------
   consensus/100%                                   ............................uh...D..h...h..........tht...t..h...................
   consensus/90%                                    tu.           .t.t...h.h....Gh..hD  hh..h.p.. ph.t.pLp.h.t.hht...............
   consensus/80%                                    pu.           hs.tphta h.ph.Ghh.hD  hh..hp+ph +h.phpLc.lup.hhtpt.............
   consensus/70%                                    sA            hGppphpa l phsGhl.hD  hh.hhp+sa +LspYpL-tVup.hlspp.............

                                                 801       .         .         .         .         :         .         .         .88
 1 Acanthamoeba_castellanii_mamavirus       100.0%  EKEDEDTDDKTYDVNIYTKSTKALEKDSYIQIMVNDGYSSSPLSEGAKYKVYDIQTITEKKLNEKTNKEEIFVYQAIKTK
 2 Acanthamoeba_polyphaga_mimivirus          98.6%  EKEDEDTDDKTYDVNIYTKSTKALEKDSYIQIMVNDGYSSSPLSEGAKYKVYDIQTITEKKLNEKTNKEEIFVYQAIKTK
 3 Acanthamoeba_polyphaga_moumouvirus        56.0%  ----------ELDISIYTESTKALESDSYIQIMVDDGYSSSPLCEGAKYKIHKIETVSETQFDEQEQKNKPYTFQAMKTK
 4 Amsacta_moorei_entomopoxvirus             10.1%  ----------PLNTKANKNKISIFYDVIRTANYCFINNNPY--------------------------------KIKNKTE
 5 Anomala_cuprea_entomopoxvirus              9.7%  ----------VYPYESSNIKKEMFYKVLRTANYCFINDKAFKIYDKKN-----------------IINNESDLYNFESIK
 6 ASFV_BAV71                                 9.7%  --------------------------------------------------------------------------------
 7 ASFV_E75                                   9.7%  --------------------------------------------------------------------------------
 8 Brazilian_marseillevirus                  12.6%  --------------------------------------------------------------------------------
 9 Cafeteria_roenbergensis_virus_BV-PW1      20.9%  ----------VNKFELICDQVNDLHQDDYIHLELMESFVSEDIGDKFKI----------------------IKLDIENKI
10 Cannes_8_virus                            13.3%  --------------------------------------------------------------------------------
11 Catovirus_CTV1                            24.3%  ----------ILTKSRDGVKINQYITVNYNDGITENKHMSGKKFKIID------------FIKEKVLEKDKNKGEIYVER
12 Cedratvirus_A11                           12.1%  --------------------------------------------------------------------------------
13 Choristoneura_biennis_entomopoxvirus      10.5%  ----------PLDTADNKNKVSIFYDVIRTANYCFINNIPY--------------------------------KIKDKTK
14 Common_midwife_toad_ranavirus             13.2%  --------------------------------------------------------------------------------
15 Cowpox_virus                              10.5%  ----------GDDTTDAKGKAAAFAKVLTTGNYVTVDDDIICKVIHKD-------------------------IWENGFK
16 Diadromus_pulchellus_ascovirus_4a         12.7%  --------------------------------------------------------------------------------
17 Ectromelia_virus                          10.5%  ----------GDDTTDAKGKAAAFAKVLTTGNYVTVNEDIICKVIHKD-------------------------IWENGFK
18 Emiliania_huxleyi_virus_145               14.6%  --------------------------------------------------------------------------------
19 Emiliania_huxleyi_virus_86                14.6%  --------------------------------------------------------------------------------
20 European_catfish_virus                    13.1%  --------------------------------------------------------------------------------
21 Faustovirus_D3                             8.8%  --------------------------------------------------------------------------------
22 Faustovirus_E12                            8.9%  --------------------------------------------------------------------------------
23 Faustovirus_E24                            8.9%  --------------------------------------------------------------------------------
24 Faustovirus_E9                             9.0%  --------------------------------------------------------------------------------
25 Faustovirus_Liban                          9.0%  --------------------------------------------------------------------------------
26 Feldmannia_species_virus                  13.4%  --------------------------------------------------------------------------------
27 Frog_virus_3                              13.2%  --------------------------------------------------------------------------------
28 Heliothis_virescens_ascovirus_3e          11.3%  --------------------------------------------------------------------------------
29 Hokovirus_HKV1                            20.3%  ----------TESSYGLYPERYIKIYYNDSLSDYTCEDINKNKFMVL---------------KIDKKIKKIGNKDLDAIY
30 Infectious_spleen_and_kidney_necrosis_virus 12.7%  --------------------------------------------------------------------------------
```

```
31 Insectomime_virus                              12.3%   --------------------------------------------------------------------------------
32 Invertebrate_iridescent_virus_22               13.4%   --------------------------------------------------------------------------------
33 Invertebrate_iridescent_virus_3                14.2%   --------------------------------------------------------------------------------
34 Kaumoebavirus                                  10.2%   --------------------------------------------------------------------------------
35 Klosneuvirus_KNV1                              15.8%   ----------TKSTYGLEIGRYIKINYNDGLSDNSYKNEFKFK------------------------------VIKVTKD
36 Kurlavirus_BKC-1                               12.6%   --------------------------------------------------------------------------------
37 Lausannevirus                                  12.4%   --------------------------------------------------------------------------------
38 Lumpy_skin_disease_virus_NI-2490               10.5%   ----------GDNTTDDIGKISSFSEVLSTGNYITIVDDVFKIVDKS--------------------------VGNDKFQ
39 Lymphocystis_disease_virus                     13.5%   --------------------------------------------------------------------------------
40 Marseillevirus_marseillevirus_T19              13.3%   --------------------------------------------------------------------------------
41 Megavirus_chiliensis                           60.1%   ----------NLDISIYTESTKALEKDSYIQIMVDDGYSSSPLCEGAKYKVIDIDGITETVYDENEKKNVTYSYQAIKTK
42 Megavirus_courdo7                              60.4%   ----------NLDISIYTESTKALEKDSYIQIMVDDGYSSSPLCEGAKYKVIDIDGITETVYDENEKKNVTYSYQAIKTK
43 Megavirus_ursino                               60.1%   ----------DLDISIYTESTKALEKDSYIQIMVDDGYSSSPLCEGAKYKVIDIDGITETVYDENDKKNVTYSYQAIKTK
44 Melbournevirus                                 13.3%   --------------------------------------------------------------------------------
45 Micromonas_pusilla_virus_SP1                   16.3%   --------------------------------------------------------------------------------
46 Mollivirus_sibericum                            9.9%   --------------------------------------------------------------------------------
47 Molluscum_contagiosum_virus_subtype_1           9.1%   ----------GGPDANAEDRLRLFCEVLQTGNYVTLDTVHVCRILGKR-------------------------FFEHGFA
48 Monkeypox_virus                                10.5%   ----------GDDTTDAKGKADTFAKVLTTGNYVTVDEDIICKVIRKD-------------------------IWENGFK
49 Myxoma_virus                                   10.7%   ----------GDHTTDAKATVNVFSQVLSTGNYINIGDDIFKVFNKT--------------------------ITKDSFT
50 Noumeavirus                                    12.5%   --------------------------------------------------------------------------------
51 Orf_virus                                      10.2%   ----------GDRSTDADGNAAVFARVLSTGNYVTVDERVC-RVLHKR-------------------------VGEDGFT
52 Organic_Lake_phycodnavirus_1                   18.2%   ----------YSKNMKGLERLHYVHFEIHNHSSELYLDGKKFQI------------------------------------
53 Orpheovirus_LCC2                               12.8%   --------------------------------------------------------------------------------
54 Ostreococcus_lucimarinus_virus_1               16.7%   --------------------------------------------------------------------------------
55 Ostreococcus_tauri_virus_OtV5                  16.7%   --------------------------------------------------------------------------------
56 Pacmanvirus_A23                                 9.1%   --------------------------------------------------------------------------------
57 Pandoravirus_dulcis                             9.2%   --------------------------------------------------------------------------------
58 Pandoravirus_inopinatum                         8.8%   --------------------------------------------------------------------------------
59 Pandoravirus_salinus                           10.5%   --------------------------------------------------------------------------------
60 Paramecium_bursaria_Chlorella_virus_CVK2       15.4%   --------------------------------------------------------------------------------
61 Paramecium_bursaria_chlorella_virus_MT325      15.1%   --------------------------------------------------------------------------------
62 Paramecium_bursaria_Chlorella_virus_NYs1       14.2%   --------------------------------------------------------------------------------
63 Phaeocystis_globosa_virus                      16.9%   ----------IYSKNLTGLTVGCYVKFDEVSHSTNNYKKGQKYEILDIN------------------------------L
64 Phaeocystis_globosa_virus_14T                  16.8%   ----------IYSKNLTGLTVGCYVKFDEVSHSTNNYKKGQKYEILDI------------------------------NL
65 Pithovirus_massiliensis                        11.6%   --------------------------------------------------------------------------------
66 Pithovirus_sibericum                           11.8%   --------------------------------------------------------------------------------
67 Port-miou_virus                                12.4%   --------------------------------------------------------------------------------
68 Powai_lake_megavirus                           60.0%   ----------DLDISIYTESTKALEKDSYIQIMVDDGYSSSPLCEGAKYKVIDIDGVTETVYDENDKKNVTYSYQAIKTR
69 Scale_drop_disease_virus                       12.8%   --------------------------------------------------------------------------------
70 Short-finned_eel_ranavirus                     13.1%   --------------------------------------------------------------------------------
71 Singapore_grouper_iridovirus                   13.3%   --------------------------------------------------------------------------------
72 Skunkpox_virus                                 11.0%   ----------GDDTTDAKGKAASFAKVLTTGNYVTVDDDIICKVISKD-------------------------IWDNGFK
73 Spodoptera_frugiperda_ascovirus_1a             10.9%   --------------------------------------------------------------------------------
74 Testudo_hermanni_ranavirus                     13.2%   --------------------------------------------------------------------------------
75 Tiger_frog_virus                               13.1%   --------------------------------------------------------------------------------
76 Tokyovirus_A1                                  12.9%   --------------------------------------------------------------------------------
77 Trichoplusia_ni_ascovirus_2c                   10.4%   --------------------------------------------------------------------------------
78 Tunisvirus_fontaine2                           12.2%   --------------------------------------------------------------------------------
79 Vaccinia_virus                                 10.6%   ----------GDDTTDAKGKAAAFAKVLTTGNYVTVDEDIICKVIRKD-------------------------IWENGFK
80 Variola_virus                                  10.3%   ----------GDDTTDAKGKAAVFAKVLTTGNYVTV-DDIICKVIHKD-------------------------IWENGFK
81 Volepox_virus                                  10.8%   ----------GDDTTDIKGKAAAFAKVLTTGNYVTVDDDTICKVISKD-------------------------IWENGFK
82 Wiseana_iridescent_virus                       14.4%   --------------------------------------------------------------------------------
83 Yaba_monkey_tumor_virus                        10.8%   ----------GNSLTDVKGKSLLFSKVLSTGNYITINDDVYKILKKV--------------------------FNCDEFY
84 Yellowstone_lake_phycodnavirus_1               17.0%   --------------------------------------------------------------------------------
   consensus/100%                                         ................................................................................
   consensus/90%                                                    ......................................            ..................
   consensus/80%                                                    .....................................                            .......
   consensus/70%                                                    ...................................                              .......

                                                      881      .         9         .         .         .         .         :         . 96
 1 Acanthamoeba_castellanii_mamavirus            100.0%   ICQKDIQQLRETIKNPLLGISWTFAKDDMHH--TKINEYFEEG-------------------------------------
 2 Acanthamoeba_polyphaga_mimivirus               98.6%   ICQKDIQQLRETIKNPLLGISWTFAKDDMHH--TKINEYFEEG-------------------------------------
 3 Acanthamoeba_polyphaga_moumouvirus             56.0%   MSQKDVALLREVLQNKLLKVYWTFAKDDMHH--TLINKYFNEG-------------------------------------
 4 Amsacta_moorei_entomopoxvirus                  10.1%   IIDDIEKLYDLTSIKNSHNKKFTIYENDIPI--NDNYATVMLSKDDVDIGDKNAYVNF----------------------
 5 Anomala_cuprea_entomopoxvirus                   9.7%   KSIGKEFKIYNNDIEISNNVEITLSKDDVEIWDKNAYKNY----------------------------------------
 6 ASFV_BAV71                                      9.7%   -------------------------KVDLPY--HLMWKYYETR-------------------------------------
 7 ASFV_E75                                        9.7%   -------------------------KVDLPY--HLMWKYYETR-------------------------------------
 8 Brazilian_marseillevirus                       12.6%   -------------------------KKDVTA--KELFKIMETSMILESFQKTSVPFRERR--------------------
 9 Cafeteria_roenbergensis_virus_BV-PW1           20.9%   ITIKTKLDLIEECDFTRGKIYWSQAKDDVPP--KEIFKMQHMGS------------------------------------
10 Cannes_8_virus                                 13.3%   -------------------------KKDVSA--KELFKVMEISMALEKRGKLGFREMR----------------------
11 Catovirus_CTV1                                 24.3%   DAIIVEGCIDTDIFGKGYKIFWCQAKDDVSA--KDIFRLQKG--------------------------------------
12 Cedratvirus_A11                                12.1%   -------------------------KHDISA--VEMFSIYEKYTSNREK-------------------------------
13 Choristoneura_biennis_entomopoxvirus           10.5%   IINDKEKLYDPISIENSLYQQFKIYKNNTPI--SDETTKVMLSKDDVDIGNKNAYVNF----------------------
14 Common_midwife_toad_ranavirus                  13.2%   -------------------------KDPVTA--ADIFRAY----------------------------------------
15 Cowpox_virus                                   10.5%   VVLSC-PTLT------NDTYKLSFGKDDVDL--AQMYKDY----------------------------------------
16 Diadromus_pulchellus_ascovirus_4a              12.7%   -------------------------KDDMNF--QDIMKAIISHESGSAD-------------------------------
17 Ectromelia_virus                               10.5%   VVLSC-PTLT------NDKYKLSFGKDDVDL--AQMYKDY----------------------------------------
18 Emiliania_huxleyi_virus_145                    14.6%   -------------------------KVDLPY--QEMFDCVRPGA------------------------------------
19 Emiliania_huxleyi_virus_86                     14.6%   -------------------------KVDLPY--QEMFDCVRPGA------------------------------------
20 European_catfish_virus                         13.1%   -------------------------KDPVTA--ADIFRAY----------------------------------------
21 Faustovirus_D3                                  8.8%   -------------------------KDDMPI--SRLWRIKRYCDALDSVKSCHCGNNSDH--------------------
22 Faustovirus_E12                                 8.9%   -------------------------KDDMPI--SRLWRIKRYGDALDDIKECHCSNPLRK--------------------
23 Faustovirus_E24                                 8.9%   -------------------------KDDMPI--SRLWRIKRYGDALDDIKECHCSNPLRK--------------------
24 Faustovirus_E9                                  9.0%   -------------------------KDDMPI--SRLWRIKRYCDALDGNPTCHCKITATT--------------------
25 Faustovirus_Liban                               9.0%   -------------------------KDDMPI--SRLWRIKRYGDALNDIKECHCSNPLRK--------------------
26 Feldmannia_species_virus                       13.4%   -------------------------KDDVSY--EYILSACESK-------------------------------------
27 Frog_virus_3                                   13.2%   -------------------------KDPVTA--ADIFRAY----------------------------------------
28 Heliothis_virescens_ascovirus_3e               11.3%   -------------------------KDPVTL--KDLNELHSRLMLDDDH-------------------------------
29 Hokovirus_HKV1                                 20.3%   IKNILPYEVKEILFKKKYKLYWCQAKDDMPY--RHMFKLQEG--------------------------------------
30 Infectious_spleen_and_kidney_necrosis_virus    12.7%   -------------------------KDPIRP--RDIFHAY----------------------------------------
31 Insectomime_virus                              12.3%   -------------------------KKDVSA--KELFKIMEVSMILESFGRTSVPFRERR--------------------
32 Invertebrate_iridescent_virus_22               13.4%   -------------------------KDDLDP--KSIFRCYREGIKTQMLIIVKVGKKHFKLVGVENEDNLKQQNFFDEVT
33 Invertebrate_iridescent_virus_3                14.2%   -------------------------KDDLDP--TSIFRCYREGTRDDSPK------------------------------
34 Kaumoebavirus                                  10.2%   -------------------------KDHMPI--PWMWTICRKLEKLIKQNEQDDDPVHPDIMRR----------------
35 Klosneuvirus_KNV1                              15.8%   TLTIEGILDGEALELTKYKVSWCQAKDDVGP--HDIFRLQKG--------------------------------------
36 Kurlavirus_BKC-1                               12.6%   -------------------------KKDVSA--KELFKVMEVSMILESFQNTRVPFRERR--------------------
37 Lausannevirus                                  12.4%   -------------------------KKDVTA--KELFKVMEVSMILESFQSTRVPFRERR--------------------
38 Lumpy_skin_disease_virus_NI-2490               10.5%   LTI-LNKNNTLKKNIDNDIYTISFGKDDVSL--SDMYKNY----------------------------------------
39 Lymphocystis_disease_virus                     13.5%   -------------------------KDPITF--KDIFKAH----------------------------------------
40 Marseillevirus_marseillevirus_T19              13.3%   -------------------------KKDVSA--KELFKVMEISMALEKRGKLGFREMR----------------------
41 Megavirus_chiliensis                           60.1%   ISQKDTEQLREVLTNKLLRVYWTFAKDDMHH--TLINKYFNEG-------------------------------------
42 Megavirus_courdo7                              60.4%   ISQKDTEQLREVLTNKLLRVYWTFAKDDMHH--TLINKYFNEG-------------------------------------
43 Megavirus_ursino                               60.1%   ISQKDTEQLREVLTNKLLRVYWTFAKDDMHH--TLINKYFNEG-------------------------------------
44 Melbournevirus                                 13.3%   -------------------------KKDVSA--KELFKVMEISMALE--KRGKLGFREMR--------------------
```

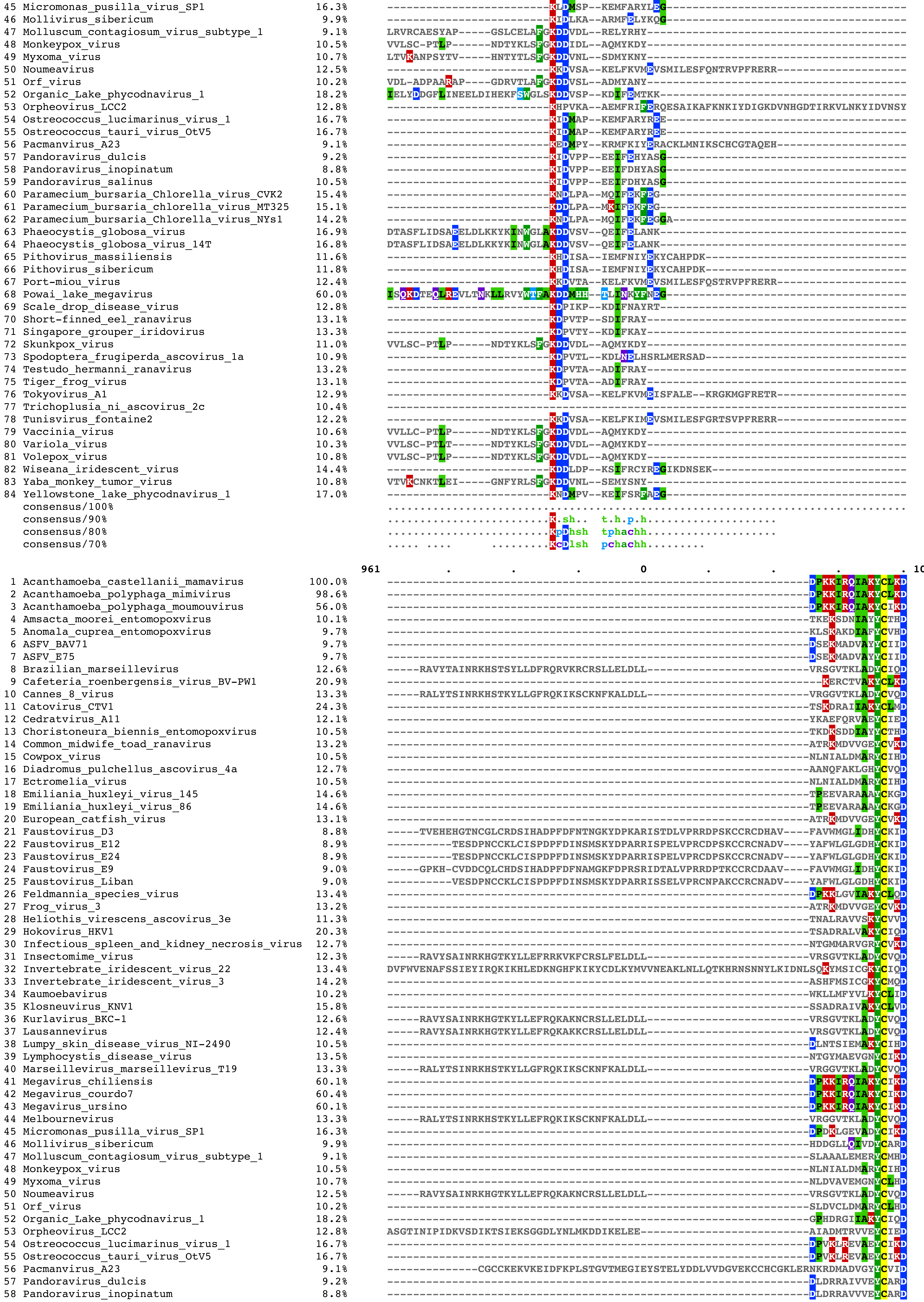

```
59 Pandoravirus_salinus 10.5% -----------------------------------------------------------------DLDRRAVVVEYCARD
60 Paramecium_bursaria_Chlorella_virus_CVK2 15.4% -----------------------------------------------------------------DSEDRAVIAAYAAKD
61 Paramecium_bursaria_chlorella_virus_MT325 15.1% -----------------------------------------------------------------GPEDRAVIAKYAAQD
62 Paramecium_bursaria_Chlorella_virus_NYs1 14.2% -------------------------------------------------------------------DDRAIIAAYAAKD
63 Phaeocystis_globosa_virus 16.9% -----------------------------------------------------------------SDIDRFTVGKYCIGD
64 Phaeocystis_globosa_virus_14T 16.8% -----------------------------------------------------------------SDIDRFTVGKYCIGD
65 Pithovirus_massiliensis 11.6% -----------------------------------------------------------------FQAEFRSVAEYCIED
66 Pithovirus_sibericum 11.8% -----------------------------------------------------------------FQAEFRSVAEYCIED
67 Port-miou_virus 12.4% -----RAVYSAINRKHGTKYLLEFRQKAKKCRSLLELDLL-------------------------VRSGVTKLADYCVQD
68 Powai_lake_megavirus 60.0% -----------------------------------------------------------------NPKKIRQIAKYCIKD
69 Scale_drop_disease_virus 12.8% -------------------------------------------------------------------KNMALVGKYCMQD
70 Short-finned_eel_ranavirus 13.1% -----------------------------------------------------------------ATRKMDVVAEYCVKD
71 Singapore_grouper_iridovirus 13.3% -----------------------------------------------------------------RTKKMAVVGKYCVKD
72 Skunkpox_virus 11.0% -----------------------------------------------------------------NLNIALDMARYCIHD
73 Spodoptera_frugiperda_ascovirus_1a 10.9% -----------------------------------------------------------------VDRLRAVVSKYCIRD
74 Testudo_hermanni_ranavirus 13.2% -----------------------------------------------------------------ATRKMDVVGEYCVKD
75 Tiger_frog_virus 13.1% -----------------------------------------------------------------ATRKMDVVGEYCVKD
76 Tokyovirus_A1 12.9% -----RRLYTSINRRHSTKYLIDFRQKIKTCKSFKALDLL-------------------------VREGVTKLADYCVQD
77 Trichoplusia_ni_ascovirus_2c 10.4% --------------------------------------------------------------------------------
78 Tunisvirus_fontaine2 12.2% -----RAVYSAINRKHGTKYLLEFRRKVKFCRSLFELDLL-------------------------VRSGVTKLADYCVQD
79 Vaccinia_virus 10.6% -----------------------------------------------------------------NLNIALDMARYCIHD
80 Variola_virus 10.3% -----------------------------------------------------------------NLNIALDMARYCIHD
81 Volepox_virus 10.8% -----------------------------------------------------------------NLNIALDMARYCIHD
82 Wiseana_iridescent_virus 14.4% -----------------------------------------------------------------ASNYMSICGKYCIQD
83 Yaba_monkey_tumor_virus 10.8% -----------------------------------------------------------------DLQTSLEMGKYCIHD
84 Yellowstone_lake_phycodnavirus_1 17.0% -----------------------------------------------------------------DPARLGEVAEYCLKD
consensus/100% ................................................................................
consensus/90%       ...................................                         ....h..hstYCh.D
consensus/80%            ..............................                         s.t.hh.hucYChtD
consensus/70%                                                                   s.ptht.lucYClpD

1041          :         .         .         .         .         1         .        . 11
1 Acanthamoeba_castellanii_mamavirus 100.0% CKLVNLLLAKLEIIVNSVGMAKVCHVPLSYLFLRGQGVKIFSLVSKK------CR-----EKN--FLIP-----------
2 Acanthamoeba_polyphaga_mimivirus 98.6% CKLVNLLLAKLEIIVNSVGMAKVCHVPLSYLFLRGQGVKIFSLVSKK------CR-----EKN--FLIP-----------
3 Acanthamoeba_polyphaga_moumouvirus 56.0% CKLVNLLLAKLEIIVNSVGMAKVCHVPLSYLFLRGQGVKIFSLVSKK------CR-----EKN--FLIP-----------
4 Amsacta_moorei_entomopoxvirus 10.1% TVLCNCIFKYDMIHDKIIAFSNEYLLPQCMAFKYKSSNNISGLLLKT------LY-----SNK--TMIY-----------
5 Anomala_cuprea_entomopoxvirus 9.7% TILCNKLFEIDMIDYKIAAFSQLYLLPQNKSLLYRNSTNSLGQILYT------LL-----KNK--MMIV-----------
6 ASFV_BAV71 9.7% AQRCQDLLVRHNVIPDRREVGILSYTSLYDCIYYAGGHKVCNMLIAY------AI-----HDE--YGRI-----------
7 ASFV_E75 9.7% AQRCQDLLVRHNVIPDRREVGILSYTSLYDCIYYAGGHKVCNMLIAY------AI-----HDE--YGRI-----------
8 Brazilian_marseillevirus 12.6% TVLPLRLLKHFDVELNMDMLAGVFCVPREYLQTRGQQVKVFSMLYRE------MQ-----SED--LIVE-----------
9 Cafeteria_roenbergensis_virus_BV-PW1 20.9% CKLVNLLINKLEIVTKNIEMANVCYVPLSYLFVRGQGIKLFSLCLKE------YR-----KHK--FLFP-----------
10 Cannes_8_virus 13.3% TVLPLRLLEKRDVVLNMDMLASVFCVPREYLQTRGQQVKVFSRLYRE------LQ-----KDN--VIVE-----------
11 Catovirus_CTV1 24.3% CALCNKLMAKLCVITNNVGMANVCHVPLSYIFYRGQGIKIFSLVAKK------CR-----EKE--HLIP-----------
12 Cedratvirus_A11 12.1% ALLVVDLIEKLNVWIWLVEFSSAMGVSVADLFIRGQQVRCFSLLQDL------AI-----KRG--IVMN-----------
13 Choristoneura_biennis_entomopoxvirus 10.5% TVLCNCIFKYDMIHDKVIAFSNEYLLPQYMSFKYKSTTNISGLLLKT------LF-----CNR--SMIV-----------
14 Common_midwife_toad_ranavirus 13.2% VQLCVDLMEKLQVWVGLTEMAKVCRVNAFTLFTQGQQIRIYSQVYCH------CE-----KNG--YVVT-----------
15 Cowpox_virus 10.5% ACLCQYLWEYYGVETKTDAGASTYVLPQSMVFEYRASTVIKGPLLKL------LL-----ETK--TILV-----------
16 Diadromus_pulchellus_ascovirus_4a 12.7% SRLVLDLFLHLQTWLSLSEMSKTTSTPIMMVHLNGQQKKFYNQVLRY------CW-----YND--ILVE-----------
17 Ectromelia_virus 10.5% ACLCQYLWEYYGVETKTDAGASTYVLPQSMVFEYRASTVIKGPLLKL------LL-----ETK--TILV-----------
18 Emiliania_huxleyi_virus_145 14.6% VLLPIRLMKALQVMPGMIEMSRVTFTTINQLVFRGQSIKVMQQITRY------SH-----QLG--HVVN-----------
19 Emiliania_huxleyi_virus_86 14.6% VLLPIRLMKALQVMPGMIEMSRVTFTTINQLVFRGQSIKVMQQITRY------SH-----QLG--HVVN-----------
20 European_catfish_virus 13.1% VQLCVDLMEKLQVWVGLTEMAKVCRVNAFTLFTQGQQIRIYSQVYCH------CE-----KNG--YVVT-----------
21 Faustovirus_D3 8.8% SHRTQQLIYKRGIYIDKCELANLSFVQLYDAFYRADGMKVRNIIGRY------AP-----KFN--IMFS-----------
22 Faustovirus_E12 8.9% SHRTQQLIFKRGIYVDKCELANLSYVQLYDAFYRADGMKVRNIIGRY------AP-----RFN--IMFS-----------
23 Faustovirus_E24 8.9% SHRTQQLIFKRGIYVDKCELANLSYVQLYDAFYRADGMKVRNIIGRY------AP-----RFN--IMFS-----------
24 Faustovirus_E9 9.0% SHRTQQLIYKRGIYIDKCELANLSFVQLYDAFYRADGMKVRNIIGRY------AP-----KFN--IMFS-----------
25 Faustovirus_Liban 9.0% SHRTQQLIFKRGIYVDKCELANLSYVQLYDAFYRADGMKVRNIIGRY------AP-----RFN--IMFS-----------
26 Feldmannia_species_virus 13.4% AWLTLRLVSSLKDVYNGLEMSKLCVVPLSYIESRGQQIKCLSLILDRVHGEFVCN-----KAS--RVLP-----------
27 Frog_virus_3 13.2% VQLCVDLMEKLQVWVGLTEMAKVCRVNAFTLFTQGQQIRIYSQVYCH------CE-----KNG--YVVT-----------
28 Heliothis_virescens_ascovirus_3e 11.3% SRLTLQLCQKCAHMTSLTEMARITNTPITMVHYQKQQRRMFHLMFSE------CA-----RKG--VAMQ-----------
29 Hokovirus_HKV1 20.3% CKLCNILMEKLQVLTNNIGMAMVCHVPLPFIFLRGQGIKIHSLVGKM------CK-----EDN--HLMRKNYKNKTKEQL
30 Infectious_spleen_and_kidney_necrosis_virus 12.7% TQLCKQLVDYLNTWVALCEMAGVCNTSIMQLFTQGQQVRVFAQIYRD------CTPMDVVDKV--YVIP-----------
31 Insectomime_virus 12.3% TVLPLRLLKHFDVELNMDMLAGVFCVPREYLQTRGQQVKVFSMLYRE------MQ-----SED--LIVE-----------
32 Invertebrate_iridescent_virus_22 13.4% SMLVSKLMDKLNVWYSLSEMAVICNVPMITLFTKGQQIKVYSNLYKF------CL-----ENK--IIPE-----------
33 Invertebrate_iridescent_virus_3 14.2% SMLVYKLFEKLNVWYGLSEMAVVCNVPMITLFTKGQQIKVYSQLYKY------CL-----AAK--IIPE-----------
34 Kaumoebavirus 10.2% SVRCHDLLIKRTVVVDKRGVAKMTYISMYDAFFRADGMKVVNAISGY------TY-----KKDPNYFTT-----------
35 Klosneuvirus_KNV1 15.8% CILCNKLMNKLQVLTNNIGMANVCHVPLSYIFLRGQGIKIFSLVAKK------CR-----ERN--HLMP-----------
36 Kurlavirus_BKC-1 12.6% TVLPLRLLKKFNVELNMDMLASVFCVPREYLQTRGQQVKVFSMLYRE------MQ-----DDD--LIVE-----------
37 Lausannevirus 12.4% TVLPLRLLKKFNVELNMDMLASVFCVPREYLQTRGQQVKVFSMLYRE------MQ-----DDD--LIVE-----------
38 Lumpy_skin_disease_virus_NI-2490 10.5% ACLCKYLWNYYGIETKIDAGAFTYILPQSMVFEYRASTLIKGPLLNL------LL-----DEK--IILS-----------
39 Lymphocystis_disease_virus 13.5% ADLCLKLTDILNLWIGLTELAKICNVDIMSLYARGQQIRVYSQLYAY------CS-----QNN--IVVG-----------
40 Marseillevirus_marseillevirus_T19 13.3% TVLPLRLLEKRDVVLNMDMLASVFCVPREYLQTRGQQVKVFSRLYRE------LQ-----KDN--VIVE-----------
41 Megavirus_chiliensis 60.1% CKLVNLLLAKLEIIVNSVGMAKVCHVPLSYLFLRGQGVKIFSLVSKK------CR-----EKN--FLIP-----------
42 Megavirus_courdo7 60.4% CKLVNLLLAKLEIIVNSVGMAKVCHVPLSYLFLRGQGVKIFSLVSKK------CR-----EKN--FLIP-----------
43 Megavirus_ursino 60.1% CKLVNLLLAKLEIIVNSVGMAKVCHVPLSYLFLRGQGVKIFSLVSKK------CR-----EKN--FLIP-----------
44 Melbournevirus 13.3% TVLPLRLLEKRDVVLNMDMLASVFCVPREYLQTRGQQVKVFSRLYRE------LQ-----KDN--VIVE-----------
45 Micromonas_pusilla_virus_SP1 16.3% TLLPHKLLKKMCILLNLLEMAKATWVPLCYLVERGQQIKVFSQLTKK------AR-----EMG--FMVP-----------
46 Mollivirus_sibericum 9.9% AVLVIMLEEKLMMMVELIEMSKVSTTTIDDILTRGQTAKGVNRLAWE------CH-----RMETHIVVN-----------
47 Molluscum_contagiosum_virus_subtype_1 9.1% ACLCKYLWSYYRVPSKIDAAAATYLLPQCLALEYRASTLIKGPLLRL------ML-----RER--VVYV-----------
48 Monkeypox_virus 10.5% ACLCQYLWEYYGVETKTDAGASTYVLPQSMVFEYRASTVIKGPLLKL------LL-----ETK--TILV-----------
49 Myxoma_virus 10.7% ACLCKYLWNYYGIETKADAGAFTYILPQSMVFEYRASTLIKGPLLKL------LL-----EKK--IIMT-----------
50 Noumeavirus 12.5% TVLPLRLLKKFNVELNMDMLASVFCVPREYLQTRGQQVKVFSMLYRE------MQ-----DDD--LIVE-----------
51 Orf_virus 10.2% ACLCLYLWSHYGVETKIAAAASTYLLPQSVVFEYRASTCIKGPLMKL------LL-----ENR--TVMV-----------
52 Organic_Lake_phycodnavirus_1 18.2% CNLVHQIFQKVDVLTTFSEMSKLCSVPIQFLVLRGQGIKLTSYISKK------CR-----EKD--TLMP-----------
53 Orpheovirus_LCC2 12.8% SELVLDLMQKLNVWISLIEMSNIVCVPVFDIFTRGQQIRVLHQVYKL------TS-----KKG--IVID-----------
54 Ostreococcus_lucimarinus_virus_1 16.7% TLLPHKLMKKLCTLLNLVEMAKATWVPANFLVERGQQIKVFSQLTKK------AR-----ELG--FMVP-----------
55 Ostreococcus_tauri_virus_OtV5 16.7% TLLPHRLMKKLCTLLNMVEMAKATWVPANFLVERGQQIKVFSQLTKK------AR-----ELG--FMVP-----------
56 Pacmanvirus_A23 9.1% CVRPQQLYVKRTIVPDKRELSTMSYVSLYDSFYRADGMKVRNVIGKN------CF-----KRG--IAFS-----------
57 Pandoravirus_dulcis 9.2% CRLPLALEEHLMTLTGVVEMARITRTPLPLMLISGQQVKTWSQIVYE------AH-----TMG--YVVN-----------
58 Pandoravirus_inopinatum 8.8% CRLPLALEEHLMTLTGVVEMARITRTPLPLMLISGQQVKTWSQIVYE------AH-----TMG--YVVN-----------
59 Pandoravirus_salinus 10.5% CRLPLALEEHLMTLTGVVEMARITRTPLPLMLISGQQVKTWSQIVYE------AH-----TMG--YVVN-----------
60 Paramecium_bursaria_Chlorella_virus_CVK2 15.4% TDLPLKLLKKMAILEDLTEMANAVKVPVDYINFRGQQIRAFSCLVGK------AR-----QMN--YAIP-----------
61 Paramecium_bursaria_chlorella_virus_MT325 15.1% TLLPLKLLSKLAIFEDITEMANAVKVPVDWIGFRGQQVRAFSCLFGK------AR-----EMN--YAIP-----------
62 Paramecium_bursaria_Chlorella_virus_NYs1 14.2% TDLPLKLLKKMAILEDITEMANAVKVPVDYINFRGQQVRAFSCLVGK------AR-----QMN--YAIP-----------
63 Phaeocystis_globosa_virus 16.9% CDNVIWLLIKVDIITDKVEMSNLCDVPLNFLLQRGQGIKLQSYVSKK------CG-----EKN--TLMP-----------
64 Phaeocystis_globosa_virus_14T 16.8% CDNVIWLLIKVDIITDKVEMSNLCDVPLNFLLQRGQGIKLQSYVSKK------CG-----EKN--TLMP-----------
65 Pithovirus_massiliensis 11.6% SLLVVDLIEKLNVWIWLVEFSSVTGVSVSDLFTRGQQMRCFSLIQHL------TA-----QKG--IVID-----------
66 Pithovirus_sibericum 11.8% SLLVVDLIEKLNVWIWLVEFSSVTGVSVSDLFTRGQQMRCFSLIQHL------TS-----QKG--IVID-----------
67 Port-miou_virus 12.4% TVLPLRLLKKFNVELNMDMLASVFCVPREYLQTRGQQVKVFSMLYRE------MQ-----DDD--LIVE-----------
68 Powai_lake_megavirus 60.0% CKLVNLLLAKLEIIVNSVGMAKVCHVPLSYLFLRGQGVKIFSLVSKK------CR-----EKN--FLIP-----------
69 Scale_drop_disease_virus 12.8% VYLCRDLLNHLNVWIALCEMAKVCNTDIMPLFTNGQQIKVFSQIYRE------CTPCKMSDKM--YVIP-----------
70 Short-finned_eel_ranavirus 13.1% VQLCVDLMEKLQVWVGLTEMAKVCRVNAFTLFTQGQQIRIYSQVYCH------CE-----KNG--YVVT-----------
71 Singapore_grouper_iridovirus 13.3% SQLCVDLMENLQVWVGLTEMAKVCKVNIFTLFTQGQQIKIFSQVYCH------CE-----KNG--YVVT-----------
72 Skunkpox_virus 11.0% ACLCQYLWEYYGVETKTDAGAATYVLPQSMVFEYRASTVIKGPLLKL------LL-----ETK--TILV-----------
```

```
73 Spodoptera_frugiperda_ascovirus_1a                 10.9%     SRLTLALCNKCAHITSLTEMARITNTPIVMVHYQKQQRRMFHLMFSE-------CM-----REN--VAMQ----------
74 Testudo_hermanni_ranavirus                         13.2%     VQLCVDLMEKLQVWVGLTEMAKVCRVNAFTLFTQGQQIRIYSQVYCH-------CE-----KNG--YVVT----------
75 Tiger_frog_virus                                   13.1%     VQLCVDLMEKLQVWVGLTEMAKVCRVNAFTLFTQGQQIRIYSQVYCH-------CE-----KNG--YVVT----------
76 Tokyovirus_A1                                      12.9%     TVLPLRLLEKRDVVLNMDMLASVFCVPREYLQTRGQQVKVFSRLYRE-------LQ-----KDD--VIVE----------
77 Trichoplusia_ni_ascovirus_2c                       10.4%     ------------------EMAIITRTPLDMVHHQKQQRRVYNLMYSE-------CL-----RQN--IAFQ----------
78 Tunisvirus_fontaine2                               12.2%     TVLPLRLLKHFDVELNMDMLAGVFCVPREYLQTRGQQVKVFSMLYRE-------MQ-----SED--LIVE----------
79 Vaccinia_virus                                     10.6%     ACLCQYLWEYYGVETKTDAGASTYVLPQSMVFEYRASTVIKGPLLKL-------LL-----ETK--TILV----------
80 Variola_virus                                      10.3%     ACLCQYLWEYYGVETKTDAGASTYVLPQSMVFEYKASTVIKGPLLKL-------LL-----ETK--TILV----------
81 Volepox_virus                                      10.8%     ACLCQYLWEYYGVETKTDAGAATYVLPQSMVFEYRASTVIKGPLLKL-------LL-----ETK--TILV----------
82 Wiseana_iridescent_virus                           14.4%     SMLVAKLMEKLNVWYGLSEMAVVCNVPMITLFTKGQQIKVYSNLYKY-------CL-----HHK--IVPE----------
83 Yaba_monkey_tumor_virus                            10.8%     ACLCKYLWDYYGVEKKTDAGASTYILPQSMVFEYRASTLIKGPLLSL-------LL-----ETK--TILA----------
84 Yellowstone_lake_phycodnavirus_1                   17.0%     TELPHKLLDKLCQIQNLVEMAKACWVPLAFLSERGQQIKVFSQMAYK-------AR-----ELN--FIIP----------
   consensus/100%                                               ...................hu....h........ttt....t.h.......................h............
   consensus/90%                                                s.hs..Lh.hhth..t...hu.h..hs...h...ttthhhhs.l.t.       h.     p.t  hhh.
   consensus/80%                                                s.Ls..Lhthhtl.hthsthuphhhlsh..lh.htpth+lhs.lhp.       h.     p.t  hhh.
   consensus/70%                                                shLs.tLhp+htlhhshsthAplhhlPh.hlhhpuQph+lhu.lhch       h.     cps  hlls

                                                           1121          .          .          :          .          .          .          .      2 12
 1 Acanthamoeba_castellanii_mamavirus                100.0%     --------------------------------------------------------------------------------
 2 Acanthamoeba_polyphaga_mimivirus                   98.6%     --------------------------------------------------------------------------------
 3 Acanthamoeba_polyphaga_moumouvirus                 56.0%     --------------------------------------------------------------------------------
 4 Amsacta_moorei_entomopoxvirus                      10.1%     --------------------------------------------------------------------------------
 5 Anomala_cuprea_entomopoxvirus                       9.7%     --------------------------------------------------------------------------------
 6 ASFV_BAV71                                          9.7%     --------------------------------------------------------------------------------
 7 ASFV_E75                                            9.7%     --------------------------------------------------------------------------------
 8 Brazilian_marseillevirus                           12.6%     --------------------------------------------------------------------------------
 9 Cafeteria_roenbergensis_virus_BV-PW1               20.9%     --------------------------------------------------------------------------------
10 Cannes_8_virus                                     13.3%     --------------------------------------------------------------------------------
11 Catovirus_CTV1                                     24.3%     ---------------------------------------------LLEKKKAMTDEEKKQYEKENTEEVQFEKFIKELNN
12 Cedratvirus_A11                                    12.1%     --------------------------------------------------------------------------------
13 Choristoneura_biennis_entomopoxvirus               10.5%     --------------------------------------------------------------------------------
14 Common_midwife_toad_ranavirus                      13.2%     ------------------------------------------------------------------DPADG-----KRVP
15 Cowpox_virus                                       10.5%     --------------------------------------------------------------------------------
16 Diadromus_pulchellus_ascovirus_4a                  12.7%     --------------------------------------------------------------------------------
17 Ectromelia_virus                                   10.5%     --------------------------------------------------------------------------------
18 Emiliania_huxleyi_virus_145                        14.6%     --------------------------------------------------------------------------------
19 Emiliania_huxleyi_virus_86                         14.6%     --------------------------------------------------------------------------------
20 European_catfish_virus                             13.1%     ------------------------------------------------------------------DPADG-----KRVP
21 Faustovirus_D3                                      8.8%     --------------------------------------------------------------------------------
22 Faustovirus_E12                                     8.9%     --------------------------------------------------------------------------------
23 Faustovirus_E24                                     8.9%     --------------------------------------------------------------------------------
24 Faustovirus_E9                                      9.0%     --------------------------------------------------------------------------------
25 Faustovirus_Liban                                   9.0%     --------------------------------------------------------------------------------
26 Feldmannia_species_virus                           13.4%     --------------------------------------------------------------------------------
27 Frog_virus_3                                       13.2%     ------------------------------------------------------------------DPADG-----KRVP
28 Heliothis_virescens_ascovirus_3e                   11.3%     -------------------------------------------------------------------DDFGRDRRLTMLD
29 Hokovirus_HKV1                                     20.3%     AREKKARDIKEKIYIEEIKTPEQIENDKKIKKKNILAMRKQKKHVKNIKQKYDEYFEILRRMKNNENVNETSLKYYNMIC
30 Infectious_spleen_and_kidney_necrosis_virus        12.7%     --------------------------------------------------------------------------------
31 Insectomime_virus                                  12.3%     --------------------------------------------------------------------------------
32 Invertebrate_iridescent_virus_22                   13.4%     --------------------------------------------------------------------------------
33 Invertebrate_iridescent_virus_3                    14.2%     --------------------------------------------------------------------------------
34 Kaumoebavirus                                      10.2%     ------------------------------------------------------------------------PYDPDYIP
35 Klosneuvirus_KNV1                                  15.8%     ------------------------------------------------------------------VIKKPYKPKEGEVP
36 Kurlavirus_BKC-1                                   12.6%     --------------------------------------------------------------------------------
37 Lausannevirus                                      12.4%     --------------------------------------------------------------------------------
38 Lumpy_skin_disease_virus_NI-2490                   10.5%     --------------------------------------------------------------------------------
39 Lymphocystis_disease_virus                         13.5%     --------------------------------------------------------------------------------
40 Marseillevirus_marseillevirus_T19                  13.3%     --------------------------------------------------------------------------------
41 Megavirus_chiliensis                               60.1%     --------------------------------------------------------------------------------
42 Megavirus_courdo7                                  60.4%     --------------------------------------------------------------------------------
43 Megavirus_ursino                                   60.1%     --------------------------------------------------------------------------------
44 Melbournevirus                                     13.3%     --------------------------------------------------------------------------------
45 Micromonas_pusilla_virus_SP1                       16.3%     --------------------------------------------------------------------------------
46 Mollivirus_sibericum                                9.9%     -------VEDATARRKEDKQRQEAEERARAYSEKPAKPKAVQMTLDQALMGSAGRKRSRDGLIQVNDKSITAETKSGRIN
47 Molluscum_contagiosum_virus_subtype_1               9.1%     --------------------------------------------------------------------------------
48 Monkeypox_virus                                    10.5%     --------------------------------------------------------------------------------
49 Myxoma_virus                                       10.7%     --------------------------------------------------------------------------------
50 Noumeavirus                                        12.5%     --------------------------------------------------------------------------------
51 Orf_virus                                          10.2%     --------------------------------------------------------------------------------
52 Organic_Lake_phycodnavirus_1                       18.2%     --------------------------------------------------------------------------------
53 Orpheovirus_LCC2                                   12.8%     --------------------------------------------------------------------------------
54 Ostreococcus_lucimarinus_virus_1                   16.7%     --------------------------------------------------------------------------------
55 Ostreococcus_tauri_virus_OtV5                      16.7%     --------------------------------------------------------------------------------
56 Pacmanvirus_A23                                     9.1%     --------------------------------------------------------------------------------
57 Pandoravirus_dulcis                                 9.2%     --------------------------APENRDADGGTRDWLRGAYGAGDHGGVGDAWLPGVIAMASIRAGGGNADGHAVP
58 Pandoravirus_inopinatum                             8.8%     ---------------------------ASEGRDRGGGTRDWLRGAYGVGDHGVGDQWLPGVIAMASIRAGGGNADGHQMP
59 Pandoravirus_salinus                               10.5%     --------------------------APEGRDRSGGGTRDWLRGAYGVGDHGVGDQWLPGVIAMASIRAGGGNADGHIMP
60 Paramecium_bursaria_Chlorella_virus_CVK2           15.4%     --------------------------------------------------------------------------------
61 Paramecium_bursaria_chlorella_virus_MT325          15.1%     --------------------------------------------------------------------------------
62 Paramecium_bursaria_Chlorella_virus_NYs1           14.2%     --------------------------------------------------------------------------------
63 Phaeocystis_globosa_virus                          16.9%     --------------------------------------------------------------------------------
64 Phaeocystis_globosa_virus_14T                      16.8%     --------------------------------------------------------------------------------
65 Pithovirus_massiliensis                            11.6%     --------------------------------------------------------------------------------
66 Pithovirus_sibericum                               11.8%     --------------------------------------------------------------------------------
67 Port-miou_virus                                    12.4%     --------------------------------------------------------------------------------
68 Powai_lake_megavirus                               60.0%     --------------------------------------------------------------------------------
69 Scale_drop_disease_virus                           12.8%     --------------------------------------------------------------------------------
70 Short-finned_eel_ranavirus                         13.1%     ------------------------------------------------------------------DPADG-----KRVP
71 Singapore_grouper_iridovirus                       13.3%     ------------------------------------------------------------------DPQDG-----KKTA
72 Skunkpox_virus                                     11.0%     --------------------------------------------------------------------------------
73 Spodoptera_frugiperda_ascovirus_1a                 10.9%     ------------------------------------------------------------------------------DD
74 Testudo_hermanni_ranavirus                         13.2%     ------------------------------------------------------------------DPADG-----KRVP
75 Tiger_frog_virus                                   13.1%     ------------------------------------------------------------------DPADG-----KRVP
76 Tokyovirus_A1                                      12.9%     --------------------------------------------------------------------------------
77 Trichoplusia_ni_ascovirus_2c                       10.4%     --------------------------------------------------------------------------------
78 Tunisvirus_fontaine2                               12.2%     --------------------------------------------------------------------------------
79 Vaccinia_virus                                     10.6%     --------------------------------------------------------------------------------
80 Variola_virus                                      10.3%     --------------------------------------------------------------------------------
81 Volepox_virus                                      10.8%     --------------------------------------------------------------------------------
82 Wiseana_iridescent_virus                           14.4%     --------------------------------------------------------------------------------
83 Yaba_monkey_tumor_virus                            10.8%     --------------------------------------------------------------------------------
84 Yellowstone_lake_phycodnavirus_1                   17.0%     --------------------------------------------------------------------------------
   consensus/100%                                               ................................................................................
   consensus/90%                                                                                                                  ..... ........
```

```
   consensus/80%                                                                                                      ..
   consensus/70%

                                                              1201          .         .         .         :         .         .         12
 1 Acanthamoeba_castellanii_mamavirus                 100.0%  ---VLRRKS----KD-------------NEGDEDETYEGATVITPKPNV-YL----------------------SPIGVL
 2 Acanthamoeba_polyphaga_mimivirus                    98.6%  ---VLRRKS----KD-------------NEGDEDETYEGATVITPKPNV-YL----------------------SPIGVL
 3 Acanthamoeba_polyphaga_moumouvirus                  56.0%  ---VLRKNK----KD-------------NNGDEDETYEGATVITPKPNV-YL----------------------SPIGVL
 4 Amsacta_moorei_entomopoxvirus                       10.1%  -------------PG-------------NVEFEK--FEGGYVIEPKQKY-ID----------------------SLTAVF
 5 Anomala_cuprea_entomopoxvirus                        9.7%  -------------TG-------------HVDYDK--YEGAMVYDPKKKY-VS----------------------NPTLIF
 6 ASFV_BAV71                                           9.7%  -----ACST----IA-------------RGKREHGKYPGAFVIDPVKGL-EQD---------------------KPTTGL
 7 ASFV_E75                                             9.7%  -----ACST----IA-------------RGKREHGKYPGAFVIDPVKGL-EQD---------------------KPTTGL
 8 Brazilian_marseillevirus                            12.6%  -----FLGY----DK-------------EVSNVK--YKGATVFDAKTGF-W-----------------------EDVLVW
 9 Cafeteria_roenbergensis_virus_BV-PW1                20.9%  ------VVK----KP-------------DVDLGS--YEGAIVFDPIPKVSY-----------------------QAHATK
10 Cannes_8_virus                                      13.3%  -----FRGY----DR-------------EASNVK--FKGATVFDAKIGL-W-----------------------EDILVW
11 Catovirus_CTV1                                      24.3%  KGLEDHNDE----TD-------------DDENDS--YEGATVFPPQKGV-HF----------------------DPVPVL
12 Cedratvirus_A11                                     12.1%  ---------K---EV-------------MADTGK--YQGAYVSEPTPGL-F-----------------------EHVACL
13 Choristoneura_biennis_entomopoxvirus                10.5%  -------------SG-------------NLEFEK--FEGGYVLEPKQKY-ID----------------------SITAVF
14 Common_midwife_toad_ranavirus                       13.2%  WG---QDPP----VS-------------DESDED--YIGAHVVEPSPGI-Y-----------------------DNVVPL
15 Cowpox_virus                                        10.5%  ---------R---SE-------------TKQKFP--YEGGKVFAPKQKM-FS----------------------NNVLIF
16 Diadromus_pulchellus_ascovirus_4a                   12.7%  ------SNA----YQ-------------SKATDR--YTGAYVFDPVPGL-Y-----------------------EYVVPL
17 Ectromelia_virus                                    10.5%  ---------R---SE-------------TKQKFP--YEGGKVFAPKQKM-FS----------------------NNVLIF
18 Emiliania_huxleyi_virus_145                         14.6%  --------PV---PR-------------PINTSG--YEGAIVIDAKSGF-YT----------------------DPVATL
19 Emiliania_huxleyi_virus_86                          14.6%  --------PV---PR-------------PINTSG--YEGAIVIDAKSGF-YT----------------------DPVATL
20 European_catfish_virus                              13.1%  WG---QDPP----VS-------------DEPDED--YIGAHVVEPSPGI-Y-----------------------DNVVPL
21 Faustovirus_D3                                       8.8%  ----NARKN----IE-------------QKMKQH--YPGAWVFPPKYGY-YKH---------------------RPITGE
22 Faustovirus_E12                                      8.9%  ----NARKN----IE-------------QKMKQH--YPGAWVFPPKYGY-YKN---------------------RPITGE
23 Faustovirus_E24                                      8.9%  ----NARKN----IE-------------QKMKQH--YPGAWVFPPKYGY-YKN---------------------RPITGE
24 Faustovirus_E9                                       9.0%  ----NARKN----IE-------------QKMKQH--YPGAWVFPPKYGY-YKH---------------------RPITGE
25 Faustovirus_Liban                                    9.0%  ----NARKN----IE-------------QKMKQH--YPGAWVFPPKYGY-YKN---------------------RPITGE
26 Feldmannia_species_virus                            13.4%  -------------------------------GGVK--FQGATVIDATKGF-HNK---------------------DPVVCL
27 Frog_virus_3                                        13.2%  WG---QDPP----VS-------------DEPDED--YIGAHVVEPSPGI-Y-----------------------DNVVPL
28 Heliothis_virescens_ascovirus_3e                    11.3%  EDSTDGSSN----VD-------------NKRQLN--YSGAYVKDPEPGL-Y-----------------------NMVGSL
29 Hokovirus_HKV1                                      20.3%  DITKKDLER----EEQKLNEFQEYKDELEEKNLNEGYEGATVLDPITGL-YY----------------------DPITVL
30 Infectious_spleen_and_kidney_necrosis_virus         12.7%  ------DGG----CD-------------SDVVSPSSYTGAYVYEPVPGV-Y-----------------------KNVIPM
31 Insectomime_virus                                   12.3%  -----FLGY----DK-------------EVSNVK--YKGATVFDAKIGF-W-----------------------EDVLVW
32 Invertebrate_iridescent_virus_22                    13.4%  -----KDGY----TV--------------SENER--YVGAYVFVPKPGL-Y-----------------------INVVPF
33 Invertebrate_iridescent_virus_3                     14.2%  ----KDGY-----IV--------------AENER--YVGAHVFTPKPGL-Y-----------------------ENVIPL
34 Kaumoebavirus                                       10.2%  KDLILINHR----RR-------------DTGGAKVKFQGAFVLQPKKGI-YTD---------------------RPCGGL
35 Klosneuvirus_KNV1                                   15.8%  KFPQKKMEE----DE-------------EEEDEG--YEGATVFNPDTGVHY-----------------------EPITVL
36 Kurlavirus_BKC-1                                    12.6%  -----FLGY----DK-------------EVSNVK--YKGATVFDAKVGL-W-----------------------EDVLVW
37 Lausannevirus                                       12.4%  -----FLGY----DK-------------EVSNVK--YKGATVFDAKVGL-W-----------------------EDVLVW
38 Lumpy_skin_disease_virus_NI-2490                    10.5%  ---------R---SE-------------KKNKYH--YEGGKVFAPKQKM-FI----------------------NNVLIF
39 Lymphocystis_disease_virus                          13.5%  -------------RL-------------KAGDEQ--YVGAYVVDPVPGL-Y-----------------------ENVVPL
40 Marseillevirus_marseillevirus_T19                   13.3%  -----FRGY----DR-------------EASNVK--FKGATVFDAKIGL-W-----------------------EDILVW
41 Megavirus_chiliensis                                60.1%  ---VLRRNK----KD-------------NDGDDDNTYEGATVITPKPNV-YL----------------------SPIGVL
42 Megavirus_courdo7                                   60.4%  ---VLRRNK----KD-------------NDGDDDNTYEGATVITPKPNV-YL----------------------SPIGVL
43 Megavirus_ursino                                    60.1%  ---VLRRNK----KD-------------NDGDDDNTYEGATVITPKPNV-YL----------------------SPIGVL
44 Melbournevirus                                      13.3%  -----FRGY----DR-------------EASNVK--FKGATVFDAKIGL-W-----------------------EDILVW
45 Micromonas_pusilla_virus_SP1                        16.3%  ------TIR----YG-------------AIPEEP--YEGATVLDAQKGA-YY----------------------TPITAL
46 Mollivirus_sibericum                                 9.9%  QGIKDRGGG----GGEKEEKEEEEEEPTKSKKKQKRYGGGYVLDPIRGF-YD----------------------SFIPTE
47 Molluscum_contagiosum_virus_subtype_1                9.1%  ---------R---TQ-------------EPARYT--YGGGKVFLPRQKL-FD----------------------TNVMVF
48 Monkeypox_virus                                     10.5%  ---------R---SE-------------TKQKFP--YEGGKVFAPKQKM-FS----------------------NNVLIF
49 Myxoma_virus                                        10.7%  ---------R---SE-------------KKNKYP--YEGGKVFAPKQKM-FI----------------------NNVLIF
50 Noumeavirus                                         12.5%  -----FLGY----DK-------------EVSNVK--YKGATVFDAKVGL-W-----------------------EDVLVW
51 Orf_virus                                           10.2%  ---------R---AD-------------TKSKFF--YEGGRVMAPKQKM-HN----------------------KHVLIF
52 Organic_Lake_phycodnavirus_1                        18.2%  ------LIS----KG-------------NDVD-A--YEGAIVLEPKCGL-YLN---------------------NPVACV
53 Orpheovirus_LCC2                                    12.8%  -------------RR-------------DINKIP--YKGAYVVDPIPGL-Y-----------------------KNVICL
54 Ostreococcus_lucimarinus_virus_1                    16.7%  ------TIR----YG-------------AIPEEP--YEGATVLEAQKGA-YY----------------------TPITAL
55 Ostreococcus_tauri_virus_OtV5                       16.7%  ------TIR----YG-------------AIPEEP--YEGATVLEAQKGA-YY----------------------TPITAL
56 Pacmanvirus_A23                                      9.1%  ----NARSD----KE-------------MTDKDH--YPGAWVFPPNRGL-HSDGWIDVEFITPEGKKIKKRVRCRPITGL
57 Pandoravirus_dulcis                                  9.2%  TAGGKVTGV----AA-------------DAEG----YVGATVLQPRAGY-YD----------------------VPIVTL
58 Pandoravirus_inopinatum                              8.8%  QDGGKVAGG----PG-------------AADAEG--YTGATVLQPRAGY-YD----------------------VPIVTL
59 Pandoravirus_salinus                                10.5%  QD--GGKVA----GA-------------ADAEEG--YAGATVLQPRAGY-YD----------------------VPIVTL
60 Paramecium_bursaria_Chlorella_virus_CVK2            15.4%  -------DD----KA-------------WATEGK--YEGATVLDAKKGA-YF----------------------TPIAAL
61 Paramecium_bursaria_chlorella_virus_MT325           15.1%  -------DD----KA-------------WAAEGK--FEGATVLEPKKGA-YF----------------------TPIAAL
62 Paramecium_bursaria_Chlorella_virus_NYs1            14.2%  -------------DD-------------KMWTVDGKYEGATVLDAKKGA-YF----------------------TPIAAL
63 Phaeocystis_globosa_virus                           16.9%  --------IV---EK-------------NLNDGG--YEGAHVFNPKTGL-YLE---------------------DPVACV
64 Phaeocystis_globosa_virus_14T                       16.8%  -------------IV-------------EKNLNDGGYEGAHVFNPKTGL-YLE---------------------DPVACV
65 Pithovirus_massiliensis                             11.6%  ----------------------------TITQTRDKYSGAYVSDPIPGL-Y-----------------------DYIACI
66 Pithovirus_sibericum                                11.8%  -------------TI-------------TQSRDK--YSGAYVSDPIPGL-Y-----------------------DYIACI
67 Port-miou_virus                                     12.4%  -----FLGY----DK-------------EVSNVK--YKGATVFDAKVGL-W-----------------------EDVLVW
68 Powai_lake_megavirus                                60.0%  ---VLRRNK----KD-------------NDGDDDNTYEGATVITPKPNV-YL----------------------SPIGVL
69 Scale_drop_disease_virus                            12.8%  -----SHLE----PS-------------AEEEFS--YTGAFVYEPTPGI-Y-----------------------NNVIPM
70 Short-finned_eel_ranavirus                          13.1%  WG---QDPP----MS-------------DEPDED--YIGAHVVEPSPGI-Y-----------------------DNVVPL
71 Singapore_grouper_iridovirus                        13.3%  WAAVLEEP------A-------------DQDSSD--YVGAHVVEPIPGL-Y-----------------------ENVVPL
72 Skunkpox_virus                                      11.0%  ---------R---SE-------------TKQKFP--YEGGKVFAPKQKM-FS----------------------NNVLIF
73 Spodoptera_frugiperda_ascovirus_1a                  10.9%  FGLANAAPFETVAAT-------------QKKQLS--YSGAYVKDPEPGL-Y-----------------------SMVGSL
74 Testudo_hermanni_ranavirus                          13.2%  WG---QDPP----VS-------------DESDED--YIGAHVVEPSPGI-Y-----------------------DNVVPL
75 Tiger_frog_virus                                    13.1%  WG---QDPP----VS-------------DEPDED--YIGAHVVEPSPGI-Y-----------------------DNVVPL
76 Tokyovirus_A1                                       12.9%  -----FRGY----EK-------------EESNVK--FKGATVFDAKIGL-W-----------------------EDILVW
77 Trichoplusia_ni_ascovirus_2c                        10.4%  DNMFNSKKV----VT-------------NDDVVG--YSGAYVKDPVPGI-H-----------------------RRVGSL
78 Tunisvirus_fontaine2                                12.2%  -----FLGY----DK-------------EVSNVK--YKGATVFDAKIGF-W-----------------------EDVLVW
79 Vaccinia_virus                                      10.6%  ---------R---SE-------------TKQKFP--YEGGKVFAPKQKM-FS----------------------NNVLIF
80 Variola_virus                                       10.3%  ---------R---SE-------------TKQKFP--YEGGKVFAPKQKM-FS----------------------NNVLIF
81 Volepox_virus                                       10.8%  ---------R---SE-------------TKQKFP--YEGGKVFAPKQKM-FS----------------------NNVLIF
82 Wiseana_iridescent_virus                            14.4%  -----KDGY----TI--------------SENER--YVGAYVFPPIPGL-Y-----------------------ENVVAL
83 Yaba_monkey_tumor_virus                             10.8%  ---------R---TE-------------KNNKFP--YEGGKVFTPKQKM-FV----------------------NNVLVF
84 Yellowstone_lake_phycodnavirus_1                    17.0%  -----TFER----GP-------------ALDDDK--YQGATVLDAQTGA-YY----------------------SPITAL
   consensus/100%                                             ............................a.GuhV..s..th.......................h.
   consensus/90%                                              .........    ..             ...t....a.Guhh.s..th a..                       p.lh.h
   consensus/80%                                              .. ......    .t             t.tp.t  Y.GAhVhtsp.sh a.                       pslhsh
   consensus/70%                                                  ....     .t             pttp.p  YpGAhVhsPp.Gh a.                       pslhsh

                                                              1281          .         3         .         .         .         :         .         13
 1 Acanthamoeba_castellanii_mamavirus                 100.0%  DYSSLYPNSMRERNLSQECYV-----DDSKY-------DNLPGYI-------YH---DVEIILKDKKGKILRNIDGTPQ
 2 Acanthamoeba_polyphaga_mimivirus                    98.6%  DYSSLYPNSMRERNLSQECYV-----DDSKY-------DNLPGYI-------YH---DVEIILKDKKGKILRNIDGTPQ
 3 Acanthamoeba_polyphaga_moumouvirus                  56.0%  DYSSLYPNSMRERNLSQECYV-----NDPKY-------DNLPGYI-------YH---DITIILKDKKGKIL--------
 4 Amsacta_moorei_entomopoxvirus                       10.1%  DFNSEYPSIIIEANLSPEKVV-KVIKLFDDEEAANKVEKYLKDNYK-------YP---DYCYIKIIKD-----------
 5 Anomala_cuprea_entomopoxvirus                        9.7%  DFKSLYPTAIIEGNLSPEKIE--KVIISDDEIANTYIDTYLADTYK-------YP---KYLYIKIKSNINN---------
 6 ASFV_BAV71                                           9.7%  DFASLYPSLIMAYNFSPEKFV-----ASRDEAKS--LMAKGESLH-------YV---SFHFNNRLVEG-----------
 7 ASFV_E75                                             9.7%  DFASLYPSLIMAYNFSPEKFV-----ASRDEAKS-LMAKG-ESLH-------YV---SFHFNNRLVEG-----------
 8 Brazilian_marseillevirus                            12.6%  DFESLYPSEIISRNIDYTSFC-----TNDESVPD----EECNIVE-------WD---QHEFCKCPLDSKAG--------
 9 Cafeteria_roenbergensis_virus_BV-PW1                20.9%  DFASLYPSAIIQKNMSHETKV-----DNDEF-------DNIPGIE-------YF---NAKFKDD---------------
10 Cannes_8_virus                                      13.3%  DFESLYPSEIISNNIDYTSFC-----TNDETVPD----SDCNVIE-------WD---QHEFCGCPLDSKAG--------
```

```
11 Catovirus_CTV1                                  24.3%   DYASLYPRSMILKNLSHEYLV------MDDEKY------GNLPGYR-------YH---LTTYMVSKIVEDVD--------
12 Cedratvirus_A11                                 12.1%   DFNSLYPNIMRAYNMCYTTLI---RPEQQDHLESLGLTLDDVHIIE-------WD---EEDEKTKEKK------------
13 Choristoneura_biennis_entomopoxvirus            10.5%   DFNSEYPSNIIEANLSPEKVE-KVIKLQDDEYAVDIVENYLKEKYP-------YP---DYCYMLIKKD------------
14 Common_midwife_toad_ranavirus                   13.2%   DFSSLYPSIMIAKNVCYSTRV-------DPGT-------PGSETFE-------WE---DH--LNCVHDPRKV--------
15 Cowpox_virus                                    10.5%   DYNSLYPNVCIFGNLSPETLV--GVVVSTNRLEEEINNQLLLQKYP-------PP---RYITVHCEPRLPNL--------
16 Diadromus_pulchellus_ascovirus_4a               12.7%   DFASLYPSIMIAYNLDYSTIV------MPDSNVPE----EHTVKME-------WE---DH--CGCEHDPLVV--------
17 Ectromelia_virus                                10.5%   DYNSLYPNVCIFGNLSPETLV--GVVVSTNRLEEEINNQLLLQKYP-------PP---RYITVHCEPRLPNL--------
18 Emiliania_huxleyi_virus_145                     14.6%   DFASLYPSIMLAHNLCYSTFV-------EKGT-------PRVDGVE-------YE---THK-------------------
19 Emiliania_huxleyi_virus_86                      14.6%   DFASLYPSIMLAHNLCYSTFV-------EKGT-------PRVDGVE-------YE---THK-------------------
20 European_catfish_virus                          13.1%   DFSSLYPSIMIAKNVCYSTRV-------DPGT-------PGSETFE-------WE---DH--LNCVHDPRKV--------
21 Faustovirus_D3                                   8.8%   DFSSLYPSIIMCYNFSPDRVV------TNPAKAEA-LIAQGYKLHP-------VG---PIKIEQGEKKGQAG--------
22 Faustovirus_E12                                  8.9%   DFSSLYPSIMMCYNYSPDRVV------TNPAKAEE-LKALGYKLHP-------VG---PIRYEQGEKKGQAG--------
23 Faustovirus_E24                                  8.9%   DFSSLYPSIMMCYNYSPDRVV------TNPAKAEE-LKALGYKLHP-------VG---PIRYEQGEKKGQAG--------
24 Faustovirus_E9                                   9.0%   DFSSLYPSIIMCYNFSPDRVV------TNPAKAEA-LIAQGYKLHP-------VG---PITVERGEKKGQAG--------
25 Faustovirus_Liban                                9.0%   DFSSLYPSIMMCYNYSPDRVV------TNPAKAEE-LKALGYKLHP-------VG---PIRYEQGEKKGQAG--------
26 Feldmannia_species_virus                        13.4%   DFASLYPSIIRWKNLCYTTYL------DSDEF-------ANIPGVH-------YE---RFE-------------------
27 Frog_virus_3                                    13.2%   DFSSLYPSIMIAKNVCYSTRV-------DPGT-------PGSETFT-------WE---DH--LNCVHDPRKV--------
28 Heliothis_virescens_ascovirus_3e                11.3%   DVNSMYPTLMIAYNLCYSTVI-------DDDAHSD-YTDENFEIVE-------WE---DH--VGCEHDPNIK--------
29 Hokovirus_HKV1                                  20.3%   DYASLYPSSMIQKNMSHECVV------RDPQY-------DNLPDYK-------YT---DVKYYHPD--------------
30 Infectious_spleen_and_kidney_necrosis_virus     12.7%   DFQSLYPSIIISKNICYSTLV-----------------DQGGEEYA-------WQ---EH--EGCEHDPQYA--------
31 Insectomime_virus                               12.3%   DFESLYPSEIISRNIDYTSFC------TNDESVPD----EECNIVE-------WD---QHEFCKCPLDSKAG--------
32 Invertebrate_iridescent_virus_22                13.4%   DFSALYPSLIIAYNIDYSSCA-------FDPNIPD----ELCHVME-------WE---DH--ISCKHDPKVI--------
33 Invertebrate_iridescent_virus_3                 14.2%   DFSSLYPSLMIAYNIDYSTCA-------FDASIPD----QLCHIME-------WE---DH--IGCAHDPKVV--------
34 Kaumoebavirus                                   10.2%   DFGSLYPNIIRSSALSPENII------TDLEVAR-----EICGALN------------DNLIKKRKAKPDAT--------
35 Klosneuvirus_KNV1                               15.8%   DYNSLYPNSMRMKGLSQELLV------KNPEY-------DNLPGYY-------YN---SATYTNKD--------------
36 Kurlavirus_BKC-1                                12.6%   DFESLYPSEIISRNVDYTSFC------TNDESVPD----EDCNIVE-------WD---QHEFCECPLDTKAG--------
37 Lausannevirus                                   12.4%   DFESLYPSEIISRNVDYTSFC------TNDESVPD----EDCNIVE-------WD---QHEFCECPLDTKAG--------
38 Lumpy_skin_disease_virus_NI-2490                10.5%   DYNSLYPNVCLFGNLSPETLV--SVFVANNRLEAEINKQEIEKKYP-------PP---RYISIHCEPRCDDL--------
39 Lymphocystis_disease_virus                      13.5%   DFSSLYPSIIIAKNICYSTFS------LRPT--------EYTEAFE-------WE---DH--VNCIHDPKIA--------
40 Marseillevirus_marseillevirus_T19               13.3%   DFESLYPSEIISNNIDYTSFC------TNDETVPD----SDCNVIE-------WD---QHEFCGCPLDSKAG--------
41 Megavirus_chiliensis                            60.1%   DYSSLYPNSMRERNLSPECYI------NDNKY-------DNLPGYI-------YH---DVTIILKDKKGKIIRNLDGSPK
42 Megavirus_courdo7                               60.4%   DYSSLYPNSMRERNLSPECYI------NDNKY-------DNLPGYI-------YH---DVTIILKDKKGKIIRNLDGSPK
43 Megavirus_ursino                                60.1%   DYSSLYPNSMRERNLSPECYI------NDNKY-------DNLPGYI-------YH---DVTIILKDKKGKIIRNLDGSPK
44 Melbournevirus                                  13.3%   DFESLYPSEIISNNIDYTSFC------TNDETVPD----SDCNVIE-------WD---QHEFCGCPLDSKAG--------
45 Micromonas_pusilla_virus_SP1                    16.3%   DFEALYPSIMMAHNLCYSTYV------MDEKNY------GNVPGVT-------YE---TF--------------------
46 Mollivirus_sibericum                             9.9%   DFNSLYPSIIQRSNYCYSTYVVQERYRRLLRAACTYDITPPMPGAG-------ED---EVTYLSNMPREVWE--------
47 Molluscum_contagiosum_virus_subtype_1            9.1%   DYNSLYPNVCIFGNLSPETLV--GVVANAHRLDAEINMQELRRRFP-------EP---AFLHVLCEARARDG--------
48 Monkeypox_virus                                 10.5%   DYNSLYPNVCIFGNLSPETLV--GVVVSTNRLEEEINNQLLLQKYP-------PP---RYITVHCEPRLPNL--------
49 Myxoma_virus                                    10.7%   DYNSLYPNVCLFGNLSPETLV--CVFVANNKLEAEINKQEIQKMYP-------GP---RYISVQCEPRSEDL--------
50 Noumeavirus                                     12.5%   DFESLYPSEIISRNVDYTSFC------TNDESVPD----EDCNIVE-------WD---QHEFCECPLDTKAG--------
51 Orf_virus                                       10.2%   DYNSLYPNVCIYANLSPETLV--GVVVSDNRLDAEVAAVDVRRMFP-------AP---RYIAVPCEPRSPEL--------
52 Organic_Lake_phycodnavirus_1                    18.2%   DYSSLYPSSIISENISHDSKVWTKEFDLNDHIKKDEKGREKMTGIKDEHSNFIYDNLPEYEYVDIQYDTFEY--------
53 Orpheovirus_LCC2                                12.8%   DFASLYPSIMRAYNICYTTLV-----PMENTDVPD----EMCHIFE-------WE---EEIDESKIKKASGT--------
54 Ostreococcus_lucimarinus_virus_1                16.7%   DFEALYPSIMMAHNLCYSSYV------MDEKKY------GNVPGIT-------YE---TF--------------------
55 Ostreococcus_tauri_virus_OtV5                   16.7%   DFEALYPSIMMAHNLCYSSYV------MDEKRY------GSVPGIT-------YE---TF--------------------
56 Pacmanvirus_A23                                  9.1%   DFASLYPSLMMTYNLSPDTVV------YTKEEAEK-LRNEGYSLYH-------IE---PFEFERGEKKGNSG--------
57 Pandoravirus_dulcis                              9.2%   DYQSLYPSIMEANNLCPSTRV------TSRTVHAQ--LARHCREVR-------EP---VSEVLRADGAGQDD--------
58 Pandoravirus_inopinatum                          8.8%   DYQSLYPSIMEANNLCPSTRV------TSPHIHKE--LARHCREVR-------EP---ASEVLRADGAGQDA--------
59 Pandoravirus_salinus                            10.5%   DYQSLYPSIMEANNLCPSTRV------TAEPIHRE--LARHCREVR-------EA---TSEVLRADGAGQDA--------
60 Paramecium_bursaria_Chlorella_virus_CVK2        15.4%   DFASLYPSIIRAHNMSPETLV------MEKRF-------ENVPGVE-------YY---EI--------------------
61 Paramecium_bursaria_chlorella_virus_MT325       15.1%   DFASLYPSIIRAHNMSPETLV------MDARY-------KNLPGVE-------YY---EI--------------------
62 Paramecium_bursaria_Chlorella_virus_NYs1        14.2%   DFASLYPSIIRAHNMSPETLV------MDKRF-------ENLPGIE-------YY---EI--------------------
63 Phaeocystis_globosa_virus                       16.9%   DYSSLYPSSMISENLSHDSKVWTKEYDLSDNLIHSTGEKDADENFI-------YDNLPNYTYVDVKYDTYEY--------
64 Phaeocystis_globosa_virus_14T                   16.8%   DYSSLYPSSMISENLSHDSKVWTKEYDLSDNLIHSTGEKDADENFI-------YDNLPNYTYVDVKYDTYEY--------
65 Pithovirus_massiliensis                         11.6%   DFNSLYPSIMIAYNMCYTTLI----HPSDEKEVLKFLTPDDYWVVE-------WD----------EIDEETK--------
66 Pithovirus_sibericum                            11.8%   DFNSLYPSIMIAYNMCYTTLI----HPSDEKEVLKVLTPDDYWVVE-------WD----------ETDEETK--------
67 Port-miou_virus                                 12.4%   DFESLYPSEIISRNVDYTSFC------TNDESVPD----EDCNIVE-------WD---QHEFCECPLDTKAG--------
68 Powai_lake_megavirus                            60.0%   DYSSLYPNSMRERNLSPECYI------NDNKY-------DNLPGYI-------YH---DVTIILKDKKGKIIRNLDGSPK
69 Scale_drop_disease_virus                        12.8%   DFSSLYPSIIISKNICYSTYV-------DTGSIED--VPDNVEVYK-------WQ---EH--NGCEHDTVYL--------
70 Short-finned_eel_ranavirus                      13.1%   DFSSLYPSIMIAKNVCYSTRV-------DPGT-------PGSETFE-------WE---DH--LNCVHDPRKV--------
71 Singapore_grouper_iridovirus                    13.3%   DFSSLYPSIMIAKNICYSTQA----------DLND----PTAEIFE-------WE---DH--LNCPHDHRKQ--------
72 Skunkpox_virus                                  11.0%   DYNSLYPNVCIFGNLSPETLV--GVVVSTNRLEEEINNQLLLQKYP-------SP---RYITVRCEPRLPNL--------
73 Spodoptera_frugiperda_ascovirus_1a              10.9%   DVNNMYPTLMIAYNLCYSTVV--------DEHSPSAFTDDHFEYIR-------WE---DH--VGCEHDPVQV--------
74 Testudo_hermanni_ranavirus                      13.2%   DFSSLYPSIMIAKNVCYSTRV-------DPGT-------PGSETFE-------WE---DH--LNCVHDPRKV--------
75 Tiger_frog_virus                                13.1%   DFSSLYPSIMIAKNVCYSTRV-------DPGT-------PGSETFE-------WE---DH--LNCVHDPRKV--------
76 Tokyovirus_A1                                   12.9%   DFESLYPSEIISNNIDYTSFC------TDDKSVPD----SDCNVIE-------WD---QHEFCGCPLDSKAG--------
77 Trichoplusia_ni_ascovirus_2c                    10.4%   DVNSMYPTLIIAYNLCYTTVI------NYDDETP--YKDEDFEVIE-------WE---DH--IGCEHNPIVV--------
78 Tunisvirus_fontaine2                            12.2%   DFESLYPSEIISRNIDYTSFC------TNDESVPD----EECNIVE-------WD---QHEFCKCPLDSKAG--------
79 Vaccinia_virus                                  10.6%   DYNSLYPNVCIFGNLSPETLV--GVVVSTNRLEEEINNQLLLQKYP-------PP---RYITVHCEPRLPNL--------
80 Variola_virus                                   10.3%   DYNSLYPNVCIFGNLSPETLV--GVVVSSNRLEEEINNQLLLQKYP-------PP---RYITVHCEPRLPNL--------
81 Volepox_virus                                   10.8%   DYNSLYPNVCIFGNLSPETLV--GVVVSTNRLEEEINNQLLLQKYP-------SP---RYITVRCEPRLPNL--------
82 Wiseana_iridescent_virus                        14.4%   DFNSLYPNLIIAYNIDYSTCV-------FDPTIPD----ELCHIME-------WE---DH--ISCKHDPKVI--------
83 Yaba_monkey_tumor_virus                         10.8%   DYNSLYPNVCIFGNLSPETLV--GVFVSNNTLEAEINKQNISKMYP-------PP---RYISINCEPRSPEL--------
84 Yellowstone_lake_phycodnavirus_1                17.0%   DFASLYPSIMCAHNLCYSTLV------MDPKF-------DNLPGVT-------YE---QF--------------------
   consensus/100%                                          Dhts.YPp.h...shs.p..........................h...................................
   consensus/90%                                           DatSLYPs.hh..Nhs.pphh  .......t.............h.       ..   .h............
   consensus/80%                                           DapSLYPs.hhthNls.pshs  .....s.t..........h.hh.       h.   ph....h..t....
   consensus/70%                                           DasSLYPohhhttNls.pohl      .ssph... ...tthphh.       at   ph..htt..c...h

                                                   1361             .          .          .          4          .          .          .          . 14
 1 Acanthamoeba_castellanii_mamavirus             100.0%   KEYHRFAQEIITDEQINRELKDIFDKINTVFENNVAIIQNQKYFTEKNISELIDKHKNISDSKIEDIEFDESLSDKRKNK
 2 Acanthamoeba_polyphaga_mimivirus                98.6%   KEYHRFAQEIITDEQINRELKDIFDKINTVFENNVAIIQNQKYFTEKNISELIDKHKNISDSKIEDIEFDESLSDKRKNK
 3 Acanthamoeba_polyphaga_moumouvirus              56.0%   ------------------------------------------------------------------------------RN
 4 Amsacta_moorei_entomopoxvirus                   10.1%   --------------------------------------------------------------------------------
 5 Anomala_cuprea_entomopoxvirus                    9.7%   --------------------------------------------------------------------------------
 6 ASFV_BAV71                                       9.7%   --------------------------------------------------------------------------------
 7 ASFV_E75                                         9.7%   --------------------------------------------------------------------------------
 8 Brazilian_marseillevirus                        12.6%   --------------------------------------------------------------------------------
 9 Cafeteria_roenbergensis_virus_BV-PW1            20.9%   --------------------------------------------------------------------------------
10 Cannes_8_virus                                  13.3%   --------------------------------------------------------------------------------
11 Catovirus_CTV1                                  24.3%   --------------------------------------------KKYPQASIIIF-------------------------
12 Cedratvirus_A11                                 12.1%   --------------------------------------------------------------------------------
13 Choristoneura_biennis_entomopoxvirus            10.5%   --------------------------------------------------------------------------------
14 Common_midwife_toad_ranavirus                   13.2%   -----------------------------------------------EYERLSSE-------------------------
15 Cowpox_virus                                    10.5%   --------------------------------------------------------------------------------
16 Diadromus_pulchellus_ascovirus_4a               12.7%   --------------------------------------------------------------------------------
17 Ectromelia_virus                                10.5%   --------------------------------------------------------------------------------
18 Emiliania_huxleyi_virus_145                     14.6%   --------------------------------------------------------------------------------
19 Emiliania_huxleyi_virus_86                      14.6%   --------------------------------------------------------------------------------
20 European_catfish_virus                          13.1%   -----------------------------------------------EYERLSSE-------------------------
21 Faustovirus_D3                                   8.8%   --------------------------------------------NTFGTVSGWMV-------------------------
22 Faustovirus_E12                                  8.9%   --------------------------------------------NIIGTVSGWMV-------------------------
23 Faustovirus_E24                                  8.9%   --------------------------------------------NIIGTVSGWMVSHGNIYD------------------
24 Faustovirus_E9                                   9.0%   --------------------------------------------NTFSTVSGWMVAHGNI--------------------
```

```
25 Faustovirus_Liban                               9.0%    --------------------------------------------NTIGTVSGWMV-------------------------
26 Feldmannia_species_virus                       13.4%    --------------------------------------------------------------------------------
27 Frog_virus_3                                   13.2%    -----------------------------------------------EYERLSSE-------------------------
28 Heliothis_virescens_ascovirus_3e               11.3%    -----------------------------------------------EREKLKSI-----------------FEDLAKVE
29 Hokovirus_HKV1                                 20.3%    --------------------------------------------------------------------------------
30 Infectious_spleen_and_kidney_necrosis_virus    12.7%    --------------------------------------------------------------------------------
31 Insectomime_virus                              12.3%    --------------------------------------------------------------------------------
32 Invertebrate_iridescent_virus_22               13.4%    --------------------------------------------------------------------------------
33 Invertebrate_iridescent_virus_3                14.2%    --------------------------------------------------------------------------------
34 Kaumoebavirus                                  10.2%    --------------------------------------------------------------------------------
35 Klosneuvirus_KNV1                              15.8%    --------------------------------------------------------------------------------
36 Kurlavirus_BKC-1                               12.6%    --------------------------------------------------------------------------------
37 Lausannevirus                                  12.4%    --------------------------------------------------------------------------------
38 Lumpy_skin_disease_virus_NI-2490               10.5%    --------------------------------------------------------------------------------
39 Lymphocystis_disease_virus                     13.5%    --------------------------------------------------------------------------------
40 Marseillevirus_marseillevirus_T19              13.3%    --------------------------------------------------------------------------------
41 Megavirus_chiliensis                           60.1%    KEHHRFAQELISDKQINTEMKDVFDKINKTMDNNIETILQQTYFTIKNIEDKIKKEKLITDSRIEDISFNDDFSTKKKAK
42 Megavirus_courdo7                              60.4%    KEHHRFAQELINDKQINTEMKDVFDKINKTMDNNIETILQQTYFTIKNIEDKIKKEKLITDSRIEDISFNDDFSTKKKAK
43 Megavirus_ursino                               60.1%    KEHHRFAQELISDKQINTEMKDVFDKITKTMNNNIETILQQTYFTIKNIEDKIKKEKLITDSRIEDISFNDDFSTKKKVK
44 Melbournevirus                                 13.3%    --------------------------------------------------------------------------------
45 Micromonas_pusilla_virus_SP1                   16.3%    --------------------------------------------------------------------------------
46 Mollivirus_sibericum                            9.9%    --------------------------------------------------------------------------------
47 Molluscum_contagiosum_virus_subtype_1           9.1%    --------------------------------------------------------------------------------
48 Monkeypox_virus                                10.5%    --------------------------------------------------------------------------------
49 Myxoma_virus                                   10.7%    --------------------------------------------------------------------------------
50 Noumeavirus                                    12.5%    --------------------------------------------------------------------------------
51 Orf_virus                                      10.2%    --------------------------------------------------------------------------------
52 Organic_Lake_phycodnavirus_1                   18.2%    --------------------------------------------------------------------------------
53 Orpheovirus_LCC2                               12.8%    --------------------------------------------------------------------------------
54 Ostreococcus_lucimarinus_virus_1               16.7%    --------------------------------------------------------------------------------
55 Ostreococcus_tauri_virus_OtV5                  16.7%    --------------------------------------------------------------------------------
56 Pacmanvirus_A23                                 9.1%    --------------------------------------------NKRLSQEGWAVRHNGIFNTRKDTNIVTQYVKYISFT
57 Pandoravirus_dulcis                             9.2%    --------------------------------------------------------------------------------
58 Pandoravirus_inopinatum                         8.8%    --------------------------------------------------------------------------------
59 Pandoravirus_salinus                           10.5%    --------------------------------------------------------------------------------
60 Paramecium_bursaria_Chlorella_virus_CVK2       15.4%    --------------------------------------------------------------------------------
61 Paramecium_bursaria_chlorella_virus_MT325      15.1%    --------------------------------------------------------------------------------
62 Paramecium_bursaria_Chlorella_virus_NYs1       14.2%    --------------------------------------------------------------------------------
63 Phaeocystis_globosa_virus                      16.9%    --------------------------------------------------------------------------------
64 Phaeocystis_globosa_virus_14T                  16.8%    --------------------------------------------------------------------------------
65 Pithovirus_massiliensis                        11.6%    --------------------------------------------------------------------------------
66 Pithovirus_sibericum                           11.8%    --------------------------------------------------------------------------------
67 Port-miou_virus                                12.4%    --------------------------------------------------------------------------------
68 Powai_lake_megavirus                           60.0%    KEHHRFAQELISDKQINTEMKDIFDKITKIMNNNIAIILEQTHFTVKNIEDKIKKEKLLTDSRIEDISFNDDFSTKKKAK
69 Scale_drop_disease_virus                       12.8%    --------------------------------------------------------------------------------
70 Short-finned_eel_ranavirus                     13.1%    -----------------------------------------------EYERLSSE-------------------------
71 Singapore_grouper_iridovirus                   13.3%    -----------------------------------------------EYDRLSLE-------------------------
72 Skunkpox_virus                                 11.0%    --------------------------------------------------------------------------------
73 Spodoptera_frugiperda_ascovirus_1a             10.9%    --------------------------------------------ELKSLRSMFEM---------------KALTDKRKRI
74 Testudo_hermanni_ranavirus                     13.2%    -----------------------------------------------EYERLSSE-------------------------
75 Tiger_frog_virus                               13.1%    -----------------------------------------------EYERLSSE-------------------------
76 Tokyovirus_A1                                  12.9%    --------------------------------------------------------------------------------
77 Trichoplusia_ni_ascovirus_2c                   10.4%    --------------------------------------------EFESLKKILYP---------------KLQQNNTPSK
78 Tunisvirus_fontaine2                           12.2%    --------------------------------------------------------------------------------
79 Vaccinia_virus                                 10.6%    --------------------------------------------------------------------------------
80 Variola_virus                                  10.3%    --------------------------------------------------------------------------------
81 Volepox_virus                                  10.8%    --------------------------------------------------------------------------------
82 Wiseana_iridescent_virus                       14.4%    --------------------------------------------------------------------------------
83 Yaba_monkey_tumor_virus                        10.8%    --------------------------------------------------------------------------------
84 Yellowstone_lake_phycodnavirus_1               17.0%    --------------------------------------------------------------------------------
   consensus/100%                                          ................................................................................
   consensus/90%                                                                                       ................          ..........
   consensus/80%                                                                                          ........
   consensus/70%

                                                      1441         :         .         .         .         .         5         .         . 15
 1 Acanthamoeba_castellanii_mamavirus            100.0%    LVDAEKDSLDKNIGFYQKIKSQIDKIKLDSKIEIDNLSKNLNEEEKSKQINKMELNTKNLISKVFSKYLITEQQREELIV
 2 Acanthamoeba_polyphaga_mimivirus               98.6%    LVDAEKDSLDKNIGFYQKIKSQIDKIKLDSKIEIDNLSKNLNEEEKSKQINKMELNTKNLISKVFSKYLITEQQREELIV
 3 Acanthamoeba_polyphaga_moumouvirus             56.0%    LDGTPKKEHHRFAQEIISDKQINIEMKDIFQKIKQNMEKNLKIIEDQLYFNLKNIEDKIKKEKEITEAKIEDISFNDNIS
 4 Amsacta_moorei_entomopoxvirus                  10.1%    -------------------------------------------------------------------------------K
 5 Anomala_cuprea_entomopoxvirus                   9.7%    -----------------------------------------------------------------------------KTI
 6 ASFV_BAV71                                      9.7%    --------------------------------------------------------------------------------
 7 ASFV_E75                                        9.7%    --------------------------------------------------------------------------------
 8 Brazilian_marseillevirus                       12.6%    --------------------------------------------------------KNKKKDQEV------------VCG
 9 Cafeteria_roenbergensis_virus_BV-PW1           20.9%    -----------------------------------------------------------------------------TGN
10 Cannes_8_virus                                 13.3%    --------------------------------------------------------KKKKKDEEV------------VCG
11 Catovirus_CTV1                                 24.3%    -------------------KDRLQKTIKKYKELKYVIEQVKKNNGDNVTSIYDIDKEGKKRFLVTEITINEKEFR--LVN
12 Cedratvirus_A11                                12.1%    --------------------------------------------------------------------------------
13 Choristoneura_biennis_entomopoxvirus           10.5%    -------------------------------------------------------------------------------K
14 Common_midwife_toad_ranavirus                  13.2%    LWDLDSEAREL----RRERDAIPRKRVDDRRLVVDTLNTVLDAQR-----------MLRTTRATLKNQLGTKT----VCA
15 Cowpox_virus                                   10.5%    -------------------------------------------------------------------------------I
16 Diadromus_pulchellus_ascovirus_4a              12.7%    -----------------------------------------QKRVLETLIETTHDKARKREFSKKRAEIIKKINKKPICQ
17 Ectromelia_virus                               10.5%    -------------------------------------------------------------------------------I
18 Emiliania_huxleyi_virus_145                    14.6%    -----------------------------------------------------------------------------ISP
19 Emiliania_huxleyi_virus_86                     14.6%    -----------------------------------------------------------------------------ISP
20 European_catfish_virus                         13.1%    LWDLDSEAREL----RRERDAIPRKRVDDRRLVVDTLNTVLDAQR-----------MLRTTRAALKNQLGTKT----VCA
21 Faustovirus_D3                                  8.8%    ---AHGNIYDPKVDTQVVNEFEKEITYKIVDADNTKTLKFVQPIRAEWTAPVEETPADIHAEVEAARVAGMEVLR--EVK
22 Faustovirus_E12                                 8.9%    -SHGNIYDPKKDKQVVNEYEKQVVYKVLNDNKQVTSMHTFVQPIRAEWTAPVEETPADIRATIDEAKAAGAKVLR--EVK
23 Faustovirus_E24                                 8.9%    --------PKKDKQVVNEYEKQVVYKVLNDNKQVTSMHTFVQPIRAEWTAPVEETPADIRATIDEAKAAGAKVLR--EVK
24 Faustovirus_E9                                  9.0%    YDPRVDTQVVNEFEKEITYKVQVNDSDNNDANNGVQTYKFVQPIRAEWTAPLEDTPDAIKTEVEAARAAGAKVVR--EVK
25 Faustovirus_Liban                               9.0%    -SHGNIYDPKKDKQVVNEYEKQVVYKVLDDNKQVTSMYTFVQPIRAEWTAPVEETPEDIKTSIDAVKAAGAKVLR--EVK
26 Feldmannia_species_virus                       13.4%    -----------------------------------------------------------------------------ISP
27 Frog_virus_3                                   13.2%    LWDLDSEAREL----RRERDAIPRKRVDDRRLVVDTLNTVLDAQR-----------MLRTTRAALKNQLGTKT----VCA
28 Heliothis_virescens_ascovirus_3e               11.3%    KRKFIGVQPITKYFKPTSTTRKPVDDVEEHSECNGFTDDNYNDSPVSSIKLSSVDLQRAAIRLKVLKSRMSGGSK--LCA
29 Hokovirus_HKV1                                 20.3%    -----------------------------------------------------------------------------GIN
30 Infectious_spleen_and_kidney_necrosis_virus    12.7%    --------------------------KQHALGIEIGVLQCNMAALPRRATQERARLRERIADMKIQYASMTPAAV--KCN
31 Insectomime_virus                              12.3%    --------------------------------------------------------KNKKKDQEV------------VCG
32 Invertebrate_iridescent_virus_22               13.4%    --------------------------------EKNRLTKLIDELTSKKKSDKALISKLRKQRSEITKSLNKNV----MCE
33 Invertebrate_iridescent_virus_3                14.2%    --------------------------------EKERLTQLINTLKNKEE-----VSRMRKERSEITKSLSKNV----MCE
34 Kaumoebavirus                                  10.2%    ------------------------------------------------------------TAQNFPYNRWFRLQKHVVEW
35 Klosneuvirus_KNV1                              15.8%    ------------------------------------------------------------------------------GS
36 Kurlavirus_BKC-1                               12.6%    --------------------------------------------------------KKKKKDQEV------------VCG
37 Lausannevirus                                  12.4%    --------------------------------------------------------KKKKKDQEV------------VCG
38 Lumpy_skin_disease_virus_NI-2490               10.5%    -------------------------------------------------------------------------------I
```

```
39 Lymphocystis_disease_virus                 13.5%   ----------------------KIEEYTKSIDELTALIKTKKSIKNQEDQLLIFRCEDRVKSLRTKRADLKKSKGKIICA
40 Marseillevirus_marseillevirus_T19          13.3%   --------------------------------------------------------KKKKKDEEV------------VCG
41 Megavirus_chiliensis                       60.1%   LIEIEKIEMKNRLKIYLEIDEKIKEISIKKKLSIDNIDKSLDADQKSKEIGKIEKTYNENIKNVLEKYIISKEEREEMIT
42 Megavirus_courdo7                          60.4%   LIETEKIEMKNRLKIYLEIDEKIKEISIKKKLSIDNIDKSLDADQKSKEIGKIEKTYNENIKNVLEKYIISKEEREEMIT
43 Megavirus_ursino                           60.1%   LIETEKIEMKNRLKIYLEIDEKIKEINIKKKLSIDNIDKSLDADQKSKEIDKIEKTYNENIKNVLEKYIISKEEREEMIT
44 Melbournevirus                             13.3%   --------------------------------------------------------KKKKKDEEV------------VCG
45 Micromonas_pusilla_virus_SP1               16.3%   -----------------------------------------------------------------------------EIG
46 Mollivirus_sibericum                        9.9%   --------------------------------------------------------PRGYNVPKPPGLVAIKIVRHPDMD
47 Molluscum_contagiosum_virus_subtype_1       9.1%   -------------------------------------------------------------------------------Y
48 Monkeypox_virus                            10.5%   -------------------------------------------------------------------------------I
49 Myxoma_virus                               10.7%   -------------------------------------------------------------------------------V
50 Noumeavirus                                12.5%   --------------------------------------------------------KKKKKDQEV------------VCG
51 Orf_virus                                  10.2%   -------------------------------------------------------------------------------V
52 Organic_Lake_phycodnavirus_1               18.2%   --------------------------------------------------------VRNTPTSAATKQ---------LTG
53 Orpheovirus_LCC2                           12.8%   ------------------------------------------ANDDDFDVGDDEEDDDEEDDDELESSKQTKKKVTTKII
54 Ostreococcus_lucimarinus_virus_1           16.7%   -----------------------------------------------------------------------------RVA
55 Ostreococcus_tauri_virus_OtV5              16.7%   -----------------------------------------------------------------------------NIG
56 Pacmanvirus_A23                             9.1%   CSDPELGPLQFKFKAEDGPTVEQKKQLEIYKSRKQIADVPIKAEQQKLNEIIKNCPEGVKINKLPEYIEQKKILD--EVK
57 Pandoravirus_dulcis                         9.2%   --------------------------------------GALNRVFASRRDWLPGQFAVDTDRPGVPRTVAYREVS--PAP
58 Pandoravirus_inopinatum                     8.8%   --------------------------------------SALNRVFASRRDWLPGQFAVDTDRPGIQRTVAYREVT--PAT
59 Pandoravirus_salinus                       10.5%   --------------------------------------TALNRVFASRPDWLPGQFAVDTDRRGIQRTVAYREVT--PAA
60 Paramecium_bursaria_Chlorella_virus_CVK2   15.4%   -----------------------------------------------------------------------------ETG
61 Paramecium_bursaria_chlorella_virus_MT325  15.1%   -----------------------------------------------------------------------------GTG
62 Paramecium_bursaria_Chlorella_virus_NYs1   14.2%   -----------------------------------------------------------------------------ETG
63 Phaeocystis_globosa_virus                  16.9%   --------------------------------------------------------LRKTPKAAEKKT---------VVG
64 Phaeocystis_globosa_virus_14T              16.8%   --------------------------------------------------------LRKTPKAAEKKT---------VVG
65 Pithovirus_massiliensis                    11.6%   -----------------------------------------------------------------------------VEK
66 Pithovirus_sibericum                       11.8%   -----------------------------------------------------------------------------VEK
67 Port-miou_virus                            12.4%   --------------------------------------------------------KKKKKDQEV------------VCG
68 Powai_lake_megavirus                       60.0%   LIETEKIEMKNRLKIYLEIDEKIKEINLKKKLSIDNIDKSLNADEKSKEISKIENTYNENIQNVLEKYIISKEEREEMIN
69 Scale_drop_disease_virus                   12.8%   -------------------EYDKLRNVLGQLQTKQAALNLNKKLHKEQLAKLVKQIKEVKTQLKETKPTSMKN----KCS
70 Short-finned_eel_ranavirus                 13.1%   LWDLDSEAREL----RRERDAIPRKRADDRRLVVDTLNTVLDAQK-----------MLRTTRATLKNQLGTKT----VCA
71 Singapore_grouper_iridovirus               13.3%   LWDLEREAKEL----RRERDGISRKNAAGRQTVQDTLNAVLSAQK-----------LLRTTRAAIKNSLGTKT----VCA
72 Skunkpox_virus                             11.0%   -------------------------------------------------------------------------------I
73 Spodoptera_frugiperda_ascovirus_1a         10.9%   GVTAITKYFKPIQGPRIIQKIMDDRYAESTCRYDDGDEFCENDDEKEVIAAIKTPLEMQRAAIRLKVLKSRASGGSVLCA
74 Testudo_hermanni_ranavirus                 13.2%   LWDLESEAREL----RRERDAIPRKRVDDRRLVVDTLNTVLDAQR-----------MLRTTRATLKNQLGTKT----VCA
75 Tiger_frog_virus                           13.1%   LWDLDSEAREL----RRERDAIPRKRVDDRRLVVDTLNTVLDAQR-----------MLRTTRAALKNQLGTKT----VCA
76 Tokyovirus_A1                              12.9%   --------------------------------------------------------KKKKKDEEV------------VCG
77 Trichoplusia_ni_ascovirus_2c               10.4%   RSPKGDGVVSNFFLPRNNKRCITNENDDVDEDEDQDILNMVTRTKQPSIEEQRATIRYKLLKARISAMSGKR-----VCE
78 Tunisvirus_fontaine2                       12.2%   --------------------------------------------------------KNKKKDQEV------------VCG
79 Vaccinia_virus                             10.6%   -------------------------------------------------------------------------------I
80 Variola_virus                              10.3%   -------------------------------------------------------------------------------I
81 Volepox_virus                              10.8%   -------------------------------------------------------------------------------I
82 Wiseana_iridescent_virus                   14.4%   --------------------------------EKIKLTKTIDELTSKKGYDKLLVSQLRKQRSEITKSLNKNV----MCA
83 Yaba_monkey_tumor_virus                    10.8%   -------------------------------------------------------------------------------V
84 Yellowstone_lake_phycodnavirus_1           17.0%   --------------------------------------------------------------------------------
   consensus/100%                                     ................................................................................
   consensus/90%                                      ................................................................................
   consensus/80%                                      ...........    .............................................................  ...
   consensus/70%                                                            ....................................................   .ht

                                               1521          .         .         :         .         .         .         .        6 16
 1 Acanthamoeba_castellanii_mamavirus        100.0%   LE------KERAKRSVNAEKAKVYNTVDGITVRYGILPEILTELLNKRKETN--------------GKLA---------N
 2 Acanthamoeba_polyphaga_mimivirus           98.6%   LE------KERAKRSVNAEKAKVYNTVDGITVRYGILPEILTELLNKRKETN--------------GKLA---------N
 3 Acanthamoeba_polyphaga_moumouvirus         56.0%   SKEKLVMEKERAEKEIASEKSKVYNVVNGKTVRYGILPEILTELLNKRKETN--------------LRLA---------K
 4 Amsacta_moorei_entomopoxvirus              10.1%   MY------KFILMDRR----------------ELGVTTQMVKDGREMKNMYK--------------DLKN---------K
 5 Anomala_cuprea_entomopoxvirus               9.7%   YT------YIVTDKSS-----------------PGLISIILMKGLDERNKYK--------------QLKS---------E
 6 ASFV_BAV71                                  9.7%   ----------WFVRHN------------NVPDKMGLYPKVLIDLLNKRTALK--------------QELK---------K
 7 ASFV_E75                                    9.7%   ----------WFVRHN------------NVPDKMGLYPKVLIDLLNKRTALK--------------QELK---------K
 8 Brazilian_marseillevirus                   12.6%   HF------KQRFKKSK---------VLENGEIQEGVLPRMLRHVLARRKAVK--------------GEMG---------Q
 9 Cafeteria_roenbergensis_virus_BV-PW1       20.9%   WQ------YRRFAKLP---------------DEFGVVPTILRTLLGERKAVK--------------KLMK---------V
10 Cannes_8_virus                             13.3%   HF------VQRFKKSR---------VLDDGTVEEGVLPRMLRGVLTQRKVVK--------------KMMS---------D
11 Catovirus_CTV1                             24.3%   FE------TSKFAEKL--------------DGTKGIIPEILQGLLDAREHTK--------------KLME---------G
12 Cedratvirus_A11                            12.1%   HF------KNVFVKEH---------------IKKGIFPELLGELIDKRNAVK--------------RDMR----------
13 Choristoneura_biennis_entomopoxvirus       10.5%   TY------KFIVMDRR----------------KPGIITQMIDKGMKSKNEYK--------------NLKN---------I
14 Common_midwife_toad_ranavirus              13.2%   CR------RLAFLEPA---------------TKKGVMPTILTDLLDGRKRAK--------------KAKA---------E
15 Cowpox_virus                               10.5%   SE------IAIFDRSI-----------------EGTIPRLLRTFLAERARYK--------------KMLK---------Q
16 Diadromus_pulchellus_ascovirus_4a          12.7%   PN------SFRFIKQE--------------VYGKGVLPTIIQTLLDARKSVR--------------AQMK---------N
17 Ectromelia_virus                           10.5%   SE------IAIFDRSI-----------------EGTIPRLLRTFLAERARYK--------------KMLK---------Q
18 Emiliania_huxleyi_virus_145                14.6%   TE------EYTFALNV-----------------PGVLTHMLKYLLGARKKAK--------------KQMA---------A
19 Emiliania_huxleyi_virus_86                 14.6%   TE------EYTFALNV-----------------PGVLTHMLKYLLGARKKAK--------------KQMA---------A
20 European_catfish_virus                     13.1%   CR------RLAFLEPA---------------TKKGVMPTILTNLLDGRKRAK--------------KAKA---------E
21 Faustovirus_D3                              8.8%   YL------PKPARSPL-------------INEKMGIFGYVLKKLFDKRVPIK--------------KEFVKLCEILEIMK
22 Faustovirus_E12                             8.9%   YL------PKPSREPL-------------VNERMGIFGYVLKKLFDKRVPIK--------------REFVKLCKIMEIMK
23 Faustovirus_E24                             8.9%   YL------PKPSREPL-------------VNERMGIFGYVLKKLFDKRVPIK--------------REFVKLCKIMEIMK
24 Faustovirus_E9                              9.0%   YL------PKPSRAPL-------------VNEKMGIFGYVLKKLFDKRVPIK--------------KEFVKLCEILEIMK
25 Faustovirus_Liban                           9.0%   YL------PKPSREPL-------------VNERMGIFGYVLKKLFDKRVPIK--------------REFVKLCKIMEIMK
26 Feldmannia_species_virus                   13.4%   GV------YETFATRP---------------GHKGILSAIEEDLGEARRQTK--------------AAMK---------V
27 Frog_virus_3                               13.2%   CR------LLAFLEPA---------------TKKGVMPTILTDLLDGRKRAK--------------KAKA---------E
28 Heliothis_virescens_ascovirus_3e           11.3%   KQ------RVRILKTR-----------------RGLLPDLVEYFLDARRKVR--------------AEMK---------N
29 Hokovirus_HKV1                             20.3%   FS------VCRFAKRK--------------DGKIGILPKILKHLLDQRKIMR--------------NRA----------E
30 Infectious_spleen_and_kidney_necrosis_virus 12.7%  VF------SFRFTHAH-----------------EGVLPRVLRNLLESRARIR--------------ARIK---------T
31 Insectomime_virus                          12.3%   HF------KQRFKKSK---------VLENGEIQEGVLPRMLRRVLARRKAVK--------------EEMG---------Q
32 Invertebrate_iridescent_virus_22           13.4%   KR------KYRFLKVT-----------NQNPEFKGVLPTIVQSLLDARKETR--------------KEIS---------N
33 Invertebrate_iridescent_virus_3            14.2%   KR------KYRFLKST-----------TEDGRFKGVLPTIVQNMLDARKETR--------------AEMG---------R
34 Kaumoebavirus                              10.2%   AE------RTFIAWTILPPADDNNMVDKEYVKWHGLFPRVLGDIFDVRLRMKLRVAQLKGEVEKLEVEMK---------A
35 Klosneuvirus_KNV1                          15.8%   IN------TCKFAWPK--------------DGKLGILPEILTELLNKRSEMR--------------NLAE---------E
36 Kurlavirus_BKC-1                           12.6%   HF------KQRFKKSK---------VLENGEIQEGVLPRMLRRVLARRKAVK--------------GEMG---------Q
37 Lausannevirus                              12.4%   HF------KQRFRKSK---------VLENGEVQEGVLPRMLRRVLARRKAVK--------------GEMG---------Q
38 Lumpy_skin_disease_virus_NI-2490           10.5%   SE------IAVFDRNV-----------------EGIIPKLLKTFLTERARYK--------------KKYK---------E
39 Lymphocystis_disease_virus                 13.5%   KR------NYHFIKSI---------------HKKGVVPAIVADLLEYRKRVK--------------VQIA---------E
40 Marseillevirus_marseillevirus_T19          13.3%   HF------VQRFKKSR---------VLDDGTVEEGVLPRMLRGVLTQRKVVK--------------KMMS---------D
41 Megavirus_chiliensis                       60.1%   ME------REKATKSMNDEKSKVYNIVDGKMVRYGILPEILTELLNKRKETN--------------GRLA---------V
42 Megavirus_courdo7                          60.4%   ME------REKATKSMNDEKSKVYNIVDGKMVRYGILPEILTELLNKRKETN--------------GRLA---------V
43 Megavirus_ursino                           60.1%   ME------QEKATKSMNDEKSKVYNVVDGKMVRYGILPEILTELLNKRKETN--------------GRLA---------I
44 Melbournevirus                             13.3%   HF------VQRFKKSR---------VLDDGTVEEGVLPRMLRGVLTQRKVVK--------------KMMS---------D
45 Micromonas_pusilla_virus_SP1               16.3%   DR------KYKFAQGV-----------------ESLLPSILLELKQFRKNAK--------------KDMA---------A
46 Mollivirus_sibericum                        9.9%   NI------EDMFVLHI-----------------KGAIPILLGTLKTSRAAVR--------------KDMG---------I
47 Molluscum_contagiosum_virus_subtype_1       9.1%   SE------IAVFDRRR-----------------QGIIPKLLGTFMEQRARYK--------------ALMR---------A
48 Monkeypox_virus                            10.5%   SE------IAIFDRSI-----------------EGTIPRLLRTFLAERARYK--------------KMLK---------Q
49 Myxoma_virus                               10.7%   SE------IAVFDRSV-----------------EGIIPKLLKTFLTERLKYK--------------KMIK---------E
50 Noumeavirus                                12.5%   HF------KQRFKKSK---------VLENGEIQEGVLPRMLRRVLARRKAVK--------------GEMG---------Q
51 Orf_virus                                  10.2%   AE------VAIFDREA-----------------KGIIPMLLRSFLDARAKYK--------------KLMK---------A
52 Organic_Lake_phycodnavirus_1               18.2%   YK------ICRYAQFP--------------DNKKAILPSILQELLAARKATK--------------KQMQ---------K
```

```
53 Orpheovirus_LCC2                            12.8%   KY------RFRFIKAPIPEGNNNENKQYHEKGQQGILPELLTNLVNERNKAK--------------KEMA---------K
54 Ostreococcus_lucimarinus_virus_1            16.7%   DR------TYKFAQDV-----------------PSLLPAILLELKQFRKQAK--------------RDMA---------N
55 Ostreococcus_tauri_virus_OtV5               16.7%   DR------TYKFAQDV-----------------PSLLPAILAELKQFRKQAK--------------RDMA---------A
56 Pacmanvirus_A23                              9.1%   KQ------EVKVMRKV--IYDPVRGREALPGERMGLFPTIVKKLFDKRVPIK--------------AEFVHLSEMLESME
57 Pandoravirus_dulcis                          9.2%   SR------THVFVQHV-----------------QGVVPRILTALKNQRKKVR--------------ADQK---------A
58 Pandoravirus_inopinatum                      8.8%   GR------THVFVQHV-----------------QGVVPRILTALKNQRKRVR--------------ADQK---------A
59 Pandoravirus_salinus                        10.5%   GR------THVFVQHV-----------------QGVVPRILTALKNQRKRVR--------------ADQK---------A
60 Paramecium_bursaria_Chlorella_virus_CVK2    15.4%   LG------KFKYAQKN-----------DETGEGQGVVPALLDDLAKFRKLAK--------------KHMA---------E
61 Paramecium_bursaria_chlorella_virus_MT325   15.1%   IG------TFRYSQQS-----------------QGVVPALLDDLAKFRKNAK--------------KLMA---------A
62 Paramecium_bursaria_Chlorella_virus_NYs1    14.2%   LG------TFKYAQKN-----------DETGEGQGVVPALLDDLAKFRKQAK--------------KHMA---------E
63 Phaeocystis_globosa_virus                   16.9%   YK------ICRFAQFP---------------KGKAIMPAILEDLLSARKATK--------------KLMG---------K
64 Phaeocystis_globosa_virus_14T               16.8%   YK------ICRFAQFP---------------KGKAIMPAILEDLLSARKATK--------------KLMG---------K
65 Pithovirus_massiliensis                     11.6%   HF------RHLFVKEH---------------IKKGLLPELVAGLIQERTDVR--------------NSMR---------G
66 Pithovirus_sibericum                        11.8%   HF------RHLFVKEH---------------IKKGLLPELVAGLIQERTDVR--------------NSMR---------G
67 Port-miou_virus                             12.4%   HF------KQRFRKSK---------VLENGEVQEGVLPRMLRRVLARRKAVK--------------GEMG---------Q
68 Powai_lake_megavirus                        60.0%   ME------REKATKSMNDEKSKVYNVVDGKMVRYGILPEILTELLNKRKETN--------------GRLA---------V
69 Scale_drop_disease_virus                    12.8%   NF------SFGFYNAD---------------ARPGILPKVLSCLLSSRAKIR--------------AQMKLL-------D
70 Short-finned_eel_ranavirus                  13.1%   CR------RLAFLEPA---------------AKKGVMPTILTDLLDGRKRAK--------------KAKE---------E
71 Singapore_grouper_iridovirus                13.3%   TR------RLAFYKSD---------------TIKGVMPTILTNLLEGRARAK--------------KAKT---------A
72 Skunkpox_virus                              11.0%   SE------IAIFDRSV-----------------EGIIPRLLRTFLAERARYK--------------KMLK---------Q
73 Spodoptera_frugiperda_ascovirus_1a          10.9%   KQ------CVKILKTR-----------------RGILPDLVECLLQARKRVR--------------GNMK---------S
74 Testudo_hermanni_ranavirus                  13.2%   CR------RLAFLEPA---------------TKKGVMPTILTDLLDGRKRAK--------------KAKA---------D
75 Tiger_frog_virus                            13.1%   CR------RLAFLEPA---------------TKKGVMPTILTDLLDGRKRAK--------------KAKA---------E
76 Tokyovirus_A1                               12.9%   HF------VQRFKKSR---------ILDDGSVEEGVLPRMLRGVLTQRKVVK--------------KMMG---------D
77 Trichoplusia_ni_ascovirus_2c                10.4%   TQ------RLKVLKSS---------------VRPGILPNVVNKLLVERKRIR--------------NIAS---------V
78 Tunisvirus_fontaine2                        12.2%   HF------KQRFKKSK---------VLENGDIQEGVLPRMLRRVLARRKAVK--------------EEMG---------Q
79 Vaccinia_virus                              10.6%   SE------IAIFDRSI-----------------EGTIPRLLRTFLAERARYK--------------KMLK---------Q
80 Variola_virus                               10.3%   SE------IAIFDRSI-----------------EGTIPRLLRTFLAERARYK--------------KMLK---------Q
81 Volepox_virus                               10.8%   SE------IAIFDRSV-----------------EGIIPRLLRTFLAERARYK--------------KMLK---------Q
82 Wiseana_iridescent_virus                    14.4%   KR------KYRFLKVT-----------EENPEFKGVLPTVVQSLLDARKETR--------------KEMS---------Q
83 Yaba_monkey_tumor_virus                     10.8%   SE------IAVFDRNV-----------------EGTIPKLLKKFLAERLRYK--------------QLLK---------K
84 Yellowstone_lake_phycodnavirus_1            17.0%   -G------PHRFAQNV-----------------PSLLPVILTDLKAYRKKAK--------------KLMA---------Q
   consensus/100%                                      ..................................uhhs.h...hh..+..hp............................
   consensus/90%                                       ..      ..hh.p....................Glhs.hltthht.Rh.h+              t.ht         .
   consensus/80%                                       ..      ..hhhp.h         .........GlhPtlLpphhstRtth+              t.ht         t
   consensus/70%                                       h.      hhhFhcph           .......GllPplLppllstRpthK              t.ht         p

                                                   1601      .         .         .         .         :         .         .         . 16
 1 Acanthamoeba_castellanii_mamavirus         100.0%   E---KDP----FVKA--------------ILNALQLAFKVTA--------------------------------------
 2 Acanthamoeba_polyphaga_mimivirus            98.6%   E---KDP----FVKA--------------ILNALQLAFKVTA--------------------------------------
 3 Acanthamoeba_polyphaga_moumouvirus          56.0%   E---KDA----FVKA--------------ILNALQLAFKVTA--------------------------------------
 4 Amsacta_moorei_entomopoxvirus               10.1%   N---KDN----VDLHN-------------FYSSALYSKITI---------------------------------------
 5 Anomala_cuprea_entomopoxvirus                9.7%   N---SNN----EILCN-------------LYDNMQYGIKIII--------------------------------------
 6 ASFV_BAV71                                   9.7%   LGEKKEC----IHES--HPGFKELQFRHAMVDAKQKALKIFM--------------------------------------
 7 ASFV_E75                                     9.7%   LGEKKEC----IHES--HPGFKELQFRHAMVDAKQKALKIFM--------------------------------------
 8 Brazilian_marseillevirus                    12.6%   A---ARN----ADKEVDPEKKAQFKTKEKVANASQLALKIAA--------------------------------------
 9 Cafeteria_roenbergensis_virus_BV-PW1        20.9%   E---KDP----FKYA--------------ILDAKQLALKVTA--------------------------------------
10 Cannes_8_virus                              13.3%   A---QKL----ADAETDPEKKKELTAKVKVFNSTQLALKIAA--------------------------------------
11 Catovirus_CTV1                              24.3%   E---SDK----FKKS--------------VLDGKQLAEKVTA--------------------------------------
12 Cedratvirus_A11                             12.1%   ----GVDVESLFYK---------------VCDKKQLGLKVVA--------------------------------------
13 Choristoneura_biennis_entomopoxvirus        10.5%   N---KNN----PVLYN-------------YYTSALYSKITI---------------------------------------
14 Common_midwife_toad_ranavirus               13.2%   A---KDS----ITKI--------------TMDKRQLAYKVSA--------------------------------------
15 Cowpox_virus                                10.5%   A---TSS----TEKA--------------IYDSMQYTYKIVA--------------------------------------
16 Diadromus_pulchellus_ascovirus_4a           12.7%   E---RDP----NIIA--------------ILNQRQLSYKVSA--------------------------------------
17 Ectromelia_virus                            10.5%   A---TSS----TEKA--------------IYDSMQYTYKIVA--------------------------------------
18 Emiliania_huxleyi_virus_145                 14.6%   A---KTP----EEKA--------------IYNARQLALKISC--------------------------------------
19 Emiliania_huxleyi_virus_86                  14.6%   A---KTP----EEKA--------------IYNARQLALKISC--------------------------------------
20 European_catfish_virus                      13.1%   A---KDS----ITKI--------------TMDKRQLAYKVSA--------------------------------------
21 Faustovirus_D3                               8.8%   KQGVNEL----VYTDGKTYHLHEVEFDAAKLDAVQKALKVLS--------------------------------------
22 Faustovirus_E12                              8.9%   K---NELTTMVYDVDGKTYHLKDVEFDAAKLDAIQKALKVLC--------------------------------------
23 Faustovirus_E24                              8.9%   K---NELTTMVYDVDGKTYHLKDVEFDAAKLDAIQKALKVLC--------------------------------------
24 Faustovirus_E9                               9.0%   KQGVTEL----VYTDGRTYHRHEVEFDAAKLDAVQKALKVLS--------------------------------------
25 Faustovirus_Liban                            9.0%   K---NELTTMVYDVDGKTYHLKDVEFDAAKLDAIQKALKVLC--------------------------------------
26 Feldmannia_species_virus                    13.4%   E---KDS----KKLQ--------------LLNSKQLAQKVTM--------------------------------------
27 Frog_virus_3                                13.2%   A---KDS----ITKI--------------TMDKRQLAYKVSA--------------------------------------
28 Heliothis_virescens_ascovirus_3e            11.3%   V---SDP----LARD--------------ILDKSQLAYKVTA--------------------------------------
29 Hokovirus_HKV1                              20.3%   AVAETDK----FLSK--------------VLDGLQLAYKVTA--------------------------------------
30 Infectious_spleen_and_kidney_necrosis_virus 12.7%   T---DDP----DIRA--------------VLDKRQLAYKISA--------------------------------------
31 Insectomime_virus                           12.3%   A---GKS----AEKESDPEKKAKFETKKKVANASQLALKIAA--------------------------------------
32 Invertebrate_iridescent_virus_22            13.4%   L---KNK----LKEINNEQQKREIETTIDILNQRQLAYKISA--------------------------------------
33 Invertebrate_iridescent_virus_3             14.2%   L---KKRLGTAVGEE-----ATHLQTQIAILNQRQLAYKVSA--------------------------------------
34 Kaumoebavirus                               10.2%   AEEAGDE----ELAKKLAHKLEELELERNIVDSQQKAAKVLM--------------------------------------
35 Klosneuvirus_KNV1                           15.8%   E---KDP----FKKK--------------VLDGLQLALKLSL--------------------------------------
36 Kurlavirus_BKC-1                            12.6%   A---ARN----ADNEPDPEKKAEFKTKEKVANASQLALKIAA--------------------------------------
37 Lausannevirus                               12.4%   A---ARN----AEKETDPEKKAEFETKEKVANASQLALKIAA--------------------------------------
38 Lumpy_skin_disease_virus_NI-2490            10.5%   A---TLS----TDKS--------------IYNSMQYTYKIIA--------------------------------------
39 Lymphocystis_disease_virus                  13.5%   T---TDS----TLKI--------------VLDKRQLACKISA--------------------------------------
40 Marseillevirus_marseillevirus_T19           13.3%   A---QKL----ADAETDPEKKKELTAKVKVFNSTQLALKIAA--------------------------------------
41 Megavirus_chiliensis                        60.1%   E---KDS----F-VKA-------------ILNALQLAFKVTA--------------------------------------
42 Megavirus_courdo7                           60.4%   E---KDS----FVKA--------------ILNALQLAFKVTA--------------------------------------
43 Megavirus_ursino                            60.1%   E---KDS----FVKA--------------ILNALQLAFKVTA--------------------------------------
44 Melbournevirus                              13.3%   A---QKL----ADAETDPEKKKELTAKVKVFNSTQLALKIAA--------------------------------------
45 Micromonas_pusilla_virus_SP1                16.3%   A---TGS----MKE---------------VYNGKQLAYKVSM--------------------------------------
46 Mollivirus_sibericum                         9.9%   M---EESKLENTPRYA-------------ILNRRQNNIKVFG--------------------------------------
47 Molluscum_contagiosum_virus_subtype_1        9.1%   A---SSG----LERT--------------LYDSMQYVYKVVA--------------------------------------
48 Monkeypox_virus                             10.5%   A---TSS----TEKA--------------IYDSMQYTYKIVA--------------------------------------
49 Myxoma_virus                                10.7%   T---NDV----VEKA--------------IYDSMQYTYKIIA--------------------------------------
50 Noumeavirus                                 12.5%   A---ARN----ADNEPDPEKKAEFKTKEKVANASQLALKIAA--------------------------------------
51 Orf_virus                                   10.2%   A---ETA----VDRE--------------IYNSMQYTYKITA--------------------------------------
52 Organic_Lake_phycodnavirus_1                18.2%   E---TDP----FKKN--------------ILDKRQLSIKITA--------------------------------------
53 Orpheovirus_LCC2                            12.8%   Y---AEG----TVEHI-------------VLDKRQNALKVSA--------------------------------------
54 Ostreococcus_lucimarinus_virus_1            16.7%   A----TG----FMKE--------------VYNGKQLAYKISM--------------------------------------
55 Ostreococcus_tauri_virus_OtV5               16.7%   A----TG----FMKE--------------VYNGKQLAYKVSM--------------------------------------
56 Pacmanvirus_A23                              9.1%   NMDVKEWTVKNPDGSESVVKYEDIEFSKNKVESKQKAIKVLA--------------------------------------
57 Pandoravirus_dulcis                          9.2%   Y---EKG----TALWG-------------VYENRQLGIKITANCFPADDHEILTETGFMNYAAVVEHFKHHARLSVACYV
58 Pandoravirus_inopinatum                      8.8%   Y---EKG----TALWG-------------VYENRQLGIKITA--------------------------------------
59 Pandoravirus_salinus                        10.5%   Y---EKG----TALWG-------------VYENRQLGIKITANCFPADDHEILTETGFMNYAAVTEHFKRHAHLSVACYV
60 Paramecium_bursaria_Chlorella_virus_CVK2    15.4%   AKRNGDD----FKEA--------------LYDAQQRSFKVVM--------------------------------------
61 Paramecium_bursaria_chlorella_virus_MT325   15.1%   AHKEGDD----FKEA--------------LYDASQRSYKVVM--------------------------------------
62 Paramecium_bursaria_Chlorella_virus_NYs1    14.2%   AKKNDDE----FREA--------------LYDAQQRSFKVVM--------------------------------------
63 Phaeocystis_globosa_virus                   16.9%   E---EDP----FKQN--------------IYDKRQLSIKVTA--------------------------------------
64 Phaeocystis_globosa_virus_14T               16.8%   E---EDP----FKQN--------------IYDKRQLSIKVTA--------------------------------------
65 Pithovirus_massiliensis                     11.6%   E---KCQ----ITLD--------------TKEKRQLGLKVVA--------------------------------------
66 Pithovirus_sibericum                        11.8%   E---KCQ----ITLD--------------TKEKRQLGLKVVA--------------------------------------
```

```
67 Port-miou_virus                             12.4%   A---ARN----AEKETDPEKKAEFETKEKVANASQLALKIAA--------------------------------------
68 Powai_lake_megavirus                        60.0%   E---KDS----FVKA--------------ILNALQLAFKVTA--------------------------------------
69 Scale_drop_disease_virus                    12.8%   P---NDP----MKS---------------VLDKRQLAYKISA--------------------------------------
70 Short-finned_eel_ranavirus                  13.1%   A---KDS----ITKI--------------TMDKRQLAYKVSA--------------------------------------
71 Singapore_grouper_iridovirus                13.3%   A---KDP----ITRI--------------TMDKRQLAYKVSA--------------------------------------
72 Skunkpox_virus                              11.0%   A---TSS----TEKA--------------IYDSMQYTYKIVA--------------------------------------
73 Spodoptera_frugiperda_ascovirus_1a          10.9%   V---TDP----MARD--------------ILDKSQLAYKVTA--------------------------------------
74 Testudo_hermanni_ranavirus                  13.2%   A---KDS----ITKI--------------TMDKRQLAYKVSA--------------------------------------
75 Tiger_frog_virus                            13.1%   A---KDS----ITKI--------------TMDKRQLAYKVSA--------------------------------------
76 Tokyovirus_A1                               12.9%   M---QKL----ADAETDPEKKKELLAKVKVFNSTQLALKIAA--------------------------------------
77 Trichoplusia_ni_ascovirus_2c                10.4%   P---GDP----VEKA--------------LLDKRQLAYKITA--------------------------------------
78 Tunisvirus_fontaine2                        12.2%   A---GKS----AEKESDPEKKAKFETKKKVANASQLALKIAA--------------------------------------
79 Vaccinia_virus                              10.6%   A---TSS----TEKA--------------IYDSMQYTYKIVA--------------------------------------
80 Variola_virus                               10.3%   A---TSS----TEKA--------------IYDSMQYTYKIIA--------------------------------------
81 Volepox_virus                               10.8%   A---TSS----TEKA--------------IYDSMQYTYKIVA--------------------------------------
82 Wiseana_iridescent_virus                    14.4%   L---TES----LKNKICEQQQKEIKTTIDILNQRQLAYKISA--------------------------------------
83 Yaba_monkey_tumor_virus                     10.8%   S---SSS----TEKA--------------IYDSMQYTYKIVA--------------------------------------
84 Yellowstone_lake_phycodnavirus_1            17.0%   A---EGT----PMEA--------------IYNGQQLAYKISM--------------------------------------
   consensus/100%                                      .............................hhpt...s.Kl........................................
   consensus/90%                                       ....tp.    ..................hhst.QhshKl.h
   consensus/80%                                       t   pp.    h.t...............hhsthQhuhKlss
   consensus/70%                                       t   pcs    htpt.             lhsshQhuhKlsA

                                                   1681        .         7         .         .         .         .         :         . 17

 1 Acanthamoeba_castellanii_mamavirus         100.0%   --------------------------------------------------------------------------------
 2 Acanthamoeba_polyphaga_mimivirus            98.6%   --------------------------------------------------------------------------------
 3 Acanthamoeba_polyphaga_moumouvirus          56.0%   --------------------------------------------------------------------------------
 4 Amsacta_moorei_entomopoxvirus               10.1%   --------------------------------------------------------------------------------
 5 Anomala_cuprea_entomopoxvirus                9.7%   --------------------------------------------------------------------------------
 6 ASFV_BAV71                                   9.7%   --------------------------------------------------------------------------------
 7 ASFV_E75                                     9.7%   --------------------------------------------------------------------------------
 8 Brazilian_marseillevirus                    12.6%   --------------------------------------------------------------------------------
 9 Cafeteria_roenbergensis_virus_BV-PW1        20.9%   --------------------------------------------------------------------------------
10 Cannes_8_virus                              13.3%   --------------------------------------------------------------------------------
11 Catovirus_CTV1                              24.3%   --------------------------------------------------------------------------------
12 Cedratvirus_A11                             12.1%   --------------------------------------------------------------------------------
13 Choristoneura_biennis_entomopoxvirus        10.5%   --------------------------------------------------------------------------------
14 Common_midwife_toad_ranavirus               13.2%   --------------------------------------------------------------------------------
15 Cowpox_virus                                10.5%   --------------------------------------------------------------------------------
16 Diadromus_pulchellus_ascovirus_4a           12.7%   --------------------------------------------------------------------------------
17 Ectromelia_virus                            10.5%   --------------------------------------------------------------------------------
18 Emiliania_huxleyi_virus_145                 14.6%   --------------------------------------------------------------------------------
19 Emiliania_huxleyi_virus_86                  14.6%   --------------------------------------------------------------------------------
20 European_catfish_virus                      13.1%   --------------------------------------------------------------------------------
21 Faustovirus_D3                               8.8%   --------------------------------------------------------------------------------
22 Faustovirus_E12                              8.9%   --------------------------------------------------------------------------------
23 Faustovirus_E24                              8.9%   --------------------------------------------------------------------------------
24 Faustovirus_E9                               9.0%   --------------------------------------------------------------------------------
25 Faustovirus_Liban                            9.0%   --------------------------------------------------------------------------------
26 Feldmannia_species_virus                    13.4%   --------------------------------------------------------------------------------
27 Frog_virus_3                                13.2%   --------------------------------------------------------------------------------
28 Heliothis_virescens_ascovirus_3e            11.3%   --------------------------------------------------------------------------------
29 Hokovirus_HKV1                              20.3%   --------------------------------------------------------------------------------
30 Infectious_spleen_and_kidney_necrosis_virus 12.7%   --------------------------------------------------------------------------------
31 Insectomime_virus                           12.3%   --------------------------------------------------------------------------------
32 Invertebrate_iridescent_virus_22            13.4%   --------------------------------------------------------------------------------
33 Invertebrate_iridescent_virus_3             14.2%   --------------------------------------------------------------------------------
34 Kaumoebavirus                               10.2%   --------------------------------------------------------------------------------
35 Klosneuvirus_KNV1                           15.8%   --------------------------------------------------------------------------------
36 Kurlavirus_BKC-1                            12.6%   --------------------------------------------------------------------------------
37 Lausannevirus                               12.4%   --------------------------------------------------------------------------------
38 Lumpy_skin_disease_virus_NI-2490            10.5%   --------------------------------------------------------------------------------
39 Lymphocystis_disease_virus                  13.5%   --------------------------------------------------------------------------------
40 Marseillevirus_marseillevirus_T19           13.3%   --------------------------------------------------------------------------------
41 Megavirus_chiliensis                        60.1%   --------------------------------------------------------------------------------
42 Megavirus_courdo7                           60.4%   --------------------------------------------------------------------------------
43 Megavirus_ursino                            60.1%   --------------------------------------------------------------------------------
44 Melbournevirus                              13.3%   --------------------------------------------------------------------------------
45 Micromonas_pusilla_virus_SP1                16.3%   --------------------------------------------------------------------------------
46 Mollivirus_sibericum                         9.9%   --------------------------------------------------------------------------------
47 Molluscum_contagiosum_virus_subtype_1        9.1%   --------------------------------------------------------------------------------
48 Monkeypox_virus                             10.5%   --------------------------------------------------------------------------------
49 Myxoma_virus                                10.7%   --------------------------------------------------------------------------------
50 Noumeavirus                                 12.5%   --------------------------------------------------------------------------------
51 Orf_virus                                   10.2%   --------------------------------------------------------------------------------
52 Organic_Lake_phycodnavirus_1                18.2%   --------------------------------------------------------------------------------
53 Orpheovirus_LCC2                            12.8%   --------------------------------------------------------------------------------
54 Ostreococcus_lucimarinus_virus_1            16.7%   --------------------------------------------------------------------------------
55 Ostreococcus_tauri_virus_OtV5               16.7%   --------------------------------------------------------------------------------
56 Pacmanvirus_A23                              9.1%   --------------------------------------------------------------------------------
57 Pandoravirus_dulcis                          9.2%   DGQLQYHDIAESNVVANTGDHRLVQFEAGSSTGKRATTNGVSLCCTDNHRIYARVGSTWGNRIWRRKGTESQESAAPPFT
58 Pandoravirus_inopinatum                      8.8%   --------------------------------------------------------------------------------
59 Pandoravirus_salinus                        10.5%   DGQLQYHDITEANVVANTGDHRLVQFEAGSSTGKRATTNGVSLCCTDNHRIYARVGPTWGNRIWRRKGTESQESAAPPFT
60 Paramecium_bursaria_Chlorella_virus_CVK2    15.4%   --------------------------------------------------------------------------------
61 Paramecium_bursaria_chlorella_virus_MT325   15.1%   --------------------------------------------------------------------------------
62 Paramecium_bursaria_Chlorella_virus_NYs1    14.2%   --------------------------------------------------------------------------------
63 Phaeocystis_globosa_virus                   16.9%   --------------------------------------------------------------------------------
64 Phaeocystis_globosa_virus_14T               16.8%   --------------------------------------------------------------------------------
65 Pithovirus_massiliensis                     11.6%   --------------------------------------------------------------------------------
66 Pithovirus_sibericum                        11.8%   --------------------------------------------------------------------------------
67 Port-miou_virus                             12.4%   --------------------------------------------------------------------------------
68 Powai_lake_megavirus                        60.0%   --------------------------------------------------------------------------------
69 Scale_drop_disease_virus                    12.8%   --------------------------------------------------------------------------------
70 Short-finned_eel_ranavirus                  13.1%   --------------------------------------------------------------------------------
71 Singapore_grouper_iridovirus                13.3%   --------------------------------------------------------------------------------
72 Skunkpox_virus                              11.0%   --------------------------------------------------------------------------------
73 Spodoptera_frugiperda_ascovirus_1a          10.9%   --------------------------------------------------------------------------------
74 Testudo_hermanni_ranavirus                  13.2%   --------------------------------------------------------------------------------
75 Tiger_frog_virus                            13.1%   --------------------------------------------------------------------------------
76 Tokyovirus_A1                               12.9%   --------------------------------------------------------------------------------
77 Trichoplusia_ni_ascovirus_2c                10.4%   --------------------------------------------------------------------------------
78 Tunisvirus_fontaine2                        12.2%   --------------------------------------------------------------------------------
79 Vaccinia_virus                              10.6%   --------------------------------------------------------------------------------
80 Variola_virus                               10.3%   --------------------------------------------------------------------------------
```

```
81 Volepox_virus                                     10.8%      --------------------------------------------------------------------------------
82 Wiseana_iridescent_virus                          14.4%      --------------------------------------------------------------------------------
83 Yaba_monkey_tumor_virus                           10.8%      --------------------------------------------------------------------------------
84 Yellowstone_lake_phycodnavirus_1                  17.0%      --------------------------------------------------------------------------------
   consensus/100%                                               ................................................................................
   consensus/90%
   consensus/80%
   consensus/70%

                                                           1761         .         .         .         8         .         .         .         . 18
 1 Acanthamoeba_castellanii_mamavirus               100.0%      --------------------------------------------------------------------------------
 2 Acanthamoeba_polyphaga_mimivirus                  98.6%      --------------------------------------------------------------------------------
 3 Acanthamoeba_polyphaga_moumouvirus                56.0%      --------------------------------------------------------------------------------
 4 Amsacta_moorei_entomopoxvirus                     10.1%      --------------------------------------------------------------------------------
 5 Anomala_cuprea_entomopoxvirus                      9.7%      --------------------------------------------------------------------------------
 6 ASFV_BAV71                                         9.7%      --------------------------------------------------------------------------------
 7 ASFV_E75                                           9.7%      --------------------------------------------------------------------------------
 8 Brazilian_marseillevirus                          12.6%      --------------------------------------------------------------------------------
 9 Cafeteria_roenbergensis_virus_BV-PW1              20.9%      --------------------------------------------------------------------------------
10 Cannes_8_virus                                    13.3%      --------------------------------------------------------------------------------
11 Catovirus_CTV1                                    24.3%      --------------------------------------------------------------------------------
12 Cedratvirus_A11                                   12.1%      --------------------------------------------------------------------------------
13 Choristoneura_biennis_entomopoxvirus              10.5%      --------------------------------------------------------------------------------
14 Common_midwife_toad_ranavirus                     13.2%      --------------------------------------------------------------------------------
15 Cowpox_virus                                      10.5%      --------------------------------------------------------------------------------
16 Diadromus_pulchellus_ascovirus_4a                 12.7%      --------------------------------------------------------------------------------
17 Ectromelia_virus                                  10.5%      --------------------------------------------------------------------------------
18 Emiliania_huxleyi_virus_145                       14.6%      --------------------------------------------------------------------------------
19 Emiliania_huxleyi_virus_86                        14.6%      --------------------------------------------------------------------------------
20 European_catfish_virus                            13.1%      --------------------------------------------------------------------------------
21 Faustovirus_D3                                     8.8%      --------------------------------------------------------------------------------
22 Faustovirus_E12                                    8.9%      --------------------------------------------------------------------------------
23 Faustovirus_E24                                    8.9%      --------------------------------------------------------------------------------
24 Faustovirus_E9                                     9.0%      --------------------------------------------------------------------------------
25 Faustovirus_Liban                                  9.0%      --------------------------------------------------------------------------------
26 Feldmannia_species_virus                          13.4%      --------------------------------------------------------------------------------
27 Frog_virus_3                                      13.2%      --------------------------------------------------------------------------------
28 Heliothis_virescens_ascovirus_3e                  11.3%      --------------------------------------------------------------------------------
29 Hokovirus_HKV1                                    20.3%      --------------------------------------------------------------------------------
30 Infectious_spleen_and_kidney_necrosis_virus       12.7%      --------------------------------------------------------------------------------
31 Insectomime_virus                                 12.3%      --------------------------------------------------------------------------------
32 Invertebrate_iridescent_virus_22                  13.4%      --------------------------------------------------------------------------------
33 Invertebrate_iridescent_virus_3                   14.2%      --------------------------------------------------------------------------------
34 Kaumoebavirus                                     10.2%      --------------------------------------------------------------------------------
35 Klosneuvirus_KNV1                                 15.8%      --------------------------------------------------------------------------------
36 Kurlavirus_BKC-1                                  12.6%      --------------------------------------------------------------------------------
37 Lausannevirus                                     12.4%      --------------------------------------------------------------------------------
38 Lumpy_skin_disease_virus_NI-2490                  10.5%      --------------------------------------------------------------------------------
39 Lymphocystis_disease_virus                        13.5%      --------------------------------------------------------------------------------
40 Marseillevirus_marseillevirus_T19                 13.3%      --------------------------------------------------------------------------------
41 Megavirus_chiliensis                              60.1%      --------------------------------------------------------------------------------
42 Megavirus_courdo7                                 60.4%      --------------------------------------------------------------------------------
43 Megavirus_ursino                                  60.1%      --------------------------------------------------------------------------------
44 Melbournevirus                                    13.3%      --------------------------------------------------------------------------------
45 Micromonas_pusilla_virus_SP1                      16.3%      --------------------------------------------------------------------------------
46 Mollivirus_sibericum                               9.9%      --------------------------------------------------------------------------------
47 Molluscum_contagiosum_virus_subtype_1              9.1%      --------------------------------------------------------------------------------
48 Monkeypox_virus                                   10.5%      --------------------------------------------------------------------------------
49 Myxoma_virus                                      10.7%      --------------------------------------------------------------------------------
50 Noumeavirus                                       12.5%      --------------------------------------------------------------------------------
51 Orf_virus                                         10.2%      --------------------------------------------------------------------------------
52 Organic_Lake_phycodnavirus_1                      18.2%      --------------------------------------------------------------------------------
53 Orpheovirus_LCC2                                  12.8%      --------------------------------------------------------------------------------
54 Ostreococcus_lucimarinus_virus_1                  16.7%      --------------------------------------------------------------------------------
55 Ostreococcus_tauri_virus_OtV5                     16.7%      --------------------------------------------------------------------------------
56 Pacmanvirus_A23                                    9.1%      --------------------------------------------------------------------------------
57 Pandoravirus_dulcis                                9.2%      IQSAGDILAAGTRDPSTVVQFTAACSKGVALDGDDLPFAATLGLHTEDQIDAFVELYGYWLGDGWLDVSCQAIAFSPVKT
58 Pandoravirus_inopinatum                            8.8%      --------------------------------------------------------------------------------
59 Pandoravirus_salinus                              10.5%      IQSAGDILDAGTRDPSTMVQFTAVCSKGVTLDGGDLPFVEALGLRTEDQVDAFVELYGYWLGDGWLDVSCQAIAFSPVKT
60 Paramecium_bursaria_Chlorella_virus_CVK2          15.4%      --------------------------------------------------------------------------------
61 Paramecium_bursaria_chlorella_virus_MT325         15.1%      --------------------------------------------------------------------------------
62 Paramecium_bursaria_Chlorella_virus_NYs1          14.2%      --------------------------------------------------------------------------------
63 Phaeocystis_globosa_virus                         16.9%      --------------------------------------------------------------------------------
64 Phaeocystis_globosa_virus_14T                     16.8%      --------------------------------------------------------------------------------
65 Pithovirus_massiliensis                           11.6%      --------------------------------------------------------------------------------
66 Pithovirus_sibericum                              11.8%      --------------------------------------------------------------------------------
67 Port-miou_virus                                   12.4%      --------------------------------------------------------------------------------
68 Powai_lake_megavirus                              60.0%      --------------------------------------------------------------------------------
69 Scale_drop_disease_virus                          12.8%      --------------------------------------------------------------------------------
70 Short-finned_eel_ranavirus                        13.1%      --------------------------------------------------------------------------------
71 Singapore_grouper_iridovirus                      13.3%      --------------------------------------------------------------------------------
72 Skunkpox_virus                                    11.0%      --------------------------------------------------------------------------------
73 Spodoptera_frugiperda_ascovirus_1a                10.9%      --------------------------------------------------------------------------------
74 Testudo_hermanni_ranavirus                        13.2%      --------------------------------------------------------------------------------
75 Tiger_frog_virus                                  13.1%      --------------------------------------------------------------------------------
76 Tokyovirus_A1                                     12.9%      --------------------------------------------------------------------------------
77 Trichoplusia_ni_ascovirus_2c                      10.4%      --------------------------------------------------------------------------------
78 Tunisvirus_fontaine2                              12.2%      --------------------------------------------------------------------------------
79 Vaccinia_virus                                    10.6%      --------------------------------------------------------------------------------
80 Variola_virus                                     10.3%      --------------------------------------------------------------------------------
81 Volepox_virus                                     10.8%      --------------------------------------------------------------------------------
82 Wiseana_iridescent_virus                          14.4%      --------------------------------------------------------------------------------
83 Yaba_monkey_tumor_virus                           10.8%      --------------------------------------------------------------------------------
84 Yellowstone_lake_phycodnavirus_1                  17.0%      --------------------------------------------------------------------------------
   consensus/100%                                               ................................................................................
   consensus/90%
   consensus/80%
   consensus/70%

                                                           1841         :         .         .         .         .         9         .         . 19
 1 Acanthamoeba_castellanii_mamavirus               100.0%      --------------------------------------------------------------------------------
 2 Acanthamoeba_polyphaga_mimivirus                  98.6%      --------------------------------------------------------------------------------
 3 Acanthamoeba_polyphaga_moumouvirus                56.0%      --------------------------------------------------------------------------------
 4 Amsacta_moorei_entomopoxvirus                     10.1%      --------------------------------------------------------------------------------
```

```
 5 Anomala_cuprea_entomopoxvirus                  9.7%   --------------------------------------------------------------------------------
 6 ASFV_BAV71                                     9.7%   --------------------------------------------------------------------------------
 7 ASFV_E75                                       9.7%   --------------------------------------------------------------------------------
 8 Brazilian_marseillevirus                      12.6%   --------------------------------------------------------------------------------
 9 Cafeteria_roenbergensis_virus_BV-PW1          20.9%   --------------------------------------------------------------------------------
10 Cannes_8_virus                                13.3%   --------------------------------------------------------------------------------
11 Catovirus_CTV1                                24.3%   --------------------------------------------------------------------------------
12 Cedratvirus_A11                               12.1%   --------------------------------------------------------------------------------
13 Choristoneura_biennis_entomopoxvirus          10.5%   --------------------------------------------------------------------------------
14 Common_midwife_toad_ranavirus                 13.2%   --------------------------------------------------------------------------------
15 Cowpox_virus                                  10.5%   --------------------------------------------------------------------------------
16 Diadromus_pulchellus_ascovirus_4a             12.7%   --------------------------------------------------------------------------------
17 Ectromelia_virus                              10.5%   --------------------------------------------------------------------------------
18 Emiliania_huxleyi_virus_145                   14.6%   --------------------------------------------------------------------------------
19 Emiliania_huxleyi_virus_86                    14.6%   --------------------------------------------------------------------------------
20 European_catfish_virus                        13.1%   --------------------------------------------------------------------------------
21 Faustovirus_D3                                 8.8%   --------------------------------------------------------------------------------
22 Faustovirus_E12                                8.9%   --------------------------------------------------------------------------------
23 Faustovirus_E24                                8.9%   --------------------------------------------------------------------------------
24 Faustovirus_E9                                 9.0%   --------------------------------------------------------------------------------
25 Faustovirus_Liban                              9.0%   --------------------------------------------------------------------------------
26 Feldmannia_species_virus                      13.4%   --------------------------------------------------------------------------------
27 Frog_virus_3                                  13.2%   --------------------------------------------------------------------------------
28 Heliothis_virescens_ascovirus_3e              11.3%   --------------------------------------------------------------------------------
29 Hokovirus_HKV1                                20.3%   --------------------------------------------------------------------------------
30 Infectious_spleen_and_kidney_necrosis_virus   12.7%   --------------------------------------------------------------------------------
31 Insectomime_virus                             12.3%   --------------------------------------------------------------------------------
32 Invertebrate_iridescent_virus_22              13.4%   --------------------------------------------------------------------------------
33 Invertebrate_iridescent_virus_3               14.2%   --------------------------------------------------------------------------------
34 Kaumoebavirus                                 10.2%   --------------------------------------------------------------------------------
35 Klosneuvirus_KNV1                             15.8%   --------------------------------------------------------------------------------
36 Kurlavirus_BKC-1                              12.6%   --------------------------------------------------------------------------------
37 Lausannevirus                                 12.4%   --------------------------------------------------------------------------------
38 Lumpy_skin_disease_virus_NI-2490              10.5%   --------------------------------------------------------------------------------
39 Lymphocystis_disease_virus                    13.5%   --------------------------------------------------------------------------------
40 Marseillevirus_marseillevirus_T19             13.3%   --------------------------------------------------------------------------------
41 Megavirus_chiliensis                          60.1%   --------------------------------------------------------------------------------
42 Megavirus_courdo7                             60.4%   --------------------------------------------------------------------------------
43 Megavirus_ursino                              60.1%   --------------------------------------------------------------------------------
44 Melbournevirus                                13.3%   --------------------------------------------------------------------------------
45 Micromonas_pusilla_virus_SP1                  16.3%   --------------------------------------------------------------------------------
46 Mollivirus_sibericum                           9.9%   --------------------------------------------------------------------------------
47 Molluscum_contagiosum_virus_subtype_1          9.1%   --------------------------------------------------------------------------------
48 Monkeypox_virus                               10.5%   --------------------------------------------------------------------------------
49 Myxoma_virus                                  10.7%   --------------------------------------------------------------------------------
50 Noumeavirus                                   12.5%   --------------------------------------------------------------------------------
51 Orf_virus                                     10.2%   --------------------------------------------------------------------------------
52 Organic_Lake_phycodnavirus_1                  18.2%   --------------------------------------------------------------------------------
53 Orpheovirus_LCC2                              12.8%   --------------------------------------------------------------------------------
54 Ostreococcus_lucimarinus_virus_1              16.7%   --------------------------------------------------------------------------------
55 Ostreococcus_tauri_virus_OtV5                 16.7%   --------------------------------------------------------------------------------
56 Pacmanvirus_A23                                9.1%   --------------------------------------------------------------------------------
57 Pandoravirus_dulcis                            9.2%   ADSAYLAALFARLPLPVLTEDTRGPGAIGAFITPKPSEARQRARYSAWAKPHRHYIYTPSWWRYFAEQYGHKYSGAALEH
58 Pandoravirus_inopinatum                        8.8%   --------------------------------------------------------------------------------
59 Pandoravirus_salinus                          10.5%   ADSAYLAALFARLPLPLLTKDTRGPGAIGAFVTPEPAGVKRRARYTAWTTPHRHYIYTPSWWRYFAEQYGHKYSGAALEH
60 Paramecium_bursaria_Chlorella_virus_CVK2      15.4%   --------------------------------------------------------------------------------
61 Paramecium_bursaria_chlorella_virus_MT325     15.1%   --------------------------------------------------------------------------------
62 Paramecium_bursaria_Chlorella_virus_NYs1      14.2%   --------------------------------------------------------------------------------
63 Phaeocystis_globosa_virus                     16.9%   --------------------------------------------------------------------------------
64 Phaeocystis_globosa_virus_14T                 16.8%   --------------------------------------------------------------------------------
65 Pithovirus_massiliensis                       11.6%   --------------------------------------------------------------------------------
66 Pithovirus_sibericum                          11.8%   --------------------------------------------------------------------------------
67 Port-miou_virus                               12.4%   --------------------------------------------------------------------------------
68 Powai_lake_megavirus                          60.0%   --------------------------------------------------------------------------------
69 Scale_drop_disease_virus                      12.8%   --------------------------------------------------------------------------------
70 Short-finned_eel_ranavirus                    13.1%   --------------------------------------------------------------------------------
71 Singapore_grouper_iridovirus                  13.3%   --------------------------------------------------------------------------------
72 Skunkpox_virus                                11.0%   --------------------------------------------------------------------------------
73 Spodoptera_frugiperda_ascovirus_1a            10.9%   --------------------------------------------------------------------------------
74 Testudo_hermanni_ranavirus                    13.2%   --------------------------------------------------------------------------------
75 Tiger_frog_virus                              13.1%   --------------------------------------------------------------------------------
76 Tokyovirus_A1                                 12.9%   --------------------------------------------------------------------------------
77 Trichoplusia_ni_ascovirus_2c                  10.4%   --------------------------------------------------------------------------------
78 Tunisvirus_fontaine2                          12.2%   --------------------------------------------------------------------------------
79 Vaccinia_virus                                10.6%   --------------------------------------------------------------------------------
80 Variola_virus                                 10.3%   --------------------------------------------------------------------------------
81 Volepox_virus                                 10.8%   --------------------------------------------------------------------------------
82 Wiseana_iridescent_virus                      14.4%   --------------------------------------------------------------------------------
83 Yaba_monkey_tumor_virus                       10.8%   --------------------------------------------------------------------------------
84 Yellowstone_lake_phycodnavirus_1              17.0%   --------------------------------------------------------------------------------
   consensus/100%                                        ................................................................................
   consensus/90%
   consensus/80%
   consensus/70%

                                                1921           .         .         :         .         .         .         .         0 20
 1 Acanthamoeba_castellanii_mamavirus           100.0%   --------------------------------------------------------------------------------
 2 Acanthamoeba_polyphaga_mimivirus              98.6%   --------------------------------------------------------------------------------
 3 Acanthamoeba_polyphaga_moumouvirus            56.0%   --------------------------------------------------------------------------------
 4 Amsacta_moorei_entomopoxvirus                 10.1%   --------------------------------------------------------------------------------
 5 Anomala_cuprea_entomopoxvirus                  9.7%   --------------------------------------------------------------------------------
 6 ASFV_BAV71                                     9.7%   --------------------------------------------------------------------------------
 7 ASFV_E75                                       9.7%   --------------------------------------------------------------------------------
 8 Brazilian_marseillevirus                      12.6%   --------------------------------------------------------------------------------
 9 Cafeteria_roenbergensis_virus_BV-PW1          20.9%   --------------------------------------------------------------------------------
10 Cannes_8_virus                                13.3%   --------------------------------------------------------------------------------
11 Catovirus_CTV1                                24.3%   --------------------------------------------------------------------------------
12 Cedratvirus_A11                               12.1%   --------------------------------------------------------------------------------
13 Choristoneura_biennis_entomopoxvirus          10.5%   --------------------------------------------------------------------------------
14 Common_midwife_toad_ranavirus                 13.2%   --------------------------------------------------------------------------------
15 Cowpox_virus                                  10.5%   --------------------------------------------------------------------------------
16 Diadromus_pulchellus_ascovirus_4a             12.7%   --------------------------------------------------------------------------------
17 Ectromelia_virus                              10.5%   --------------------------------------------------------------------------------
18 Emiliania_huxleyi_virus_145                   14.6%   --------------------------------------------------------------------------------
```

```
19 Emiliania_huxleyi_virus_86                     14.6%   --------------------------------------------------------------------------------
20 European_catfish_virus                         13.1%   --------------------------------------------------------------------------------
21 Faustovirus_D3                                  8.8%   --------------------------------------------------------------------------------
22 Faustovirus_E12                                 8.9%   --------------------------------------------------------------------------------
23 Faustovirus_E24                                 8.9%   --------------------------------------------------------------------------------
24 Faustovirus_E9                                  9.0%   --------------------------------------------------------------------------------
25 Faustovirus_Liban                               9.0%   --------------------------------------------------------------------------------
26 Feldmannia_species_virus                       13.4%   --------------------------------------------------------------------------------
27 Frog_virus_3                                   13.2%   --------------------------------------------------------------------------------
28 Heliothis_virescens_ascovirus_3e               11.3%   --------------------------------------------------------------------------------
29 Hokovirus_HKV1                                 20.3%   --------------------------------------------------------------------------------
30 Infectious_spleen_and_kidney_necrosis_virus    12.7%   --------------------------------------------------------------------------------
31 Insectomime_virus                              12.3%   --------------------------------------------------------------------------------
32 Invertebrate_iridescent_virus_22               13.4%   --------------------------------------------------------------------------------
33 Invertebrate_iridescent_virus_3                14.2%   --------------------------------------------------------------------------------
34 Kaumoebavirus                                  10.2%   --------------------------------------------------------------------------------
35 Klosneuvirus_KNV1                              15.8%   --------------------------------------------------------------------------------
36 Kurlavirus_BKC-1                               12.6%   --------------------------------------------------------------------------------
37 Lausannevirus                                  12.4%   --------------------------------------------------------------------------------
38 Lumpy_skin_disease_virus_NI-2490               10.5%   --------------------------------------------------------------------------------
39 Lymphocystis_disease_virus                     13.5%   --------------------------------------------------------------------------------
40 Marseillevirus_marseillevirus_T19              13.3%   --------------------------------------------------------------------------------
41 Megavirus_chiliensis                           60.1%   --------------------------------------------------------------------------------
42 Megavirus_courdo7                              60.4%   --------------------------------------------------------------------------------
43 Megavirus_ursino                               60.1%   --------------------------------------------------------------------------------
44 Melbournevirus                                 13.3%   --------------------------------------------------------------------------------
45 Micromonas_pusilla_virus_SP1                   16.3%   --------------------------------------------------------------------------------
46 Mollivirus_sibericum                            9.9%   --------------------------------------------------------------------------------
47 Molluscum_contagiosum_virus_subtype_1           9.1%   --------------------------------------------------------------------------------
48 Monkeypox_virus                                10.5%   --------------------------------------------------------------------------------
49 Myxoma_virus                                   10.7%   --------------------------------------------------------------------------------
50 Noumeavirus                                    12.5%   --------------------------------------------------------------------------------
51 Orf_virus                                      10.2%   --------------------------------------------------------------------------------
52 Organic_Lake_phycodnavirus_1                   18.2%   --------------------------------------------------------------------------------
53 Orpheovirus_LCC2                               12.8%   --------------------------------------------------------------------------------
54 Ostreococcus_lucimarinus_virus_1               16.7%   --------------------------------------------------------------------------------
55 Ostreococcus_tauri_virus_OtV5                  16.7%   --------------------------------------------------------------------------------
56 Pacmanvirus_A23                                 9.1%   --------------------------------------------------------------------------------
57 Pandoravirus_dulcis                             9.2%   VMQEALRSGSDLPARRPDAKPLAGKARALATLSPVRDSRPSGACAAQR--------------------------------
58 Pandoravirus_inopinatum                         8.8%   --------------------------------------------------------------------------------
59 Pandoravirus_salinus                           10.5%   VIQGALRSGADLPARRPDAKPSTGKARTLASSTSMGGGRLSAACATRRNRINDGVAERVCYSDHCASAGVWQPLTTAFPR
60 Paramecium_bursaria_Chlorella_virus_CVK2       15.4%   --------------------------------------------------------------------------------
61 Paramecium_bursaria_chlorella_virus_MT325      15.1%   --------------------------------------------------------------------------------
62 Paramecium_bursaria_Chlorella_virus_NYs1       14.2%   --------------------------------------------------------------------------------
63 Phaeocystis_globosa_virus                      16.9%   --------------------------------------------------------------------------------
64 Phaeocystis_globosa_virus_14T                  16.8%   --------------------------------------------------------------------------------
65 Pithovirus_massiliensis                        11.6%   --------------------------------------------------------------------------------
66 Pithovirus_sibericum                           11.8%   --------------------------------------------------------------------------------
67 Port-miou_virus                                12.4%   --------------------------------------------------------------------------------
68 Powai_lake_megavirus                           60.0%   --------------------------------------------------------------------------------
69 Scale_drop_disease_virus                       12.8%   --------------------------------------------------------------------------------
70 Short-finned_eel_ranavirus                     13.1%   --------------------------------------------------------------------------------
71 Singapore_grouper_iridovirus                   13.3%   --------------------------------------------------------------------------------
72 Skunkpox_virus                                 11.0%   --------------------------------------------------------------------------------
73 Spodoptera_frugiperda_ascovirus_1a             10.9%   --------------------------------------------------------------------------------
74 Testudo_hermanni_ranavirus                     13.2%   --------------------------------------------------------------------------------
75 Tiger_frog_virus                               13.1%   --------------------------------------------------------------------------------
76 Tokyovirus_A1                                  12.9%   --------------------------------------------------------------------------------
77 Trichoplusia_ni_ascovirus_2c                   10.4%   --------------------------------------------------------------------------------
78 Tunisvirus_fontaine2                           12.2%   --------------------------------------------------------------------------------
79 Vaccinia_virus                                 10.6%   --------------------------------------------------------------------------------
80 Variola_virus                                  10.3%   --------------------------------------------------------------------------------
81 Volepox_virus                                  10.8%   --------------------------------------------------------------------------------
82 Wiseana_iridescent_virus                       14.4%   --------------------------------------------------------------------------------
83 Yaba_monkey_tumor_virus                        10.8%   --------------------------------------------------------------------------------
84 Yellowstone_lake_phycodnavirus_1               17.0%   --------------------------------------------------------------------------------
   consensus/100%                                         ................................................................................
   consensus/90%
   consensus/80%
   consensus/70%

                                                     2001          .         .         .         .         :         .         .         . 20
 1 Acanthamoeba_castellanii_mamavirus            100.0%   --------------------------------------------------------------------------------
 2 Acanthamoeba_polyphaga_mimivirus               98.6%   --------------------------------------------------------------------------------
 3 Acanthamoeba_polyphaga_moumouvirus             56.0%   --------------------------------------------------------------------------------
 4 Amsacta_moorei_entomopoxvirus                  10.1%   --------------------------------------------------------------------------------
 5 Anomala_cuprea_entomopoxvirus                   9.7%   --------------------------------------------------------------------------------
 6 ASFV_BAV71                                      9.7%   --------------------------------------------------------------------------------
 7 ASFV_E75                                        9.7%   --------------------------------------------------------------------------------
 8 Brazilian_marseillevirus                       12.6%   --------------------------------------------------------------------------------
 9 Cafeteria_roenbergensis_virus_BV-PW1           20.9%   --------------------------------------------------------------------------------
10 Cannes_8_virus                                 13.3%   --------------------------------------------------------------------------------
11 Catovirus_CTV1                                 24.3%   --------------------------------------------------------------------------------
12 Cedratvirus_A11                                12.1%   --------------------------------------------------------------------------------
13 Choristoneura_biennis_entomopoxvirus           10.5%   --------------------------------------------------------------------------------
14 Common_midwife_toad_ranavirus                  13.2%   --------------------------------------------------------------------------------
15 Cowpox_virus                                   10.5%   --------------------------------------------------------------------------------
16 Diadromus_pulchellus_ascovirus_4a              12.7%   --------------------------------------------------------------------------------
17 Ectromelia_virus                               10.5%   --------------------------------------------------------------------------------
18 Emiliania_huxleyi_virus_145                    14.6%   --------------------------------------------------------------------------------
19 Emiliania_huxleyi_virus_86                     14.6%   --------------------------------------------------------------------------------
20 European_catfish_virus                         13.1%   --------------------------------------------------------------------------------
21 Faustovirus_D3                                  8.8%   --------------------------------------------------------------------------------
22 Faustovirus_E12                                 8.9%   --------------------------------------------------------------------------------
23 Faustovirus_E24                                 8.9%   --------------------------------------------------------------------------------
24 Faustovirus_E9                                  9.0%   --------------------------------------------------------------------------------
25 Faustovirus_Liban                               9.0%   --------------------------------------------------------------------------------
26 Feldmannia_species_virus                       13.4%   --------------------------------------------------------------------------------
27 Frog_virus_3                                   13.2%   --------------------------------------------------------------------------------
28 Heliothis_virescens_ascovirus_3e               11.3%   --------------------------------------------------------------------------------
29 Hokovirus_HKV1                                 20.3%   --------------------------------------------------------------------------------
30 Infectious_spleen_and_kidney_necrosis_virus    12.7%   --------------------------------------------------------------------------------
31 Insectomime_virus                              12.3%   --------------------------------------------------------------------------------
32 Invertebrate_iridescent_virus_22               13.4%   --------------------------------------------------------------------------------
```

```
33 Invertebrate_iridescent_virus_3              14.2%    --------------------------------------------------------------------------------
34 Kaumoebavirus                                10.2%    --------------------------------------------------------------------------------
35 Klosneuvirus_KNV1                            15.8%    --------------------------------------------------------------------------------
36 Kurlavirus_BKC-1                             12.6%    --------------------------------------------------------------------------------
37 Lausannevirus                                12.4%    --------------------------------------------------------------------------------
38 Lumpy_skin_disease_virus_NI-2490             10.5%    --------------------------------------------------------------------------------
39 Lymphocystis_disease_virus                   13.5%    --------------------------------------------------------------------------------
40 Marseillevirus_marseillevirus_T19            13.3%    --------------------------------------------------------------------------------
41 Megavirus_chiliensis                         60.1%    --------------------------------------------------------------------------------
42 Megavirus_courdo7                            60.4%    --------------------------------------------------------------------------------
43 Megavirus_ursino                             60.1%    --------------------------------------------------------------------------------
44 Melbournevirus                               13.3%    --------------------------------------------------------------------------------
45 Micromonas_pusilla_virus_SP1                 16.3%    --------------------------------------------------------------------------------
46 Mollivirus_sibericum                          9.9%    --------------------------------------------------------------------------------
47 Molluscum_contagiosum_virus_subtype_1         9.1%    --------------------------------------------------------------------------------
48 Monkeypox_virus                              10.5%    --------------------------------------------------------------------------------
49 Myxoma_virus                                 10.7%    --------------------------------------------------------------------------------
50 Noumeavirus                                  12.5%    --------------------------------------------------------------------------------
51 Orf_virus                                    10.2%    --------------------------------------------------------------------------------
52 Organic_Lake_phycodnavirus_1                 18.2%    --------------------------------------------------------------------------------
53 Orpheovirus_LCC2                             12.8%    --------------------------------------------------------------------------------
54 Ostreococcus_lucimarinus_virus_1             16.7%    --------------------------------------------------------------------------------
55 Ostreococcus_tauri_virus_OtV5                16.7%    --------------------------------------------------------------------------------
56 Pacmanvirus_A23                               9.1%    --------------------------------------------------------------------------------
57 Pandoravirus_dulcis                           9.2%    -------------------------NYIARRNKLTARYDAMFAANTGKRRTTPDAEEVRSAKWMWSWVWRRLHPGRLRLL
58 Pandoravirus_inopinatum                       8.8%    --------------------------------------------------------------------------------
59 Pandoravirus_salinus                         10.5%    HSPKGSGKATYGGKCRKCVSARAYQNRIARRTQLAARYDAMFAANTGKRQTALGAEEVKSAKWMWSWVWRRLHPDRLRLL
60 Paramecium_bursaria_Chlorella_virus_CVK2     15.4%    --------------------------------------------------------------------------------
61 Paramecium_bursaria_chlorella_virus_MT325    15.1%    --------------------------------------------------------------------------------
62 Paramecium_bursaria_Chlorella_virus_NYs1     14.2%    --------------------------------------------------------------------------------
63 Phaeocystis_globosa_virus                    16.9%    --------------------------------------------------------------------------------
64 Phaeocystis_globosa_virus_14T                16.8%    --------------------------------------------------------------------------------
65 Pithovirus_massiliensis                      11.6%    --------------------------------------------------------------------------------
66 Pithovirus_sibericum                         11.8%    --------------------------------------------------------------------------------
67 Port-miou_virus                              12.4%    --------------------------------------------------------------------------------
68 Powai_lake_megavirus                         60.0%    --------------------------------------------------------------------------------
69 Scale_drop_disease_virus                     12.8%    --------------------------------------------------------------------------------
70 Short-finned_eel_ranavirus                   13.1%    --------------------------------------------------------------------------------
71 Singapore_grouper_iridovirus                 13.3%    --------------------------------------------------------------------------------
72 Skunkpox_virus                               11.0%    --------------------------------------------------------------------------------
73 Spodoptera_frugiperda_ascovirus_1a           10.9%    --------------------------------------------------------------------------------
74 Testudo_hermanni_ranavirus                   13.2%    --------------------------------------------------------------------------------
75 Tiger_frog_virus                             13.1%    --------------------------------------------------------------------------------
76 Tokyovirus_A1                                12.9%    --------------------------------------------------------------------------------
77 Trichoplusia_ni_ascovirus_2c                 10.4%    --------------------------------------------------------------------------------
78 Tunisvirus_fontaine2                         12.2%    --------------------------------------------------------------------------------
79 Vaccinia_virus                               10.6%    --------------------------------------------------------------------------------
80 Variola_virus                                10.3%    --------------------------------------------------------------------------------
81 Volepox_virus                                10.8%    --------------------------------------------------------------------------------
82 Wiseana_iridescent_virus                     14.4%    --------------------------------------------------------------------------------
83 Yaba_monkey_tumor_virus                      10.8%    --------------------------------------------------------------------------------
84 Yellowstone_lake_phycodnavirus_1             17.0%    --------------------------------------------------------------------------------
   consensus/100%                                        ................................................................................
   consensus/90%
   consensus/80%
   consensus/70%

                                                  2081         .         1         .         .         .         .         :         . 21
 1 Acanthamoeba_castellanii_mamavirus          100.0%    --------------------------------------------------------------------------------
 2 Acanthamoeba_polyphaga_mimivirus             98.6%    --------------------------------------------------------------------------------
 3 Acanthamoeba_polyphaga_moumouvirus           56.0%    --------------------------------------------------------------------------------
 4 Amsacta_moorei_entomopoxvirus                10.1%    --------------------------------------------------------------------------------
 5 Anomala_cuprea_entomopoxvirus                 9.7%    --------------------------------------------------------------------------------
 6 ASFV_BAV71                                    9.7%    --------------------------------------------------------------------------------
 7 ASFV_E75                                      9.7%    --------------------------------------------------------------------------------
 8 Brazilian_marseillevirus                     12.6%    --------------------------------------------------------------------------------
 9 Cafeteria_roenbergensis_virus_BV-PW1         20.9%    --------------------------------------------------------------------------------
10 Cannes_8_virus                               13.3%    --------------------------------------------------------------------------------
11 Catovirus_CTV1                               24.3%    --------------------------------------------------------------------------------
12 Cedratvirus_A11                              12.1%    --------------------------------------------------------------------------------
13 Choristoneura_biennis_entomopoxvirus         10.5%    --------------------------------------------------------------------------------
14 Common_midwife_toad_ranavirus                13.2%    --------------------------------------------------------------------------------
15 Cowpox_virus                                 10.5%    --------------------------------------------------------------------------------
16 Diadromus_pulchellus_ascovirus_4a            12.7%    --------------------------------------------------------------------------------
17 Ectromelia_virus                             10.5%    --------------------------------------------------------------------------------
18 Emiliania_huxleyi_virus_145                  14.6%    --------------------------------------------------------------------------------
19 Emiliania_huxleyi_virus_86                   14.6%    --------------------------------------------------------------------------------
20 European_catfish_virus                       13.1%    --------------------------------------------------------------------------------
21 Faustovirus_D3                                8.8%    --------------------------------------------------------------------------------
22 Faustovirus_E12                               8.9%    --------------------------------------------------------------------------------
23 Faustovirus_E24                               8.9%    --------------------------------------------------------------------------------
24 Faustovirus_E9                                9.0%    --------------------------------------------------------------------------------
25 Faustovirus_Liban                             9.0%    --------------------------------------------------------------------------------
26 Feldmannia_species_virus                     13.4%    --------------------------------------------------------------------------------
27 Frog_virus_3                                 13.2%    --------------------------------------------------------------------------------
28 Heliothis_virescens_ascovirus_3e             11.3%    --------------------------------------------------------------------------------
29 Hokovirus_HKV1                               20.3%    --------------------------------------------------------------------------------
30 Infectious_spleen_and_kidney_necrosis_virus  12.7%    --------------------------------------------------------------------------------
31 Insectomime_virus                            12.3%    --------------------------------------------------------------------------------
32 Invertebrate_iridescent_virus_22             13.4%    --------------------------------------------------------------------------------
33 Invertebrate_iridescent_virus_3              14.2%    --------------------------------------------------------------------------------
34 Kaumoebavirus                                10.2%    --------------------------------------------------------------------------------
35 Klosneuvirus_KNV1                            15.8%    --------------------------------------------------------------------------------
36 Kurlavirus_BKC-1                             12.6%    --------------------------------------------------------------------------------
37 Lausannevirus                                12.4%    --------------------------------------------------------------------------------
38 Lumpy_skin_disease_virus_NI-2490             10.5%    --------------------------------------------------------------------------------
39 Lymphocystis_disease_virus                   13.5%    --------------------------------------------------------------------------------
40 Marseillevirus_marseillevirus_T19            13.3%    --------------------------------------------------------------------------------
41 Megavirus_chiliensis                         60.1%    --------------------------------------------------------------------------------
42 Megavirus_courdo7                            60.4%    --------------------------------------------------------------------------------
43 Megavirus_ursino                             60.1%    --------------------------------------------------------------------------------
44 Melbournevirus                               13.3%    --------------------------------------------------------------------------------
45 Micromonas_pusilla_virus_SP1                 16.3%    --------------------------------------------------------------------------------
46 Mollivirus_sibericum                          9.9%    --------------------------------------------------------------------------------
```

```
47 Molluscum_contagiosum_virus_subtype_1           9.1%   --------------------------------------------------------------------------------
48 Monkeypox_virus                                10.5%   --------------------------------------------------------------------------------
49 Myxoma_virus                                   10.7%   --------------------------------------------------------------------------------
50 Noumeavirus                                    12.5%   --------------------------------------------------------------------------------
51 Orf_virus                                      10.2%   --------------------------------------------------------------------------------
52 Organic_Lake_phycodnavirus_1                   18.2%   --------------------------------------------------------------------------------
53 Orpheovirus_LCC2                               12.8%   --------------------------------------------------------------------------------
54 Ostreococcus_lucimarinus_virus_1               16.7%   --------------------------------------------------------------------------------
55 Ostreococcus_tauri_virus_OtV5                  16.7%   --------------------------------------------------------------------------------
56 Pacmanvirus_A23                                 9.1%   --------------------------------------------------------------------------------
57 Pandoravirus_dulcis                             9.2%   LRGLRIADGDMAGGDRGGGAIYTTSRRFAEEVVRVAIHAGYTAMIQPRCAAGAVTSLNQKGVPIVATTQNWVVNYSDFTR
58 Pandoravirus_inopinatum                         8.8%   --------------------------------------------------------------------------------
59 Pandoravirus_salinus                           10.5%   LRGLRMADGDMAGGDRGGGAIYTSSRRFAEEVVRVAMHAGYTAMIQPRCAAGDVTGLNQKGVPIVATAQNWVVNYSDFTR
60 Paramecium_bursaria_Chlorella_virus_CVK2       15.4%   --------------------------------------------------------------------------------
61 Paramecium_bursaria_chlorella_virus_MT325      15.1%   --------------------------------------------------------------------------------
62 Paramecium_bursaria_Chlorella_virus_NYs1       14.2%   --------------------------------------------------------------------------------
63 Phaeocystis_globosa_virus                      16.9%   --------------------------------------------------------------------------------
64 Phaeocystis_globosa_virus_14T                  16.8%   --------------------------------------------------------------------------------
65 Pithovirus_massiliensis                        11.6%   --------------------------------------------------------------------------------
66 Pithovirus_sibericum                           11.8%   --------------------------------------------------------------------------------
67 Port-miou_virus                                12.4%   --------------------------------------------------------------------------------
68 Powai_lake_megavirus                           60.0%   --------------------------------------------------------------------------------
69 Scale_drop_disease_virus                       12.8%   --------------------------------------------------------------------------------
70 Short-finned_eel_ranavirus                     13.1%   --------------------------------------------------------------------------------
71 Singapore_grouper_iridovirus                   13.3%   --------------------------------------------------------------------------------
72 Skunkpox_virus                                 11.0%   --------------------------------------------------------------------------------
73 Spodoptera_frugiperda_ascovirus_1a             10.9%   --------------------------------------------------------------------------------
74 Testudo_hermanni_ranavirus                     13.2%   --------------------------------------------------------------------------------
75 Tiger_frog_virus                               13.1%   --------------------------------------------------------------------------------
76 Tokyovirus_A1                                  12.9%   --------------------------------------------------------------------------------
77 Trichoplusia_ni_ascovirus_2c                   10.4%   --------------------------------------------------------------------------------
78 Tunisvirus_fontaine2                           12.2%   --------------------------------------------------------------------------------
79 Vaccinia_virus                                 10.6%   --------------------------------------------------------------------------------
80 Variola_virus                                  10.3%   --------------------------------------------------------------------------------
81 Volepox_virus                                  10.8%   --------------------------------------------------------------------------------
82 Wiseana_iridescent_virus                       14.4%   --------------------------------------------------------------------------------
83 Yaba_monkey_tumor_virus                        10.8%   --------------------------------------------------------------------------------
84 Yellowstone_lake_phycodnavirus_1               17.0%   --------------------------------------------------------------------------------
   consensus/100%                                         ................................................................................
   consensus/90%
   consensus/80%
   consensus/70%

                                                 2161            .         .         .         2         .         .         .         .22
 1 Acanthamoeba_castellanii_mamavirus            100.0%   ------------------------------------------------------NSLYGQTGAPT-SPLYFIAIAACTTA
 2 Acanthamoeba_polyphaga_mimivirus               98.6%   ------------------------------------------------------NSLYGQTGAPT-SPLYFIAIAACTTA
 3 Acanthamoeba_polyphaga_moumouvirus             56.0%   ------------------------------------------------------NSLYGQTGAPT-SPIYFIAIAASTTA
 4 Amsacta_moorei_entomopoxvirus                  10.1%   ------------------------------------------------------NSMYGLSGSER-FIFNSPYCAEYCTV
 5 Anomala_cuprea_entomopoxvirus                   9.7%   ------------------------------------------------------NSIYGLLGSNQ-FIFRSKYCAQYCTA
 6 ASFV_BAV71                                      9.7%   ------------------------------------------------------NTFYGEAGNNL-SPFFLLPLAGGVTS
 7 ASFV_E75                                        9.7%   ------------------------------------------------------NTFYGEAGNNL-SPFFLLPLAGGVTS
 8 Brazilian_marseillevirus                       12.6%   ------------------------------------------------------NSAYGALGATQ-GISPLVEAAAAVTT
 9 Cafeteria_roenbergensis_virus_BV-PW1           20.9%   ------------------------------------------------------NSLYGQLGAAT-SPVRDRDIAACTTS
10 Cannes_8_virus                                 13.3%   ------------------------------------------------------NSGYGALGATQ-GLAPLVEAAAAVTT
11 Catovirus_CTV1                                 24.3%   ------------------------------------------------------NSLYGQTGSPV-SAIFMKEIAASTTA
12 Cedratvirus_A11                                12.1%   ------------------------------------------------------NSGYGFTGASQ-GFLPCKAIAASVTA
13 Choristoneura_biennis_entomopoxvirus           10.5%   ------------------------------------------------------NSLYGLLGSER-FDFNSPYCAEYCTA
14 Common_midwife_toad_ranavirus                  13.2%   ------------------------------------------------------NSMYGAMGVKR-GYLPFQDGAMTVTY
15 Cowpox_virus                                   10.5%   ------------------------------------------------------NSVYGLMGFRN-SALYSYASAKSCTS
16 Diadromus_pulchellus_ascovirus_4a              12.7%   ------------------------------------------------------NSMYGATGVRA-GALPFMPIAMCVTF
17 Ectromelia_virus                               10.5%   ------------------------------------------------------NSVYGLMGFRN-SALYSYASAKSCTS
18 Emiliania_huxleyi_virus_145                    14.6%   ------------------------------------------------------NSIYGFCGAEKLGKYPLGAIAKCTTF
19 Emiliania_huxleyi_virus_86                     14.6%   ------------------------------------------------------NSIYGFCGAEKLGKYPLGAIAKCTTF
20 European_catfish_virus                         13.1%   ------------------------------------------------------NSMYGAMGVKR-GYLPFQDGAMTVTY
21 Faustovirus_D3                                  8.8%   ------------------------------------------------------NTFYGESGNFR-SAIYALIVAAGITA
22 Faustovirus_E12                                 8.9%   ------------------------------------------------------NTFYGESGNFR-SAIYELLVAAGITS
23 Faustovirus_E24                                 8.9%   ------------------------------------------------------NTFYGESGNFR-SAIYELLVAAGITS
24 Faustovirus_E9                                  9.0%   ------------------------------------------------------NTFYGESGNFR-SAIYALIVAAGITA
25 Faustovirus_Liban                               9.0%   ------------------------------------------------------NTFYGESGNFR-SAIYELLVAAGITS
26 Feldmannia_species_virus                       13.4%   ------------------------------------------------------NSLYGFCGTVN-GCLPLVAIAAAVTC
27 Frog_virus_3                                   13.2%   ------------------------------------------------------NSMYGAMGVKR-GYLPFQDGAMTVTY
28 Heliothis_virescens_ascovirus_3e               11.3%   ------------------------------------------------------NSVYGSTGAVN-GKLPCQNVAKVTTA
29 Hokovirus_HKV1                                 20.3%   ------------------------------------------------------NSLYGQTGSSV-SAIYNKDIAASTTA
30 Infectious_spleen_and_kidney_necrosis_virus    12.7%   ------------------------------------------------------NSVYGTMGTQR-GYLPFMAGAMTTTY
31 Insectomime_virus                              12.3%   ------------------------------------------------------NSAYGALGATQ-GISPLVEAAAAVTT
32 Invertebrate_iridescent_virus_22               13.4%   ------------------------------------------------------NSMYGITGVKA-GMLPFMPVAMSITY
33 Invertebrate_iridescent_virus_3                14.2%   ------------------------------------------------------NSMYGITGVKA-GMLPFMPVAMSITF
34 Kaumoebavirus                                  10.2%   ------------------------------------------------------NSFYGEAGNQS-SCLFNIMISGTTTM
35 Klosneuvirus_KNV1                              15.8%   --------------------------------------------------------------------------------
36 Kurlavirus_BKC-1                               12.6%   ------------------------------------------------------NSAYGALGASQ-GIAPLVEAAAAVTT
37 Lausannevirus                                  12.4%   ------------------------------------------------------NSAYGALGASQ-GIAPLVEAAAAVTT
38 Lumpy_skin_disease_virus_NI-2490               10.5%   ------------------------------------------------------NSVYGLMGFKN-SVLYSYSSAKSCTA
39 Lymphocystis_disease_virus                     13.5%   ------------------------------------------------------NSIYGSMGVSK-GYLPFMPGAMCITR
40 Marseillevirus_marseillevirus_T19              13.3%   ------------------------------------------------------NSGYGALGATQ-GLAPLVEAAAAVTT
41 Megavirus_chiliensis                           60.1%   ------------------------------------------------------NSLYGQTGAPT-SPIFFIAIAASTTA
42 Megavirus_courdo7                              60.4%   ------------------------------------------------------NSLYGQTGAPT-SPIFFIAIAASTTA
43 Megavirus_ursino                               60.1%   ------------------------------------------------------NSLYGQTGAPT-SPIFFIAIAASTTA
44 Melbournevirus                                 13.3%   ------------------------------------------------------NSGYGALGATQ-GLAPLVEAAAAVTT
45 Micromonas_pusilla_virus_SP1                   16.3%   ------------------------------------------------------NSVYGFTGAGK-GILPCVPIASTTTC
46 Mollivirus_sibericum                            9.9%   ------------------------------------------------------NSVYGINGGQ--SKLACMALARSVTA
47 Molluscum_contagiosum_virus_subtype_1           9.1%   ------------------------------------------------------NSVYGLMGFYN-SSLYSYSSAKCCTT
48 Monkeypox_virus                                10.5%   ------------------------------------------------------NSVYGLMGFRN-SALYSYASAKSCTS
49 Myxoma_virus                                   10.7%   ------------------------------------------------------NSVYGLMGFKN-SALYSYASAKSCTA
50 Noumeavirus                                    12.5%   ------------------------------------------------------NSAYGALGASQ-GIAPLVEAAAAVTT
51 Orf_virus                                      10.2%   ------------------------------------------------------NSVYGLMGFRN-SALFSYASAKSCTA
52 Organic_Lake_phycodnavirus_1                   18.2%   ------------------------------------------------------NSLYGQTGAKT-SSFYEMDVAASTTS
53 Orpheovirus_LCC2                               12.8%   ------------------------------------------------------NSMYGALGAQEGGKMPLPEGAMCVTF
54 Ostreococcus_lucimarinus_virus_1               16.7%   ------------------------------------------------------NSVYGFTGAGK-GILPCVPIASTTTC
55 Ostreococcus_tauri_virus_OtV5                  16.7%   ------------------------------------------------------NSVYGFTGAGK-GILPCVPIASTTTS
56 Pacmanvirus_A23                                 9.1%   ------------------------------------------------------NTFYGESGNFK-SSVYELLVAAGITC
57 Pandoravirus_dulcis                             9.2%   EAQPKITVATQMSSAPYHGTVWCVNVPVEPHLIVVRRIVQRHGVSVPSRAVVVGNSVYGFLGAVKRGRMPCVEVSESVTC
58 Pandoravirus_inopinatum                         8.8%   ------------------------------------------------------NCFPADDHEIL-TETGFMNYAAVMEH
59 Pandoravirus_salinus                           10.5%   EAQPKITVATQMSSAPYHGTVWCVNVPVEPHLIVVRRIVQRYGVSVPSRAVVVGNSVYGFLGAVKRGRMPCVEVSESVTC
60 Paramecium_bursaria_Chlorella_virus_CVK2       15.4%   ------------------------------------------------------NSVYGFLGASK-GFIPCVPIAASVTA
```

```
61 Paramecium_bursaria_chlorella_virus_MT325        15.1%  ------------------------------------------------------NSVYGFLGASK-GFLPCVPIAASVTA
62 Paramecium_bursaria_Chlorella_virus_NYs1         14.2%  ------------------------------------------------------NSVYGFLGASR-GFIPCVPIAASVTA
63 Phaeocystis_globosa_virus                        16.9%  ------------------------------------------------------NSLYGQCGART-SAFYEKDVAASCTA
64 Phaeocystis_globosa_virus_14T                    16.8%  ------------------------------------------------------NSLYGQCGART-SAFYEKDVAASCTA
65 Pithovirus_massiliensis                          11.6%  ------------------------------------------------------NSTYGFTGARQ-GRLPIPQIAASVTA
66 Pithovirus_sibericum                             11.8%  ------------------------------------------------------NSTYGFTGARQ-GRLPIPQIAASVTA
67 Port-miou_virus                                  12.4%  ------------------------------------------------------NSAYGALGASQ-GIAPLVEAAAAVTT
68 Powai_lake_megavirus                             60.0%  ------------------------------------------------------NSLYGQTGAPT-SPIFFIAIAASTTA
69 Scale_drop_disease_virus                         12.8%  ------------------------------------------------------NSVYGSMGTTR-GYLPFMQGAMTTTF
70 Short-finned_eel_ranavirus                       13.1%  ------------------------------------------------------NSMYGAMGVKR-GYLPFQDGAMTVTY
71 Singapore_grouper_iridovirus                     13.3%  ------------------------------------------------------NSMYGAMGVKR-GYLPFQEGAMTVTY
72 Skunkpox_virus                                   11.0%  ------------------------------------------------------NSVYGLMGFRN-SALYSYASAKSCTS
73 Spodoptera_frugiperda_ascovirus_1a               10.9%  ------------------------------------------------------NSIYGSTGASN-GKLPCQNVAKVTTA
74 Testudo_hermanni_ranavirus                       13.2%  ------------------------------------------------------NSMYGAMGVKR-GYLPFQDGAMTVTY
75 Tiger_frog_virus                                 13.1%  ------------------------------------------------------NSMYGAMGVKR-GYLPFQDGAMTVTY
76 Tokyovirus_A1                                    12.9%  ------------------------------------------------------NSGYGALGATQ-GLAPLVEAAAAVTT
77 Trichoplusia_ni_ascovirus_2c                     10.4%  ------------------------------------------------------NSIYGATGASN-GKLPCKNVAKAITA
78 Tunisvirus_fontaine2                             12.2%  ------------------------------------------------------NSAYGALGATQ-GISPLVEAAAAVTT
79 Vaccinia_virus                                   10.6%  ------------------------------------------------------NSVYGLMGFRN-SALYSYASAKSCTS
80 Variola_virus                                    10.3%  ------------------------------------------------------NSVYGLMGFRN-SALYSYASAKSCTS
81 Volepox_virus                                    10.8%  ------------------------------------------------------NSVYGLMGFRN-SALYSYASAKSCTS
82 Wiseana_iridescent_virus                         14.4%  ------------------------------------------------------NSMYGITGVKA-GMLPFMPVAMTITY
83 Yaba_monkey_tumor_virus                          10.8%  ------------------------------------------------------NSVYGLMGFRN-SVLYSYASAKTCTA
84 Yellowstone_lake_phycodnavirus_1                 17.0%  ------------------------------------------------------NSIYGFTGASK-GMLPLVAIASTVTM
   consensus/100%                                          ................................................................................
   consensus/90%                                                                                                 NohYG..G..t u.h.....A.shT.
   consensus/80%                                                                                                 NShYG.hGs.p uhh..h.hAtssTh
   consensus/70%                                                                                                 NShYG.hGspp uhh..hthAtssTs

                                                     2241         :         .         .         .         .         3         .         . 23
 1 Acanthamoeba_castellanii_mamavirus              100.0%  IGRERL-------------------------------HYAKKT-VEDNF-PGS---------------------------
 2 Acanthamoeba_polyphaga_mimivirus                 98.6%  IGRERL-------------------------------HYAKKT-VEDNF-PGS---------------------------
 3 Acanthamoeba_polyphaga_moumouvirus               56.0%  IGRERL-------------------------------YYARKM-VEDNF-PGS---------------------------
 4 Amsacta_moorei_entomopoxvirus                    10.1%  QGQNCI-------------------------------KYIQTLVNNSKYIDNV---------------------------
 5 Anomala_cuprea_entomopoxvirus                     9.7%  LSKKSI-------------------------------MYIHEI-LDKSIYKSN------------------ILTI-NSCN
 6 ASFV_BAV71                                        9.7%  SGQYNL-------------------------------KLVYNFVINKGY-------------------------------
 7 ASFV_E75                                          9.7%  SGQYNL-------------------------------KLVYNFVINKGY-------------------------------
 8 Brazilian_marseillevirus                         12.6%  AGRQDI-------------------------------VKVVDR-ILERW-PEG---------------------------
 9 Cafeteria_roenbergensis_virus_BV-PW1             20.9%  TGREML-------------------------------IYAKKY--DEEILPWI---------INGLQKAYQTNDTDTIEY
10 Cannes_8_virus                                   13.3%  AGRQDI-------------------------------QKVVQM-ILERW-PEG---------------------------
11 Catovirus_CTV1                                   24.3%  TGKEHL-------------------------------EFSRDF-LEGLY-NNMINLALTDKKEYINLCKKEFSKISDNKF
12 Cedratvirus_A11                                  12.1%  MGRKHI-------------------------------LEANDY-ISQKYGY-----------------------------
13 Choristoneura_biennis_entomopoxvirus             10.5%  LGQKCI-------------------------------KYIKNLVDKSRYIDNN---------------------------
14 Common_midwife_toad_ranavirus                    13.2%  LGRQCI-------------------------------EKAAS--IIGSE-HGG---------------------------
15 Cowpox_virus                                     10.5%  IGRRMI-------------------------------LYLESV-LNGAELSNG----------------------MLRFA
16 Diadromus_pulchellus_ascovirus_4a                12.7%  TGRQSI-------------------------------IKASN--IIKSL--GG---------------------------
17 Ectromelia_virus                                 10.5%  IGRRMI-------------------------------LYLESV-LNGAELSDG----------------------MLRFA
18 Emiliania_huxleyi_virus_145                      14.6%  NGRKMI-------------------------------NKTSEM-AIELFKPWI---------------------------
19 Emiliania_huxleyi_virus_86                       14.6%  NGRKMI-------------------------------NKTSEM-AIELFKPWI---------------------------
20 European_catfish_virus                           13.1%  FGRQCI-------------------------------EKAAS--IIGSE-HGG---------------------------
21 Faustovirus_D3                                    8.8%  QGQLEI-------------------------------KFVSDGVIVNGF-------------------------------
22 Faustovirus_E12                                   8.9%  QGQLEI-------------------------------KFVSDGVIINGF-------------------------------
23 Faustovirus_E24                                   8.9%  QGQLEI-------------------------------KFVSDGVIINGF-------------------------------
24 Faustovirus_E9                                    9.0%  QGQLEI-------------------------------KFVSDGVIVNGF-------------------------------
25 Faustovirus_Liban                                 9.0%  QGQLEI-------------------------------KFVSDGVIINGF-------------------------------
26 Feldmannia_species_virus                         13.4%  TGRSMI-------------------------------KTTADF-IRTEM--GG---------------------------
27 Frog_virus_3                                     13.2%  FGRQCI-------------------------------EKAAS--IIGSE-HGG---------------------------
28 Heliothis_virescens_ascovirus_3e                 11.3%  LGRKTI-------------------------------LQSIDIAQRDRS-------------------------------
29 Hokovirus_HKV1                                   20.3%  TGREQLLGAKTFVEKVFAELATCAFDNNFANYKERINKLFDDNLCDNELLFCD-------------YNLNVNAFNDYSKD
30 Infectious_spleen_and_kidney_necrosis_virus      12.7%  CGRKLI-------------------------------EKAAH--LLKTV-VGA---------------------------
31 Insectomime_virus                                12.3%  AGRQDI-------------------------------VKVVER-ILERW-PEG---------------------------
32 Invertebrate_iridescent_virus_22                 13.4%  KGREGV-------------------------------KQVSDI-LQKQY--GG---------------------------
33 Invertebrate_iridescent_virus_3                  14.2%  MGRTNI-------------------------------ARVAQL-LQTQY--QG---------------------------
34 Kaumoebavirus                                    10.2%  RGRTYL-------------------------------KTMYRYLIDEHH-------------------------------
35 Klosneuvirus_KNV1                                15.8%
36 Kurlavirus_BKC-1                                 12.6%  AGRQDI-------------------------------MKVVER-ILERW-PEG---------------------------
37 Lausannevirus                                    12.4%  AGRQDI-------------------------------MKVVER-ILERW-PDG---------------------------
38 Lumpy_skin_disease_virus_NI-2490                 10.5%  IGRKMI-------------------------------DYLNSV-LNGSKVENG----------------------KVLLA
39 Lymphocystis_disease_virus                       13.5%  VGRLSI-------------------------------EKAAAV-IESKY--GG---------------------------
40 Marseillevirus_marseillevirus_T19                13.3%  AGRQDI-------------------------------QKVVQM-ILERW-PEG---------------------------
41 Megavirus_chiliensis                             60.1%  IGRERL-------------------------------HYARKM-VEDNF-PGS---------------------------
42 Megavirus_courdo7                                60.4%  IGRERL-------------------------------HYARKM-VEDNF-PGS---------------------------
43 Megavirus_ursino                                 60.1%  IGRERL-------------------------------HYARKM-VEDNF-PGS---------------------------
44 Melbournevirus                                   13.3%  AGRQDI-------------------------------QKVVQM-ILERW-PEG---------------------------
45 Micromonas_pusilla_virus_SP1                     16.3%  RGRGMI-------------------------------DETKKY-VEENF-PGA---------------------------
46 Mollivirus_sibericum                              9.9%  DGRNLIKTTRYYVENHMVHYVHGTPQCEVYFHKNFMALPEPDP-VTGAYAAADVWNCEPLAAYRTPDDGLEGCELEVVPS
47 Molluscum_contagiosum_virus_subtype_1             9.1%  IGRVMI-------------------------------TYLDRV-VDGATLCAG----------------------VLRLA
48 Monkeypox_virus                                  10.5%  IGRRMI-------------------------------LYLESV-LNGAELSNG----------------------MLRFA
49 Myxoma_virus                                     10.7%  IGRQMI-------------------------------HYLNSV-LNGCKLSSG----------------------KFQLA
50 Noumeavirus                                      12.5%  AGRQDI-------------------------------MKVVER-ILERW-PEG---------------------------
51 Orf_virus                                        10.2%  IGRTMI-------------------------------AYLERT-LDGASVCGT----------------------RLSLA
52 Organic_Lake_phycodnavirus_1                     18.2%  VGRTLL-------------------------------IYAKE--IIENVYGNT---------------------------
53 Orpheovirus_LCC2                                 12.8%  KGRELI-------------------------------GSVGSY-LVKKY--NA---------------------------
54 Ostreococcus_lucimarinus_virus_1                 16.7%  KGRSMI-------------------------------EETKNY-VEANF-PGA---------------------------
55 Ostreococcus_tauri_virus_OtV5                    16.7%  KGRSMI-------------------------------EETKNY-VEKNF-PGA---------------------------
56 Pacmanvirus_A23                                   9.1%  AGQANI-------------------------------KRVAGFVQNKGF-------------------------------
57 Pandoravirus_dulcis                               9.2%  IGRDMINATKAYVETHLPRYVGGLLDDPALVAAAAERQRAKER-LDAGHATAD---------------------LSAVTL
58 Pandoravirus_inopinatum                           8.8%  FKHHPR-------------------------------LSVACY-VDGQLQYHD---------------------------
59 Pandoravirus_salinus                             10.5%  IGRDMINATKAYVETHLPRYVSGLLDDPALVAAAAERQRAKER-LDASFASAG--------LGAVAVTDVALAQPALEPA
60 Paramecium_bursaria_Chlorella_virus_CVK2         15.4%  TGRKMI-------------------------------EHTAKR-AVELL-PGS---------------------------
61 Paramecium_bursaria_chlorella_virus_MT325        15.1%  TGRNMI-------------------------------DVASRR-AIELL-PGS---------------------------
62 Paramecium_bursaria_Chlorella_virus_NYs1         14.2%  TGRKMI-------------------------------EHTSKR-VTELL-PGS---------------------------
63 Phaeocystis_globosa_virus                        16.9%  IGRKLL-------------------------------FYGKDV-IEGCY-NNV---------------------------
64 Phaeocystis_globosa_virus_14T                    16.8%  IGRKLL-------------------------------FYGKDV-IEGCY-NNV---------------------------
65 Pithovirus_massiliensis                          11.6%  MGRKLI-------------------------------HQGNDY-LKEKYNMDA---------------------------
66 Pithovirus_sibericum                             11.8%  MGRKLI-------------------------------HQGNDY-LKEKY--GM---------------------------
67 Port-miou_virus                                  12.4%  AGRQDI-------------------------------MKVVER-ILERW-PDG---------------------------
68 Powai_lake_megavirus                             60.0%  IGRERL-------------------------------HYARKM-VEDNF-PGS---------------------------
69 Scale_drop_disease_virus                         12.8%  CGRKMI-------------------------------ERAAE--LLKTV-ANA---------------------------
70 Short-finned_eel_ranavirus                       13.1%  FGRQCI-------------------------------EKAAS--IIGSE-HGG---------------------------
71 Singapore_grouper_iridovirus                     13.3%  LGRQCI-------------------------------ETAAT--LIGSE-HGG---------------------------
72 Skunkpox_virus                                   11.0%  IGRRMI-------------------------------LYLESV-LNGSVLSNG----------------------ILHFA
73 Spodoptera_frugiperda_ascovirus_1a               10.9%  LGRMTI-------------------------------LQAIE--IARTE-RNV---------------------------
74 Testudo_hermanni_ranavirus                       13.2%  FGRQCI-------------------------------EKAAS--IIGSE-HGG---------------------------
```

```
75 Tiger_frog_virus                          13.1%   FGRQCI-------------------------------EKAAS--IIGSE-HGG---------------------------
76 Tokyovirus_A1                             12.9%   AGRQDI-------------------------------QKVVQM-ILERW-PDG---------------------------
77 Trichoplusia_ni_ascovirus_2c              10.4%   LGRKVI-------------------------------KESIQMASDKGI-------------------------------
78 Tunisvirus_fontaine2                      12.2%   AGRQDI-------------------------------VKVVER-ILERW-PEG---------------------------
79 Vaccinia_virus                            10.6%   IGRRMI-------------------------------LYLESV-LNGAELSNG----------------------MLRFA
80 Variola_virus                             10.3%   IGRRMI-------------------------------LYLESV-LNGAELSNG----------------------MLRFA
81 Volepox_virus                             10.8%   IGRRMI-------------------------------LYLESV-LNGSVLSNG----------------------ILHFA
82 Wiseana_iridescent_virus                  14.4%   MGRKSV-------------------------------LKAAKY---GKEKFNG---------------------------
83 Yaba_monkey_tumor_virus                   10.8%   IGRMMI-------------------------------LYLNSV-LDGSKINSG----------------------KFTFA
84 Yellowstone_lake_phycodnavirus_1          17.0%   RGRQMI-------------------------------EETKTY-VEENF-PGA---------------------------
   consensus/100%                                    ................................................................................
   consensus/90%                                     .Gp..l                               ..h.p..h.tt.....                      .....
   consensus/80%                                     hGRp.l                               .hh.ph l.tt...t.                      .....
   consensus/70%                                     hGRp.I                               .hstph l.tph..ts

                                                 2321         .         .         :         .         .         .        4 24
 1 Acanthamoeba_castellanii_mamavirus       100.0%   ----------------------------------------------------------------EVIYGDSVTGDTPIIT
 2 Acanthamoeba_polyphaga_mimivirus          98.6%   ----------------------------------------------------------------EVIYGDSVTGDTPIIT
 3 Acanthamoeba_polyphaga_moumouvirus        56.0%   ----------------------------------------------------------------EVIYGDSVTGDTPIIV
 4 Amsacta_moorei_entomopoxvirus             10.1%   ---LILNKCNNPFTNEPIKTNYPGN--------------------------------LNVNFTFNVKYG-----------
 5 Anomala_cuprea_entomopoxvirus              9.7%   NIFTQK----------LIKTKYEGN--------------------------------INEDFIIDIVYG-----------
 6 ASFV_BAV71                                 9.7%   ----------------------------------------------------------------GIKYG-----------
 7 ASFV_E75                                   9.7%   ----------------------------------------------------------------GIKYG-----------
 8 Brazilian_marseillevirus                  12.6%   ----------------------------------------------------------------ILVYG-----------
 9 Cafeteria_roenbergensis_virus_BV-PW1      20.9%   LYNKELKDPANEKLKEQIKDFCLKT--------------------------------NDYVFNPIIKYGDSVIGDTPLLL
10 Cannes_8_virus                            13.3%   ----------------------------------------------------------------TLVYG-----------
11 Catovirus_CTV1                            24.3%   VQPKNGFVNREQFYDAFYNKVSKML--------------------------------ADHRTKINVIYGDSVTGETPLLL
12 Cedratvirus_A11                           12.1%   ----------------------------------------------------------------KVVYN-----------
13 Choristoneura_biennis_entomopoxvirus      10.5%   ---LYLNEQNNPFSNEPVITRYSGN--------------------------------LDVNFTFYIIYG-----------
14 Common_midwife_toad_ranavirus             13.2%   ----------------------------------------------------------------QLVYG-----------
15 Cowpox_virus                              10.5%   NPLSNPFYMDDRDINPIVKTSLP----------------------------------IDYRFRFRSVYG-----------
16 Diadromus_pulchellus_ascovirus_4a         12.7%   ----------------------------------------------------------------TIVYG-----------
17 Ectromelia_virus                          10.5%   NPLSNPFYMDDRDINPIVKTSLS----------------------------------IDYRFRFRSVYG-----------
18 Emiliania_huxleyi_virus_145               14.6%   ---------------------------------------------------------------AEIIYG-----------
19 Emiliania_huxleyi_virus_86                14.6%   ---------------------------------------------------------------AEIIYG-----------
20 European_catfish_virus                    13.1%   ----------------------------------------------------------------QLVYG-----------
21 Faustovirus_D3                             8.8%   ----------------------------------------------------------------KLYYG-----------
22 Faustovirus_E12                            8.9%   ----------------------------------------------------------------KLYYG-----------
23 Faustovirus_E24                            8.9%   ----------------------------------------------------------------KLYYG-----------
24 Faustovirus_E9                             9.0%   ----------------------------------------------------------------KLYYG-----------
25 Faustovirus_Liban                          9.0%   ----------------------------------------------------------------KLYYG-----------
26 Feldmannia_species_virus                  13.4%   ----------------------------------------------------------------TVIYG-----------
27 Frog_virus_3                              13.2%   ----------------------------------------------------------------QLVYG-----------
28 Heliothis_virescens_ascovirus_3e          11.3%   ---------------------------------------------------------------VSTIYS-----------
29 Hokovirus_HKV1                            20.3%   KKYTNREEFIEWTFNEFVNILNYNN--------------------------------IKYSIKPICIYG-----------
30 Infectious_spleen_and_kidney_necrosis_virus 12.7% ----------------------------------------------------------------TIVYG-----------
31 Insectomime_virus                         12.3%   ----------------------------------------------------------------VLVYG-----------
32 Invertebrate_iridescent_virus_22          13.4%   ----------------------------------------------------------------ELVYG-----------
33 Invertebrate_iridescent_virus_3           14.2%   ----------------------------------------------------------------ELVYG-----------
34 Kaumoebavirus                             10.2%   ---------------------------------------------------------------CKIQYG-----------
35 Klosneuvirus_KNV1                         15.8%   --------------------------------------------------------------------------------
36 Kurlavirus_BKC-1                          12.6%   ----------------------------------------------------------------VLVYG-----------
37 Lausannevirus                             12.4%   ----------------------------------------------------------------VLVYG-----------
38 Lumpy_skin_disease_virus_NI-2490          10.5%   TKPINPFFEDGRNVDINIDTKLP----------------------------------TEYTFNFKSVYG-----------
39 Lymphocystis_disease_virus                13.5%   ----------------------------------------------------------------ALVYG-----------
40 Marseillevirus_marseillevirus_T19         13.3%   ----------------------------------------------------------------TLVYG-----------
41 Megavirus_chiliensis                      60.1%   ----------------------------------------------------------------EVIYGDSVTGDTPIMI
42 Megavirus_courdo7                         60.4%   ----------------------------------------------------------------EVIYGDSVTGDTPIMI
43 Megavirus_ursino                          60.1%   ----------------------------------------------------------------EVIYGDSVTGDTPIMT
44 Melbournevirus                            13.3%   ----------------------------------------------------------------TLVYG-----------
45 Micromonas_pusilla_virus_SP1              16.3%   ----------------------------------------------------------------KVRYG-----------
46 Mollivirus_sibericum                       9.9%   SQVKPWDEVVEPCLRLATDRKTGAKFVDALILPISAIYDPEKRARLHPGLVALLERLVAVVASARVIYG-----------
47 Molluscum_contagiosum_virus_subtype_1      9.1%   AEPRNPLLASAPPRARDVDSAALG---------------------------------HELELRFRSVYG-----------
48 Monkeypox_virus                           10.5%   NTLSNPFYMDDRDINPIVKTSLP----------------------------------IDYRFRFRSVYG-----------
49 Myxoma_virus                              10.7%   AYPINPFFQDDRSVDLYASTNLP----------------------------------TEYNYDFKGVYG-----------
50 Noumeavirus                               12.5%   ----------------------------------------------------------------VLVYG-----------
51 Orf_virus                                 10.2%   AAPDNPLLRDEAFAGRAAELEIDAAVAGE----------------------------RTERVGFRSVYG-----------
52 Organic_Lake_phycodnavirus_1              18.2%   -------------------IVDTKY--------------------------------GKMKSNAEYIYG-----------
53 Orpheovirus_LCC2                          12.8%   ----------------------------------------------------------------KIIYG-----------
54 Ostreococcus_lucimarinus_virus_1          16.7%   ----------------------------------------------------------------KVRYG-----------
55 Ostreococcus_tauri_virus_OtV5             16.7%   ----------------------------------------------------------------KVRYG-----------
56 Pacmanvirus_A23                            9.1%   ----------------------------------------------------------------IVHYG-----------
57 Pandoravirus_dulcis                        9.2%   SPPKDDRSDREKNREALLDALAAAGVT------------------------------PCDITGATVVYG-----------
58 Pandoravirus_inopinatum                    8.8%   ------------------------------------------------------------ITESNVVAN-----------
59 Pandoravirus_salinus                      10.5%   AKDDRSEREKNRDALLDALTSASVK--------------------------------PGDITGATVVYGDSVGADTPLLL
60 Paramecium_bursaria_Chlorella_virus_CVK2  15.4%   ----------------------------------------------------------------EVIYG-----------
61 Paramecium_bursaria_chlorella_virus_MT325 15.1%   ----------------------------------------------------------------EVIYG-----------
62 Paramecium_bursaria_Chlorella_virus_NYs1  14.2%   ----------------------------------------------------------------EVIYG-----------
63 Phaeocystis_globosa_virus                 16.9%   ------------------EITLSDG--------------------------------VKVVTKAECVYG-----------
64 Phaeocystis_globosa_virus_14T             16.8%   ------------------EITLSDG--------------------------------VKVVTKAECVYG-----------
65 Pithovirus_massiliensis                   11.6%   ------------------------------------------------------------------VYN-----------
66 Pithovirus_sibericum                      11.8%   ----------------------------------------------------------------DAVYN-----------
67 Port-miou_virus                           12.4%   ----------------------------------------------------------------VLVYG-----------
68 Powai_lake_megavirus                      60.0%   ----------------------------------------------------------------EVIYGDSVTGDTPIMI
69 Scale_drop_disease_virus                  12.8%   ----------------------------------------------------------------RLIYG-----------
70 Short-finned_eel_ranavirus                13.1%   ----------------------------------------------------------------QLVYG-----------
71 Singapore_grouper_iridovirus              13.3%   ----------------------------------------------------------------TLVYG-----------
72 Skunkpox_virus                            11.0%   NPLSNPFYMDDRDVNPIVKTSLP----------------------------------IEYEFRFRSVYG-----------
73 Spodoptera_frugiperda_ascovirus_1a        10.9%   ----------------------------------------------------------------TTIYS-----------
74 Testudo_hermanni_ranavirus                13.2%   ----------------------------------------------------------------QLVYG-----------
75 Tiger_frog_virus                          13.1%   ----------------------------------------------------------------QLVYG-----------
76 Tokyovirus_A1                             12.9%   ----------------------------------------------------------------TLVYG-----------
77 Trichoplusia_ni_ascovirus_2c              10.4%   ----------------------------------------------------------------PVIYG-----------
78 Tunisvirus_fontaine2                      12.2%   ----------------------------------------------------------------VLVYG-----------
79 Vaccinia_virus                            10.6%   NPLSNPFYMDDRDINPIVKTSLP----------------------------------IDYRFRFRSVYG-----------
80 Variola_virus                             10.3%   NPLSNPFYMDDRDINPIVKTSLP----------------------------------IDYRFRFRSVYG-----------
81 Volepox_virus                             10.8%   NPLSNPFYIDDRDVNPIVKTSLP----------------------------------IDYEFHFRSVYG-----------
82 Wiseana_iridescent_virus                  14.4%   ----------------------------------------------------------------LLVAS-----------
83 Yaba_monkey_tumor_virus                   10.8%   KLPLNPFFEDGRYVTLNTDTGVD----------------------------------KHYNFVFKTVYG-----------
84 Yellowstone_lake_phycodnavirus_1          17.0%   ----------------------------------------------------------------KVRYG-----------
   consensus/100%                                    ................................................................................
   consensus/90%                                     .........................                                .........hYG...........
   consensus/80%                                     .......................                                  .......thlYG
   consensus/70%                                                                                                    .pllYG
```

```
                                                        2401     .         .         .         .         :         .         .         . 24
 1 Acanthamoeba_castellanii_mamavirus            100.0%  RHQNGDINITTIEELGSKWKPYEIF----------------------------------------------------KAH
 2 Acanthamoeba_polyphaga_mimivirus               98.6%  RHQNGDINITTIEELGSKWKPYEIF----------------------------------------------------KAH
 3 Acanthamoeba_polyphaga_moumouvirus             56.0%  KLPNSNDVEIKTIQELTTFW-------------------------------------------------------YEYDA
 4 Amsacta_moorei_entomopoxvirus                  10.1%  --------------------------------------------------------------------------------
 5 Anomala_cuprea_entomopoxvirus                   9.7%  --------------------------------------------------------------------------------
 6 ASFV_BAV71                                      9.7%  --------------------------------------------------------------------------------
 7 ASFV_E75                                        9.7%  --------------------------------------------------------------------------------
 8 Brazilian_marseillevirus                       12.6%  --------------------------------------------------------------------------------
 9 Cafeteria_roenbergensis_virus_BV-PW1           20.9%  KNKFTNEILINKIKDLSSNWSNYHNGKESCEIDTYQTWTETGWTDIKRVIRHKLESNKKLLKIQTHNGEVIVTDEHSLLN
10 Cannes_8_virus                                 13.3%  --------------------------------------------------------------------------------
11 Catovirus_CTV1                                 24.3%  KNPTTNKIVIKRIDELGTEWKNYNNYKSSDSNRYFRELLTILFKNKNVEKKRENFVPVSQWMYIKNNKYRYINDAYSNNT
12 Cedratvirus_A11                                12.1%  --------------------------------------------------------------------------------
13 Choristoneura_biennis_entomopoxvirus           10.5%  --------------------------------------------------------------------------------
14 Common_midwife_toad_ranavirus                  13.2%  --------------------------------------------------------------------------------
15 Cowpox_virus                                   10.5%  --------------------------------------------------------------------------------
16 Diadromus_pulchellus_ascovirus_4a              12.7%  --------------------------------------------------------------------------------
17 Ectromelia_virus                               10.5%  --------------------------------------------------------------------------------
18 Emiliania_huxleyi_virus_145                    14.6%  --------------------------------------------------------------------------------
19 Emiliania_huxleyi_virus_86                     14.6%  --------------------------------------------------------------------------------
20 European_catfish_virus                         13.1%  --------------------------------------------------------------------------------
21 Faustovirus_D3                                  8.8%  --------------------------------------------------------------------------------
22 Faustovirus_E12                                 8.9%  --------------------------------------------------------------------------------
23 Faustovirus_E24                                 8.9%  --------------------------------------------------------------------------------
24 Faustovirus_E9                                  9.0%  --------------------------------------------------------------------------------
25 Faustovirus_Liban                               9.0%  --------------------------------------------------------------------------------
26 Feldmannia_species_virus                       13.4%  --------------------------------------------------------------------------------
27 Frog_virus_3                                   13.2%  --------------------------------------------------------------------------------
28 Heliothis_virescens_ascovirus_3e               11.3%  --------------------------------------------------------------------------------
29 Hokovirus_HKV1                                 20.3%  --------------------------------------------------------------------------------
30 Infectious_spleen_and_kidney_necrosis_virus    12.7%  --------------------------------------------------------------------------------
31 Insectomime_virus                              12.3%  --------------------------------------------------------------------------------
32 Invertebrate_iridescent_virus_22               13.4%  --------------------------------------------------------------------------------
33 Invertebrate_iridescent_virus_3                14.2%  --------------------------------------------------------------------------------
34 Kaumoebavirus                                  10.2%  --------------------------------------------------------------------------------
35 Klosneuvirus_KNV1                              15.8%  --------------------------------------------------------------------------------
36 Kurlavirus_BKC-1                               12.6%  --------------------------------------------------------------------------------
37 Lausannevirus                                  12.4%  --------------------------------------------------------------------------------
38 Lumpy_skin_disease_virus_NI-2490               10.5%  --------------------------------------------------------------------------------
39 Lymphocystis_disease_virus                     13.5%  --------------------------------------------------------------------------------
40 Marseillevirus_marseillevirus_T19              13.3%  --------------------------------------------------------------------------------
41 Megavirus_chiliensis                           60.1%  KDKNNNINIVTIKELGEK------------------------------------------------------WKPYDIFK
42 Megavirus_courdo7                              60.4%  KDKNNNINIVTIKELGEK------------------------------------------------------WKPYDIFK
43 Megavirus_ursino                               60.1%  KDKNNNINIITIKELGEQ------------------------------------------------------WKPYDIFK
44 Melbournevirus                                 13.3%  --------------------------------------------------------------------------------
45 Micromonas_pusilla_virus_SP1                   16.3%  --------------------------------------------------------------------------------
46 Mollivirus_sibericum                            9.9%  --------------------------------------------------------------------------------
47 Molluscum_contagiosum_virus_subtype_1           9.1%  --------------------------------------------------------------------------------
48 Monkeypox_virus                                10.5%  --------------------------------------------------------------------------------
49 Myxoma_virus                                   10.7%  --------------------------------------------------------------------------------
50 Noumeavirus                                    12.5%  --------------------------------------------------------------------------------
51 Orf_virus                                      10.2%  --------------------------------------------------------------------------------
52 Organic_Lake_phycodnavirus_1                   18.2%  --------------------------------------------------------------------------------
53 Orpheovirus_LCC2                               12.8%  --------------------------------------------------------------------------------
54 Ostreococcus_lucimarinus_virus_1               16.7%  --------------------------------------------------------------------------------
55 Ostreococcus_tauri_virus_OtV5                  16.7%  --------------------------------------------------------------------------------
56 Pacmanvirus_A23                                 9.1%  --------------------------------------------------------------------------------
57 Pandoravirus_dulcis                             9.2%  --------------------------------------------------------------------------------
58 Pandoravirus_inopinatum                         8.8%  --------------------------------------------------------------------------------
59 Pandoravirus_salinus                           10.5%  RFDGKHVDYVRADQVDGILARGDTSAAASLWSAYQGDKEAFCLARPVEVWTERGWTAVN-------------RVIRHRAG
60 Paramecium_bursaria_Chlorella_virus_CVK2       15.4%  --------------------------------------------------------------------------------
61 Paramecium_bursaria_chlorella_virus_MT325      15.1%  --------------------------------------------------------------------------------
62 Paramecium_bursaria_Chlorella_virus_NYs1       14.2%  --------------------------------------------------------------------------------
63 Phaeocystis_globosa_virus                      16.9%  --------------------------------------------------------------------------------
64 Phaeocystis_globosa_virus_14T                  16.8%  --------------------------------------------------------------------------------
65 Pithovirus_massiliensis                        11.6%  --------------------------------------------------------------------------------
66 Pithovirus_sibericum                           11.8%  --------------------------------------------------------------------------------
67 Port-miou_virus                                12.4%  --------------------------------------------------------------------------------
68 Powai_lake_megavirus                           60.0%  KDKNNNINIVTIKELGEQ------------------------------------------------------WKPYDIFK
69 Scale_drop_disease_virus                       12.8%  --------------------------------------------------------------------------------
70 Short-finned_eel_ranavirus                     13.1%  --------------------------------------------------------------------------------
71 Singapore_grouper_iridovirus                   13.3%  --------------------------------------------------------------------------------
72 Skunkpox_virus                                 11.0%  --------------------------------------------------------------------------------
73 Spodoptera_frugiperda_ascovirus_1a             10.9%  --------------------------------------------------------------------------------
74 Testudo_hermanni_ranavirus                     13.2%  --------------------------------------------------------------------------------
75 Tiger_frog_virus                               13.1%  --------------------------------------------------------------------------------
76 Tokyovirus_A1                                  12.9%  --------------------------------------------------------------------------------
77 Trichoplusia_ni_ascovirus_2c                   10.4%  --------------------------------------------------------------------------------
78 Tunisvirus_fontaine2                           12.2%  --------------------------------------------------------------------------------
79 Vaccinia_virus                                 10.6%  --------------------------------------------------------------------------------
80 Variola_virus                                  10.3%  --------------------------------------------------------------------------------
81 Volepox_virus                                  10.8%  --------------------------------------------------------------------------------
82 Wiseana_iridescent_virus                       14.4%  --------------------------------------------------------------------------------
83 Yaba_monkey_tumor_virus                        10.8%  --------------------------------------------------------------------------------
84 Yellowstone_lake_phycodnavirus_1               17.0%  --------------------------------------------------------------------------------
   consensus/100%                                        ................................................................................
   consensus/90%                                         ..................                                                           ...
   consensus/80%
   consensus/70%

                                                        2481     .         5         .         .         .         .         :         . 25
 1 Acanthamoeba_castellanii_mamavirus            100.0%  EKNSNRKFKQQSQYPTDSEVWTAKGWAKIKRVIRHKTVKKIYRVLTHTGCIDVTEDHSLLDPNQNIIKPINCQIGTELLH
 2 Acanthamoeba_polyphaga_mimivirus               98.6%  EKNSNRKFKQQSQYPTDSEVWTAKGWAKIKRVIRHKTVKKIYRVLTHTGCIDVTEDHSLLDPNQNIIKPINCQIGTELLH
 3 Acanthamoeba_polyphaga_moumouvirus             56.0%  FKAGDSNRKDKQQGILDYEVWTDKGWAKIKRVIRHQTKKSIYRVKTDNGVVDVTEDHSLLNTDKEIIKPLDCNSNTKLLH
 4 Amsacta_moorei_entomopoxvirus                  10.1%  --------------------------------------------------------------------------------
 5 Anomala_cuprea_entomopoxvirus                   9.7%  --------------------------------------------------------------------------------
 6 ASFV_BAV71                                      9.7%  --------------------------------------------------------------------------------
 7 ASFV_E75                                        9.7%  --------------------------------------------------------------------------------
 8 Brazilian_marseillevirus                       12.6%  --------------------------------------------------------------------------------
 9 Cafeteria_roenbergensis_virus_BV-PW1           20.9%  KNGKTINAKNVKVGDNILHSFPSYINNIDNTNSINYHNKFYNKKMCNELAYILGCFMKYGLCDSSKKCFTINNKDINLIE
10 Cannes_8_virus                                 13.3%  --------------------------------------------------------------------------------
11 Catovirus_CTV1                                 24.3%  YLQVNLNNNKNMICDVEDIDIINCELKNISKMILNKIIKLFPKQIQAKLQKYEVNYINENSLDNRKCNLSIDVDNQTIDK
12 Cedratvirus_A11                                12.1%
```

```
13 Choristoneura_biennis_entomopoxvirus              10.5%   --------------------------------------------------------------------------------
14 Common_midwife_toad_ranavirus                     13.2%   --------------------------------------------------------------------------------
15 Cowpox_virus                                      10.5%   --------------------------------------------------------------------------------
16 Diadromus_pulchellus_ascovirus_4a                 12.7%   --------------------------------------------------------------------------------
17 Ectromelia_virus                                  10.5%   --------------------------------------------------------------------------------
18 Emiliania_huxleyi_virus_145                       14.6%   --------------------------------------------------------------------------------
19 Emiliania_huxleyi_virus_86                        14.6%   --------------------------------------------------------------------------------
20 European_catfish_virus                            13.1%   --------------------------------------------------------------------------------
21 Faustovirus_D3                                     8.8%   --------------------------------------------------------------------------------
22 Faustovirus_E12                                    8.9%   --------------------------------------------------------------------------------
23 Faustovirus_E24                                    8.9%   --------------------------------------------------------------------------------
24 Faustovirus_E9                                     9.0%   --------------------------------------------------------------------------------
25 Faustovirus_Liban                                  9.0%   --------------------------------------------------------------------------------
26 Feldmannia_species_virus                          13.4%   --------------------------------------------------------------------------------
27 Frog_virus_3                                      13.2%   --------------------------------------------------------------------------------
28 Heliothis_virescens_ascovirus_3e                  11.3%   --------------------------------------------------------------------------------
29 Hokovirus_HKV1                                    20.3%   --------------------------------------------------------------------------------
30 Infectious_spleen_and_kidney_necrosis_virus       12.7%   --------------------------------------------------------------------------------
31 Insectomime_virus                                 12.3%   --------------------------------------------------------------------------------
32 Invertebrate_iridescent_virus_22                  13.4%   --------------------------------------------------------------------------------
33 Invertebrate_iridescent_virus_3                   14.2%   --------------------------------------------------------------------------------
34 Kaumoebavirus                                     10.2%   --------------------------------------------------------------------------------
35 Klosneuvirus_KNV1                                 15.8%   --------------------------------------------------------------------------------
36 Kurlavirus_BKC-1                                  12.6%   --------------------------------------------------------------------------------
37 Lausannevirus                                     12.4%   --------------------------------------------------------------------------------
38 Lumpy_skin_disease_virus_NI-2490                  10.5%   --------------------------------------------------------------------------------
39 Lymphocystis_disease_virus                        13.5%   --------------------------------------------------------------------------------
40 Marseillevirus_marseillevirus_T19                 13.3%   --------------------------------------------------------------------------------
41 Megavirus_chiliensis                              60.1%   SHEINSNRKYKQQADFNGEVWTSNGWAKIKRVIRHKTVKKLYRVLTNTGCIDVTEDHSLLDTNKNIIKPIDCKIGTELLH
42 Megavirus_courdo7                                 60.4%   SHEINSNRKYKQQADFNGEVWTSNGWAKIKRVIRHKTVKKLYRVLTNTGCIDVTEDHSLLDTNKNIIKPIDCKIGTELLH
43 Megavirus_ursino                                  60.1%   SHDINSNRKYKQQADFNGEVWTSNGWVKIKRVIRHKTVKKLYRVLTNTGCIDVTEDHSLLDTNKNIIKPMNCKIGTELLH
44 Melbournevirus                                    13.3%   --------------------------------------------------------------------------------
45 Micromonas_pusilla_virus_SP1                      16.3%   --------------------------------------------------------------------------------
46 Mollivirus_sibericum                               9.9%   --------------------------------------------------------------------------------
47 Molluscum_contagiosum_virus_subtype_1              9.1%   --------------------------------------------------------------------------------
48 Monkeypox_virus                                   10.5%   --------------------------------------------------------------------------------
49 Myxoma_virus                                      10.7%   --------------------------------------------------------------------------------
50 Noumeavirus                                       12.5%   --------------------------------------------------------------------------------
51 Orf_virus                                         10.2%   --------------------------------------------------------------------------------
52 Organic_Lake_phycodnavirus_1                      18.2%   --------------------------------------------------------------------------------
53 Orpheovirus_LCC2                                  12.8%   --------------------------------------------------------------------------------
54 Ostreococcus_lucimarinus_virus_1                  16.7%   --------------------------------------------------------------------------------
55 Ostreococcus_tauri_virus_OtV5                     16.7%   --------------------------------------------------------------------------------
56 Pacmanvirus_A23                                    9.1%   --------------------------------------------------------------------------------
57 Pandoravirus_dulcis                                9.2%   --------------------------------------------------------------------------------
58 Pandoravirus_inopinatum                            8.8%   --------------------------------------------------------------------------------
59 Pandoravirus_salinus                              10.5%   KKMFRVLTHTGCVDVTEDHSLLDPNAEKVKPTEVSVGSALLHADLPTHECATASLKSRVPMSTQDITDDDGDDDSAKVSA
60 Paramecium_bursaria_Chlorella_virus_CVK2          15.4%   --------------------------------------------------------------------------------
61 Paramecium_bursaria_chlorella_virus_MT325         15.1%   --------------------------------------------------------------------------------
62 Paramecium_bursaria_Chlorella_virus_NYs1          14.2%   --------------------------------------------------------------------------------
63 Phaeocystis_globosa_virus                         16.9%   --------------------------------------------------------------------------------
64 Phaeocystis_globosa_virus_14T                     16.8%   --------------------------------------------------------------------------------
65 Pithovirus_massiliensis                           11.6%   --------------------------------------------------------------------------------
66 Pithovirus_sibericum                              11.8%   --------------------------------------------------------------------------------
67 Port-miou_virus                                   12.4%   --------------------------------------------------------------------------------
68 Powai_lake_megavirus                              60.0%   SHEVNSNRKYKQQSEFNGEVWTSNGWAKIKRVIRHKTVKKLYRVLTNTGCIDVTEDHSLLDTNKNIIKPIDCQIGTELLH
69 Scale_drop_disease_virus                          12.8%   --------------------------------------------------------------------------------
70 Short-finned_eel_ranavirus                        13.1%   --------------------------------------------------------------------------------
71 Singapore_grouper_iridovirus                      13.3%   --------------------------------------------------------------------------------
72 Skunkpox_virus                                    11.0%   --------------------------------------------------------------------------------
73 Spodoptera_frugiperda_ascovirus_1a                10.9%   --------------------------------------------------------------------------------
74 Testudo_hermanni_ranavirus                        13.2%   --------------------------------------------------------------------------------
75 Tiger_frog_virus                                  13.1%   --------------------------------------------------------------------------------
76 Tokyovirus_A1                                     12.9%   --------------------------------------------------------------------------------
77 Trichoplusia_ni_ascovirus_2c                      10.4%   --------------------------------------------------------------------------------
78 Tunisvirus_fontaine2                              12.2%   --------------------------------------------------------------------------------
79 Vaccinia_virus                                    10.6%   --------------------------------------------------------------------------------
80 Variola_virus                                     10.3%   --------------------------------------------------------------------------------
81 Volepox_virus                                     10.8%   --------------------------------------------------------------------------------
82 Wiseana_iridescent_virus                          14.4%   --------------------------------------------------------------------------------
83 Yaba_monkey_tumor_virus                           10.8%   --------------------------------------------------------------------------------
84 Yellowstone_lake_phycodnavirus_1                  17.0%   --------------------------------------------------------------------------------
   consensus/100%                                            ................................................................................
   consensus/90%                                             ................................................................................
   consensus/80%
   consensus/70%

                                                     2561           .         .         .         6         .         .         .         . 26
 1 Acanthamoeba_castellanii_mamavirus               100.0%   GFPESNNVYDNISEQEAYVWGFFMGDGSCGSYQTKNGIKYSWALNNQDLDVLNKCKKYLEETENIQFKILDTMKSSSVYK
 2 Acanthamoeba_polyphaga_mimivirus                  98.6%   GFPESNNVYDNISEQEAYVWGFFMGDGSCGSYQTKNGIKYSWALNNQDLDVLNKCKKYLEETENIQFKILDTMKSSSVYK
 3 Acanthamoeba_polyphaga_moumouvirus                56.0%   GFMETNNIYQNITPHQAYLLGLNFGKVDVYWDIINAQSIWDVINIITNATTKIKQEFIKG--------------------
 4 Amsacta_moorei_entomopoxvirus                     10.1%   --------------------------------------------------------------------------------
 5 Anomala_cuprea_entomopoxvirus                      9.7%   --------------------------------------------------------------------------------
 6 ASFV_BAV71                                         9.7%   --------------------------------------------------------------------------------
 7 ASFV_E75                                           9.7%   --------------------------------------------------------------------------------
 8 Brazilian_marseillevirus                          12.6%   --------------------------------------------------------------------------------
 9 Cafeteria_roenbergensis_virus_BV-PW1              20.9%   SLKKMCENIFDEFKWKISSSSHLSDNIYKLVPFQNEIKLIDFIKYFTNKMYNNGEKKVPQCILNSSKEYIKIFLIGLYPE
10 Cannes_8_virus                                    13.3%   --------------------------------------------------------------------------------
11 Catovirus_CTV1                                    24.3%   IIEDIENDQKDRYSKEQSTTNYLAYTDKGWAKINKIIRHKTTKKIYRILTTNSIVEVTCDHSLIDENGNYIMPKDCVIGK
12 Cedratvirus_A11                                   12.1%   --------------------------------------------------------------------------------
13 Choristoneura_biennis_entomopoxvirus              10.5%   --------------------------------------------------------------------------------
14 Common_midwife_toad_ranavirus                     13.2%   --------------------------------------------------------------------------------
15 Cowpox_virus                                      10.5%   --------------------------------------------------------------------------------
16 Diadromus_pulchellus_ascovirus_4a                 12.7%   --------------------------------------------------------------------------------
17 Ectromelia_virus                                  10.5%   --------------------------------------------------------------------------------
18 Emiliania_huxleyi_virus_145                       14.6%   --------------------------------------------------------------------------------
19 Emiliania_huxleyi_virus_86                        14.6%   --------------------------------------------------------------------------------
20 European_catfish_virus                            13.1%   --------------------------------------------------------------------------------
21 Faustovirus_D3                                     8.8%   --------------------------------------------------------------------------------
22 Faustovirus_E12                                    8.9%   --------------------------------------------------------------------------------
23 Faustovirus_E24                                    8.9%   --------------------------------------------------------------------------------
24 Faustovirus_E9                                     9.0%   --------------------------------------------------------------------------------
25 Faustovirus_Liban                                  9.0%   --------------------------------------------------------------------------------
26 Feldmannia_species_virus                          13.4%   --------------------------------------------------------------------------------
```

```
27 Frog_virus_3                                  13.2%  --------------------------------------------------------------------------------
28 Heliothis_virescens_ascovirus_3e              11.3%  --------------------------------------------------------------------------------
29 Hokovirus_HKV1                                20.3%  --------------------------------------------------------------------------------
30 Infectious_spleen_and_kidney_necrosis_virus   12.7%  --------------------------------------------------------------------------------
31 Insectomime_virus                             12.3%  --------------------------------------------------------------------------------
32 Invertebrate_iridescent_virus_22              13.4%  --------------------------------------------------------------------------------
33 Invertebrate_iridescent_virus_3               14.2%  --------------------------------------------------------------------------------
34 Kaumoebavirus                                 10.2%  --------------------------------------------------------------------------------
35 Klosneuvirus_KNV1                             15.8%  --------------------------------------------------------------------------------
36 Kurlavirus_BKC-1                              12.6%  --------------------------------------------------------------------------------
37 Lausannevirus                                 12.4%  --------------------------------------------------------------------------------
38 Lumpy_skin_disease_virus_NI-2490              10.5%  --------------------------------------------------------------------------------
39 Lymphocystis_disease_virus                    13.5%  --------------------------------------------------------------------------------
40 Marseillevirus_marseillevirus_T19             13.3%  --------------------------------------------------------------------------------
41 Megavirus_chiliensis                          60.1%  GFPEINNNHNKLSLEIYKELDITSELFDCMIESNKKWNNEKMKAYFIGSEYRKQNKNISNEILNCSKKIKKYFLLG----
42 Megavirus_courdo7                             60.4%  GFPEINNNHNKLSLEIYKELDITSELFDCMIESNKKWNNEKMKACFIGSEYRKQNKNISNEILNCSKKIKKYFLLG----
43 Megavirus_ursino                              60.1%  GFPEINNNHNKLSLEIYKEFDITSELFGCMIESDKKWSNEKMKACFIGSEYRKQNKNISNEILNCSKKIKKYFLLG----
44 Melbournevirus                                13.3%  --------------------------------------------------------------------------------
45 Micromonas_pusilla_virus_SP1                  16.3%  --------------------------------------------------------------------------------
46 Mollivirus_sibericum                           9.9%  --------------------------------------------------------------------------------
47 Molluscum_contagiosum_virus_subtype_1          9.1%  --------------------------------------------------------------------------------
48 Monkeypox_virus                               10.5%  --------------------------------------------------------------------------------
49 Myxoma_virus                                  10.7%  --------------------------------------------------------------------------------
50 Noumeavirus                                   12.5%  --------------------------------------------------------------------------------
51 Orf_virus                                     10.2%  --------------------------------------------------------------------------------
52 Organic_Lake_phycodnavirus_1                  18.2%  --------------------------------------------------------------------------------
53 Orpheovirus_LCC2                              12.8%  --------------------------------------------------------------------------------
54 Ostreococcus_lucimarinus_virus_1              16.7%  --------------------------------------------------------------------------------
55 Ostreococcus_tauri_virus_OtV5                 16.7%  --------------------------------------------------------------------------------
56 Pacmanvirus_A23                                9.1%  --------------------------------------------------------------------------------
57 Pandoravirus_dulcis                            9.2%  --------------------------------------------------------------------------------
58 Pandoravirus_inopinatum                        8.8%  --------------------------------------------------------------------------------
59 Pandoravirus_salinus                          10.5%  ASSTSADGSVLVSAADKAHAWAMGMFFAEGSCNEHPRDNCTQYCWRIANKDMALVRMAFDGLEGRYPGVTFSISGPCTDG
60 Paramecium_bursaria_Chlorella_virus_CVK2      15.4%  --------------------------------------------------------------------------------
61 Paramecium_bursaria_chlorella_virus_MT325     15.1%  --------------------------------------------------------------------------------
62 Paramecium_bursaria_Chlorella_virus_NYs1      14.2%  --------------------------------------------------------------------------------
63 Phaeocystis_globosa_virus                     16.9%  --------------------------------------------------------------------------------
64 Phaeocystis_globosa_virus_14T                 16.8%  --------------------------------------------------------------------------------
65 Pithovirus_massiliensis                       11.6%  --------------------------------------------------------------------------------
66 Pithovirus_sibericum                          11.8%  --------------------------------------------------------------------------------
67 Port-miou_virus                               12.4%  --------------------------------------------------------------------------------
68 Powai_lake_megavirus                          60.0%  GFPEINNNNKLPLEIYKELNVTPELFDCMIESDKKRNNEKMKACFIGSEYRKQNINISSKILNCSKKIKKYFLLG-----
69 Scale_drop_disease_virus                      12.8%  --------------------------------------------------------------------------------
70 Short-finned_eel_ranavirus                    13.1%  --------------------------------------------------------------------------------
71 Singapore_grouper_iridovirus                  13.3%  --------------------------------------------------------------------------------
72 Skunkpox_virus                                11.0%  --------------------------------------------------------------------------------
73 Spodoptera_frugiperda_ascovirus_1a            10.9%  --------------------------------------------------------------------------------
74 Testudo_hermanni_ranavirus                    13.2%  --------------------------------------------------------------------------------
75 Tiger_frog_virus                              13.1%  --------------------------------------------------------------------------------
76 Tokyovirus_A1                                 12.9%  --------------------------------------------------------------------------------
77 Trichoplusia_ni_ascovirus_2c                  10.4%  --------------------------------------------------------------------------------
78 Tunisvirus_fontaine2                          12.2%  --------------------------------------------------------------------------------
79 Vaccinia_virus                                10.6%  --------------------------------------------------------------------------------
80 Variola_virus                                 10.3%  --------------------------------------------------------------------------------
81 Volepox_virus                                 10.8%  --------------------------------------------------------------------------------
82 Wiseana_iridescent_virus                      14.4%  --------------------------------------------------------------------------------
83 Yaba_monkey_tumor_virus                       10.8%  --------------------------------------------------------------------------------
84 Yellowstone_lake_phycodnavirus_1              17.0%  --------------------------------------------------------------------------------
   consensus/100%                                       ................................................................................
   consensus/90%                                        ...........................................................................
   consensus/80%
   consensus/70%

                                                 2641            :         .         .         .         .         7         .         . 27
 1 Acanthamoeba_castellanii_mamavirus           100.0%  LVPIRKIKYMVNKYRK------------IFYDNKKYKLVPKEILNSTKDIKNSFLEGYYAADGSRKETENMGCRRCDIKG
 2 Acanthamoeba_polyphaga_mimivirus              98.6%  LVPIRKIKYMVNKYRK------------IFYDNKKYKLVPKEILNSTKDIKNSFLEGYYAADGSRKETENMGCRRCDIKG
 3 Acanthamoeba_polyphaga_moumouvirus            56.0%  ---------------------------------------------------------------------------WKKQG
 4 Amsacta_moorei_entomopoxvirus                 10.1%  --------------------------------------------------------------------------------
 5 Anomala_cuprea_entomopoxvirus                  9.7%  --------------------------------------------------------------------------------
 6 ASFV_BAV71                                     9.7%  --------------------------------------------------------------------------------
 7 ASFV_E75                                       9.7%  --------------------------------------------------------------------------------
 8 Brazilian_marseillevirus                      12.6%  --------------------------------------------------------------------------------
 9 Cafeteria_roenbergensis_virus_BV-PW1          20.9%  YKLENNQ--------------------------------------------------------------------QFIYT
10 Cannes_8_virus                                13.3%  --------------------------------------------------------------------------------
11 Catovirus_CTV1                                24.3%  GLMQSYPHTNKLKYN------------------------------------------------------------DKYDK
12 Cedratvirus_A11                               12.1%  --------------------------------------------------------------------------------
13 Choristoneura_biennis_entomopoxvirus          10.5%  --------------------------------------------------------------------------------
14 Common_midwife_toad_ranavirus                 13.2%  --------------------------------------------------------------------------------
15 Cowpox_virus                                  10.5%  --------------------------------------------------------------------------------
16 Diadromus_pulchellus_ascovirus_4a             12.7%  --------------------------------------------------------------------------------
17 Ectromelia_virus                              10.5%  --------------------------------------------------------------------------------
18 Emiliania_huxleyi_virus_145                   14.6%  --------------------------------------------------------------------------------
19 Emiliania_huxleyi_virus_86                    14.6%  --------------------------------------------------------------------------------
20 European_catfish_virus                        13.1%  --------------------------------------------------------------------------------
21 Faustovirus_D3                                 8.8%  --------------------------------------------------------------------------------
22 Faustovirus_E12                                8.9%  --------------------------------------------------------------------------------
23 Faustovirus_E24                                8.9%  --------------------------------------------------------------------------------
24 Faustovirus_E9                                 9.0%  --------------------------------------------------------------------------------
25 Faustovirus_Liban                              9.0%  --------------------------------------------------------------------------------
26 Feldmannia_species_virus                      13.4%  --------------------------------------------------------------------------------
27 Frog_virus_3                                  13.2%  --------------------------------------------------------------------------------
28 Heliothis_virescens_ascovirus_3e              11.3%  --------------------------------------------------------------------------------
29 Hokovirus_HKV1                                20.3%  --------------------------------------------------------------------------------
30 Infectious_spleen_and_kidney_necrosis_virus   12.7%  --------------------------------------------------------------------------------
31 Insectomime_virus                             12.3%  --------------------------------------------------------------------------------
32 Invertebrate_iridescent_virus_22              13.4%  --------------------------------------------------------------------------------
33 Invertebrate_iridescent_virus_3               14.2%  --------------------------------------------------------------------------------
34 Kaumoebavirus                                 10.2%  --------------------------------------------------------------------------------
35 Klosneuvirus_KNV1                             15.8%  --------------------------------------------------------------------------------
36 Kurlavirus_BKC-1                              12.6%  --------------------------------------------------------------------------------
37 Lausannevirus                                 12.4%  --------------------------------------------------------------------------------
38 Lumpy_skin_disease_virus_NI-2490              10.5%  --------------------------------------------------------------------------------
39 Lymphocystis_disease_virus                    13.5%  --------------------------------------------------------------------------------
40 Marseillevirus_marseillevirus_T19             13.3%  --------------------------------------------------------------------------------
```

```
41 Megavirus_chiliensis                         60.1%    ---------------------------------------------------------------------------YLGND
42 Megavirus_courdo7                            60.4%    ---------------------------------------------------------------------------YLGND
43 Megavirus_ursino                             60.1%    ---------------------------------------------------------------------------YLGND
44 Melbournevirus                               13.3%    --------------------------------------------------------------------------------
45 Micromonas_pusilla_virus_SP1                 16.3%    --------------------------------------------------------------------------------
46 Mollivirus_sibericum                          9.9%    --------------------------------------------------------------------------------
47 Molluscum_contagiosum_virus_subtype_1         9.1%    --------------------------------------------------------------------------------
48 Monkeypox_virus                              10.5%    --------------------------------------------------------------------------------
49 Myxoma_virus                                 10.7%    --------------------------------------------------------------------------------
50 Noumeavirus                                  12.5%    --------------------------------------------------------------------------------
51 Orf_virus                                    10.2%    --------------------------------------------------------------------------------
52 Organic_Lake_phycodnavirus_1                 18.2%    --------------------------------------------------------------------------------
53 Orpheovirus_LCC2                             12.8%    --------------------------------------------------------------------------------
54 Ostreococcus_lucimarinus_virus_1             16.7%    --------------------------------------------------------------------------------
55 Ostreococcus_tauri_virus_OtV5                16.7%    --------------------------------------------------------------------------------
56 Pacmanvirus_A23                               9.1%    --------------------------------------------------------------------------------
57 Pandoravirus_dulcis                           9.2%    --------------------------------------------------------------------------------
58 Pandoravirus_inopinatum                       8.8%    --------------------------------------------------------------------------------
59 Pandoravirus_salinus                         10.5%    MAYVVANGPGKMGLVANYRTAFYDPVYALKRVPTEILNAHVEIKRAFIRGYFAGDGNKKLYGADGTYCGSRCGGKGKIGM
60 Paramecium_bursaria_Chlorella_virus_CVK2     15.4%    --------------------------------------------------------------------------------
61 Paramecium_bursaria_chlorella_virus_MT325    15.1%    --------------------------------------------------------------------------------
62 Paramecium_bursaria_Chlorella_virus_NYs1     14.2%    --------------------------------------------------------------------------------
63 Phaeocystis_globosa_virus                    16.9%    --------------------------------------------------------------------------------
64 Phaeocystis_globosa_virus_14T                16.8%    --------------------------------------------------------------------------------
65 Pithovirus_massiliensis                      11.6%    --------------------------------------------------------------------------------
66 Pithovirus_sibericum                         11.8%    --------------------------------------------------------------------------------
67 Port-miou_virus                              12.4%    --------------------------------------------------------------------------------
68 Powai_lake_megavirus                         60.0%    ---------------------------------------------------------------------------YLGND
69 Scale_drop_disease_virus                     12.8%    --------------------------------------------------------------------------------
70 Short-finned_eel_ranavirus                   13.1%    --------------------------------------------------------------------------------
71 Singapore_grouper_iridovirus                 13.3%    --------------------------------------------------------------------------------
72 Skunkpox_virus                               11.0%    --------------------------------------------------------------------------------
73 Spodoptera_frugiperda_ascovirus_1a           10.9%    --------------------------------------------------------------------------------
74 Testudo_hermanni_ranavirus                   13.2%    --------------------------------------------------------------------------------
75 Tiger_frog_virus                             13.1%    --------------------------------------------------------------------------------
76 Tokyovirus_A1                                12.9%    --------------------------------------------------------------------------------
77 Trichoplusia_ni_ascovirus_2c                 10.4%    --------------------------------------------------------------------------------
78 Tunisvirus_fontaine2                         12.2%    --------------------------------------------------------------------------------
79 Vaccinia_virus                               10.6%    --------------------------------------------------------------------------------
80 Variola_virus                                10.3%    --------------------------------------------------------------------------------
81 Volepox_virus                                10.8%    --------------------------------------------------------------------------------
82 Wiseana_iridescent_virus                     14.4%    --------------------------------------------------------------------------------
83 Yaba_monkey_tumor_virus                      10.8%    --------------------------------------------------------------------------------
84 Yellowstone_lake_phycodnavirus_1             17.0%    --------------------------------------------------------------------------------
   consensus/100%                                        ................................................................................
   consensus/90%                                                                                                                    .....
   consensus/80%
   consensus/70%

                                                     2721         .         .         :         .         .         .         .        8 28
 1 Acanthamoeba_castellanii_mamavirus          100.0%    KISAQCLFYLLKSLGYNVSINIRSDKNQIYRLTFSNKKQRKNPIAIKKIQLMNETSNDHDGDYVYDLETESGSFHAGVGE
 2 Acanthamoeba_polyphaga_mimivirus             98.6%    KISAQCLFYLLKSLGYNVSINIRSDKNQIYRLTFSNKKQRKNPIAIKKIQLMNETSNDHDGDYVYDLETESGSFHAGVGE
 3 Acanthamoeba_polyphaga_moumouvirus           56.0%    FYNIENKVEAQFLYYVLKSLGHNVNIHMPITKEDIYRLSYGKNISNINKTTIQYLRETYDGEYVYDLETESGTFHAGIGE
 4 Amsacta_moorei_entomopoxvirus                10.1%    --------------------------------------------------------------------------------
 5 Anomala_cuprea_entomopoxvirus                 9.7%    --------------------------------------------------------------------------------
 6 ASFV_BAV71                                    9.7%    --------------------------------------------------------------------------------
 7 ASFV_E75                                      9.7%    --------------------------------------------------------------------------------
 8 Brazilian_marseillevirus                     12.6%    --------------------------------------------------------------------------------
 9 Cafeteria_roenbergensis_virus_BV-PW1         20.9%    CKNNEFSLGIYYLIKKLGYHVKLNSNDSSDSSDSIYTFEISHKLENNNNVITKITEWEHKETYVYDLTTENHHFHAGVGS
10 Cannes_8_virus                               13.3%    --------------------------------------------------------------------------------
11 Catovirus_CTV1                               24.3%    SIFTNKNYKECMKYYHYNKSHGFNITIDENNGIITLKRTKDNINNINKIVKIFDLGDISTDRYVYDLETEHGRFHAGVGE
12 Cedratvirus_A11                              12.1%    --------------------------------------------------------------------------------
13 Choristoneura_biennis_entomopoxvirus         10.5%    --------------------------------------------------------------------------------
14 Common_midwife_toad_ranavirus                13.2%    --------------------------------------------------------------------------------
15 Cowpox_virus                                 10.5%    --------------------------------------------------------------------------------
16 Diadromus_pulchellus_ascovirus_4a            12.7%    --------------------------------------------------------------------------------
17 Ectromelia_virus                             10.5%    --------------------------------------------------------------------------------
18 Emiliania_huxleyi_virus_145                  14.6%    --------------------------------------------------------------------------------
19 Emiliania_huxleyi_virus_86                   14.6%    --------------------------------------------------------------------------------
20 European_catfish_virus                       13.1%    --------------------------------------------------------------------------------
21 Faustovirus_D3                                8.8%    --------------------------------------------------------------------------------
22 Faustovirus_E12                               8.9%    --------------------------------------------------------------------------------
23 Faustovirus_E24                               8.9%    --------------------------------------------------------------------------------
24 Faustovirus_E9                                9.0%    --------------------------------------------------------------------------------
25 Faustovirus_Liban                             9.0%    --------------------------------------------------------------------------------
26 Feldmannia_species_virus                     13.4%    --------------------------------------------------------------------------------
27 Frog_virus_3                                 13.2%    --------------------------------------------------------------------------------
28 Heliothis_virescens_ascovirus_3e             11.3%    --------------------------------------------------------------------------------
29 Hokovirus_HKV1                               20.3%    --------------------------------------------------------------------------------
30 Infectious_spleen_and_kidney_necrosis_virus  12.7%    --------------------------------------------------------------------------------
31 Insectomime_virus                            12.3%    --------------------------------------------------------------------------------
32 Invertebrate_iridescent_virus_22             13.4%    --------------------------------------------------------------------------------
33 Invertebrate_iridescent_virus_3              14.2%    --------------------------------------------------------------------------------
34 Kaumoebavirus                                10.2%    --------------------------------------------------------------------------------
35 Klosneuvirus_KNV1                            15.8%    --------------------------------------------------------------------------------
36 Kurlavirus_BKC-1                             12.6%    --------------------------------------------------------------------------------
37 Lausannevirus                                12.4%    --------------------------------------------------------------------------------
38 Lumpy_skin_disease_virus_NI-2490             10.5%    --------------------------------------------------------------------------------
39 Lymphocystis_disease_virus                   13.5%    --------------------------------------------------------------------------------
40 Marseillevirus_marseillevirus_T19            13.3%    --------------------------------------------------------------------------------
41 Megavirus_chiliensis                         60.1%    KEYITNNKINAQIIYCLMKCLEYNIVIDLIESSYKLCIIDNINEPYCINKIIQLQDTSINGEYVYDLETESGTFHAGIGE
42 Megavirus_courdo7                            60.4%    KEYITNNKINAQMVYYLMKCLGYNIVIDLIESSYKLCIVDNINKPYCINKIIQLQDTSINGEYVYDLETESGTFHAGIGE
43 Megavirus_ursino                             60.1%    KEYITNNKINAQMIYCLMKCLGYNIVIDLIESSYKLCIVDNISKPYCINKIIQLQDTSINGEYVYDLETESGTFHAGIGE
44 Melbournevirus                               13.3%    --------------------------------------------------------------------------------
45 Micromonas_pusilla_virus_SP1                 16.3%    --------------------------------------------------------------------------------
46 Mollivirus_sibericum                          9.9%    --------------------------------------------------------------------------------
47 Molluscum_contagiosum_virus_subtype_1         9.1%    --------------------------------------------------------------------------------
48 Monkeypox_virus                              10.5%    --------------------------------------------------------------------------------
49 Myxoma_virus                                 10.7%    --------------------------------------------------------------------------------
50 Noumeavirus                                  12.5%    --------------------------------------------------------------------------------
51 Orf_virus                                    10.2%    --------------------------------------------------------------------------------
52 Organic_Lake_phycodnavirus_1                 18.2%    --------------------------------------------------------------------------------
53 Orpheovirus_LCC2                             12.8%    --------------------------------------------------------------------------------
54 Ostreococcus_lucimarinus_virus_1             16.7%    --------------------------------------------------------------------------------
```

```
55 Ostreococcus_tauri_virus_OtV5                 16.7%   --------------------------------------------------------------------------------
56 Pacmanvirus_A23                                9.1%   --------------------------------------------------------------------------------
57 Pandoravirus_dulcis                            9.2%   --------------------------------------------------------------------------------
58 Pandoravirus_inopinatum                        8.8%   --------------------------------------------------------------------------------
59 Pandoravirus_salinus                          10.5%   AGIYYLLSACGYLASVDTCGGPERDTYRINFCDAATARRTQRKPADTVKKIIPLDSAAFDGAYVYDLETANHHFAAGIGR
60 Paramecium_bursaria_Chlorella_virus_CVK2      15.4%   --------------------------------------------------------------------------------
61 Paramecium_bursaria_chlorella_virus_MT325     15.1%   --------------------------------------------------------------------------------
62 Paramecium_bursaria_Chlorella_virus_NYs1      14.2%   --------------------------------------------------------------------------------
63 Phaeocystis_globosa_virus                     16.9%   --------------------------------------------------------------------------------
64 Phaeocystis_globosa_virus_14T                 16.8%   --------------------------------------------------------------------------------
65 Pithovirus_massiliensis                       11.6%   --------------------------------------------------------------------------------
66 Pithovirus_sibericum                          11.8%   --------------------------------------------------------------------------------
67 Port-miou_virus                               12.4%   --------------------------------------------------------------------------------
68 Powai_lake_megavirus                          60.0%   KEYITNDKINAQIIYCLMKCLGYNIVIDLIESSYKLCIIDNISKPYCINKIIQLQYTSINGEYVYDLETESGTFHAGIGE
69 Scale_drop_disease_virus                      12.8%   --------------------------------------------------------------------------------
70 Short-finned_eel_ranavirus                    13.1%   --------------------------------------------------------------------------------
71 Singapore_grouper_iridovirus                  13.3%   --------------------------------------------------------------------------------
72 Skunkpox_virus                                11.0%   --------------------------------------------------------------------------------
73 Spodoptera_frugiperda_ascovirus_1a            10.9%   --------------------------------------------------------------------------------
74 Testudo_hermanni_ranavirus                    13.2%   --------------------------------------------------------------------------------
75 Tiger_frog_virus                              13.1%   --------------------------------------------------------------------------------
76 Tokyovirus_A1                                 12.9%   --------------------------------------------------------------------------------
77 Trichoplusia_ni_ascovirus_2c                  10.4%   --------------------------------------------------------------------------------
78 Tunisvirus_fontaine2                          12.2%   --------------------------------------------------------------------------------
79 Vaccinia_virus                                10.6%   --------------------------------------------------------------------------------
80 Variola_virus                                 10.3%   --------------------------------------------------------------------------------
81 Volepox_virus                                 10.8%   --------------------------------------------------------------------------------
82 Wiseana_iridescent_virus                      14.4%   --------------------------------------------------------------------------------
83 Yaba_monkey_tumor_virus                       10.8%   --------------------------------------------------------------------------------
84 Yellowstone_lake_phycodnavirus_1              17.0%   --------------------------------------------------------------------------------
   consensus/100%                                        ................................................................................
   consensus/90%                                         ................................................................................
   consensus/80%
   consensus/70%

                                                    2801          .         .         .         :         .         .         . 28
 1 Acanthamoeba_castellanii_mamavirus           100.0%   MIVKNTDSIFINFHIKDENGE-----------------------------------------------------------
 2 Acanthamoeba_polyphaga_mimivirus              98.6%   MIVKNTDSIFINF--HIKDEN-----------------------------------------------------------
 3 Acanthamoeba_polyphaga_moumouvirus            56.0%   MIVKNTDSIFINF--HIKDEN-----------------------------------------------------------
 4 Amsacta_moorei_entomopoxvirus                 10.1%   ----DTDSLFITVNFESKFNS-----------------------------------------------------------
 5 Anomala_cuprea_entomopoxvirus                  9.7%   ----DTDSLFMSI--IFNKEM-----------------------------------------------------------
 6 ASFV_BAV71                                     9.7%   ----DTDSLYITC--PDSLYT-----------------------------------------------------------
 7 ASFV_E75                                       9.7%   ----DTDSLYITC--PDSLYT-----------------------------------------------------------
 8 Brazilian_marseillevirus                      12.6%   ----DTDSCMIHF--PG---------------------------------------------------------------
 9 Cafeteria_roenbergensis_virus_BV-PW1          20.9%   IIVHNTDSIFSCYQYRENTSKISTENKLTLFQEIIKFGKELILPFIPREYQEEFDKLYTKYYGKIRNLKLNSNLKVKPEP
10 Cannes_8_virus                                13.3%   ----DTDSCMIHY--P----------------------------------------------------------------
11 Catovirus_CTV1                                24.3%   LIVTNTDSTFFKP--QITNNK-----------------------------------------------------------
12 Cedratvirus_A11                               12.1%   ----DTDSVFFVV--K----------------------------------------------------------------
13 Choristoneura_biennis_entomopoxvirus          10.5%   ----DTDSLFINIKFDNKFDN-----------------------------------------------------------
14 Common_midwife_toad_ranavirus                 13.2%   ----DTDSNYVTF--AD---------------------------------------------------------------
15 Cowpox_virus                                  10.5%   ----DTDSVFTEI--D----------------------------------------------------------------
16 Diadromus_pulchellus_ascovirus_4a             12.7%   ----DTDSNYITF--DDISGDH----------------------------------------------------------
17 Ectromelia_virus                              10.5%   ----DTDSVFTEI--D----------------------------------------------------------------
18 Emiliania_huxleyi_virus_145                   14.6%   ----DTDSVFVRV--KDKNGK-----------------------------------------------------------
19 Emiliania_huxleyi_virus_86                    14.6%   ----DTDSVFVRV--KDKNGK-----------------------------------------------------------
20 European_catfish_virus                        13.1%   ----DTDSNYVTF--AD---------------------------------------------------------------
21 Faustovirus_D3                                 8.8%   ----DTDSMYISA--PEVIFE-----------------------------------------------------------
22 Faustovirus_E12                                8.9%   ----DTDSMYISA--PEKIFE-----------------------------------------------------------
23 Faustovirus_E24                                8.9%   ----DTDSMYISA--PEKIFE-----------------------------------------------------------
24 Faustovirus_E9                                 9.0%   ----DTDSMYISA--PEAIFE-----------------------------------------------------------
25 Faustovirus_Liban                              9.0%   ----DTDSMYISA--PEKIFE-----------------------------------------------------------
26 Feldmannia_species_virus                      13.4%   ----DTDSVMCTF--PAPQTV-----------------------------------------------------------
27 Frog_virus_3                                  13.2%   ----DTDSNYVTF--AD---------------------------------------------------------------
28 Heliothis_virescens_ascovirus_3e              11.3%   ----DTDSMYVQL--G----------------------------------------------------------------
29 Hokovirus_HKV1                                20.3%   ----DTDSVFIKY--DIKNSI-----------------------------------------------------------
30 Infectious_spleen_and_kidney_necrosis_virus   12.7%   ----DTDSCYIQLGHD----------------------------------------------------------------
31 Insectomime_virus                             12.3%   ----DTDSCMIHF--PG---------------------------------------------------------------
32 Invertebrate_iridescent_virus_22              13.4%   ----DTDSNYVSFQHK----------------------------------------------------------------
33 Invertebrate_iridescent_virus_3               14.2%   ----DTDSNYVSF--NHK--------------------------------------------------------------
34 Kaumoebavirus                                 10.2%   ----DTDSLYVVC--REEIFR-----------------------------------------------------------
35 Klosneuvirus_KNV1                             15.8%   --------------------------------------------------------------------------------
36 Kurlavirus_BKC-1                              12.6%   ----DTDSCMIHF--PG---------------------------------------------------------------
37 Lausannevirus                                 12.4%   ----DTDSCMIHF--PG---------------------------------------------------------------
38 Lumpy_skin_disease_virus_NI-2490              10.5%   ----DTDSIFLEM--D----------------------------------------------------------------
39 Lymphocystis_disease_virus                    13.5%   ----DTDSNYVQF--KD---------------------------------------------------------------
40 Marseillevirus_marseillevirus_T19             13.3%   ----DTDSCMIHY--P----------------------------------------------------------------
41 Megavirus_chiliensis                          60.1%   LIVKNTDSIFINF--HIKDEN-----------------------------------------------------------
42 Megavirus_courdo7                             60.4%   LIVKNTDSIFINF--HIKDEN-----------------------------------------------------------
43 Megavirus_ursino                              60.1%   LIVKNTDSIFINF--HIKDEN-----------------------------------------------------------
44 Melbournevirus                                13.3%   ----DTDSCMIHY--P----------------------------------------------------------------
45 Micromonas_pusilla_virus_SP1                  16.3%   ----DTDSVMVEF--DVGDRK-----------------------------------------------------------
46 Mollivirus_sibericum                           9.9%   ----DTDSVMIQFLGPDGVR------------------------------------------------------------
47 Molluscum_contagiosum_virus_subtype_1          9.1%   ----DTDSIFLEL--N----------------------------------------------------------------
48 Monkeypox_virus                               10.5%   ----DTDSVFTEI--D----------------------------------------------------------------
49 Myxoma_virus                                  10.7%   ----DTDSVFLEI--N----------------------------------------------------------------
50 Noumeavirus                                   12.5%   ----DTDSCMIHF--PG---------------------------------------------------------------
51 Orf_virus                                     10.2%   ----DTDSVFLEV--G----------------------------------------------------------------
52 Organic_Lake_phycodnavirus_1                  18.2%   ----DTDSVFFTF--NFKDLK-----------------------------------------------------------
53 Orpheovirus_LCC2                              12.8%   ----DTDSCMIDV--G----------------------------------------------------------------
54 Ostreococcus_lucimarinus_virus_1              16.7%   ----DTDSVMVEFDVGDRKG------------------------------------------------------------
55 Ostreococcus_tauri_virus_OtV5                 16.7%   ----DTDSVMVEFDVGDRKG------------------------------------------------------------
56 Pacmanvirus_A23                                9.1%   ----DTDSLYLSC--PDSVYT-----------------------------------------------------------
57 Pandoravirus_dulcis                            9.2%   ----DTDSVMIHF--HGVPKT-----------------------------------------------------------
58 Pandoravirus_inopinatum                        8.8%   ----TGDHRLVQF--EAGSST-----------------------------------------------------------
59 Pandoravirus_salinus                          10.5%   LVVHNTDSVMIKF--HGVPAT-----------------------------------------------------------
60 Paramecium_bursaria_Chlorella_virus_CVK2      15.4%   ----DTDSVMVKMKLPDDK-------------------------------------------------------------
61 Paramecium_bursaria_chlorella_virus_MT325     15.1%   ----DTDSIMVKMKLPEGKN------------------------------------------------------------
62 Paramecium_bursaria_Chlorella_virus_NYs1      14.2%   ----DTDSVMVKMKLPDDK-------------------------------------------------------------
63 Phaeocystis_globosa_virus                     16.9%   ----DTDSVFFKF--NLKTPE-----------------------------------------------------------
64 Phaeocystis_globosa_virus_14T                 16.8%   ----DTDSVFFKF--NLKTPE-----------------------------------------------------------
65 Pithovirus_massiliensis                       11.6%   ----DTDSIFFPT--Q----------------------------------------------------------------
66 Pithovirus_sibericum                          11.8%   ----DTDSIFFPT--Q----------------------------------------------------------------
67 Port-miou_virus                               12.4%   ----DTDSCMIHF--PG---------------------------------------------------------------
68 Powai_lake_megavirus                          60.0%   LIVKNTDSIFINF--HIKDEN-----------------------------------------------------------
```

```
69 Scale_drop_disease_virus                          12.8%   ----DTDSCYVNLGHP----------------------------------------------------------------
70 Short-finned_eel_ranavirus                        13.1%   ----DTDSNYVTF--AE---------------------------------------------------------------
71 Singapore_grouper_iridovirus                      13.3%   ----DTDSNYVTF--DG---------------------------------------------------------------
72 Skunkpox_virus                                    11.0%   ----DTDSVFTEI--D----------------------------------------------------------------
73 Spodoptera_frugiperda_ascovirus_1a                10.9%   ----DTDSMYVQL--QDD--------------------------------------------------------------
74 Testudo_hermanni_ranavirus                        13.2%   ----DTDSNYVTF--AD---------------------------------------------------------------
75 Tiger_frog_virus                                  13.1%   ----DTDSNYVTF--AD---------------------------------------------------------------
76 Tokyovirus_A1                                     12.9%   ----DTDSCMIHY--P----------------------------------------------------------------
77 Trichoplusia_ni_ascovirus_2c                      10.4%   ----DTDSMYVQL-------------------------------------------------------------------
78 Tunisvirus_fontaine2                              12.2%   ----DTDSCMIHF--PG---------------------------------------------------------------
79 Vaccinia_virus                                    10.6%   ----DTDSVFTEI--D----------------------------------------------------------------
80 Variola_virus                                     10.3%   ----DTDSVFTEI--D----------------------------------------------------------------
81 Volepox_virus                                     10.8%   ----DTDSVFTEI--D----------------------------------------------------------------
82 Wiseana_iridescent_virus                          14.4%   ----DTDSTYFSF--KD---------------------------------------------------------------
83 Yaba_monkey_tumor_virus                           10.8%   ----DTDSVFLEM--N----------------------------------------------------------------
84 Yellowstone_lake_phycodnavirus_1                  17.0%   ----DTDSVMVEFDVQGRKG------------------------------------------------------------
   consensus/100%                                            ................................................................................
   consensus/90%                                             ....sTDS.hhph........
   consensus/80%                                                 DTDShhhph  ......
   consensus/70%                                                 DTDShalph  ......

                                                      2881            .         9         .         .         .         .         :         . 29
 1 Acanthamoeba_castellanii_mamavirus               100.0%   --------------------------------------------------------------------------------
 2 Acanthamoeba_polyphaga_mimivirus                  98.6%   --------------------------------------------------------------------------G-----
 3 Acanthamoeba_polyphaga_moumouvirus                56.0%   --------------------------------------------------------------------------G-----
 4 Amsacta_moorei_entomopoxvirus                     10.1%   --------------------------------------------------------------------------------
 5 Anomala_cuprea_entomopoxvirus                      9.7%   --------------------------------------------------------------------------E-----
 6 ASFV_BAV71                                         9.7%   --------------------------------------------------------------------------------
 7 ASFV_E75                                           9.7%   --------------------------------------------------------------------------------
 8 Brazilian_marseillevirus                          12.6%   --------------------------------------------------------------------------------
 9 Cafeteria_roenbergensis_virus_BV-PW1              20.9%   THFKIILPIHDRLKQFLKEYLEESYFPWLWTLHDIFTMDLSYLDYKQRNEIMEVKLFNHGFSQIEKMRLLPSIFSSADKY
10 Cannes_8_virus                                    13.3%   --------------------------------------------------------------------------------
11 Catovirus_CTV1                                    24.3%   --------------------------------------------------------------------------T-----
12 Cedratvirus_A11                                   12.1%   --------------------------------------------------------------------------------
13 Choristoneura_biennis_entomopoxvirus              10.5%   --------------------------------------------------------------------------------
14 Common_midwife_toad_ranavirus                     13.2%   --------------------------------------------------------------------------------
15 Cowpox_virus                                      10.5%   --------------------------------------------------------------------------------
16 Diadromus_pulchellus_ascovirus_4a                 12.7%   --------------------------------------------------------------------------------
17 Ectromelia_virus                                  10.5%   --------------------------------------------------------------------------------
18 Emiliania_huxleyi_virus_145                       14.6%   --------------------------------------------------------------------------------
19 Emiliania_huxleyi_virus_86                        14.6%   --------------------------------------------------------------------------------
20 European_catfish_virus                            13.1%   --------------------------------------------------------------------------------
21 Faustovirus_D3                                     8.8%   --------------------------------------------------------------------------------
22 Faustovirus_E12                                    8.9%   --------------------------------------------------------------------------------
23 Faustovirus_E24                                    8.9%   --------------------------------------------------------------------------------
24 Faustovirus_E9                                     9.0%   --------------------------------------------------------------------------------
25 Faustovirus_Liban                                  9.0%   --------------------------------------------------------------------------------
26 Feldmannia_species_virus                          13.4%   --------------------------------------------------------------------------------
27 Frog_virus_3                                      13.2%   --------------------------------------------------------------------------------
28 Heliothis_virescens_ascovirus_3e                  11.3%   --------------------------------------------------------------------------------
29 Hokovirus_HKV1                                    20.3%   --------------------------------------------------------------------------T-----
30 Infectious_spleen_and_kidney_necrosis_virus       12.7%   --------------------------------------------------------------------------------
31 Insectomime_virus                                 12.3%   --------------------------------------------------------------------------------
32 Invertebrate_iridescent_virus_22                  13.4%   --------------------------------------------------------------------------------
33 Invertebrate_iridescent_virus_3                   14.2%   --------------------------------------------------------------------------------
34 Kaumoebavirus                                     10.2%   --------------------------------------------------------------------------------
35 Klosneuvirus_KNV1                                 15.8%   --------------------------------------------------------------------------------
36 Kurlavirus_BKC-1                                  12.6%   --------------------------------------------------------------------------------
37 Lausannevirus                                     12.4%   --------------------------------------------------------------------------------
38 Lumpy_skin_disease_virus_NI-2490                  10.5%   --------------------------------------------------------------------------------
39 Lymphocystis_disease_virus                        13.5%   --------------------------------------------------------------------------------
40 Marseillevirus_marseillevirus_T19                 13.3%   --------------------------------------------------------------------------------
41 Megavirus_chiliensis                              60.1%   --------------------------------------------------------------------------G-----
42 Megavirus_courdo7                                 60.4%   --------------------------------------------------------------------------G-----
43 Megavirus_ursino                                  60.1%   --------------------------------------------------------------------------G-----
44 Melbournevirus                                    13.3%   --------------------------------------------------------------------------------
45 Micromonas_pusilla_virus_SP1                      16.3%   --------------------------------------------------------------------------G-----
46 Mollivirus_sibericum                               9.9%   --------------------------------------------------------------------------------
47 Molluscum_contagiosum_virus_subtype_1              9.1%   --------------------------------------------------------------------------------
48 Monkeypox_virus                                   10.5%   --------------------------------------------------------------------------------
49 Myxoma_virus                                      10.7%   --------------------------------------------------------------------------------
50 Noumeavirus                                       12.5%   --------------------------------------------------------------------------------
51 Orf_virus                                         10.2%   --------------------------------------------------------------------------------
52 Organic_Lake_phycodnavirus_1                      18.2%   --------------------------------------------------------------------------N-----
53 Orpheovirus_LCC2                                  12.8%   --------------------------------------------------------------------------------
54 Ostreococcus_lucimarinus_virus_1                  16.7%   --------------------------------------------------------------------------------
55 Ostreococcus_tauri_virus_OtV5                     16.7%   --------------------------------------------------------------------------------
56 Pacmanvirus_A23                                    9.1%   ---------------------------------------------------------------------------KCDEE
57 Pandoravirus_dulcis                                9.2%   --------------------------------------------------------------------------------
58 Pandoravirus_inopinatum                            8.8%   --------------------------------------------------------------------------G-----
59 Pandoravirus_salinus                              10.5%   --------------------------------------------------------------------------------
60 Paramecium_bursaria_Chlorella_virus_CVK2          15.4%   --------------------------------------------------------------------------------
61 Paramecium_bursaria_chlorella_virus_MT325         15.1%   --------------------------------------------------------------------------------
62 Paramecium_bursaria_Chlorella_virus_NYs1          14.2%   --------------------------------------------------------------------------------
63 Phaeocystis_globosa_virus                         16.9%   --------------------------------------------------------------------------G-----
64 Phaeocystis_globosa_virus_14T                     16.8%   --------------------------------------------------------------------------G-----
65 Pithovirus_massiliensis                           11.6%   --------------------------------------------------------------------------------
66 Pithovirus_sibericum                              11.8%   --------------------------------------------------------------------------------
67 Port-miou_virus                                   12.4%   --------------------------------------------------------------------------------
68 Powai_lake_megavirus                              60.0%   --------------------------------------------------------------------------G-----
69 Scale_drop_disease_virus                          12.8%   --------------------------------------------------------------------------------
70 Short-finned_eel_ranavirus                        13.1%   --------------------------------------------------------------------------------
71 Singapore_grouper_iridovirus                      13.3%   --------------------------------------------------------------------------------
72 Skunkpox_virus                                    11.0%   --------------------------------------------------------------------------------
73 Spodoptera_frugiperda_ascovirus_1a                10.9%   --------------------------------------------------------------------------------
74 Testudo_hermanni_ranavirus                        13.2%   --------------------------------------------------------------------------------
75 Tiger_frog_virus                                  13.1%   --------------------------------------------------------------------------------
76 Tokyovirus_A1                                     12.9%   --------------------------------------------------------------------------------
77 Trichoplusia_ni_ascovirus_2c                      10.4%   --------------------------------------------------------------------------------
78 Tunisvirus_fontaine2                              12.2%   --------------------------------------------------------------------------------
79 Vaccinia_virus                                    10.6%   --------------------------------------------------------------------------------
80 Variola_virus                                     10.3%   --------------------------------------------------------------------------------
81 Volepox_virus                                     10.8%   --------------------------------------------------------------------------------
82 Wiseana_iridescent_virus                          14.4%   --------------------------------------------------------------------------------
```

```
83 Yaba_monkey_tumor_virus                         10.8%  --------------------------------------------------------------------------------
84 Yellowstone_lake_phycodnavirus_1                17.0%  --------------------------------------------------------------------------------
   consensus/100%                                         ................................................................................
   consensus/90%                                                                                                                    .
   consensus/80%
   consensus/70%

                                                   2961        .         .         .         0         .         .         .         .    30
 1 Acanthamoeba_castellanii_mamavirus             100.0%  -------------------------------------------EKTDKEALMKT-----I------AKCQRAAKLIN-QN
 2 Acanthamoeba_polyphaga_mimivirus                98.6%  ------------------------------------------EEKTDKEALMKT-----I------AKCQRAAKLIN-QN
 3 Acanthamoeba_polyphaga_moumouvirus              56.0%  ------------------------------------------NERTDREALIQT-----I------AKCQRAAKLIN-QN
 4 Amsacta_moorei_entomopoxvirus                   10.1%  -----------------------------------------------KEEKVKVGHKCFT------FLGNVINDKKN-KI
 5 Anomala_cuprea_entomopoxvirus                    9.7%  ----------------------------------------------LDYKIRKA-----I------DIGNFLSNIINKND
 6 ASFV_BAV71                                       9.7%  -----------------------EVTDAYLNSQKTIKHYEQLCHEKVLLSMKAM-----S------TLCAEVNEYLR-QD
 7 ASFV_E75                                         9.7%  -----------------------EVTDAYLNSQKTIKHYEQLCHEKVLLSMKAM-----S------TLCAEVNEYLR-QD
 8 Brazilian_marseillevirus                        12.6%  -----------------------------------------------GRTPKDL-----I------EFGVFVGKEIS-KH
 9 Cafeteria_roenbergensis_virus_BV-PW1            20.9%  NLINQIRNFCKNTLKQYYLIPYWKFNSKTNKLEYRMDFRNKGDPIIDKRNLDKS-----I------DMGIISGELIK-KR
10 Cannes_8_virus                                  13.3%  -----------------------------------------------GKTPKEL-----I------EFGEMVGKEIS-KH
11 Catovirus_CTV1                                  24.3%  -----------------------------------------------GEIRKDF-----WSRECCIQLGIWASITIC-IL
12 Cedratvirus_A11                                 12.1%  -----------------------------------------------ADSYEEA-----W------KIAKAMEKDVS-SV
13 Choristoneura_biennis_entomopoxvirus            10.5%  -----------------------------------------------KEDLVNKSHECFQ------FLSNIINDEKN-II
14 Common_midwife_toad_ranavirus                   13.2%  -----------------------------------------------AKTPAEL-----W------DKAVAVAKAVS-SV
15 Cowpox_virus                                    10.5%  -----------------------------------------------SKDVDKS-----I------EIAKELERLIN-SR
16 Diadromus_pulchellus_ascovirus_4a               12.7%  -----------------------------------------------KDKCEKI-----W------TRALETADQIS-CH
17 Ectromelia_virus                                10.5%  -----------------------------------------------SQDVDKS-----I------EIAKELERLIN-SR
18 Emiliania_huxleyi_virus_145                     14.6%  -----------------------------------------------ELTPAEV-----F------KVGEDVAQKIS-DS
19 Emiliania_huxleyi_virus_86                      14.6%  -----------------------------------------------ELTPAEV-----F------KVGEDVAQKIS-DS
20 European_catfish_virus                          13.1%  -----------------------------------------------AKTPAEL-----W------DKAVAVAKAVS-SV
21 Faustovirus_D3                                   8.8%  -------LVDAWFKAKTEEARVVAETDPVAGEALLRKYREEYYTQMVQLTMTRM-----K------TLCEEISDRLA-AR
22 Faustovirus_E12                                  8.9%  -------------MIDAWFAERSAEAKAANDDALLRRYREEYYATMVQLTMDRM-----Q------ILCEEISDRLT-AR
23 Faustovirus_E24                                  8.9%  -------------MIDAWFAERSAEAKAANDDALLRRYREEYYATMVQLTMDRM-----Q------ILCEEISDRLT-AR
24 Faustovirus_E9                                   9.0%  -------LIDAWFKMKTEEARLVSVDDPVAGEALLRKYREEYYTQMVQLTMTRM-----K------TLCEEISDRLA-AR
25 Faustovirus_Liban                                9.0%  -------------MIDAWFAERSAEAKAANDDALLRRYREEYYTVMVQLTMDRM-----K------ILCEEISDRLT-AR
26 Feldmannia_species_virus                        13.4%  -----------------------------------------------RGQGKRALLGHAY------AMGLSAEEKSL-SL
27 Frog_virus_3                                    13.2%  -----------------------------------------------AKTPAEL-----W------DKAVAVAKAVS-SV
28 Heliothis_virescens_ascovirus_3e                11.3%  -----------------------------------------------DEAGDDP-----W------KFVRELAAHIT-SK
29 Hokovirus_HKV1                                  20.3%  -----------------------------------------LENLQNKDALKMS-----I------RLGILTSAIIN-VI
30 Infectious_spleen_and_kidney_necrosis_virus     12.7%  -----------------------------------------------RASLDEL-----W------QMAVNASDTVS-AF
31 Insectomime_virus                               12.3%  -----------------------------------------------GRTPKDL-----I------EFGEFVGAEIS-KH
32 Invertebrate_iridescent_virus_22                13.4%  -----------------------------------------------KFSITEL-----W------DYAIKVADEIS-KL
33 Invertebrate_iridescent_virus_3                 14.2%  -----------------------------------------------NMSMSEL-----W------DYAIRVADEIS-QQ
34 Kaumoebavirus                                   10.2%  --------------------------EIDRKYQSGEIDKMTYWSEMVTLSMREL-----D------RVKKAVKGRLI-EI
35 Klosneuvirus_KNV1                               15.8%  --------------------------------------------------------------------------------
36 Kurlavirus_BKC-1                                12.6%  -----------------------------------------------GRTPKDL-----I------EFGKFVGKEIS-KH
37 Lausannevirus                                   12.4%  -----------------------------------------------GRTPKDL-----I------EFGKFVGKEIS-KH
38 Lumpy_skin_disease_virus_NI-2490                10.5%  -----------------------------------------------TNDISKS-----I------RVAKELEKIIN-ER
39 Lymphocystis_disease_virus                      13.5%  -----------------------------------------------VPDLTAL-----W------NKAEEVADNVS-KE
40 Marseillevirus_marseillevirus_T19               13.3%  -----------------------------------------------GKTPKEL-----I------EFGEMVGKEIS-KH
41 Megavirus_chiliensis                            60.1%  ------------------------------------------QERTDKEALIQT-----I------AKCQRAAKLIN-QN
42 Megavirus_courdo7                               60.4%  ------------------------------------------QERTDKEALIQT-----I------AKCQRAAKLIN-QN
43 Megavirus_ursino                                60.1%  ------------------------------------------QERTDKEALIQT-----I------AKCQRAAKLIN-QN
44 Melbournevirus                                  13.3%  -----------------------------------------------GKTPKEL-----I------EFGEMVGKEIS-KH
45 Micromonas_pusilla_virus_SP1                    16.3%  -----------------------------------------------VDAVKYS-----W------EVGERAAEECS-AL
46 Mollivirus_sibericum                             9.9%  -----------------------------------------------GASLDYLSRCFQF------AISKHIARYIT-SK
47 Molluscum_contagiosum_virus_subtype_1            9.1%  -----------------------------------------------LREVQTT-----L------AVARTLERVLN-EH
48 Monkeypox_virus                                 10.5%  -----------------------------------------------SQDVDKS-----I------EIAKELERLIN-SR
49 Myxoma_virus                                    10.7%  -----------------------------------------------TNDVLTS-----I------KIAKELERVIN-TK
50 Noumeavirus                                     12.5%  -----------------------------------------------GRTPKDL-----I------EFGKFVGKEIS-KH
51 Orf_virus                                       10.2%  -----------------------------------------------ASDIAFS-----R------RVGRCLERVIN-EH
52 Organic_Lake_phycodnavirus_1                    18.2%  ------------------------------------------NTLPDKQLLDMT-----I------EVAKEAGHLCT-SF
53 Orpheovirus_LCC2                                12.8%  -----------------------------------------------IIDKKDC-----N------EAGFKLAKEVS-EY
54 Ostreococcus_lucimarinus_virus_1                16.7%  -----------------------------------------------EDAIAYS-----W------EVGERAAEECS-AL
55 Ostreococcus_tauri_virus_OtV5                   16.7%  -----------------------------------------------EEAIAYS-----W------EVGERAAEECS-AL
56 Pacmanvirus_A23                                  9.1%  YERNLVLLNEEFEGVPCEAEPSESNPRSVEFKKRRVAERLKWWNQMVAITMDVM-----N------TLKEEVSDFLL-AN
57 Pandoravirus_dulcis                              9.2%  -----------------------------------------------REGVEVA-----L------QLGVAASDYIT-TK
58 Pandoravirus_inopinatum                          8.8%  -----------------------------------------------KRATTNG-----V------SLCCTDNHRIY-AR
59 Pandoravirus_salinus                            10.5%  -----------------------------------------------REGVEVA-----L------QLGVAASDYIT-TK
60 Paramecium_bursaria_Chlorella_virus_CVK2        15.4%  -----------------------------------------------VHDMDEQ-----F------KMAKWLAGEIT-KD
61 Paramecium_bursaria_chlorella_virus_MT325       15.1%  -----------------------------------------------QEDINDH-----F------EVAKWLAGEIT-KE
62 Paramecium_bursaria_Chlorella_virus_NYs1        14.2%  -----------------------------------------------IHDMDEQ-----F------KMAKWLAGEIT-KD
63 Phaeocystis_globosa_virus                       16.9%  ------------------------------------------KRIINKQALIYT-----I------ELAKQAGELAT-KF
64 Phaeocystis_globosa_virus_14T                   16.8%  ------------------------------------------KRIINKQALIYT-----I------ELAKQAGELAT-KF
65 Pithovirus_massiliensis                         11.6%  -----------------------------------------------CSNYADA-----M------KRGEELAKEVS-DI
66 Pithovirus_sibericum                            11.8%  -----------------------------------------------CSNYADA-----M------KRGEELAKEVS-DI
67 Port-miou_virus                                 12.4%  -----------------------------------------------GRTPKDL-----I------EFGKFVGKEIS-KH
68 Powai_lake_megavirus                            60.0%  ------------------------------------------QERTDKEALIQT-----I------AKCQRAAKLIN-QN
69 Scale_drop_disease_virus                        12.8%  -----------------------------------------------KSSFEEL-----W------NLATQASKTVS-SF
70 Short-finned_eel_ranavirus                      13.1%  -----------------------------------------------AKTPAKL-----W------DKAVAVAKAVS-SV
71 Singapore_grouper_iridovirus                    13.3%  -----------------------------------------------IQNVREL-----W------EKAEAVAKAVS-AQ
72 Skunkpox_virus                                  11.0%  -----------------------------------------------SQDVDIS-----I------EIAKELEKLIN-SR
73 Spodoptera_frugiperda_ascovirus_1a              10.9%  -----------------------------------------------PASNQDP-----W------TYVRELATHIT-SK
74 Testudo_hermanni_ranavirus                      13.2%  -----------------------------------------------AKTPAEL-----W------DKAVVVAKAVS-SV
75 Tiger_frog_virus                                13.1%  -----------------------------------------------AKTPAEL-----W------DKAVAVAKAVS-SV
76 Tokyovirus_A1                                   12.9%  -----------------------------------------------GKTPREL-----I------EFGNMVGKEVS-KH
77 Trichoplusia_ni_ascovirus_2c                    10.4%  -----------------------------------------------SDDLEDP-----W------HYLQSLGDEIT-SN
78 Tunisvirus_fontaine2                            12.2%  -----------------------------------------------GRTPKDL-----I------EFGEFVGAEIS-KH
79 Vaccinia_virus                                  10.6%  -----------------------------------------------SQDVDKS-----I------EIAKELERLIN-NR
80 Variola_virus                                   10.3%  -----------------------------------------------SQDVDKS-----I------EIAKELERLIN-SR
81 Volepox_virus                                   10.8%  -----------------------------------------------SQDVDKS-----I------EIAKELERLIN-SR
82 Wiseana_iridescent_virus                        14.4%  -----------------------------------------------IKDSAKL-----W------DHAIFVANEIS-KL
83 Yaba_monkey_tumor_virus                         10.8%  -----------------------------------------------TQDVDTS-----I------IIAKELENIIN-KK
84 Yellowstone_lake_phycodnavirus_1                17.0%  -----------------------------------------------QEAIDYS-----W------QLGEQAAEQCT-KL
   consensus/100%                                         ................................................................................
   consensus/90%                                                                 ..........................s....     .      ths..htt.hs t.
   consensus/80%                                                                                   .....t.s.tt.     h      ths..hsc.ls p.
   consensus/70%                                                                                        tpshtph     h      phupthuc.ls ph

                                                   3041         :         .         .         .         .         1         .         .    31
 1 Acanthamoeba_castellanii_mamavirus             100.0%  VPKP-QS-------IVYEK-TLHPFILVAKKKYVGLLF-----------EKSPDK----YFLKSMGIVLK----RRDNAP
 2 Acanthamoeba_polyphaga_mimivirus                98.6%  VPKP-QS-------IVYEK-TLHPFILVAKKKYVGLLF-----------EKSPDK----YFLKSMGIVLK----RRDNAP
 3 Acanthamoeba_polyphaga_moumouvirus              56.0%  VPKP-QS-------IVYEK-TFHPFILVAKKKYVGLLF-----------EKNPDK----YFLKSMGIVLK----RRDNAP
 4 Amsacta_moorei_entomopoxvirus                   10.1%  LTDN-FE-------FEYEK-MYYWMILLAKKKYIGEVV--------------INMDPLQLMDDTKGTALI----RRDCTV
 5 Anomala_cuprea_entomopoxvirus                    9.7%  IFPDIFE-------FEFED-VNCWIVFTSKKKYLAEHVKLITDDIGLKKIKDDIIQ---LNFINKGTLLV----RRDYCK
 6 ASFV_BAV71                                       9.7%  NGTSYLR-------MAYEE-VLFPVCFTGKKKYYGIAH-----------VNTPNFNT--KELFIRGIDII----KQGQTK
```

```
 7 ASFV_E75                                      9.7%   NGTSYLR-------MAYEE-VLFPVCFTGKKKYYGIAH------------VNTPNFNT--KELFIRGIDII----KQGQTK
 8 Brazilian_marseillevirus                     12.6%   LVSP-MN-------LQFEK-LCKKFIIFSKKRYYTFIV------------DENGKV----IAIDKKGIVLT----RRDNCL
 9 Cafeteria_roenbergensis_virus_BV-PW1         20.9%   LPFP-HD-------LEYEK-TFWPYLILTKKRYVGNKY------------EFDRNK----YKLDYMGIVLK----RRDNAP
10 Cannes_8_virus                               13.3%   LVAP-MN-------LQFEK-LCKDFLLFSKKRYYTLIV------------DKSGKV----IATDKKGIILT----RRDNCL
11 Catovirus_CTV1                               24.3%   LNGP-MQ-------MAYEK-VLHPFIILTKKRYVGNLY------------ESDPNK----FYTKSMGIVLK----RRDNAP
12 Cedratvirus_A11                              12.1%   FPFH-LN-------VELEK-VGRAFYI-KKKMYAFWGT------------SPQGVFCAP-GKIMHKGTTLT----RRDNCL
13 Choristoneura_biennis_entomopoxvirus         10.5%   LSKN-FN-------FEYEK-MYIWMLLAKKKYIGEVV-------------SSMNPLQLISDSKGTALI----RRDCTE
14 Common_midwife_toad_ranavirus                13.2%   FPPP-IS-------LEFEQVIYTKFLILGKKRYIYLSC------------DRDGNSS---GKMGFRGVLMA----RRDNSG
15 Cowpox_virus                                 10.5%   VLFNNFK-------IEFEA-VYKNLIMQSKKKYTTMKY------------SASSNSKSV-PERINKGTSET----RRDVSK
16 Diadromus_pulchellus_ascovirus_4a            12.7%   YPKP-MK-------IEFEETIYYKFMILTKKRYMYYSC------------KRTGEIS---KKIGQKGVLLA----RRDHSQ
17 Ectromelia_virus                             10.5%   VLFNNFK-------IEFEA-VYKNLIMQSKKKYTTMKY------------SASSNSKSV-PERINKGTSET----RRDVSK
18 Emiliania_huxleyi_virus_145                  14.6%   FRED-IE-------LEMEK-VYSGFLLITKKRYFGGMH------------EPNKAGDVVFSKVDAKGVELV----RRDNCP
19 Emiliania_huxleyi_virus_86                   14.6%   FRED-IE-------LEMEK-VYSGFLLITKKRYFGGMH------------EPNKAGDVVFSKVDAKGVELV----RRDNCP
20 European_catfish_virus                       13.1%   FPPP-IS-------LEFEQVIYTKFLILGKKRYIYLSC------------DRDGNSS---GKMGFRGVLMA----RRDNSG
21 Faustovirus_D3                                8.8%   TGTRYLN-------MAYEE-VGFPTAWCGKKKYFMTAH------------IKTINFEP--DEYFLRGVEIV----KQGVNA
22 Faustovirus_E12                               8.9%   TGTRYLN-------MAYEE-VGFPTAWCGKKKYFMTAH------------IETINFEP--DEYFLRGVEIV----KQGVNQ
23 Faustovirus_E24                               8.9%   TGTRYLN-------MAYEE-VGFPTAWCGKKKYFMTAH------------IETINFEP--DEYFLRGVEIV----KQGVNQ
24 Faustovirus_E9                                9.0%   TGTRYLN-------MAYEE-VGYPTAWCGKKKYFMTAH------------IKTINFDP--DEYFLRGVEIV----KQGVNA
25 Faustovirus_Liban                             9.0%   TGTRYLN-------MAYEE-VGFPTAWCGKKKYFMTAH------------IETINFEP--DEYFLRGVEIV----KQGVNQ
26 Feldmannia_species_virus                     13.4%   FGHP-VK-------LEYEK-IYFPFLLISKKRYACMSY------------DRPDSE----PKMSTSGLVTV----RRDNAK
27 Frog_virus_3                                 13.2%   FPPP-IS-------LEFEQVIYTKFLILGKKRYIYLSC------------DRDGNSS---GKMGFRGVLMA----RRDNSG
28 Heliothis_virescens_ascovirus_3e             11.3%   LRKP-MV-------IEAEDDIHAKVLFLGKKCYIGRKL------------FRDGSVS---RELDWHGVITV----RRDHSQ
29 Hokovirus_HKV1                               20.3%   QPPP-HN-------LQYEK-TLWPFAILSKKRYVGYLY------------ERNPDI----CFQKSMGIVLK----RRDNAI
30 Infectious_spleen_and_kidney_necrosis_virus  12.7%   FERP-VR-------LEFEQCIYTKFIIFTKKRYVYRAF------------TRDGK-----QRTGSKGVMLS----RRDSAM
31 Insectomime_virus                            12.3%   LVSP-MN-------LQFEK-LCLRFIIFSKKRYYTLIA------------NKDGKI----VGIDKKGIVLT----RRDNCL
32 Invertebrate_iridescent_virus_22             13.4%   FPPP-MK-------LEFEEAVYTKFLILTKKRYMYQTA------------LRDGTIK---PEIGKKGVVLN----RRDNSA
33 Invertebrate_iridescent_virus_3              14.2%   FPPP-IK-------LEFEEAIYSKFLILTKKRYLYQTA------------LRDGTIK---KEIGKRGVVLN----RRDNSG
34 Kaumoebavirus                                10.2%   TGYGFLE-------MAYEE-ILFPYCLIARKKYFGLEH------------LGVPNFTK--PKQFIKGLDFK----KRGASP
35 Klosneuvirus_KNV1                            15.8%   ----------------------------------------------------------------------------------
36 Kurlavirus_BKC-1                             12.6%   LVSP-MN-------LQFEK-LCKRFIIFSKKRYYTIIV------------DETGEC----IAIDKKGIVLT----RRDNCL
37 Lausannevirus                                12.4%   LVSP-MN-------LQFEK-LCKRFIIFSKKRYYTIIV------------NEDGEC----IAIDKKGIVLT----RRDNCL
38 Lumpy_skin_disease_virus_NI-2490             10.5%   ILFSNFK-------IEFEA-VYKNLIMQSKKKYTTLKY------------LPSFTNESI-PERINKGTSET----RRDVSK
39 Lymphocystis_disease_virus                   13.5%   FPMP-MK-------LEFENVVYVKFLILSKKRYMYVSC------------NKSGCVD---SKLSSKGVLLA----RRDNAG
40 Marseillevirus_marseillevirus_T19            13.3%   LVAP-MN-------LQFEK-LCKDFLLFSKKRYYTLIV------------DKSGKV----IATDKKGIILT----RRDNCL
41 Megavirus_chiliensis                         60.1%   VPKP-QS-------IVYEK-TFHPFILVAKKKYVGLLF------------ETNPNK----YFLKSMGIVLK----RRDNAP
42 Megavirus_courdo7                            60.4%   VPKP-QS-------IVYEK-TFHPFILVAKKKYVGLLF------------ETNPNK----YFLKSMGIVLK----RRDNAP
43 Megavirus_ursino                             60.1%   VPKP-QS-------IVYEK-TFHPFILVAKKKYVGLLF------------ETNPNK----YFLKSMGIVLK----RRDNAP
44 Melbournevirus                               13.3%   LVAP-MN-------LQFEK-LCKDFLLFSKKRYYTLIV------------DKSGKV----IATDKKGIILT----RRDNCL
45 Micromonas_pusilla_virus_SP1                 16.3%   FKKP-NN-------LELEK-VYWPYFLYSKKRYAAKLW------------TKGKDDQMHMDYIDIKGLQVV----RRDNTP
46 Mollivirus_sibericum                          9.9%   FNSPDLT-------LGWEK-VYYPYNLLGKKKYVGVQW------------LVPCEPK---SDQDNKGVVAK----RRDTWK
47 Molluscum_contagiosum_virus_subtype_1         9.1%   VLFENFR-------VEFEA-IYQNLILQSKKKYSTTKY-------AADHRAGDA----PEHVSKGTSET----RRDVSR
48 Monkeypox_virus                              10.5%   VLFDNFK-------IEFEA-VYKNLIMQSKKKYTTMKY------------SASSNSKSV-PERINKGTSET----RRDVSK
49 Myxoma_virus                                 10.7%   VLFDNFK-------IEFEA-VYKNLIMQSKKKYTTLKY------------VPSSTQNSI-PVRINKGTSET----RRDVSK
50 Noumeavirus                                  12.5%   LVSP-MN-------LQFEK-LCKRFIIFSKKRYYTIIV------------DETGEC----IAIDKKGIVLT----RRDNCL
51 Orf_virus                                    10.2%   VLFANFK-------VEFEA-VYCNLIMQSKKKYTTIKF-----------AVSDGGG----SERVSKGTSET----RRDVAP
52 Organic_Lake_phycodnavirus_1                 18.2%   LKEP-HD-------LEYEK-TFMPFCLLSKKRYVGMLY------------EDDINS----CSRKSMGIVLK----RRDNAP
53 Orpheovirus_LCC2                             12.8%   INMNPVK-------LEFEK-AMYVFLVFKKKKYAAVLI------------DKHGEPMWGRNDRLTKGIILA----RRDNCQ
54 Ostreococcus_lucimarinus_virus_1             16.7%   FKKP-NN-------LELEK-VYWPYFLYSKKRYAAKLW------------TKGKDDKMHMDYIDIKGLQVV----RRDNTP
55 Ostreococcus_tauri_virus_OtV5                16.7%   FKKP-NN-------LELEK-VYWPYFLYSKKRYAAKLW------------TKGKDGKMHMDYIDIKGLQVV----RRDNTP
56 Pacmanvirus_A23                               9.1%   NETCFLN-------MAYEE-VGYPTVFCGKKKYFMTAH------------LKEINFYP--KELFIRGIDII----KQGQAK
57 Pandoravirus_dulcis                           9.2%   FPDQ-II-------LDTEK-AYWPYVLFRKKRYVGRMW------------TLEGKP----PYIDAKGVEVK----RRDNWA
58 Pandoravirus_inopinatum                       8.8%   VGPTWGNRIWRRKGTESQESAAPPFTIQSAGDILATGA------------RDPSTVVQFTAACSKGVAI-----EGGNLP
59 Pandoravirus_salinus                         10.5%   FPDQ-II-------LDTEK-AYWPYVLFRKKRYVGRMW------------TLDGKP----PYIDAKGVEVK----RRDNWA
60 Paramecium_bursaria_Chlorella_virus_CVK2     15.4%   FRAP-ND-------LEFEK-IYYPYILYSKKRYAAVKF------------EEPDET----GKVDVKGLALV----RRDFSP
61 Paramecium_bursaria_chlorella_virus_MT325    15.1%   YRAP-ND-------LEFEK-IYYPYILYSKKRYAAIKY------------EDPEEK----GKVDVKGLALV----RRDFSP
62 Paramecium_bursaria_Chlorella_virus_NYs1     14.2%   FRAP-ND-------LEVSS------KNSKRELYNFVIY------------DPDFTFLNIPVCSSRKSTIHTSYTPKRDTLR
63 Phaeocystis_globosa_virus                    16.9%   LKKP-HD-------LEYEK-TFWPFNLLSKKRYDGMLY------------ENDPEY----CKLKSMGNVLK----RRDNAP
64 Phaeocystis_globosa_virus_14T                16.8%   LKKP-HD-------LEYEK-TFWPFNLLSKKRYDGMLY------------ENDPEY----CKLKSMGNVLK----RRDNAP
65 Pithovirus_massiliensis                      11.6%   FPPA-LN-------LGLEK-VGRMFCI-KKKKYAFWPV------------NRDGTFKDE-VDIIHKGTIKA----RRDNCQ
66 Pithovirus_sibericum                         11.8%   FPPA-LN-------LGLEK-VGRMF-CIKKKKYAFWPV------------NRDGTFKNE-AEIIHKGTIKA----RRDNCQ
67 Port-miou_virus                              12.4%   LVSP-MN-------LQFEK-LCKRFIIFSKKRYYTIIV------------NEDGEC----IAIDKKGIVLT----RRDNCL
68 Powai_lake_megavirus                         60.0%   VPKP-QS-------IVYEK-TFHPFILVAKKKYVGLLF------------ETNPNK----YFLKSMGIVLK----RRDNAP
69 Scale_drop_disease_virus                     12.8%   FGDP-VK-------LEFEQTIYVKFIIFSKKRYIYRQK------------HISGK-----ESIGSKGVLLS----RRDNAM
70 Short-finned_eel_ranavirus                   13.1%   FPPP-IS-------LEFEQVIYTKFLILGKKRYIYLSC------------DRDGSSS---GKMGFRGVLMA----RRDNSG
71 Singapore_grouper_iridovirus                 13.3%   FPRP-IT-------LEFEKVIYTKFLILGKKRYIYLSC------------DKNGFST---GQMGYRGVLMA----RRDNSG
72 Skunkpox_virus                               11.0%   VLFNNFK-------IEFEA-VYKNLIMQSKKKYTTMKY------------SASSNSKSI-PERINKGTSET----RRDVSK
73 Spodoptera_frugiperda_ascovirus_1a           10.9%   LREP-MV-------IEAEDDIHAKVLFLGKKCYIGRKL------------FRDGSVA---RDLDWHVVITV----RRDHSE
74 Testudo_hermanni_ranavirus                   13.2%   FPPP-IS-------LEFEQVIYTKFLILGKKRYIYLSC------------DRDGNSS---GKMGFRGVLMA----RRDNSG
75 Tiger_frog_virus                             13.1%   FPPP-IS-------LEFEQVIYTKFLILGKKRYIYLSC------------DRDGNSS---GKMGFRGVLMA----RRDNSG
76 Tokyovirus_A1                                12.9%   LVAP-MN-------LQFEK-LCKDFFLLSKKRYYTKIV------------NEKGEV----IAIDKKGIILT----RRDNCL
77 Trichoplusia_ni_ascovirus_2c                 10.4%   LRKP-MR-------IESED-NISDVLFLGKKSYICRKL------------LKDGGIS---EKLDYHGGIAV----RRDHSG
78 Tunisvirus_fontaine2                         12.2%   LVSP-MN-------LQFEK-LCLRFIIFSKKRYYTLIA------------NKDGKI----VGIDKKGIVLT----RRDNCL
79 Vaccinia_virus                               10.6%   VLFNNFK-------IEFEA-VYKNLIMQSKKKYTTMKY------------SASSNSKSV-PERINKGTSET----RRDVSK
80 Variola_virus                                10.3%   VLFNNFK-------IEFEA-VYKNLIMQSKKKYTTMKY------------SASSNSKSV-PERINKGTSET----RRDVSK
81 Volepox_virus                                10.8%   VLFNNFK-------IEFEA-VYKNLIMQSKKKYTTMKY------------SASSNSKSI-PERINKGTSET----RRDVSK
82 Wiseana_iridescent_virus                     14.4%   FPPP-MK-------LEFEDAIYTKFLILTKKRYMYQSA------------TRDGTIK---PEIGKRGVVLN----RRDNST
83 Yaba_monkey_tumor_virus                      10.8%   VLFYNFK-------IEFEA-VYKNLIMQSKKKYTTLKY------------SSEYTSNSV-PERVSKGTSET----RRDVSK
84 Yellowstone_lake_phycodnavirus_1             17.0%   FKAP-ND-------LELEK-VYCPYFLYSKKRYAAKMY------------EKNKLGEIAFKKIDVKGLQVV----RRDSCP
   consensus/100%                                       ..................................................................................
   consensus/90%                                        h.....p       hthEt.hhh.hhh.sKK+Yhh..h            ..t.t.......h..hGh..h      +ps.s.
   consensus/80%                                        h..s.hp       lthEp.hhh.hhh.sKK+Yhhh.h            .ts.p.... ..h..+Gh.hh      RRDss.
   consensus/70%                                        h..s hp       lpaEc lhh.hlhhuKK+Yhshhh            ppssp...  .th..+Gl.hs      RRDss.

                                                3121        .           .         :         .          .           .          .       2 32
 1 Acanthamoeba_castellanii_mamavirus          100.0%   IVKIVVG---GIIDNILK-----NRDIDKA------IEYTKIVL---DKLMN-------------GEY--PMDKFIISKTL
 2 Acanthamoeba_polyphaga_mimivirus             98.6%   IVKIVVG---GIIDNILK-----NRDIDKA------IE---YTKIVLDKLMN-------------GEY--PMDKFIISKTL
 3 Acanthamoeba_polyphaga_moumouvirus           56.0%   IVKIVVG---GIIDHILK-----NRDINGA------VE---YTR---NVLSK---LMK-------GEY--PIDKFIISKTL
 4 Amsacta_moorei_entomopoxvirus                10.1%   IHKTILKNTINILKDFLTN----DNTGINI------NV---KIN---DYLSS---AFKNIIENIQNL--DINDFKKSVKY
 5 Anomala_cuprea_entomopoxvirus                 9.7%   FHKDTYK----NIVDIIK-----CG-LKSN---KNVEL---EIE---NYVIN---VIDDTLANIRNL--NIDDFVKTMTY
 6 ASFV_BAV71                                    9.7%   LTKTIGT---RIMEESMK-----LRRPEDH------RP---PLI---EIVKT---VLKDAVVNMKQW--NFEDFIQTDAW
 7 ASFV_E75                                      9.7%   LTKTIGT---RIMEESMK-----LRRPEDH------RP---PLI---EIVKT---VLKDAVVNMKQW--NFEDFIQTDAW
 8 Brazilian_marseillevirus                     12.6%   LVRDLFG---ATVKQVIE-----KEDTQKI------LQ---GVY---DGIYG---MMS-------RSI--PDQKYVITASI
 9 Cafeteria_roenbergensis_virus_BV-PW1         20.9%   IVKEICN---GIINKLIE-----HKDPEGA------YH---FLE---NSLNK---MFN-------NEY--NIKYFLTSKTL
10 Cannes_8_virus                               13.3%   LARDLFG---ETVTSIIA-----KKPQQEI------LQ---NVL---DRIYL---MMA-------RAL--PDQKYVITKSI
11 Catovirus_CTV1                               24.3%   IVKVVVG---GIVDQILN-----KQSSKGA------VDFTKKVL---KDILS-------------SKY--SIDKYIITKTL
12 Cedratvirus_A11                              12.1%   WQRSLFE---MTLMDCLN-----KTSLDDM------FA---HIS---EEVIN---LYR-------RKY--TWSDFVVVKSV
13 Choristoneura_biennis_entomopoxvirus         10.5%   IHKTILKNTIDILKEYLTN----NCTIQDV------NN---KIN---NYLMF---TFKNIIENIQNL--DINEFKKSVKY
14 Common_midwife_toad_ranavirus                13.2%   LARKAYS---ITAQALLE-----DRDPWAD------LT---PLM---KDMYT-------------KNC--SLRDFVITKQV
15 Cowpox_virus                                 10.5%   FHKNMIKIYKTRLSEMLS-----EGRMNSNQVCIDILR---SLE---TDLRS---EFDS------RSS--PLELFMLSRMH
16 Diadromus_pulchellus_ascovirus_4a            12.7%   FVKQNYE---TTVMNVFA-----SKSKEDV------IS---DVFEMGLSCMR-------------RQL--DDGVFRITKAV
17 Ectromelia_virus                             10.5%   FHKNMIKIYKTRLSEMLS-----EGRMNSNQVCIDILR---SLE---TDLRS---EFDS------RSS--PLELFMLSRMH
18 Emiliania_huxleyi_virus_145                  14.6%   LLKNLYK---KIVDSLVF-----DKDPLKA------IA---SVK---ETLER---VVN-------DEV--PYEEYIITKEL
19 Emiliania_huxleyi_virus_86                   14.6%   LLKNLYK---KIVDSLVF-----DKDPLKA------IA---SVK---ETLER---VVN-------DEV--PYEEYIITKEL
20 European_catfish_virus                       13.1%   LARKAYS---ITAQALLE-----DRDPWAD------LT---PLM---KDMYT-------------KNC--NLMDFVITKQV
```

```
21 Faustovirus_D3                                 8.8%    ITKQLGNEFIAEAMDIHS-----SREPIDI------AR---------DKLVK---FFT------NEW--SVEMFAQTATY
22 Faustovirus_E12                                8.9%    IIKVLGNEFIKEALDIHS-----DKEPLDI------AG---------EKLIK---FFT------HNW--AIDMFAQTATY
23 Faustovirus_E24                                8.9%    IIKVLGNEFIKEALDIHS-----DKEPLDI------AG---------EKLIK---FFT------HNW--AIDMFAQTATY
24 Faustovirus_E9                                 9.0%    ITKQLGNEFIKEAMDIHS-----DREPIDI------AK---------DKLVK---FFT------NEW--SIEMFAQTATY
25 Faustovirus_Liban                              9.0%    IIKVLGNEFIKEALDIHS-----DKEPLDI------AG---------EKLIK---FFT------HNW--SIDMFAQTATY
26 Feldmannia_species_virus                      13.4%    VVRDCAN---GVISILME-----GRGQGDV------VEYVKTVL---SKLEN------------SEI--GVEDLTISNEL
27 Frog_virus_3                                  13.2%    LARKAYS---ITAQALLE-----DRDPWAD------LT---PLM---KDMYT------------KNC--SLRDFVITKQV
28 Heliothis_virescens_ascovirus_3e              11.3%    YVKDVYR---KAVHRVFA-----DCTMEQF------KH---TIF---EQALT---LMQ------RRV--SYERLTKTSEV
29 Hokovirus_HKV1                                20.3%    IVKIVVG---GVVDQLLN-----EHSPQGA------LN---YVE---NELNK---ILS------NKY--LLEKFVISKTL
30 Infectious_spleen_and_kidney_necrosis_virus   12.7%    CARNTYA---AIMNTILE-----GSADVPF------IA---ACMMH-DMMIP------------GAL--QDDDFVLTKSV
31 Insectomime_virus                             12.3%    LVRELFG---ETVKRVIN-----KDERSEI------LH---GVY---NGIYG---MMS------RSI--PDQKYVITASI
32 Invertebrate_iridescent_virus_22              13.4%    FIRKTYE---NVVKIIFENTEDKDKLQSKV------IK---SLL---TDVNA---MFN------HQC--LVEDFIITKST
33 Invertebrate_iridescent_virus_3               14.2%    FIRKIYQ---NMVDSIFNHVAGEGRDLKSV-----VLD---VVT---ADICA---LFN------HQF--PVDDFVITKST
34 Kaumoebavirus                                 10.2%    MLKMVCQ---SILDTALS-----MDCSKTL------KQ---IVE---DKLVE---IKT------TDWSNNIDLFAKMDTC
35 Klosneuvirus_KNV1                             15.8%    --------------------------------------------------------------------------------
36 Kurlavirus_BKC-1                              12.6%    LVRELFG---ETVDRVIN-----KEDRQKI------IQ---GVF---DGIYG---MMS------RSI--SDKKYVITASI
37 Lausannevirus                                 12.4%    LVRELFK---KTVEQVIN-----KEDRQKI------VQ---GVF---DGIYG---MMS------RSI--ADQKYVITASI
38 Lumpy_skin_disease_virus_NI-2490              10.5%    FHKYMIRIYKTRLLQMLS-----GGNMSSVQVCVEILS---SLE---SDLQI---EFET-----KTA--PLDMFLLSRTH
39 Lymphocystis_disease_virus                    13.5%    CLKNIYN---NSVLAIMH-----KKNIFET------IL---NIV---SDVIR------------GVL--PIEDFVITKSI
40 Marseillevirus_marseillevirus_T19             13.3%    LARDLFG---ETVTSIIA-----KKPQQEI------LQ---NVL---DRIYL---MMA------RAL--PDQKYVITKSI
41 Megavirus_chiliensis                          60.1%    IVKIVVG---GIIDYILK-----NRDIDKA------VE---YTR---NVLSK---LMN------GEY--PIDKFIISKTL
42 Megavirus_courdo7                             60.4%    IVKIVVG---GIIDYILK-----NRDIDKA------VE---YTR---NVLSK---LMN------GEY--PIDKFIISKTL
43 Megavirus_ursino                              60.1%    IVKIVVG---GIIDYILK-----NRDIDKA------VE---YTR---NVLSK---LMN------GEY--PIDKFIISKTL
44 Melbournevirus                                13.3%    LARDLFG---ETVTSIIA-----KKPQQEI------LQ---NVL---DRIYL---MMA------RAL--PDQKYVITKSI
45 Micromonas_pusilla_virus_SP1                  16.3%    HVREVCK---ELLDVVLT-----SSDPGPP------RE---LAK---ERAIE---LLS------GDV--PHEKLILSQSL
46 Mollivirus_sibericum                           9.9%    GMRNTFK---KCATAIMRPGKQYDTVTVPV-VDDGGMTRTVEIFLPMEELSH---LLAKVVV--DEL--DVPDYETSKSL
47 Molluscum_contagiosum_virus_subtype_1          9.1%    FHKLMISRYKAQLVALLA-----EARLSSRQVCIEMLR---SLE---VDLFA---EFAA-----RAQ--PLEMFLLSRMH
48 Monkeypox_virus                               10.5%    FHKNMIKTYKTRLSEMLS-----EGRMNSNQVCIDILR---SLE---TDLRS---EFDS-----RSS--PLELFMLSRMH
49 Myxoma_virus                                  10.7%    FHKYMIKIYKTRILEMLS-----DGTSSSLQVCINILK---SLE---SDLLI---EFET-----RKA--PLDLFLLSRTH
50 Noumeavirus                                   12.5%    LVRELFG---ETVDRVIN-----KEDRQKI------IQ---GVF---DGIYG---MMS------RSI--SDKKYVTTASI
51 Orf_virus                                     10.2%    FHKLMIRKYKDMLCRALA-----EEGSGNV--GVEILR---SLE---DELTF---EFEA-----RSM--PLDWFLLSRTH
52 Organic_Lake_phycodnavirus_1                  18.2%    IVKDVYG---GIIDILMK-----EKDIEKS------IL---FLD---KMLEN---IID------ENI--IMDKLVVTKSL
53 Orpheovirus_LCC2                              12.8%    FLRDIYV---ECLDVILM-----EKPIWDT------MD---IIV---DKIEK---LVN------GEI--DWGDLTIIRGI
54 Ostreococcus_lucimarinus_virus_1              16.7%    HVREVCK---ELLDVVLT-----SSDPGPP------KE---LAK---ERAIE---LLS------GDV--PNEKLILSQGL
55 Ostreococcus_tauri_virus_OtV5                 16.7%    HVREVCK---ELLDVILT-----SSDPGPP------KE---LAK---ERAIE---LLS------GDV--PNDKLILSQGL
56 Pacmanvirus_A23                                9.1%    ISKQLGE---EFMREALS-----PENEREL------IE---IAE---DKIRK---FYR------TKL--DPNLFSLIARY
57 Pandoravirus_dulcis                            9.2%    GMRKTYK---ACLEAMME-----RMDINAV------KD---IVLRLVCDLKG------------DRV--SLDDYKISKSL
58 Pandoravirus_inopinatum                        8.8%    FVEALGLRTEDQVDAFVELYGYWLGDGWLD------VSCQAIAF---SPVKTADSAYL------ASLFARLPLPMLTKDT
59 Pandoravirus_salinus                          10.5%    GMRKTYK---ACLEAMME-----RMDINAV------KD---IVLRLVCDLKG------------DRV--SLDDYKISKSL
60 Paramecium_bursaria_Chlorella_virus_CVK2      15.4%    ITREILK---ESLDTILY-----KKDTPTA------VS---ETL---ERIRK---VLD------NEY--PMEKFMMSKTL
61 Paramecium_bursaria_chlorella_virus_MT325     15.1%    ITREILK---ESLDTILF-----AKDTPTA------VK---DTR---EKIRK---VLD------NEY--PMEKFVMSKTL
62 Paramecium_bursaria_Chlorella_virus_NYs1      14.2%    SSLRIR----MKKGKLMS-----KVSRWSV-----------ETF-----LRS------------RGK--SMEKFMMSKTL
63 Phaeocystis_globosa_virus                     16.9%    IVKDIYG---GVVGILMK-----DKSLPKS------IK---FVK---ESVQN---MID------EKY--PIEKLLVTKAL
64 Phaeocystis_globosa_virus_14T                 16.8%    IVKDIYG---GVVGILMK-----DKSLPKS------IK---FVK---ESVQN---MID------EKY--PIEKLLVTKAL
65 Pithovirus_massiliensis                       11.6%    WQQNLFY---TTLMDCLN-----RQSLKEM------FE---NIS---AQVIE---TYQ------RKF--SWQDFVIVKSL
66 Pithovirus_sibericum                          11.8%    WQQNLFY---TTLMDCLN-----RQGLKEM------FE---NIS---AQVIE---TYQ------RKF--SWQDFVVVKSL
67 Port-miou_virus                               12.4%    LVRELFK---KTVEQVIN-----KEDRQKI------VQ---GVF---DGIYG---MMS------RSI--ADQKYVITASI
68 Powai_lake_megavirus                          60.0%    IVKIVVG---GIIDYILK-----NRDIDKA------VE---YTR---NVLSK---LMN------GEY--PIDKFIISKTL
69 Scale_drop_disease_virus                      12.8%    CARTVYK---QLMDHVLT-----SNDQSDT------CT---LMS---DCIID---IMRH-----GVL--SDNMFVITRSI
70 Short-finned_eel_ranavirus                    13.1%    LARKAYS---ITAQALLE-----DRDPWTD------LT---PLM---KDMYT------------KNC--SLRDFVITKQV
71 Singapore_grouper_iridovirus                  13.3%    LARRAYR---MVAQALLE-----KRDPWTD------LN---DLL---SDMYS------------LKC--PVKDYTITKQV
72 Skunkpox_virus                                11.0%    FHKNMIKIYKTRLSQILS-----EGSMNSTQVCIDILR---SLE---VNLRS---EFDN-----RTS--PLEMFMLSRTH
73 Spodoptera_frugiperda_ascovirus_1a            10.9%    YVKSAYK---PPVYKVFA-----DCTHDQF------VT---SIA---ESCLR---LMR------RTV--PCEMLTKTSEV
74 Testudo_hermanni_ranavirus                    13.2%    LARKAYS---ITAQALLE-----DRDPWAD------LT---PLM---KDMYT------------KNC--SLRDFVITKQV
75 Tiger_frog_virus                              13.1%    LARKAYS---ITAQALLE-----DRDPWAD------LT---PLM---KDMYT------------KNC--SLRDFVITKQV
76 Tokyovirus_A1                                 12.9%    LARDLFG---ESVTSLIE-----KRPQQEI------LH---NVL---DRIYL---MMA------RAL--PDQKYVITKSI
77 Trichoplusia_ni_ascovirus_2c                  10.4%    FVKSTHN---KVVHAIFA-----DVSLQNV------KH---VIF---EECLK---LMR------RKI--PIDELTKTSEV
78 Tunisvirus_fontaine2                          12.2%    LVRELFG---ETVKRVIN-----KDERSEI------LH---GVY---NGIYG---MMS------RSI--PDQKYVITASI
79 Vaccinia_virus                                10.6%    FHKNMIKTYKTRLSEMLS-----EGRMNSNQVCIDILR---SLE---TDLRS---EFDS-----RSS--PLELFMLSRMH
80 Variola_virus                                 10.3%    FHKNMIKIYKTRLSEMLS-----EGRMNSNQVCIDILR---SLE---TDLRS---EFDS-----RSS--PLELFMLSRMH
81 Volepox_virus                                 10.8%    FHKNMIKIYKTRLSEILS-----EGSMNSTQVCIDILR---SLE---VNLRS---EFDN-----RTS--PLEMFMLSRTH
82 Wiseana_iridescent_virus                      14.4%    FIRNTYE---NIVKIIFE-----NTHTTNI-LKEKVLS---SLI---QDINE---MFN------HSF--PIEDFVITKST
83 Yaba_monkey_tumor_virus                       10.8%    FHKEMIKTYKTMILDMLS-----NCSMTSIQVCVDVLK---SLE---SDLKI---EFDV-----RSA--PLDMFLLSRTH
84 Yellowstone_lake_phycodnavirus_1              17.0%    YVRETLK---QLLNMVLE-----SDDPKPA------VN---FAKQSAKDLKA------------GLV--PIEKLLLSKQL
   consensus/100%                                         ................................................................................
   consensus/90%                                          hh+.hht.....h..hht     tt...t.......ht   .h.   t.h..   ....     tt.  s.p.hhhotth
   consensus/80%                                          hh+.hht...thh.thhp     ptp..t.      hp   .h.   ptl.t   ....     tph  s.p.ahhotph
   consensus/70%                                          hh+phht   thlptllp     pps..ph      hp   .l.   pplhp   .ht      pph  shccahlocsh

                                                      3201         .         .         .         .         :         .         .         . 32
 1 Acanthamoeba_castellanii_mamavirus           100.0%    KSR-----------------------YK-------------------------------KPSTIAHKVLAD-RMAVRDPG
 2 Acanthamoeba_polyphaga_mimivirus              98.6%    KSR-----------------------YK-------------------------------KPSTIAHKVLAD-RMAVRDPG
 3 Acanthamoeba_polyphaga_moumouvirus            56.0%    KAR-----------------------YK-------------------------------KPSTIAHKVLAD-RMAIRDPG
 4 Amsacta_moorei_entomopoxvirus                 10.1%    SGV-----------------------YK-------------------------------DPNYPIELCVKEYNLKNPND-
 5 Anomala_cuprea_entomopoxvirus                  9.7%    SGK-----------------------YK--------------------------------DKNNIIDNIVK-NYNRNNPN
 6 ASFV_BAV71                                     9.7%    RPD--------------------------------------------KDNKAVQIFMSRMHARREQLKKHG-AAASQFA-
 7 ASFV_E75                                       9.7%    RPD--------------------------------------------KDNKAVQIFMSRMHARREQLKKHG-AAASQFA-
 8 Brazilian_marseillevirus                      12.6%    KAL---------------------DEYK------------------------------NDGKGLVNVALAR-KMKKRGE-
 9 Cafeteria_roenbergensis_virus_BV-PW1          20.9%    KMK---------------------ESYA-------------------------------DWKRIAHVVLSE-RIGERDPG
10 Cannes_8_virus                                13.3%    KAL---------------------DEYK------------------------------NEGKSLSNVALAK-KMQSRGE-
11 Catovirus_CTV1                                24.3%    RGP-----------------------------------GLNFNERMLESKKEKGERYYADRNTIVHAVLAD-RIADRDYG
12 Cedratvirus_A11                               12.1%    GEE-----------------------YK--------------------------------VNTNAMAIFKE-YMAERGC-
13 Choristoneura_biennis_entomopoxvirus          10.5%    TGI-----------------------YK-------------------------------DPNFYIELCVKKYNSKNPND-
14 Common_midwife_toad_ranavirus                 13.2%    GSW----CRECAFIEQGADSIVAVGDYKIRDLEKAKAETRKITGTDGGPEYMAVLYKLVMAQLPGHVQLAN-RMIGRGE-
15 Cowpox_virus                                  10.5%    HSN-----------------------YK--------------------------------SADNPNMYLVT-EYNKNNP-
16 Diadromus_pulchellus_ascovirus_4a             12.7%    NDY-----DECRPAFDEESGKWKMGAYA--------------VPAPLEDMTEADHIAVCIGRLPAQVQLEV-RMVANGF-
17 Ectromelia_virus                              10.5%    HSN-----------------------YK--------------------------------SADNPNMYLVT-EYNKNNP-
18 Emiliania_huxleyi_virus_145                   14.6%    RKE---------------------ESYA--------------------------------NPKQEQLMLAK-KISARTNG
19 Emiliania_huxleyi_virus_86                    14.6%    RKE---------------------ESYA--------------------------------NPKQEQLMLAK-KISARTNG
20 European_catfish_virus                        13.1%    GSW----CRECAFIEQGTDSIMAVGDYKIRDLEKAKAETRKITGTDGGPEYMAVLYKLVMAQLPGHVQLAN-RMIGRGE-
21 Faustovirus_D3                                 8.8%    KPA--------------------------------------------KDNKSVKRFHERMTAIFNTFAIDN-PERELYR-
22 Faustovirus_E12                                8.9%    KPA--------------------------------------------KDNKSVKRFHERMTAVFNALPFDD-PNRELYR-
23 Faustovirus_E24                                8.9%    KPA--------------------------------------------KDNKSVKRFHERMTAVFNALPFDD-PNRELYR-
24 Faustovirus_E9                                 9.0%    KPA--------------------------------------------KDNKSVKRFHERMTAIFNTFAIDD-PARELYR-
25 Faustovirus_Liban                              9.0%    KPA--------------------------------------------KDNKSVKRFHERMTAVFNALPFDD-PNRELYR-
26 Feldmannia_species_virus                      13.4%    KKH--------------------PDQYA---------------------------------TPSAHSVLAG-KLNARAK-
27 Frog_virus_3                                  13.2%    GSW----CRECAFIEQGADSIVAVGDYKIRDLEKAKAETRKITGTDGGPEYMAVLYKLVMAQLPGHVQLAN-RMIGRGE-
28 Heliothis_virescens_ascovirus_3e              11.3%    RNV----GDCCTITLCKKTMSWMLGDYK---------VPQHSHHDILYNTKRDALKTYYVSKLPAPARLSV-SLVDRGR-
29 Hokovirus_HKV1                                20.3%    KGT-----------------------YA-------------------------------DRTSIAHVVLAD-RMALRDPG
30 Infectious_spleen_and_kidney_necrosis_virus   12.7%    QDI-------------GNGDDNNQGSYKVRNPQKAQAAATQRVAPDDAEGYAIALRQEMVKQMPAQAQLAE-RMRLQGR-
31 Insectomime_virus                             12.3%    KAL---------------------DEYK------------------------------NDGKGLVNVALAR-KMKKRGD-
32 Invertebrate_iridescent_virus_22              13.4%    GDY--GNLQPQYFLNEKGLHRATLGQYN-------VPFLTEEIKEEEGIQTPEEETNWYLDKLPAHIQLLE-KIKRRGH-
33 Invertebrate_iridescent_virus_3               14.2%    GNY--GDLQPENFVNEKGVPRAMLGQYN-------VPCLTSQVREEERIETEEQEQNWYLDKLPAHIQLLE-KIRRRGQ-
34 Kaumoebavirus                                 10.2%    KQK-----------------------------------------------RDEEGKVVDVKNVKAQTFKARMEEEAKYNP
```

```
35 Klosneuvirus_KNV1                              15.8%   --------------------------------------------------------------------------------
36 Kurlavirus_BKC-1                               12.6%   KAL---------------------EEYK------------------------------NDGKGLVNVALAR-KMKKRGE-
37 Lausannevirus                                  12.4%   KAL---------------------EEYK------------------------------NDGKGLVNVALAR-KMKKRGE-
38 Lumpy_skin_disease_virus_NI-2490               10.5%   HCN-----------------------YK--------------------------------SSDNPNMYLVN-EYNKNND-
39 Lymphocystis_disease_virus                     13.5%   NDW-----------QDKEEDDEYLGAYK------------IRDVKSLKIDDPVEKRALKIAQCPGQVKVAE-KMRLRGI-
40 Marseillevirus_marseillevirus_T19              13.3%   KAL---------------------DEYK------------------------------NEGKSLSNVALAK-KMQSRGE-
41 Megavirus_chiliensis                           60.1%   KSK-----------------------YK-------------------------------KPSTIAHKVLAD-RMAIRDPG
42 Megavirus_courdo7                              60.4%   KSK-----------------------YK-------------------------------KPSTIAHKVLAD-RMAIRDPG
43 Megavirus_ursino                               60.1%   KSK-----------------------YK-------------------------------KPSTIAHKVLAD-RMAIRDPG
44 Melbournevirus                                 13.3%   KAL---------------------DEYK------------------------------NEGKSLSNVALAK-KMQSRGE-
45 Micromonas_pusilla_virus_SP1                   16.3%   SDT-----------------------YK------------------VDGKNVSITSPESVNINQAHVQVVV-KMRERKPG
46 Mollivirus_sibericum                            9.9%   KAK-----------------------YN------------------------------RKKARPPHVVVRD-KIAQRTQG
47 Molluscum_contagiosum_virus_subtype_1           9.1%   HRN-----------------------YK--------------------------------APDNPNVELVN-RYNRENL-
48 Monkeypox_virus                                10.5%   HSN-----------------------YK--------------------------------SADNPNMYLVT-EYNKNNP-
49 Myxoma_virus                                   10.7%   HCN-----------------------YK--------------------------------SIDNPNMYLVN-EYNKNNP-
50 Noumeavirus                                    12.5%   KAL---------------------EEYK------------------------------NDGKGLVNVALAR-KMKKRGE-
51 Orf_virus                                      10.2%   HKN-----------------------FK--------------------------------SPDNPNVALVT-RYNAANA-
52 Organic_Lake_phycodnavirus_1                   18.2%   RSF-----------------------YK-------------------------------NPTRIAHCVLAN-RMGARDQ-
53 Orpheovirus_LCC2                               12.8%   GAN-----------------------YK--------------------------------SDNYFMKVFSD-ELARSGK-
54 Ostreococcus_lucimarinus_virus_1               16.7%   SDT-----------------------YK------------------VGGKNVSVTSSESVNINQSHVQVVT-KMRQRKPG
55 Ostreococcus_tauri_virus_OtV5                  16.7%   SDT-----------------------YK------------------VGGKNVSVTSADSVNINQSHVQVVT-KMRQRKPG
56 Pacmanvirus_A23                                 9.1%   KPN--------------------------------------------KRNVPVLSFVARMREIQAKYPDNP-TFMALYE-
57 Pandoravirus_dulcis                             9.2%   KRD-----------------------YS------------------------------KCKSPPPHVVVRD-KIARRNPG
58 Pandoravirus_inopinatum                         8.8%   RGP------------------GAIGAFI-------TPEPSEVRRRTRYSAWATPHRHYIYTPSWWR-YFAE-QYGHKYS-
59 Pandoravirus_salinus                           10.5%   KRD-----------------------YS------------------------------KCKSAPPHVVVRD-KIARRNPG
60 Paramecium_bursaria_Chlorella_virus_CVK2       15.4%   KTG-----------------------YK--------------------------------NECQPHLTVAN-KIYERTG-
61 Paramecium_bursaria_chlorella_virus_MT325      15.1%   KTG-----------------------YK--------------------------------NEMQPHLIVAN-KIFDRTG-
62 Paramecium_bursaria_Chlorella_virus_NYs1       14.2%   KTG-----------------------YK--------------------------------NECQPHLHVAN-KIFERTG-
63 Phaeocystis_globosa_virus                      16.9%   RGY-----------------------YK-------------------------------NPKQIAHKVLAD-RIGVREQG
64 Phaeocystis_globosa_virus_14T                  16.8%   RGY-----------------------YK-------------------------------NPKQIAHKVLAD-RIGVREQG
65 Pithovirus_massiliensis                        11.6%   GEN-----------------------YK--------------------------------DKNNCMKIFSE-ALAARGA-
66 Pithovirus_sibericum                           11.8%   GEN-----------------------YK--------------------------------DKNNCMKIFSE-ALAARGA-
67 Port-miou_virus                                12.4%   KAL---------------------EEYK------------------------------NDGKGLVNVALAR-KMKKRGE-
68 Powai_lake_megavirus                           60.0%   KSK-----------------------YK-------------------------------KPSTIAHKVLAD-RMAIRDPG
69 Scale_drop_disease_virus                       12.8%   GDI---------------GQDDDTGTYKVRNVAKAKLAA--------GENNTSALRQELVKQLPAQAQLAE-RLRLQGR-
70 Short-finned_eel_ranavirus                     13.1%   GSW----CRECAFIEQGADSIVAVGDYKIRDLEKAKAETRKITGTDGGPEYMAVLYKLVMAQLPGHVQLAN-RMIGRGE-
71 Singapore_grouper_iridovirus                   13.3%   GVW----CREYNFVNQGRDSIVTIGDYKIRDLEKAKAQAKELTKDD-EQSYMPVLYKLIMSQLPGHVQLAN-RMINRGE-
72 Skunkpox_virus                                 11.0%   HSN-----------------------YK--------------------------------SADNPNMFLVT-EYNKNNP-
73 Spodoptera_frugiperda_ascovirus_1a             10.9%   RNL----GDGCQLVLCKKTMSWMWGDYK---------VARHEQHDRLQRENPKWLRDYYVSKLPAPARLSI-LLTDRAR-
74 Testudo_hermanni_ranavirus                     13.2%   GSW----CRECAFIEQGADSIVAVGDYKIRDLEKAKAETRKITGTDGGPEYMAVLYKLVMAQLPGHVQLAN-RMIGRGE-
75 Tiger_frog_virus                               13.1%   GSW----CRECAFIEQGADSIVAVGDYKIRDLEKAKAETRKITGTDGGPEYMAVLYKLVMAQLPGHVQLAN-RMIGRGE-
76 Tokyovirus_A1                                  12.9%   RAL---------------------DEYK------------------------------NEGKGLSNVALAK-KMQTRGE-
77 Trichoplusia_ni_ascovirus_2c                   10.4%   KSIGDGFTLKPSMKNTTTLNSWKLGDYT------------VKKHPQHDTLSKEYLKRYYLEQLPAHVQLEH-KLMERGR-
78 Tunisvirus_fontaine2                           12.2%   KAL---------------------DEYK------------------------------NDGKGLVNVALAR-KMKKRGD-
79 Vaccinia_virus                                 10.6%   HSN-----------------------YK--------------------------------SADNPNMYLVT-EYNKNNP-
80 Variola_virus                                  10.3%   HLN-----------------------YK--------------------------------SADNPNMYLVT-EYNKNNP-
81 Volepox_virus                                  10.8%   HSN-----------------------YK--------------------------------SADNPNMFLVT-EYNKNNP-
82 Wiseana_iridescent_virus                       14.4%   GDY--GNLQPQYFLNEKGLHRATLGQYN-------VPFLTDEIKEEQGIQTFEEESNWYLDKLPAHIQLLE-KIKRRGQ-
83 Yaba_monkey_tumor_virus                        10.8%   HCN-----------------------YK--------------------------------SHDNPNMFLVN-EYNNNNA-
84 Yellowstone_lake_phycodnavirus_1               17.0%   ASD-----------------------YK---------------------------------VKMPHVEVRD-KIRARAPG
   consensus/100%                                         ................................................................................
   consensus/90%                                          t..    ..........................................................th.h.t th......
   consensus/80%                                          ts.               ........Yt            ....................st..sph.lsp phttps..
   consensus/70%                                          ps.                     ..YK                   .............st..sphhlsp chttps.

                                                   3281           .          3          .          .          .          .          :        .  33
 1 Acanthamoeba_castellanii_mamavirus            100.0%   N-KPQI-NDRIPFVYIVKDM--GKKKK-----KDILQGDLIEHPEYVI-ANNLKI-----DYLYYLEH--QIINPASQIL
 2 Acanthamoeba_polyphaga_mimivirus               98.6%   N-KPQI-NDRIPFVYIVKDM--GKKKK-----KDILQGDLIEHPEYVI-ANNLKI-----DYLYYLEH--QIINPASQIL
 3 Acanthamoeba_polyphaga_moumouvirus             56.0%   N-KPQI-NDRIPFVYIVKDM--GKKKK-----KDILQGDLIEHPDYVI-ANDLKI-----DYLYYLEH--QIINPATQIL
 4 Amsacta_moorei_entomopoxvirus                  10.1%   --KITK-GQRFDFIYAHKINEWSKDIKKWNIKYTIDISKHVIILEDYL-KNKNNYRI---CVEKYIK---DILSNLDQII
 5 Anomala_cuprea_entomopoxvirus                   9.7%   N-KIMK-GNRFSFVYGTEIS--K-WDKSYES-WNFKNTNDINQKIIII-GDDIKYDN---IIRICIEK--YIINLLNNIN
 6 ASFV_BAV71                                      9.7%   --EPEP-GERFSYVIVEKQVQFDIQGHRT---DSSRKGDKMEYVSEAK-AKNLPI-----DILFYINN--YVLGLCARFI
 7 ASFV_E75                                        9.7%   --EPEP-GERFSYVIVEKQV--QFDIQGHRT-DSSRKGDKMEYVSEAK-AKNLPI-----DILFYINN--YVLGLCARFI
 8 Brazilian_marseillevirus                       12.6%   --EVIP-NTRLEFVFLTVPD--KKKG------EKTLQAEQVEDFTWYI-DNKKRLGLKI-DTHLYLTK--KVMEPLAELL
 9 Cafeteria_roenbergensis_virus_BV-PW1           20.9%   N-KPQA-GDRISFAVVEVPP--K---------KGMLQGERIETPDYIK-ENNLTI-----DYLFYLTN--QIEKPALQFL
10 Cannes_8_virus                                 13.3%   --EVIP-NTRLEFVFLEVPG--AKKG------DKILQTEQIEDFTWYL-DNKKRLGLRI-DTDLYLDK--KLMKPIEELL
11 Catovirus_CTV1                                 24.3%   N-RPQS-NDRIEYVYRVVKG------------DVELQGDRVETPEFIK-ENNLSI-----DHLFYITN--QIMKPSIQFL
12 Cedratvirus_A11                                12.1%   --PINP-GDRIDYIITDKKS--------------TYLGEKMLSPEHFI-TDGAKV-----DYDYYLEH--FGIKKIEKLY
13 Choristoneura_biennis_entomopoxvirus           10.5%   --KIVK-GQRFDFIYAHEIDIWDIETKKWNTKYTSEITKYMVILEDYI-KDKNKYRI---CIEKYIK---DIISNLNQII
14 Common_midwife_toad_ranavirus                  13.2%   --TVAD-GTRLEYVVLRPSY--DGKKRRF---RGQGLSERLETSDYYK-RFAEFLEL---DTEHYVK---TLVNPLDQLL
15 Cowpox_virus                                   10.5%   --ETIELGERYYFAYICP-N--GPWTKKL---VNIKTYETIIDRSFKL-GSDQRI-----FYEVYFK---RLTSEIVNLL
16 Diadromus_pulchellus_ascovirus_4a              12.7%   --EKPE-GARMEYIVLNKR-------------NTKKQCDKIEHINEYR-NNRRVYDV---DYMYYIS---HLVDPLEQIF
17 Ectromelia_virus                               10.5%   --ETIELGERYYFAYICPAN--EPWTKKL---VNIKTYETIIDRSFKL-GSDQRI-----FYEVYFK---RLTSEIVNLL
18 Emiliania_huxleyi_virus_145                    14.6%   GVTPQP-GDRIPFVIRYDKH--A-----------KHICDRAEDLDYLR-ENNIPL-----DRLYYITN--KITKPILTIF
19 Emiliania_huxleyi_virus_86                     14.6%   GVTPQP-GDRIPFVIRYDK-------------HAKHICDRAEDLDYLR-ENNIPL-----DRLYYITN--KITKPILTIF
20 European_catfish_virus                         13.1%   --TVAD-GTRLEYVVLRPSY--DGKKRRF---RGQGLSERLETSDYYK-RFAEFLEL---DTEHYVK---TLVNPLDQLL
21 Faustovirus_D3                                  8.8%   --PPDA-GDKFKYVIVKRPE--KINQKGNK--EKMTKGDFMEYLDVYM-ASQAHEHPLELDLEWYFER--SFTTSFARFI
22 Faustovirus_E12                                 8.9%   --PPGA-GDKFKYVIVKRPE--KINQRGNK--EKTTKGDFMEYLDVYI-ASQSHAHPLELDLEWYFER--SFITSFSRFI
23 Faustovirus_E24                                 8.9%   --PPGA-GDKFKYVIVKRPE--KINQRGNK--EKTTKGDFMEYLDVYI-ASQSHAHPLELDLEWYFER--SFITSFSRFI
24 Faustovirus_E9                                  9.0%   --PPDA-GDKFKYVIVKRPE--KINQKGNK--EKMTKGDFMEYLDVYM-ASQAHEHPLELDLEWYFER--SFITSFARFI
25 Faustovirus_Liban                               9.0%   --PPGA-GDKFKYVIVKRPE--KINQRGNK--EKTTKGDFMEYLDVYI-ASQSHVHPLELDLEWYFER--SFITSFSRFI
26 Feldmannia_species_virus                       13.4%   -------NQKLYREIVRPVV--EQGGVPSLGTAYGVLEGVRRKFSF---DQRRDV-----SYEEFLRD--LANGRVAEKL
27 Frog_virus_3                                   13.2%   --TVAD-GTRLEYVVLRPSY--DGKKRRF---RGQGLSERLETSDYYK-RFAEFLEL---DTEHYVK---TLVNPLDQLL
28 Heliothis_virescens_ascovirus_3e               11.3%   --PAIE-GGRIEFLNIRISN---------------PDDLPIEEISYFK-EHGGIV-----DRLYYIK---QIINPLTKVS
29 Hokovirus_HKV1                                 20.3%   N-KPQI-GDRIQYAYIIKPN--SHK---------LKQGEIIENREYII-ENKLQL-----DYLVYITK--QIMNPTLQFL
30 Infectious_spleen_and_kidney_necrosis_virus    12.7%   --AVVS-GARIEYVVLKHQY--GV--------PEGALGARLLDFERWR-EMKVAYPL---DRLYYMK---SVVNACDQLL
31 Insectomime_virus                              12.3%   --EIIP-NTRLEFVFLTVPS--KKKG------EKTLQAEQVEDFTWYL-DNKKRLGLKI-DTHLYLTK--KVMEPIAELL
32 Invertebrate_iridescent_virus_22               13.4%   --SRNE-GSRLEYVVVETDN------------LKDKLSAKNETVMYYN-KNKGILNL---DYLYYLH---RLINPIDQIL
33 Invertebrate_iridescent_virus_3                14.2%   --MKNE-GGRLEYVIVETNS------------LKDKQSTKIETLPYYT-KNRGILKL---DYLYYLH---RCINPLDQIL
34 Kaumoebavirus                                  10.2%   DIEPPKIFQRFEYVVVEKPA--FTYNFKGKS-TKTALGDKFEYLSRVK-ANPHLFKI---DLDYYIER--EIIGKLSGFI
35 Klosneuvirus_KNV1                              15.8%   ----------------------------------------------------------------------KIVSAISK--
36 Kurlavirus_BKC-1                               12.6%   --EVIP-NTRLEFVFLDVPG--KKKG------EKVLQAEQVEDFTWYL-DNKKRLGLKI-DTHLYLSK--KVMEPLAELL
37 Lausannevirus                                  12.4%   --EVIP-NTRLEFVFLDIPG--KKKG------EKVLQADQVEDFTWYL-DNKKRLGLKI-DTHLYLSK--KVMEPLAELL
38 Lumpy_skin_disease_virus_NI-2490               10.5%   --EIIEIGERYYFAYICNKK--EPWQKKL---VNIKTYERIIDRSFKL-KSTERI-----FYEVYFK---RLATEIVNLL
39 Lymphocystis_disease_virus                     13.5%   --PVEN-GTRIEYVILKGKG---------------LLGDKMEYFDYYK-KRSRYLKL---DHLYYLK---NMINPLDQLL
40 Marseillevirus_marseillevirus_T19              13.3%   --EVIP-NTRLEFVFLEVPG--AKKG------DKILQTEQIEDFTWYL-DNKKRLGLRI-DTDLYLDK--KLMKPIEELL
41 Megavirus_chiliensis                           60.1%   N-KPQI-NDRIPFVYVVKDM--GNKKK-----KDILQGDLIEHPDYVI-QNNMKI-----DYLYYLEH--QIIKPATQIL
42 Megavirus_courdo7                              60.4%   N-KPQI-NDRIPFVYVVKDM--GNKKK-----KDILQGDLIEHPDYVI-QNNMKI-----DYLYYLEH--QIIKPATQIL
43 Megavirus_ursino                               60.1%   N-KPQI-NDRIPFVYVVKDM--GNKKK-----KDILQGDLIEHPDYVI-QNNMKI-----DYLYYLEH--QIIKPATQIL
44 Melbournevirus                                 13.3%   --EVIP-NTRLEFVFLEVPG--AKKG------DKILQTEQIEDFTWYL-DNKKRLGLRI-DTDLYLDK--KLMKPIEELL
45 Micromonas_pusilla_virus_SP1                   16.3%   S-EPQS-GDRVPYLLTKTGD------------PKARAFEKSEDPKFVE-EHDIPV-----DYHYYFQN--KFLNPVCDLL
46 Mollivirus_sibericum                            9.9%   G-EPQS-GSRVPYVTIIDP-------------KARSVSEQAEDTEWVV-QHKGTIKI---DRAYYVR---SLIRAFGQLL
47 Molluscum_contagiosum_virus_subtype_1           9.1%   --EPIELGERYYYAYVCASE--LPWQLAV---ANVRNHERIVDASYVL-PERERV---------FYEI--YFRRLATELV
48 Monkeypox_virus                                10.5%   --ETIELGERYYFAYICPAN--VPWTKKL---VNIKTYETIIDRSFKL-GSNQRI-----FYEVYFK---RLTSEIVNLL
```

```
49 Myxoma_virus                              10.7%   --ERIEIGERYYFAYLCDAK--DAWQKKL---VNIKTYERIIDKGFTL-GSTDRI-----FYEVYFK---RLATEVVNLL
50 Noumeavirus                               12.5%   --EVIP-NTRLEFVFLDVPG--KKKG------EKVLQAEQVEDFTWYL-DNKKRLGLKI-DTHLYLSK--KVMEPLAELL
51 Orf_virus                                 10.2%   --EAIEIGERYFFAYVCEEG------------PWRRRIANVKSYERVV-DKTFRLDK---NERIMYEV--YFKRLCTEIV
52 Organic_Lake_phycodnavirus_1              18.2%   --KPSP-GDRIPFVYIKNE-------------NKQLQGDKIETPDFIK-SEGLEI-----DYVFYITN--QIMKPIIQIY
53 Orpheovirus_LCC2                          12.8%   --PANP-GDRIPYIIVKHRN--GDIEESTG--KEFPLGKRMRSDEQYL-EALADGNAEQIDYIYYISK--LLVNPIEQLF
54 Ostreococcus_lucimarinus_virus_1          16.7%   S-EPQS-GDRVPYLLTKTEN------------PKAKAYEKAEDPKYVE-EHGVHV-----DYHYYFVN--KFLNPVCDLL
55 Ostreococcus_tauri_virus_OtV5             16.7%   S-EPQS-GDRVPYLLTKTQD------------PKAKAYEKAEDPKYVE-EHGVPV-----DYHYYFLN--KFLNPVCDLL
56 Pacmanvirus_A23                            9.1%   --PPEA-GDKFEYILVKKEQ--RFTLQGNK--IELKKGDQMEYLRVYK-ASQETSNPMEINLSYYMKN--SIVGLFARFI
57 Pandoravirus_dulcis                        9.2%   S-EPLA-GNRVYFVITIDE-------------RLKKKSARAEDPAYVA-ANPRLARI---DRLYYLQ---SLANPFGALL
58 Pandoravirus_inopinatum                    8.8%   -------GAGLEHVIQGALR--SGADLPARRPDAKPSVGKARVLAALSPVRDSRLSAACGAQRNYIARRDKLTARYDAMF
59 Pandoravirus_salinus                      10.5%   S-EPLA-GNRVYFVITIDD-------------RLKKKSARAEDPAYVA-ANPRLARI---DRLYYL-D--SLKNPFGALL
60 Paramecium_bursaria_Chlorella_virus_CVK2  15.4%   --FPVPSGARVPFVYIEDKK--N---------PDIKQSFKAEDPTFAR-DNDLVV-----DRLFYIEH--QLRKPICSLF
61 Paramecium_bursaria_chlorella_virus_MT325 15.1%   --FPVPSGARVPFVYVEDKD--N---------IDAKQSMRAEDPKYAM-DNGLIV-----DRLFYINH--QLLKPLTSLF
62 Paramecium_bursaria_Chlorella_virus_NYs1  14.2%   --FPVPSGARVPFVYIEDKK--N---------PDLKQSFRAEDPTFAQ-ENRLIV-----DRLFYIEH--QLRKPVCSLF
63 Phaeocystis_globosa_virus                 16.9%   N-KPGA-GDRMNYAYIKNPN------------KKALQGEKIETPEFIK-DNELKL-----DYGHYITN--QIMKPLLQLY
64 Phaeocystis_globosa_virus_14T             16.8%   N-KPGA-GDRMNYAYIKNPN------------KKALQGEKIETPEFIK-DNELKL-----DYGHYITN--QIMKPLLQLY
65 Pithovirus_massiliensis                   11.6%   --PANP-GDRLDYIIIQNDG--------------EYLGNKMMLPEHFL-QDNEEKKVEI-DYDYYIEH--FGIKNIEQLY
66 Pithovirus_sibericum                      11.8%   --PANP-GDRLDYIIVQNEG--------------QYLGNKMMLPEHFL-QDNEEKKVEI-DYDYYIEH--FGIKNIEQLY
67 Port-miou_virus                           12.4%   --EVIP-NTRLEFVFLDIPG--KKKG------EKVLQADQVEDFTWYL-DNKKRLGLKI-DTHLYLSK--KVMEPLAELL
68 Powai_lake_megavirus                      60.0%   N-KPQI-NDRIPFVYVVKDM--GNKKK-----KDILQGDLIEHPDYVI-QNNMKI-----DYLYYLEH--QIIKPATQIL
69 Scale_drop_disease_virus                  12.8%   --AVDK-GSRVEYVVLQSPR--GVLE--------ESLAQRIIDLETWK-DNKDIYPL---DRLYYVK---SMINACDQIF
70 Short-finned_eel_ranavirus                13.1%   --TVAD-GTRLEYVVLRPSY--DGKKRRF---RGQGLSERLETSDHYK-RFAEFLEL---DAEHYVK---TLVNPLDQLL
71 Singapore_grouper_iridovirus              13.3%   --TVAD-GTRLEYVVLKSPY--NGKKGRW---EDEGLSSRLETSDYYF-ANREFLKL---DVEHYVK---TLINPLDQLL
72 Skunkpox_virus                            11.0%   --TTIELGERYYFAYICPAS--EPWTKKL---VNIKTYETIIDGSFKL-GADQRI-----FYEVYFK---RLASEIVNLL
73 Spodoptera_frugiperda_ascovirus_1a        10.9%   --PPVE-GGRIEFLNVKTE---------------TKSALAIEEISYFK-DNPGCAQV---DNMYYIQ---QLVNPLTKVS
74 Testudo_hermanni_ranavirus                13.2%   --TVAD-GTRLEYVVLRPSY--DGRKRRF---RGQGLSERLETSDYYK-RFAEFLEL---DTEHYVK---TLVNPLDQLL
75 Tiger_frog_virus                          13.1%   --TVAD-GTRLEYVVLRPSY--DGKKRRF---RGQGLSERLETSDYYK-RFAEFLEL---DTEHYVK---TLVNPLDQLL
76 Tokyovirus_A1                             12.9%   --EVIP-NTRLEFVFLEVPG--AKKG------DKILQTEQIEDFTWYL-DNKKRLGLRI-DTGLYLDK--KLMKPIDELL
77 Trichoplusia_ni_ascovirus_2c              10.4%   --SAVE-GGRVEYLMVKRPG------------SKKSSASNIEEITYFK-ENSSIIQI---NELHYIL---QLVKPITKVC
78 Tunisvirus_fontaine2                      12.2%   --EIIP-NTRLEFVFLTVPS--KKKG------EKTLQAEQVEDFTWYL-DNKKRLGLKI-DTHLYLTK--KVMEPIAELL
79 Vaccinia_virus                            10.6%   --ETIELGERYYFAYICP-N--GPWTKKL---VNIKTYETIIDRSFKL-GSDQRI-----FYEVYFK---RLTSEIVNLL
80 Variola_virus                             10.3%   --ETIELGERYYFAYICPAN--VPWTKKL---VNIKTYETIIDRSFKL-GSDQRI-----FYEVYFK---RLTSEIVNLL
81 Volepox_virus                             10.8%   --TTIELGERYYFAYICPAS--EPWTKKL---VNIKTYETIIDGSFKL-GTDQRI-----FYEVYFK---RLASEIVNLL
82 Wiseana_iridescent_virus                  14.4%   --MCNE-GSRLEYVILETNN------------LKDKQSAKIETVNYFR-KNKGILNL---DYLYYLH---RLINPIDQIL
83 Yaba_monkey_tumor_virus                   10.8%   --EIIEIGERYFFAYICPSK--YPWQKKL---VNIKTYERIIDRRFKL-NQNDRI-----FYEVYFK---RIATEVVNLL
84 Yellowstone_lake_phycodnavirus_1          17.0%   S-EPQQ-GDRVQFVIVEGRG--------------RMFEKAEDPEWVK-TNGLKI-----DYEYYFGH--QLKKPVCDLL
   consensus/100%                                    .......................................................................h.t...t..
   consensus/90%                                     . .....st+h.ahhh....   ........  ......t.h...phhh ttt..h.... .h.hYhp.  .hhp.h.phh
   consensus/80%                                     . .s.. spRh.ashlp...   .......   .t.h..p.h.p.phhh tpt.hh.... sh.hYhp.  phhp.hsplh
   consensus/70%                                       ps.. GsRh.alhlp...   .......   .thhts-phEp.pahh tsp.hl..   Dh.hYlp.  plhpshspll

                                                 3361        .         .         .         4         .         .         .         . 34
 1 Acanthamoeba_castellanii_mamavirus       100.0%   EL------------------------------------------------------------MMDTKDVQKFFNKYI---
 2 Acanthamoeba_polyphaga_mimivirus          98.6%   EL------------------------------------------------------------MMDTKDVQKFFNKYI---
 3 Acanthamoeba_polyphaga_moumouvirus        56.0%   EL------------------------------------------------------------MIDSKSVMKLFNEYIIEE
 4 Amsacta_moorei_entomopoxvirus             10.1%   ----------------------------------------------------------------NDKNIIKNIDIML---
 5 Anomala_cuprea_entomopoxvirus              9.7%   ML-------------------------------------------------------------LNNKNIIKNIKDKY---
 6 ASFV_BAV71                                 9.7%   NENEEFQPPDNVSNKDEYAQRRAKSYLQKFV----------------------QSIHPKDKSVIKQGIVHRQCYKYV---
 7 ASFV_E75                                   9.7%   NENEEFQPPDNVSNKDEYAQRRAKSYLQKFV----------------------QSIHPKDKSVIKQGIVHRQCYKYV---
 8 Brazilian_marseillevirus                  12.6%   NIWEKKTIEYEK------------------------------LD---------ERIKRLKSLMSPEDKDCVTKLEFL---
 9 Cafeteria_roenbergensis_virus_BV-PW1      20.9%   KLAVPN------------------------------------VE---------KRLENLKIIRENEKKGYEDIYKYV---
10 Cannes_8_virus                            13.3%   DVWEKQTVPYEKLC---------------------------------------EKIKRLEGEVSEGDKDVVELLRNL---
11 Catovirus_CTV1                            24.3%   EL-----------------------------------------------------------LIDEPEKIFESYIVRE---
12 Cedratvirus_A11                           12.1%   NIAHGEEIEKRE------------------------------YV---------HRERCAYMALRDFLNLYPEYLETA---
13 Choristoneura_biennis_entomopoxvirus      10.5%   ----------------------------------------------------------------EDKDIINNIDNML---
14 Common_midwife_toad_ranavirus             13.2%   TT------------------------------------------------------------AGRPEDEFKAFYGYR---
15 Cowpox_virus                              10.5%   ----------------------------------------------------------------DNKVLCISFFERM---
16 Diadromus_pulchellus_ascovirus_4a         12.7%   SS------------------------------------------------------------IFKTEKFVEKSLKVF---
17 Ectromelia_virus                          10.5%   ----------------------------------------------------------------DNKVLCISFFERM---
18 Emiliania_huxleyi_virus_145               14.6%   QAFKEHIHDVQR------------------------------TI---------QSAMSRVQLQLDKQPTITSFFNKL---
19 Emiliania_huxleyi_virus_86                14.6%   QAFKEHIHDVQRTI---------------------------------------QSAMSRVQLQLDKQPTITSFFNKL---
20 European_catfish_virus                    13.1%   TT------------------------------------------------------------AGRPEDEFKAFYGYR---
21 Faustovirus_D3                             8.8%   CYHKDFAHPDLATGEIDPD-----------------------LDDTWRVYRATEWLKEQANQFSDKAARAEKMKKLQ---
22 Faustovirus_E12                            8.9%   CYHNSFAHPNPIDPDQDDEHRINSAT---------------------------EWLIEQANRFSDKEARAEKMKRLQ---
23 Faustovirus_E24                            8.9%   CYHNSFAHPNPIDPDQDDEHRINSAT---------------------------EWLIEQANRFSDKEARAEKMKRLQ---
24 Faustovirus_E9                             9.0%   CYYKDFAHPDIATGEVDPD-----------------------LDDTHRIEKATEWLIAQANQFSDKAARAEKMKKLQ---
25 Faustovirus_Liban                          9.0%   CYHKSFAHPNPIDPDQDDEHRINSAT---------------------------EWLIEQANRFSDKEARAEKMKRLQ---
26 Feldmannia_species_virus                  13.4%   KGSAPRVSECESL-----------------------------VG---------STEAKLIQEGIRTEKILNDMYREF---
27 Frog_virus_3                              13.2%   TT------------------------------------------------------------AGRPEDEFKAFYGYR---
28 Heliothis_virescens_ascovirus_3e          11.3%   EA------------------------------------------------------------VWKRSDTVIGAVTPI---
29 Hokovirus_HKV1                            20.3%   DLIDKNPERLFLKVIMIETNRRQKLR----------------PI---------TSFFKPKNTNDDDSDEESNFDKLS---
30 Infectious_spleen_and_kidney_necrosis_virus 12.7% VT-------------------------------------------------------------AGYGPVCSKVYAAH---
31 Insectomime_virus                         12.3%   NIWEKKVIEYEK------------------------------LD---------ERIKRLKSLISPEDKDCITKLEFL---
32 Invertebrate_iridescent_virus_22          13.4%   EV------------------------------------------------------------IFNLNDFMKNHYNYR---
33 Invertebrate_iridescent_virus_3           14.2%   KV------------------------------------------------------------VFDLDDFVKRQYKAR---
34 Kaumoebavirus                             10.2%   AYDDEFTKGLEQPDPNDTE----------------------------------ANNKYNKKLMDKAKKYLLEFFRTK---
35 Klosneuvirus_KNV1                         15.8%   --------------------------------------------------------------------------------
36 Kurlavirus_BKC-1                          12.6%   GIWEKRVIEYEK------------------------------LD---------ERIKRLKSLISPEDKDSVAKLEYL---
37 Lausannevirus                             12.4%   NIWEKKVIEYEKLD---------------------------------------ERIKRLKSLISPEDKDSLTKLEYL---
38 Lumpy_skin_disease_virus_NI-2490          10.5%   ----------------------------------------------------------------DNKVLSISFFEKM---
39 Lymphocystis_disease_virus                13.5%   EV------------------------------------------------------------SLNVKNFMKSVYDDR---
40 Marseillevirus_marseillevirus_T19         13.3%   DVWEKQTVPYEK------------------------------LC---------EKIKRLEGEVSEGDKDVVELLRNL---
41 Megavirus_chiliensis                      60.1%   ELMIDSRS----------------------------------VN---------KLFDEYKITEENKRKGRRSILEFV---
42 Megavirus_courdo7                         60.4%   ELMIDSRS----------------------------------VN---------KLFDEYKITEENKRKGRRSILEFV---
43 Megavirus_ursino                          60.1%   ELMIDSRS----------------------------------VN---------KLFDEYKITEENKRKGRRSILEFV---
44 Melbournevirus                            13.3%   DVWEKQTAPYEK------------------------------LC---------EKIKRLEGEVSEGDKDVVELLRNL---
45 Micromonas_pusilla_virus_SP1              16.3%   EPLFE----------------------------------------------------------NTKQEIFGEIIDQH---
46 Mollivirus_sibericum                       9.9%   GTLYDGIEIYIERAAELCEQTAAGQSVMFSESGMASKGKGRVFALTRQEALARLRLFSCVVTARDQTKPYIPFEERA---
47 Molluscum_contagiosum_virus_subtype_1      9.1%   NL-------------------------------------------------------------LDNKPLCAEFFTRL---
48 Monkeypox_virus                           10.5%   ----------------------------------------------------------------DNKVLCISFFQRM---
49 Myxoma_virus                              10.7%   ----------------------------------------------------------------DNKVLSTSFFEKM---
50 Noumeavirus                               12.5%   GIWEKRVIEYEKLD---------------------------------------ERIKRLKSLISPEDKDSVAKLEYL---
51 Orf_virus                                 10.2%   NL-------------------------------------------------------------LDNKAMCTLFFERL---
52 Organic_Lake_phycodnavirus_1              18.2%   SL----------------------------------------VL---------NDMKCFQRRKPSFIQEVETLSKNE---
53 Orpheovirus_LCC2                          12.8%   HVGYKKHVDHMVNSG---------------------------LG---------YKPFNNHCRFIPVDRPIKMITKMM---
54 Ostreococcus_lucimarinus_virus_1          16.7%   DPLYEN---------------------------------------------------------VKEEIFGEIINQHK---
55 Ostreococcus_tauri_virus_OtV5             16.7%   DPLYEN---------------------------------------------------------VKEDIFGEIINAHK---
56 Pacmanvirus_A23                            9.1%   AYHPRFQPPEGTYNCEDKEQYKEMDKHC--------------VN---------EATKYLNDLCDSITGFNKGELTQR---
57 Pandoravirus_dulcis                        9.2%   EPCFENPQ---------------------------------------------QLFADAAVFIANQQRGQAPITQWM---
58 Pandoravirus_inopinatum                    8.8%   AANTGKCRTAPD------------------------------AE---------EVRSAKWMWSWVWRRLHPSRLRLL---
59 Pandoravirus_salinus                      10.5%   EPCFDNPEQLFA---------------------------------------------DAAVFIANQQKGQAPITQWM---
60 Paramecium_bursaria_Chlorella_virus_CVK2  15.4%   EPLLDDP----------------------------------------------DKEIFEHPSLKSKIDALKNIFKAD---
61 Paramecium_bursaria_chlorella_virus_MT325 15.1%   EPLVDHPE---------------------------------------------KELFGHVDVVGKIEALTTRHKAEL---
62 Paramecium_bursaria_Chlorella_virus_NYs1  14.2%   EPLLDDP----------------------------------------------EKEIFGHPLIKEKIDRLKSTFKDD---
```

```
63 Phaeocystis_globosa_virus                   16.9%   ALELENIQEFKDKQ----------------------------FN---------IKEYNTDKKVILWEEEIIKLKEKW---
64 Phaeocystis_globosa_virus_14T               16.8%   ALELENIQEFKDKQ----------------------------FN---------IKEYNTDKKVILWEEEIIKLKEKW---
65 Pithovirus_massiliensis                     11.6%   QIAHKEQIEEYENYHIE------------------------------------FSAKYALQQFLNAFPQYVGLLQYQ---
66 Pithovirus_sibericum                        11.8%   QIAHKEQIEQHEHFH---------------------------VE---------FSAKYALQQFLTAFPQYVGLLQYQ---
67 Port-miou_virus                             12.4%   NIWEKKVIEYEKLD---------------------------------------ERIKRLKSLISPEDKDSLTKLEYL---
68 Powai_lake_megavirus                        60.0%   ELMIDSRS----------------------------------VN---------KLFDEYKITEENKRKGRRSILEFV---
69 Scale_drop_disease_virus                    12.8%   SVIGSTHVTHML--------------------------------------------------YMVHKQFCQVMSELR---
70 Short-finned_eel_ranavirus                  13.1%   TT------------------------------------------------------------AGRPEDEFKAFYGYR---
71 Singapore_grouper_iridovirus                13.3%   ET------------------------------------------------------------ACKGEGGVKAVHAAR---
72 Skunkpox_virus                              11.0%   ----------------------------------------------------------------DNKVLCISFFERM---
73 Spodoptera_frugiperda_ascovirus_1a          10.9%   EA------------------------------------------------------------VWRRSNSVNIAIAPF---
74 Testudo_hermanni_ranavirus                  13.2%   TT------------------------------------------------------------AGRPEDEFKAFYGYR---
75 Tiger_frog_virus                            13.1%   TT------------------------------------------------------------AGRPEDEFKAFYGYR---
76 Tokyovirus_A1                               12.9%   NVWEKQTIPYEK------------------------------LC---------EKIKRLEGEVSDGDKDVVDLLRNL---
77 Trichoplusia_ni_ascovirus_2c                10.4%   EA------------------------------------------------------------VWERSDIVVDAIKPI---
78 Tunisvirus_fontaine2                        12.2%   NIWEKKVIEYEK------------------------------LD---------ERIKRLKSLISPEDKDCITKLEFL---
79 Vaccinia_virus                              10.6%   ----------------------------------------------------------------DNKVLCISFFERM---
80 Variola_virus                               10.3%   ----------------------------------------------------------------DNKVLCISFFERM---
81 Volepox_virus                               10.8%   ----------------------------------------------------------------DNKVLCISFFERM---
82 Wiseana_iridescent_virus                    14.4%   EV------------------------------------------------------------IFGLKDFMKSIYMNR---
83 Yaba_monkey_tumor_virus                     10.8%   ----------------------------------------------------------------DNKVLSTSFFERM---
84 Yellowstone_lake_phycodnavirus_1            17.0%   EPLVGGDPEKVI--------------------------------------------------FAPKVKTMTDFFSLR---
   consensus/100%                                      ................................................................................
   consensus/90%                                       ...................                       ..         .............t.....h.t..
   consensus/80%                                       t.............                            ..         ...........p.p..h.thhphh
   consensus/70%                                       ph..........                              ..         ...........ptct.hhthhphh

                                                    3441      ] 3450
 1 Acanthamoeba_castellanii_mamavirus         100.0%   ----IDEQNK
 2 Acanthamoeba_polyphaga_mimivirus            98.6%   ----IDEQNK
 3 Acanthamoeba_polyphaga_moumouvirus          56.0%   ENKRKGRRSI
 4 Amsacta_moorei_entomopoxvirus               10.1%   ----NSYEPQ
 5 Anomala_cuprea_entomopoxvirus                9.7%   ----KKNN--
 6 ASFV_BAV71                                   9.7%   ---HQEIKKK
 7 ASFV_E75                                     9.7%   ---HQEIKKK
 8 Brazilian_marseillevirus                    12.6%   ----FRKTKL
 9 Cafeteria_roenbergensis_virus_BV-PW1        20.9%   ----NMNNMI
10 Cannes_8_virus                              13.3%   ----FRKRNM
11 Catovirus_CTV1                              24.3%   ----ENRRKG
12 Cedratvirus_A11                             12.1%   ----QAKEDY
13 Choristoneura_biennis_entomopoxvirus        10.5%   ----KNYEPV
14 Common_midwife_toad_ranavirus               13.2%   ----ANYRKV
15 Cowpox_virus                                10.5%   ----FGSKPT
16 Diadromus_pulchellus_ascovirus_4a           12.7%   ----EYKNKN
17 Ectromelia_virus                            10.5%   ----FGSRPT
18 Emiliania_huxleyi_virus_145                 14.6%   ----PPLPVI
19 Emiliania_huxleyi_virus_86                  14.6%   ----PPLPVI
20 European_catfish_virus                      13.1%   ----ANYRKV
21 Faustovirus_D3                               8.8%   ----AAYRRV
22 Faustovirus_E12                              8.9%   ----SAYRRV
23 Faustovirus_E24                              8.9%   ----SAYRRV
24 Faustovirus_E9                               9.0%   ----SAYRRV
25 Faustovirus_Liban                            9.0%   ----SAYRRV
26 Feldmannia_species_virus                    13.4%   ----SVFDRM
27 Frog_virus_3                                13.2%   ----VNYRKV
28 Heliothis_virescens_ascovirus_3e            11.3%   ----LNYDKV
29 Hokovirus_HKV1                              20.3%   ----NIKTPL
30 Infectious_spleen_and_kidney_necrosis_virus 12.7%   ----LQLAYV
31 Insectomime_virus                           12.3%   ----FRKTNL
32 Invertebrate_iridescent_virus_22            13.4%   ----VLKKKL
33 Invertebrate_iridescent_virus_3             14.2%   ----EAKKKV
34 Kaumoebavirus                               10.2%   ----FGNSAV
35 Klosneuvirus_KNV1                           15.8%   ----------
36 Kurlavirus_BKC-1                            12.6%   ----FRKQKL
37 Lausannevirus                               12.4%   ----FRKQKL
38 Lumpy_skin_disease_virus_NI-2490            10.5%   ----FGTRPI
39 Lymphocystis_disease_virus                  13.5%   ----KKYDAV
40 Marseillevirus_marseillevirus_T19           13.3%   ----FRKRNM
41 Megavirus_chiliensis                        60.1%   ----PDSKKK
42 Megavirus_courdo7                           60.4%   ----PDSKKK
43 Megavirus_ursino                            60.1%   ----SDSKKK
44 Melbournevirus                              13.3%   ----FRKRNM
45 Micromonas_pusilla_virus_SP1                16.3%   ---KPPKKKK
46 Mollivirus_sibericum                         9.9%   ---KTLKKAK
47 Molluscum_contagiosum_virus_subtype_1        9.1%   ----FGVRPV
48 Monkeypox_virus                             10.5%   ----FGSRPT
49 Myxoma_virus                                10.7%   ----FGTRPI
50 Noumeavirus                                 12.5%   ----FRKQKL
51 Orf_virus                                   10.2%   ----FGSKPV
52 Organic_Lake_phycodnavirus_1                18.2%   ----FDTDKR
53 Orpheovirus_LCC2                            12.8%   ----EEKRVD
54 Ostreococcus_lucimarinus_virus_1            16.7%   ----PVKPPK
55 Ostreococcus_tauri_virus_OtV5               16.7%   ----PVKPPK
56 Pacmanvirus_A23                              9.1%   ---GRDYRKI
57 Pandoravirus_dulcis                          9.2%   ----GGKRAA
58 Pandoravirus_inopinatum                      8.8%   ---LRGLRMA
59 Pandoravirus_salinus                        10.5%   ----GGKRAA
60 Paramecium_bursaria_Chlorella_virus_CVK2    15.4%   ----LKTAKR
61 Paramecium_bursaria_chlorella_virus_MT325   15.1%   ----KDTKRV
62 Paramecium_bursaria_Chlorella_virus_NYs1    14.2%   ----IKVAKR
63 Phaeocystis_globosa_virus                   16.9%   ----PDPEKY
64 Phaeocystis_globosa_virus_14T               16.8%   ----PDPEKY
65 Pithovirus_massiliensis                     11.6%   ----STWKAT
66 Pithovirus_sibericum                        11.8%   ----PTWKAT
67 Port-miou_virus                             12.4%   ----FRKQKL
68 Powai_lake_megavirus                        60.0%   ----PDSKKK
69 Scale_drop_disease_virus                    12.8%   -----RKRPL
70 Short-finned_eel_ranavirus                  13.1%   ----TNYRKV
71 Singapore_grouper_iridovirus                13.3%   ----VAYRKV
72 Skunkpox_virus                              11.0%   ----FGSRPT
73 Spodoptera_frugiperda_ascovirus_1a          10.9%   ----IYYNKV
74 Testudo_hermanni_ranavirus                  13.2%   ----VNYRKV
75 Tiger_frog_virus                            13.1%   ----VNYRKV
76 Tokyovirus_A1                               12.9%   ----FRKQNM
```

```
77 Trichoplusia_ni_ascovirus_2c              10.4%      ----CAYSKV
78 Tunisvirus_fontaine2                      12.2%      ----FRKTNL
79 Vaccinia_virus                            10.6%      ----FGSKPT
80 Variola_virus                             10.3%      ----FGSRPT
81 Volepox_virus                             10.8%      ----FGSRPT
82 Wiseana_iridescent_virus                  14.4%      ----VLKKKL
83 Yaba_monkey_tumor_virus                   10.8%      ----FGTKPI
84 Yellowstone_lake_phycodnavirus_1          17.0%      ----PAPKVE
   consensus/100%                                       ..........
   consensus/90%                                            ...p.h
   consensus/80%                                            .t.p.h
   consensus/70%                                            .thc.h
```
